# The Baltic Sea Atlantis: An integrated end-to-end modelling framework evaluating ecosystem-wide effects of human-induced pressures

**DOI:** 10.1371/journal.pone.0199168

**Published:** 2018-07-20

**Authors:** Sieme Bossier, Artur P. Palacz, J. Rasmus Nielsen, Asbjørn Christensen, Ayoe Hoff, Marie Maar, Henrik Gislason, François Bastardie, Rebecca Gorton, Elizabeth A. Fulton

**Affiliations:** 1 National Institute of Aquatic Resources, Technical University of Denmark, Lyngby, Denmark; 2 International Ocean Carbon Coordination Project, Institute of Oceanology of the Polish Academy of Sciences, Sopot, Poland; 3 Department of Food and Resource Economics, Copenhagen University, Copenhagen, Denmark; 4 Department of Bioscience, Aarhus University, Roskilde, Denmark; 5 CSIRO Oceans & Atmosphere, Hobart, Australia; 6 Centre for Marine Socioecology, University of Tasmania, Battery Point, Tasmania, Australia; Havforskningsinstituttet, NORWAY

## Abstract

Achieving good environmental status in the Baltic Sea region requires decision support tools which are based on scientific knowledge across multiple disciplines. Such tools should integrate the complexity of the ecosystem and enable exploration of different natural and anthropogenic pressures such as climate change, eutrophication and fishing pressures in order to compare alternative management strategies. We present a new framework, with a Baltic implementation of the spatially-explicit end-to-end Atlantis ecosystem model linked to two external models, to explore the different pressures on the marine ecosystem. The HBM-ERGOM initializes the Atlantis model with high-resolution physical-chemical-biological and hydrodynamic information while the FISHRENT model analyses the fisheries economics of the output of commercial fish biomass for the Atlantis terminal projection year. The Baltic Atlantis model composes 29 sub-areas, 9 vertical layers and 30 biological functional groups. The balanced calibration provides realistic levels of biomass for, among others, known stock sizes of top predators and of key fish species. Furthermore, it gives realistic levels of phytoplankton biomass and shows reasonable diet compositions and geographical distribution patterns for the functional groups. By simulating several scenarios of nutrient load reductions on the ecosystem and testing sensitivity to different fishing pressures, we show that the model is sensitive to those changes and capable of evaluating the impacts on different trophic levels, fish stocks, and fisheries associated with changed benthic oxygen conditions. We conclude that the Baltic Atlantis forms an initial basis for strategic management evaluation suited for conducting medium to long term ecosystem assessments which are of importance for a number of pan-Baltic stakeholders in relation to anthropogenic pressures such as eutrophication, climate change and fishing pressure, as well as changed biological interactions between functional groups.

## Introduction

### Baltic Sea ecosystem dynamics & pressures addressed in the Baltic Atlantis implementation

Understanding and quantifying the space- and time-varying intensity of human pressures and the resulting responses of marine ecosystems are essential for evaluating the impacts of human activities on the future provision of the goods and services we derive from the oceans [1–4]. The Baltic Sea ecosystem is subject to many interconnected and area specific pressures originating from natural changes and human activities (e.g. [5–8]), and human pressures are increasing [9]. An initial holistic assessment of ecosystem health for 2003–2007 indicated that the majority of the Baltic Sea was in a state of "poor" or "bad" health [10]. The area is geographically peripheral with respect to marine and freshwater conditions and low winter temperatures, and some ecosystem groups are therefore vulnerable to environmental changes [11–12]. Since the early 2000s, surface temperature has increased from an overall average of around 7°C to 9°C [13] and salinity has decreased with varying magnitude across the basins [11, 14]. The Western Baltic Sea is characterized as a transition zone with temperature and salinity conditions that vary substantially both seasonally and annually depending on the magnitude of saline inflows [11, 14]. Eutrophication and pollution, partly due to run-off from land based activities, as well as ocean acidification and climate change induce environmental impacts in the Baltic Sea [5, 6, 9, 10, 11]. However, so do fisheries, transport/shipping, renewable energy exploitation, gravel extraction, tourism, etc. These are other anthropogenic pressures that induce environmental impacts and also have socio-economic importance [5, 7–9]. Some of these pressures can be managed on national or local levels such as energy platforms, but several require a regionally, basin-wide integrated or even globally coordinated management approach because they have a wide spread distribution and are diffusive activities, e.g. fisheries and nutrient loads [10–11, 15–19]. For example, despite measures taken to reduce national inputs of nitrogen and phosphorus to the whole Baltic Sea, up to 18% from 1994 to 2010, the system is still characterized as highly eutrophic [10, 16]. As the system doesn’t seem to recover, the Baltic Sea eutrophication problem is therefore increasing [20].

### Challenges for Baltic Sea ecosystem models and the evaluation of natural and anthropogenic pressures

Besides fishery, eutrophication is a strong pressure on the Baltic Sea ecosystem. Studies of the biological effects of eutrophication in the Baltic Sea are numerous. Significant effects on primary production, both total area production and distribution in the water column, phytoplankton biomass, plankton community composition, water clarity and oxygen conditions have been documented (e.g. [21–22]). In [23], modelling simulations exploring the potential effects of changing oxygen and nutrient load conditions on the benthic community in the Baltic Sea have been conducted, showing significantly changes in the biogeochemical functioning of the ecosystem. Direct effects of eutrophication on fish stock recruitment is not evidenced by a number of correlative studies and a literature review ([24], and references therein). The meta-analyses in [24] finds only two fish stocks in the Baltic Sea and the North Sea where the recruitment dynamics can be related to changes in nutrient levels (Kattegat and Eastern Baltic cod). Accordingly, while there in the Baltic may be links between eutrophication and changes in fish stocks and fisheries the full chain of causality is yet to be documented. In contrast, a decrease in abundance of Baltic cod due to climate change (sea water temperature) has been documented (e.g. [25]). The complexity of the interactions also likely means that the direction of change is not uniform across species and/or regions [2, 26–28]. In general, the main challenge in studies of complex systems is to find occurrences where one pressure at a time varies, all other being equal, which is usually impossible given the variety of human activities (e.g. [2, 8]), as well as to integrate the ecosystem complexity adequately. Capturing this complexity is relevant in supporting different national and international policies and directives—such as the Marine Strategy Framework Directive (EU MSFD), the Water Framework Directive (EU WFD), the Baltic Sea Action Plan (BSAP) and the Maritime Spatial Planning Directive (EU MSP).

To consider several ecosystem drivers simultaneously, a possibility is to apply models that integrate a wide range of pressures and processes in a common framework that enables holistic evaluation [2, 7–8]. To construct such models has several advantages: they tend to increase understanding of system dynamics within and across ecosystems, help identify major processes, drivers and responses, highlight major gaps in knowledge, and inform future monitoring schemes [2, 29]. Furthermore, the final models make it possible to test management strategies virtually before they are implemented in reality [29]. These so-called end-to-end or whole-of ecosystem models [30], enable simulations of ecosystem dynamics and interactions from plankton to humans. Previous ecosystem modelling attempts in the Baltic include the EcoSim-EcoPath (EwE) modelling framework [31–33]. [34] used this framework to analyze trophic cascades and regimes shifts under scenarios of altered top-predator pressure as well as eutrophication (via assumed shifts in primary production). However, EwE has not been applied to the Western Baltic Sea, and explicit testing of eutrophication scenarios with EwE and its spatially-resolved Ecospace module would require linking with coupled physical-plankton models (e.g. POLCOMS-ERSEM) or with dedicated eutrophication models (e.g. Corps of Engineers Integrated Compartment Water Quality Model in Chesapeake Bay; [35]). Another approach for testing ecosystem effects of eutrophication in the Baltic Sea involved linking the HBM-ERGOM bio-geo-chemical model (see below) and the Stochastic Multi Species (SMS) model (see below), providing a novel platform for operational ecology as part of the FP7 Marine OpEc project (http://marine-opec.eu/).

### Moving towards a holistic management evaluation platform

To construct a holistic management evaluation platform, a new whole-of ecosystem and cyclic full-feed-back Atlantis model [36, 29] is here implemented covering the whole Baltic Sea, including the Western Baltic Sea and Kattegat. The Atlantis model [29] is designed as a tool capable of providing medium to long term management strategy evaluation, which has broad relevance and interest to many stakeholder groups. It is not, on the other hand, meant to provide short term accurate forecasts and, thus, should not be used for generating short term absolute and tactical advice [37]. Besides the key parameters and processes relevant for a specific region, the model also includes complex interactions between the different processes and actors. It consists of a hydrographic, a species community and a fisheries sub-model which are all interlinked with one another on a spatially (horizontal/vertical) and seasonally explicit scale. Besides its potential to meet the challenge of implementing an ecosystem-based approach to fisheries management [38], the fast development in a global perspective of the Atlantis model to integrate new marine sectors offers opportunities for balancing conflicting user objectives in marine resource management (e.g. [39–40]). In this context, the different implementations of Atlantis across a number of marine ecosystems around the world has been evaluated in many peer-reviewed publications and technical reports (e.g. [29, 41–51]) and this also increases relevance of a Baltic implementation for comparative purposes. The model has previously been successfully applied to support strategic decision making around marine resources and to optimize the marine monitoring of e.g. Australian and the United States [29]. Lately, the implementation of Atlantis in European and North-Eastern Atlantic regional seas has been conducted in several areas such as the Sicily Straits, English Channel [52], Nordic and Barents Seas [53], North Sea (EU-FP7-VECTORS, http://www.marine-vectors.eu/) and the Baltic Sea (current study).

**Fig 1:**
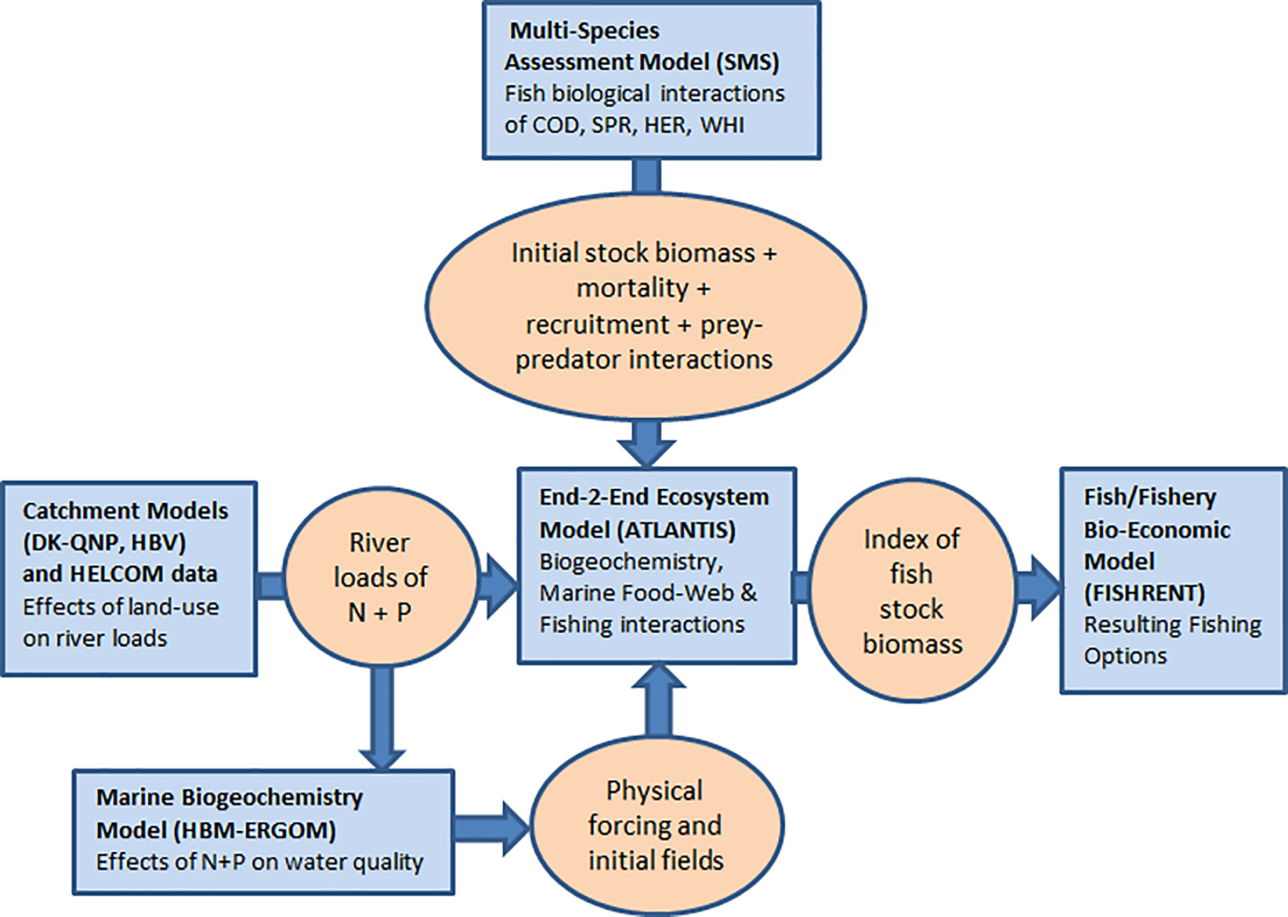
A schematic diagram of the integrated ecosystem modelling and strategic management evaluation framework. The diagram illustrates the current linking within the integrated modelling framework for investigating ecosystem-wide effects of human-induced pressures in the Baltic Sea.

Although the Atlantis model is the core of the present approach, some more detailed information, which will improve this holistic management evaluation platform, is more extensively available through other existing models. The HBM-ERGOM provides the Baltic Atlantis with high-resolution physical-chemical-biological and hydrodynamic information of the calibration year (2005) and the Atlantis output from the terminal projection year is processed with the FISHRENT fisheries economic model. A schematic overview of the models and how they are linked with the Baltic Atlantis is shown in Fig 1 and further described below.

The Baltic Atlantis implementation will become one of the most comprehensive integrated, dynamic, and spatially explicit bio-economic modelling tools of a marine system available in this region at present. In this paper, we present the implementation and parametrization of the Baltic Atlantis (using revision number 6191 of the main release of the software). [54]. We demonstrate the functionality of the model by evaluating effects of nutrient load reductions in the Kattegat and Western Baltic ecosystems, as well as pan-Baltic, including their effects on different trophic levels, fish stock biomasses and associated fisheries with consideration on changed benthic oxygen conditions. The purpose is accordingly to make a first evaluation of the effects of different scenarios (and associated assumptions) of eutrophication pressure reductions, and to test sensitivity to changed fishing pressure for key exploited fish stocks with fisheries importance across the Baltic Sea.

## Methods

### Setting up a Baltic ecosystem end-to-end modelling and management strategy evaluation framework

We present a new framework, with a Baltic implementation of the spatially-explicit end-to-end Atlantis ecosystem model linked to two external models, to explore the different pressures on the marine ecosystem (Fig 1). Atlantis is used as the main and central part of a 3 part linked model system.

The first link is between a biogeochemical-hydrodynamic model and Atlantis. Detailed input for the hydrographic part of the Baltic Atlantis model is provided by the HBM-ERGOM model system (Fig 1) consisting of the 3D ocean circulation Hiromb-BOOS model (HBM) coupled to the biogeochemical and plankton production ERGOM model [55–57]. This HBM-ERGOM model is in turn informed by the catchment models DK-QNP [58] and HBV [59] which deliver high spatial resolution information (1×1 km) on river discharge and nutrient loads. For the Danish areas, both the Atlantis model and the HBM-ERGOM model receive river data from the catchment model DK-QNP, for the other Baltic Sea rivers, the catchment model HBV or HELCOM data is used.

The second link is between Atlantis and a bio-economic fisheries model. The Atlantis output of the terminal projection year feeds into the FISHRENT model (Fig 1), which is a bio-economic multi-fish-stock-multi-fishing-fleet-model for Kattegat and the Western Baltic fishery (FISHRENT KWB henceforth; [60]).

The way the three models (HBM-ERGOM–Atlantis–FISHRENT) are connected is based on a one-way offline linking. Information is fed from one model to another without accounting for any potential dynamic coupling or feedback processes. Information about hydrodynamics obtained from the HBM-ERGOM model is passed on as input to the Baltic Atlantis for the 2005 calibration year, and information about changes in biological production obtained from the Baltic Atlantis is, with respect to fish species biomasses, passed on as input to the FISHRENT KWB model, as illustrated in Fig 1. A detailed explanation on how this is exactly done, can be found in sections A and C in S1 File. The outcomes of the Baltic fish Stochastic Multispecies Model (SMS; [61]) are also used to provide information on natural fish mortality levels and constrain dietary interactions between the fish species sprat, herring and cod. Furthermore, a couple of other models can be associated with the framework but are not currently used and described in Fig 1 (i.e., the DISPLACE [62–63] model, coupled to the SMS models [64], and the broader socio-economic CBA model, an ecosystem services assessment tool [65]).

### Initialization, forcing and parameterization of the Baltic Atlantis

Year 2005 is chosen as the initial conditions year for the Baltic Atlantis calibration because it is the most recent year with the greatest overlap of available information between all the biological variables and parameters used in the Baltic Atlantis model. Furthermore, year 2005 is characterized by lack of extreme events, in either physical forcing (river discharge, Baltic inflow) or significant ecosystem changes. In absence of data or other model outputs from that period for a given variable or parameter, the temporal search radius is extended to one year before and after 2005 until the closest estimate in time is found. Spatially averaged data were then averaged per quarter whenever data was available at a seasonal resolution. The process of informing the large number of biological parameters in the Baltic Atlantis model was based on an extensive review of literature to minimize the number of poorly constrained parameters. In a majority of cases, previous estimates of equivalent model parameters were already reported (e.g. length at infinity, mortality rate, recruitment constants), or it was possible to derive first order approximations from available data (e.g. survival rates of juvenile mammals). However, there were also a few Atlantis-specific parameters which had to be provided. The process to find these is described further down.

#### Input from HBM-ERGOM

The HBM-ERGOM model (Figure A in S1 File) provides physical, chemical and hydrodynamic parameters to the Baltic Atlantis for the 2005 calibration year. It is a 3D ecosystem model coupling an ocean circulation model with a biogeochemical module describing the major pathways for carbon, nitrogen, phosphorous and silica. It is driven by atmospheric forcing and nutrient loads (N, P, Si), and simulates the responses to climate changes and changing nutrient loadings. The HBM drives the transport of physical and biogeochemical properties while the biogeochemical model ERGOM simulates primary production and cycling of nutrients (N, P, Si) through 3 phytoplankton groups, 2 zooplankton groups, detritus and the sediment [56, 66]. ERGOM has been described and validated in previous studies [56–57, 60] based on the original formulations [21, 55]. More detailed information about the HBM-ERGOM model can be found in section A in S1 File.

**Fig 2:**
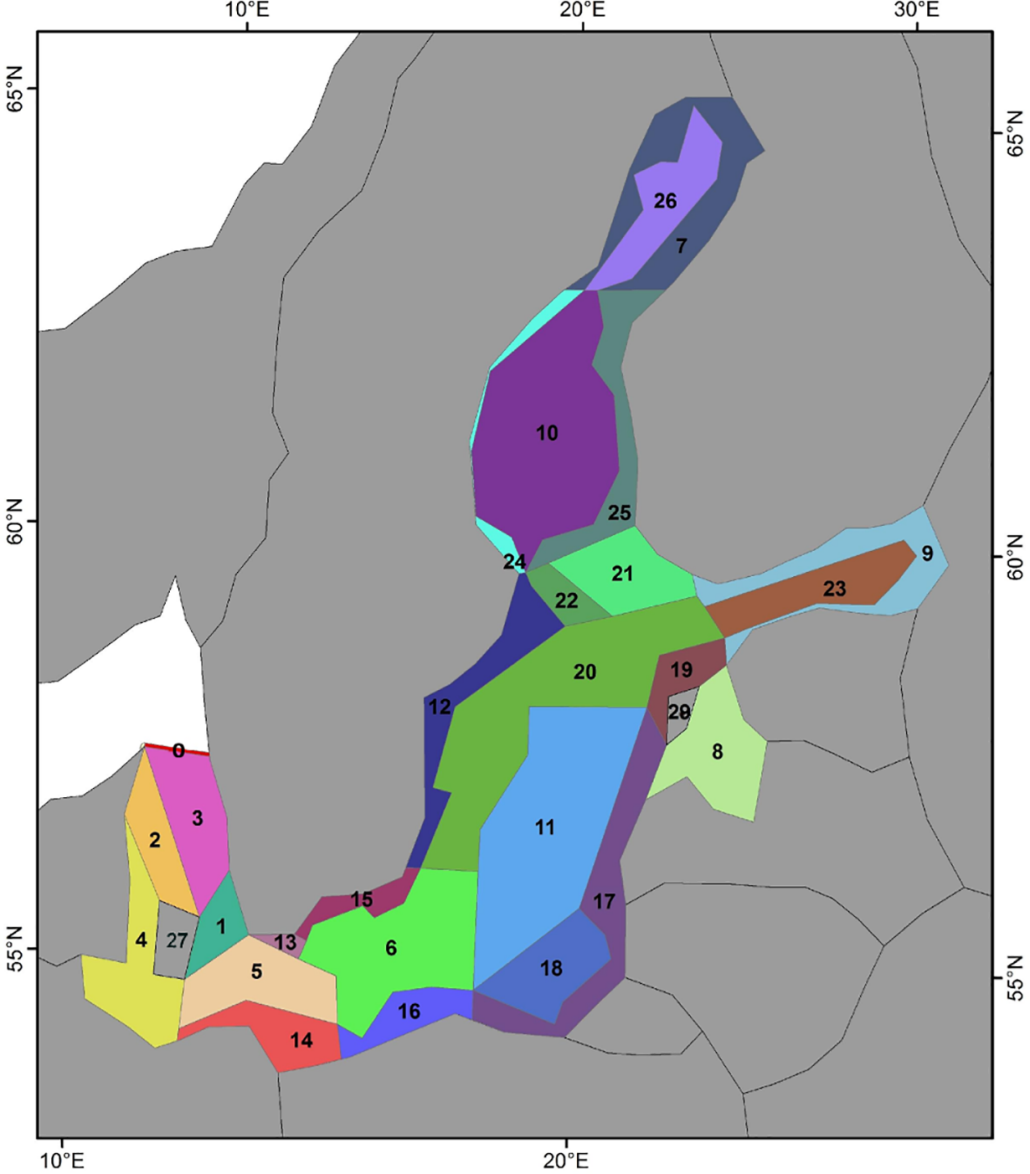
Baltic Atlantis spatial polygon structure.

The next parameters described are set values for a longer time period. A first group is the temperature, salinity, exchange volume (i.e. currents) and the external tracers for the dynamic boundary Box 0 (Fig 2)–representing the entire transport between Baltic Sea and North Sea. They are parameterized with 12h time steps for 2005, which is repeated for each projection year in Atlantis. Temperature and salinity values are fields which are calculated per box and per depth layer, while the currents are fluxes, calculated per box face. The second group consists of the tracers of atmospheric deposition fluxes and riverine inputs. These forced sources are annually averaged, but applied daily at even rates. Atmospheric deposition rate of nutrients is fixed in time and is a spatially-uniform estimate of 0.15 mmol-NH4/m^2^/day following [68], in line with atmospheric sources used in HBM-ERGOM. Even though these parameters are provided by the HBM-ERGOM model, there is no cyclic feedback between HBM-ERGOM and Atlantis in the projection. Accordingly, the biogeochemical cycling is explicitly modelled in the Baltic Atlantis, with nitrogen being the currency of the model while silicate and oxygen fields and fluxes are also tracked. Physical and geochemical parameters used to internally force the Baltic Atlantis model can be found in Table A in S1 File.

Annual river run-off and nitrogen deposition flux estimates, derived from the Review of the Fifth Baltic Sea Pollution Load Compilation for the 2013 HELCOM Ministerial Meeting [69], are used to calculate the annual rates of nitrogen riverine inputs. The fluxes are translated onto the Atlantis box model domain, combined with coastal retention and bioavailability factors estimated regionally in the Baltic Sea basin [70–72] to account for estuarine and near-coastal transformation and removal of nutrients. Parts of some of the fluxes have been put directly into the off-shore boxes to account for a lack of resolution of fine scale (and potentially complex) eddies and plumes that extend horizontal mixing of these tracers out beyond coastal waters. The estimates of per-box riverine inputs of nitrate and dissolved organic nitrogen used to force the model are summarized in Table B in S1 File. Additionally, there are two important nutrient sinks for nitrogen in the model: (i) explicitly modeled denitrification and (ii) implicitly considered decay and sediment burial. Details of how these processes are parameterized can be found in the S1 File (section B.1).

Besides the hydrographic parameters, the HBM-ERGOM also provided the information about initial condition values, more specifically for the winter 2005 initial conditions of nutrients (nitrate (NO_3_), ammonia (NH_4_), silicate (SiO_4_), dissolved oxygen (O_2_), detrital material (dissolved organic nitrogen (DON) + detrital silica + particulate organic matter: labile and refractory detritus), primary (diatoms, autotrophic flagellates, cyanobacteria) and secondary pelagic producers (microzooplankton, mesozooplankton).

#### Other initial condition values

The annual average spatial distribution of mysids and gelatinous zooplankton was based on scarce and irregular data maintained in SMHI (Swedish Meteorological and Hydrographical Institute; www.smhi.se) and ICES (International Council for Exploration of the Sea; www.ices.dk) databases, and supplemented by the results of an extensive literature review, covering a period from 2003 to 2007. Initial conditions for benthic deposit feeders, polychaetes, soft- and hard-substrate filter feeders came from the compilation of data obtained from SMHI, ICES, DCE (Danish National Center for Environment and Energy; www.dce.au.dk) and HERTTA (Finnish Environmental Institute; www.p2.ymparisto.fi/scripts/oiva.asp) databases. The data used covered a period from 2003 to 2007. Literature information on coverage and/or biomass was obtained to estimate initial conditions for spatial distribution of macroalgae [73–75] and seagrass [76].

Table C in the S1 File provides a summary of the sources used to inform the model with the required biological fields, fluxes and parameters for the 2005 initial conditions year for all vertebrate and/or commercially important species (e.g. Nephrops). A distinct spatial distribution was specified for adults and juveniles, and by season (half year), if the data was available. Details of these calculations and considerations for vertebrates and invertebrates can be found in S1 File section B.2. Because the input information is derived from observed or previously modeled mean annual concentration and biomass levels taken from the 2005 reference year, we can check whether the model is able to reproduce realistic biomass levels for those groups. We do not provide a similar comparison for invertebrates and the lower trophic levels for which we did not have accurate or equivalent data to start with. However, their values are set according to the best available knowledge. Initial values of detrital matter were purposefully set very low for the model initial conditions in order to avoid too strong grazing pressure and in general larger internal model error due to a choice of initial conditions.

#### Unique Atlantis parameters

There is a group of parameters that are unique to Atlantis, e.g. the availability of prey parameter, which is conceptually difficult to derive from stomach content data (see detailed discussion in S1 File section B.2). Such parameters were therefore initially constrained using qualitative information and often adjusted substantially during the calibration process. When no information about realistic ranges of parameters were found, the robustness of the final calibrated value was evaluated using the emergent model results (i.e. diet composition) or through sensitivity analysis by changing ranges of the parameters. Tables D-F in the S1 File provide a summary of the final calibrated values assigned to the key biological parameters describing the structure and function of the ecosystem in the Baltic Atlantis for vertebrate and invertebrate groups, respectively.

Other examples are the parameters for diffusive fluxes, which are calculated internally on top of advective fluxes. While we apply a constant vertical mixing rate across the majority of the model domain, these mixing rates needed to be adjusted in order to better simulate nutrient replenishment in the offshore surface waters. Other vertical transport processes occur across the water-sediment interface via bioirrigation and bioturbation of sediments, sediment resuspension and erosion; as well as in the water column via particle settling. Also horizontal and vertical light fields are calculated internally by the model. Daily and seasonal cycles of photosynthetically active radiation are obtained from calculations of the theoretical net incoming shortwave radiation at given longitude and latitude. The vertical extent of the light profile in the water column is an exponential decay function scaled by physical and biological light attenuation parameters (Table A in S1 File).

### The Baltic Atlantis model application: Structure and processes

#### Biological functional group structure

**Fig 3:**
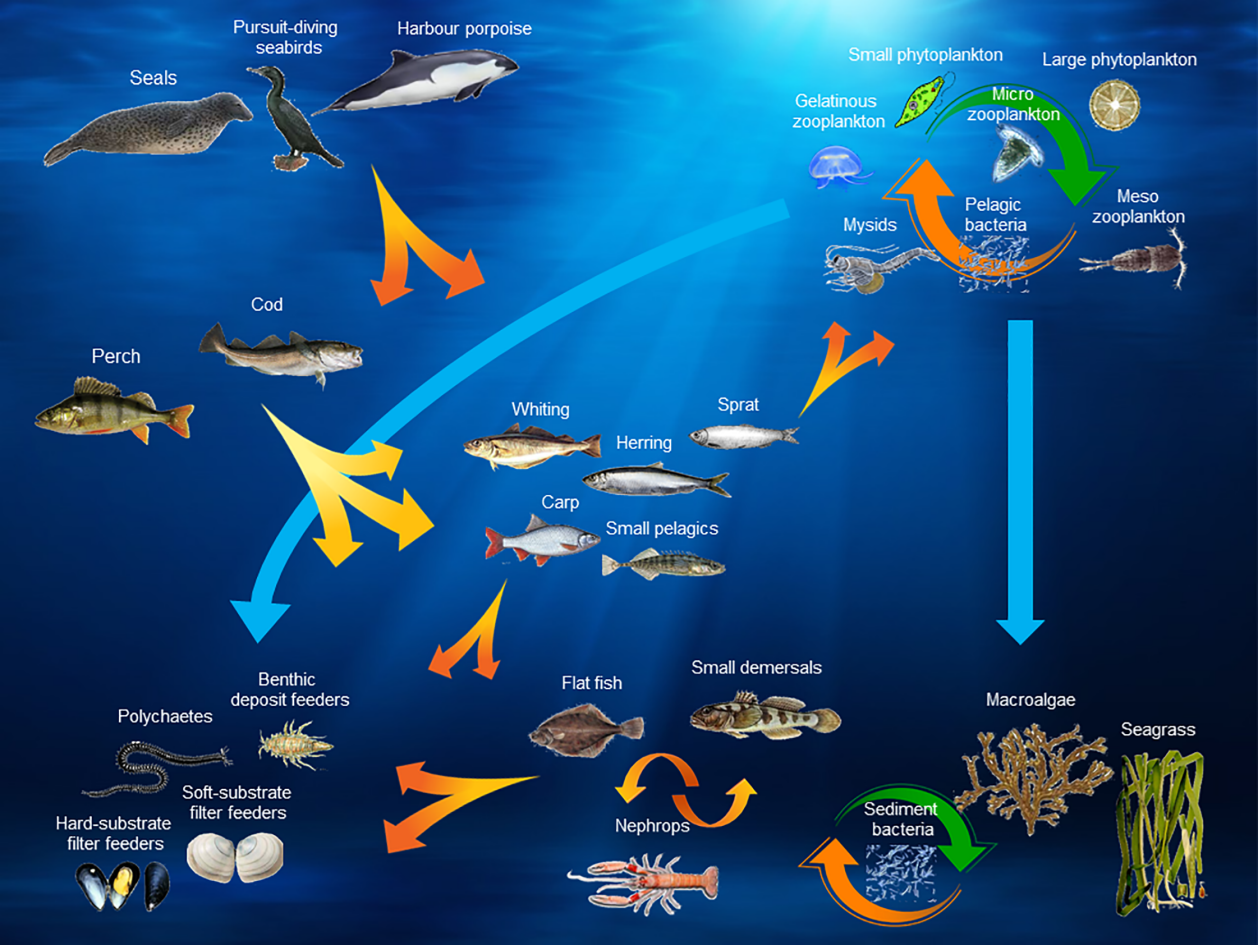
Baltic Atlantis model–biological structure. Detail list of species included in each group is found in Table G in S1 File. The figure illustrates the main interactions focused upon in the current context, i.e. a comprehensive diagram of the full biological interactions between functional groups in the Baltic Atlantis model will be way too complex to overview in one diagram.

The Baltic Atlantis model has 30 biological functional groups. Table 1 provides an overview of the biological structure of the model by indicating which and how many functional groups there are for each ecosystem level, and Fig 3 illustrates their habitats and the general interactions between the biological groups in the model. The key players of this system are cod, sprat and herring. All three of them are predated by the harbour porpoise, seals and seabirds. Sprat and herring are also the main prey species for adult cod, while the benthic invertebrate community is the main food source for juvenile cod. The majority of biological functional groups encompass multiple species, except when there is sufficient information to parameterize dynamics of individual species which are either of great economic value—e.g. the sprat, herring, and cod fish stocks—or have important and distinct ecosystem roles, e.g. harbour porpoise. For functional groups consisting of several species, the biological traits of the dominant species, or the species with a key ecosystem role, are chosen to be representative of the whole group. All biological functional groups, together with names of species aggregated within them and general model behavior parameters are listed in Tables G and H in the S1 File respectively. The reasons and criteria used to aggregate individual species into biological functional groups are described in section B.3 in S1 File.

**Table 1:** Summary of biological structure of the Baltic ATLANTIS model.

Ecosystem level	# functional groups	Population structure	Biological functional groups and model codes
Dead organic matter	3	Single biomass pool	DL (labile detritus), DR (refractory detritus), DC (carrion)
Bacteria	2	Single biomass pool	BP (pelagic bacteria), BB (sediment bacteria)
Pelagic primary producers	3	Single biomass pool	PS (small phytoplankton), PL (large phytoplankton), ^a^PC (cyanobacteria)
Benthic primary producers	3	Single biomass pool	MA (macroalgae), SG (seagrass), ^a^PB (phytobenthos)
Pelagic invertebrates	4	Single biomass pool	ZS (small zooplankton), ZM (mesozooplankton), MS (mysids), ZG (gelatinous zooplankton)
Benthic invertebrates	5	Single biomass pool	SF (soft-substrate filter feeders), HF (hard-substrate filter feeders), BF (benthic deposit feeders), NE (Nephrops), PO (polychaetes)
Fish	9	Age-structured	FSR (sprat), FHR (herring), FCD (cod), FWH (whiting), FFL (flat fish), FPR (perch), FCP (cyprinids), FSP (small pelagics), FSD (small demersals)
Seabirds	2	Age-structured	SBD (pursuit-diving seabirds), ^a^SBS (surface-feeding seabirds)
Marine mammals	2	Age-structured	MHP (harbour porpoise), SEA (seals)

All vertebrate groups are age-structured in the model and include 5 or 10 age classes, depending on their life expectancy. All invertebrate groups are represented by a single biomass pool.

aGroups currently set as inactive in the model, i.e. they will be excluded from the model. This allows to include them again more easily in future model improvements.

Mammals, seabirds and fish are age structured with a set amount of age groups (cohorts) (Table H in S1 File, section B.2 in S1 File–Demographic profiles, mortality rates & reproduction functions). Age structured functional groups mature at a set age, which is modelled as a hard transition point from a juvenile to adult stage—this distinguishes diets, habitat use, migration patterns etc; This does not assume the age of sexual maturation though, which is given by a spawning ogive which determines the proportional contribution to reproduction. This means that sexual maturity and contributions to the pool of new recruits can begin before or after the switch from juvenile to adult behavior. The number of recruits is modelled following the standard Beverton-Holt relationship [77], where the recruitment is a function of the total species biomass and the amount of spawning produced, scaled by temperature, salinity and oxygen conditions (Table D in S1 File). Details on how this is done can be found in section B.2 in S1 File–Demographic profiles, mortality rates & reproduction functions. Currently, the number of recruits for mammals and seabirds is also modelled following this approach to create a density dependence relationship with the environment they live in. The effects of temperature and salinity on the recruitment of harbour porpoises and seals are however not seen as an influential factor and are turned off for those two species accordingly. Besides recruitment processes, oxygen concentration conditions also affect all groups while living (for equations on the processes and detailed explanation see section B.2 in S1 File–Demographic profiles, mortality rates & reproduction functions). Benthic invertebrates experience an increased mortality when oxygen drops below their minimum tolerance levels (Table F in S1 File) as they cannot escape the oxygen minima that easily, while fish and other vertebrates are assumed to swim away. Although, if no suitable habitat is found (i.e. if the entire area was to become anoxic), they would be completely lost from the model. A potential uncertainty lies in the precise formulation of the fish recruitment dynamics in the Baltic Atlantis. As there are large regional differences in recruitment parameters calculated for different important fish stocks in the Baltic, the selected values are not always equally representative of both the eastern and western stock components. The final values for the Beverton-Holt stock recruitment relationship were chosen based on the ability to reach a long-term equilibrium biomass.

Age structured functional groups, i.e. all vertebrates (Table 1), also have a maximum growth rate and clearance rate for each of the age groups, while biomass pool functional groups, i.e. all invertebrate groups (Table 1), only have one specific value (Tables E and F in S1 File). Growth for primary producers is defined as a function of the maximum specific growth rate and by additional potentially limiting factors such as nutrient, light and space limitations. For consumers, growth is determined by the intake (grazing) and assimilation efficiency. Maintenance or respiration costs for age structured groups are implicitly represented in the assimilation efficiency, i.e. the rate is set lower to represent losses to respiration, as well as incomplete assimilation of food. Grazing is modelled using a modified Holling type II relation [54], where the grazing is a function of the amount of biomass of a specific prey available and the time-invariant maximum growth (grazing) rate and clearance rate (the search volume of a predator). Limitations of the available prey biomass are the time-varying spatial overlap and predator condition, the available habitat for refuge and the gape size of the predator. This together will define the amount of prey consumed, which means that the diet is not a priori determined (as in other models such as EwE), rather an emergent property of the Atlantis model.

While the initial values of vertebrate growth and consumption rates per age class were fairly well constrained using field data on morphological characteristics and physiological rates, and size-based scaling laws of metabolic theory, up to an order of magnitude adjustments had to be made for some groups. The two criteria used to adjust these rates were: (i) obtaining a close to initial structural and reserve weight per age group, and (ii) obtaining a stable and ecologically-sound diet composition.

#### Atlantis spatial domain structure

The Baltic Atlantis model resolves horizontal processes based on 29 polygon-shaped boxes, including 26 dynamic and 3 boundary boxes, and a total of 100 faces (borders) between them (Fig 2), delineated using a Geographical Information System, following the standard procedure for all Atlantis implementations [54]. The polygon structure is designed as a compromise between the need to account for spatial heterogeneity in bathymetric and hydrodynamic patterns, physical-chemical and biological habitats, biological composition and existing fisheries and spatial management units. In the vertical dimension there are eight water layers: 0-5m, 5-10m, 10-30m, 30-40m, 40-50m, 50-100m, 100-200m, >200m; and one sediment layer of 0.5m depth. The depth of the vertical water layers is variable with a higher resolution for layers shallower than 50m depth. This higher resolution helps the model to resolve key vertical processes around the halocline in offshore areas of the Baltic. The last vertical layer is a sediment cell. Which types of abiotic habitat this sediment cell has, is specified based on the high resolution topographic map information from HELCOM (Table I in S1 File).

There are a few limitations when modelling the different functional groups in this spatial domain structure. Firstly, the spatial distribution of primary and secondary producers in the model is complementary where large and small phytoplankton rarely coexist in the same polygon, similarly for micro- and mesozooplankton. While this implementation allows for the effective representation of the total biomass of both groups, the biomass by individual polygon might be biased due to the model set up. Complementary groups are therefore aggregated over polygons when plotted spatially. Secondly, the spatial distribution of the vertebrates is forced seasonally for the species, based on survey data and literature and does not allow for free movement between polygons (see section B.2 in S1 File–Quarterly abundance distribution of vertebrates). The distribution pattern is also limited by the single uniform fishing mortality imposed on all individuals of a given biological functional group. This current implementation potentially leads to approximate fishing mortalities per subareas.

### Nutrient load reduction scenarios and fishing pressure change sensitivity

In the current study, four nutrient load reduction scenarios have been evaluated and five model sensitivity runs with changed fishing pressure for key fish species were conducted. This was done to test the model sensitivity and its capability to respond similarly to realistic situations. There was one “status quo” scenario to compare against which assumed no changes in nutrient loads or fishing pressure. The sensitivity tests of fishing pressure reduction was made for fishing mortality on cod as a major benthivorous and piscivorous predator fish and on sprat as a major planktivorous prey fish in the Baltic, which are both important target species in the Baltic international commercial fishery. All scenarios were run for a period of 60 years including a “spin-up period” of 35 years after which equilibrium is reached and at which point the nutrient loads are changed depending on the scenario. Like any simulation from the Atlantis model the results of these scenario runs do not represent an actual 25-year time series forecast of the response to nutrient change, rather, they represent a possible future time slice after the biological system had adjusted to the new forcing conditions and the bio-economic model had optimized its performance. Here we use five-year averages of the terminal projection period. The model adjustment time to new nutrient load and/or fishing pressure conditions is not uniform for all ecosystem components, therefore a complete equilibrium is not guaranteed (however, equilibrium was actually reached in all cases but one). Moreover, the change in forcing in the model occurs instantaneously instead of a more likely gradual shift, as this is how the model is set up for the moment. This adjustment or model spin-up time is longer than the 10-year time scale assumed in previous similar numerical experiments (e.g. [21]) performed on much simpler ecosystems.

There were four nutrient load scenarios and five fishing mortality scenarios set up and simulated within the Baltic Atlantis model and management evaluation framework. Table 2 provides a summary of the different scenarios and the corresponding relative (%) reductions of total nitrogen river load assigned to each Baltic Atlantis polygon per nutrient load scenario and of the corresponding fishing mortalities used for each of the last five scenarios. The changes in fishing mortality were intentionally kept simple for testing purposes rather than trying to replicate complicated management options. The original fishing mortality of adult cod, applied in the Baltic Atlantis model, is 0.32 and the original, SMS-derived fishing mortality for adult sprat is 0.07. Minimum observed fishing mortality was not included in the scenarios as it was considered sufficient to look at the halved mortality rate (i.e. scenario #6 and #7). In the current settings of the Atlantis model, the fishing mortality is a constant mortality factor for the whole Baltic Sea, split into juvenile and adult. There is also no differentiation between different stocks in the current model, so there is only one fishing mortality for the juvenile and one for the adult group of the species for the whole area. In the evaluation of eutrophication scenarios with Atlantis, we keep fishing mortality constant to focus on nutrient load induced pressures and relative changes. In the subsequent FISHRENT evaluations, we accordingly evaluate relative fisheries consequences of the changed biomasses of key exploited fish stocks from the eutrophication scenarios only. We therefore have kept stock biomass and recruitment constant in the fisheries projections in FISHRENT to isolate the relative effects of the eutrophication scenarios.

**Table 2:** Relative (%) reduction of total nitrogen river load applied to certain Baltic Atlantis polygon according to the four nutrient load scenarios (#2–#5) and the changes in fishing mortality for the five fishing mortality scenarios (#6–#10). Scenario 1 is the baseline to compare the results against.

	**Scenario #1**	**Scenario #2**	**Scenario #3**	**Scenario #4**	**Scenario #5**
	'Status quo'	Nutrient load reduction	Nutrient load reduction	Nutrient load reduction matching BSAP2*	Nutrient load reduction
**Area**		Denmark	Denmark, Sweden & Germany	Selected coastal zones	pan-Baltic
**Polygon # and amount of decrease for each polygon**		12 %, 33%, 24%	33% for	9%, 20%, 15%, 32%	33%
		for polygon 1, 2, 4	polygon 1, 2, 3, 4	for polygon 1, 2, 3, 4,	
				21.4%, 23% for #5 & #9,	
				19% for #12 & #13,	
				35%, 33% for #14 & #17	
	**Scenario #6**	**Scenario #7**	**Scenario #8**	**Scenario #9**	**Scenario #10**
	50% reduction of fishing pressure on cod	50% reduction of fishing pressure on sprat	50% increase of fishing pressure on cod	50% increase of fishing pressure on sprat	The maximum 2005–2012 observed fishing pressure on cod and sprat
**Area**	pan-Baltic	pan-Baltic	pan-Baltic	pan-Baltic	pan-Baltic
Fishing mortality for adult fish**	0.16	0.035	0.64	0.14	1.2 for cod,
					0.6 for sprat

* This is a regional approach, agreed by the Baltic Sea countries, to share the burden of nutrient reductions and achieve the goal of a Baltic Sea, unaffected by eutrophication [60, http://www.helcom.fi/baltic-sea-action-plan/nutrient-reduction-scheme/targets/].

** New juvenile fishing mortality was assumed half of the new adult mortality.

Note: the corresponding polygons and their numbers can be found in Fig 2.

Analysis of these scenarios, and of the quality of the initial calibration, was based on a small set of criteria. First, the overall balance of all biological functional groups was evaluated after running the model for a 60 year projection period, to see whether equilibrium was reached. There is no guarantee that the model reaches irreversible global equilibria, it can be quasi-equilibria. However, the equilibria did generally not change when using 120 years projections compared to 60 years (Figure B in S1 File). Next, the levels of the final biomasses were compared with the initial input biomasses, especially for those groups where external abundance estimates were available. Furthermore, we compared the diets versus data to check whether the realized model diets were realistic. Due to the emergent property of the diet in the Atlantis model, we can still use the calibration data to check the output against. Besides the dynamics of the total relative prey composition over the full simulation period, the diets of juvenile and adult predators were also checked separately, averaged over a timespan of the last five years of the projection period. Next, we also explored the population demography according to age frequencies, i.e. distribution of the number of individuals over the age groups. Additionally, the spatial distribution of the biomasses as well as the oxygen level in certain areas and vertical layers were checked against what is known from literature or field surveys. We finally investigate the results of the scenarios by comparing their biomass levels with those in the baseline run, in order to evaluate the sensitivity and realism of the model response to the change in drivers and pressures. These biomass oriented evaluations are augmented for the age structured groups—mammals, seabirds and fish functional groups—where a more detailed demographic evaluation of the model projections is possible.

### Economic analysis of the Atlantis output in Kattegat-Western Baltic

The FISHRENT model [60] was used to analyze the final year output of the Atlantis model. FISHRENT is a multi-stock multi-fleet bio-economic model which was developed from best practice knowledge on fisheries gained from similar models used in Europe over the past decade (e.g. [78–79]). FISHRENT enables consideration of a diverse array of policy aims, including Maximum Sustainable Yield (MSY) and Maximum Economic Yield (MEY) goals. The main outputs from FISHRENT describe the likely trajectories and status of the modelled fisheries and fleets over a predetermined time-period given the status of the main fish stocks exploited per area and the policy aims considered. The model contains five modules: biological, economic, market, behavior, and policy (Figure C in S1 File). A key characteristic of FISHRENT is that the necessary fleet and economic data have been structured in the same way as the data collected on a national basis as part of the EU Data Collection Framework (EU DCF). This enables consistent analysis to be conducted across nations ensuring that best available national data is used. More detailed information about the FISHRENT model and its implementation in the Kattegat and the Western Baltic Sea can be found in section C, Tables J-R in S1 File. The economic consequences of the nutrient load scenarios two to four are evaluated by the FISHRENT model.

## Results

### Initialization and forcing of the Baltic Atlantis

**Fig 4:**
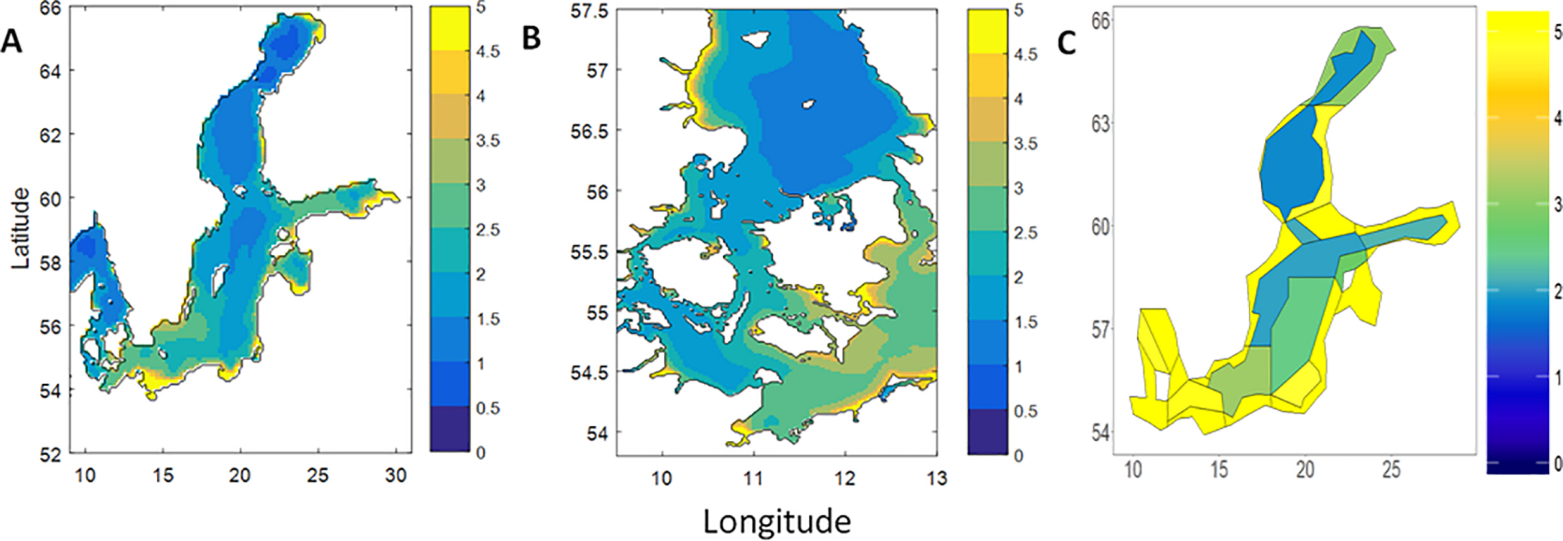
Spatial distribution of annual average surface Chl-a [mg m^-3^]. As simulated by HBM-ERGOM for the annual average over the period 2005–2014 (A-B) and Baltic Atlantis for the annual average for the last five projection years (C). Areas marked in yellow represent concentrations of 5 mg m^-3^ or higher.

A comparison of the Chl-a levels and spatial distribution in the output from the Atlantis model (annual average for the last five projection years) and the modelled output by the HBM-ERGOM model (annual average for 2005–2014) (Fig 4) shows that the relative distribution and general levels of Chl-a in Atlantis matches the patterns and levels in HBM-ERGOM. However, this is not the case for the Kattegat and the Western Baltic Sea, where the Atlantis model shows average higher Chl-a values on a more coarse geographical scale while HBM-ERGOM only shows high levels in fine scale coastal areas. Seasonal patterns in Chl-a are shown in Figure D in S1 File and are discussed further below in relation to HBM-ERGOM output.

### Baltic Atlantis projection results

#### Biomasses of biological functional groups

**Fig 5:**
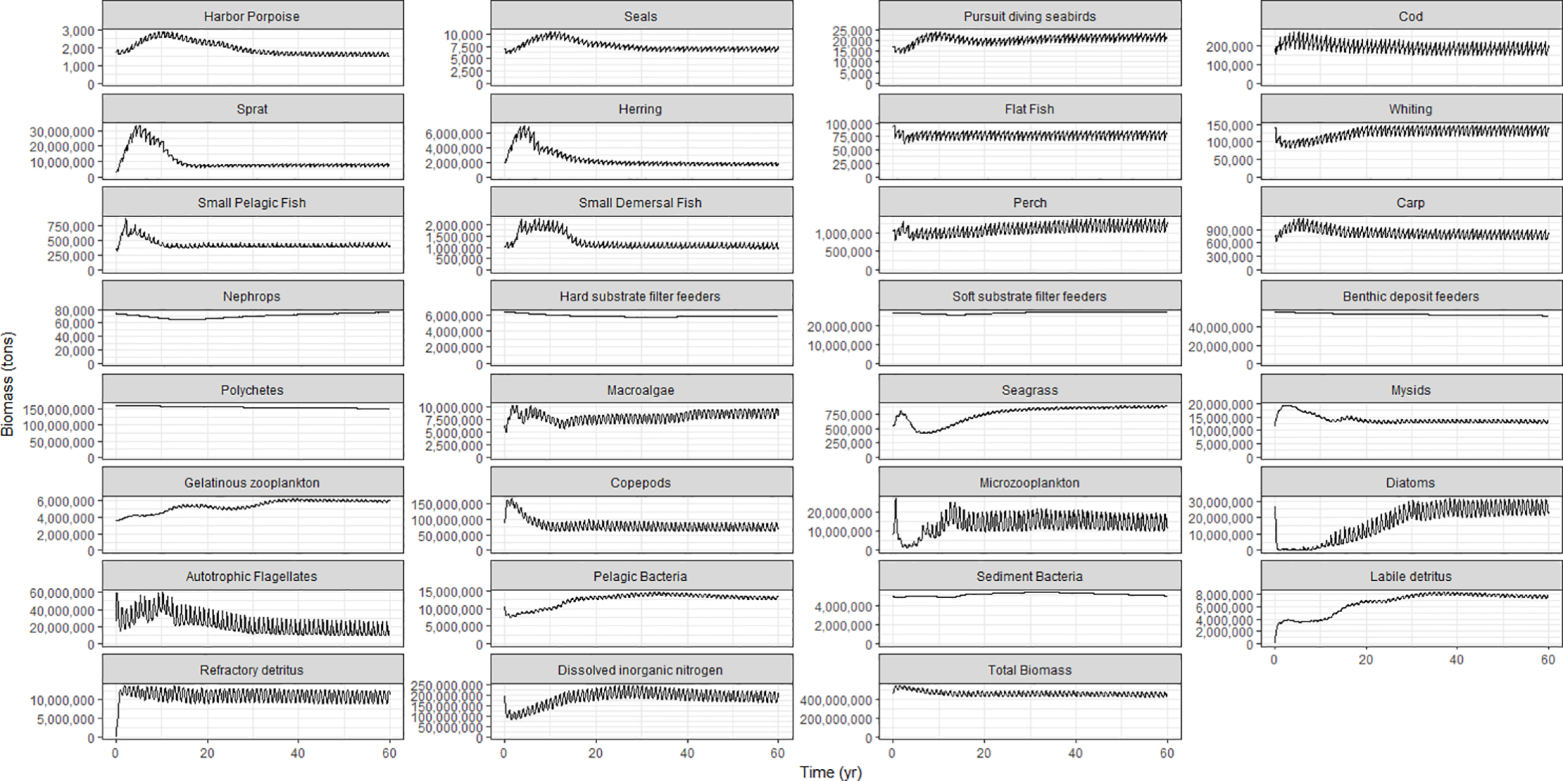
Time series evolution of Baltic Atlantis functional groups. Total biomass in metric tons of biological functional groups and total DIN (dissolved inorganic nitrogen) obtained from a 60-year reference run initialized with 2005 data.

The time series of total biomass per group (across the entire model domain) in the 60-year long calibration run are shown for all species in the overview plot Fig 5. The biomasses of all functional groups reach a balanced equilibrium with a spin-up period of 10 years for most of the groups. For some groups like the harbour porpoise, phytoplankton and gelatinous zooplankton, it takes a longer spin-up time of about 35 years. The simulated fluctuations for most of the functional groups arise from the seasonal variability. The largest variations in these fluctuations are found for the lower trophic level groups, 10 million tons biomass amplitude for the diatoms and small zooplankton, 20 million tons biomass difference for the small phytoplankton and 30 million tons difference for the mesozooplankton. Benthic organisms do not show such variation. Comparing the modelled biomasses with the input showed that most of the groups fall in the border of 0.5 times lower or higher than initial values (Figure E, Table S in S1 File). Some groups that ended up with a biomass higher than 0.5 times the initial level are sprat, gelatinous zooplankton, seagrass and labile and refractory detritus where sprat biomass is 2.72 times the initial biomass.

#### Diets of biological functional groups

**Fig 6:**
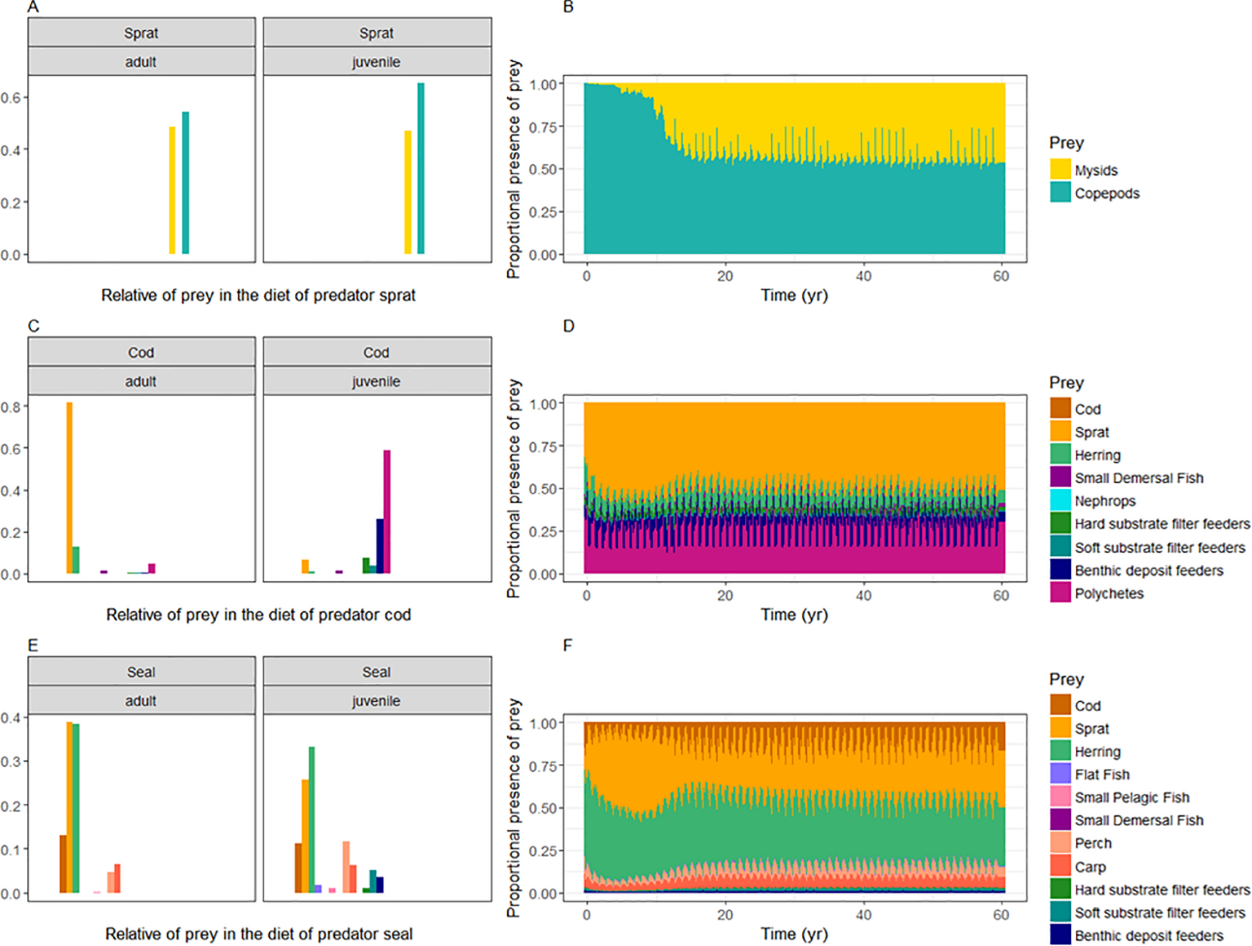
Emergent diet composition of sprat, cod, and seals from the whole of the Baltic Sea. Results are average from last 5 years of model 60-year calibration run for both juvenile and adult groups, with the prey being arranged on the x-axis according to the trophic level (A, C, E) and the dynamics of the diet composition simulated over the 60 years–combined for adults and juveniles (B, D, F).

The diets for sprat, cod and seals are given in Fig 6, while the diets of the other functional groups can be found in Figure F in S1 File, panels 1–21. The results show that the emergent diet of sprat in the model consists of mainly lower trophic level groups, namely 55–65% of mesozooplankton and 35–45% of mysids (Fig 6A and 6B). Juvenile cod predates mostly on benthic invertebrates, 55% on polychaets, 25% on benthic deposit feeders, 5% on soft substrate filter feeders and 10% on hard substrate filter feeders. The diet of adult cod consists for 80% of sprat and 15% of herring (Fig 6C and 6D). Adult seals eat primarily herring and sprat, and secondary cod. The dietary pattern of the juvenile seals consists for a large part of herring and sprat as well, besides 10% of perch and 6% of carp (Fig 6D and 6E).

#### Population structure and demography

**Fig 7:**
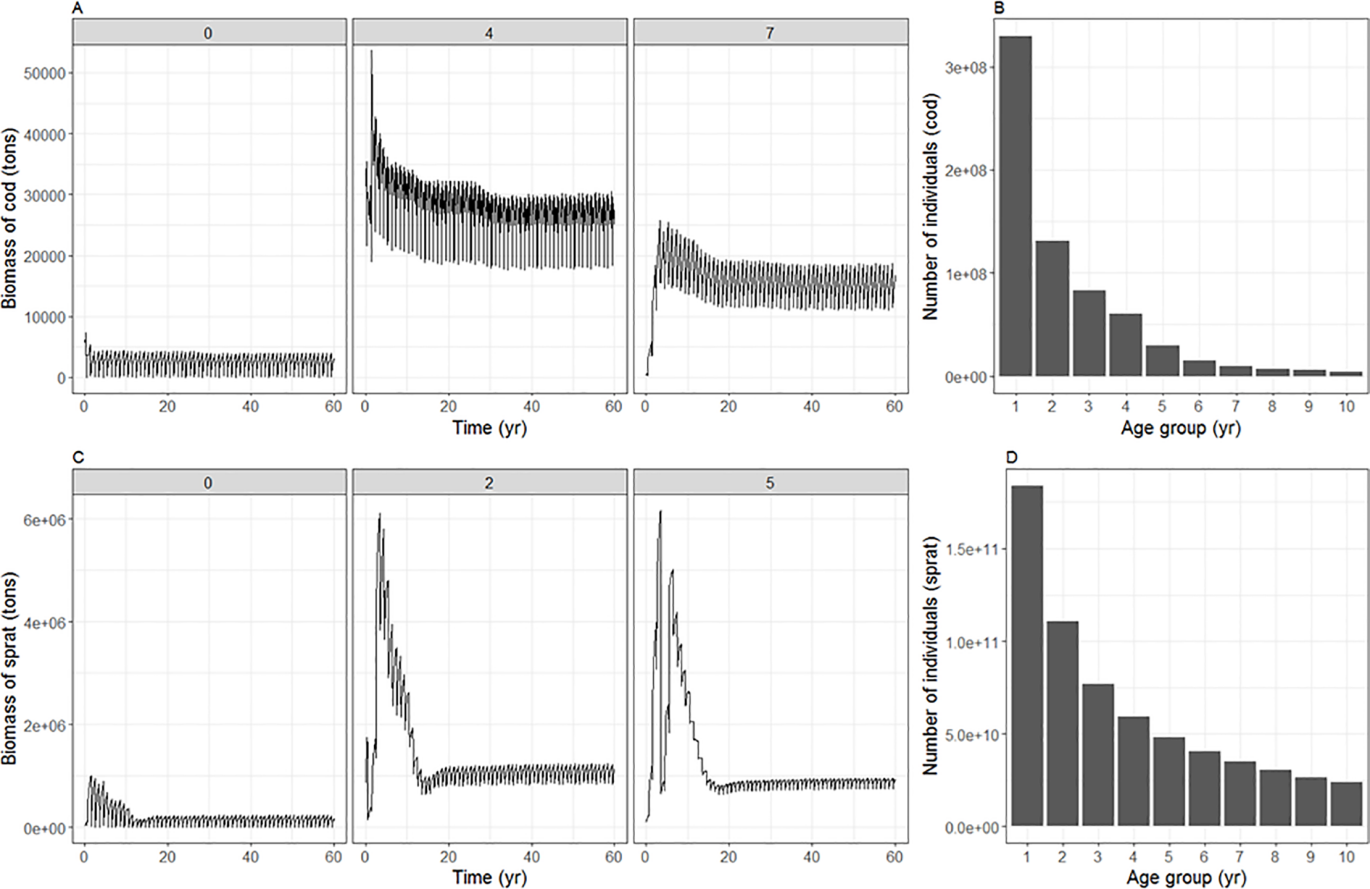
Biomass, condition and demography of Baltic cod and sprat, taken as an annual average from last 5 years of calibration run for the whole Baltic Sea. The individual and population metrics per age class include: (A, C) biomass for 3 age groups over the 60 year simulation period (B, D) number of individuals.

The equilibria reached in the overall biomass, is also found in each of the age groups (Fig 7A and 7C and Figure G in S1 File, panels 1–12). Terminal biomasses are similar to survey biomasses for all age structured groups. All groups are within the same level of total abundance compared to the observations from survey data which were used to initialize and calibrate the model (Figure H in S1 File). For all the functional groups there is an exponential decrease in numbers per cohort from the youngest to the oldest age groups, as expected (Fig 7B and 7D).

#### Spatial distribution of biological functional groups

**Fig 8:**
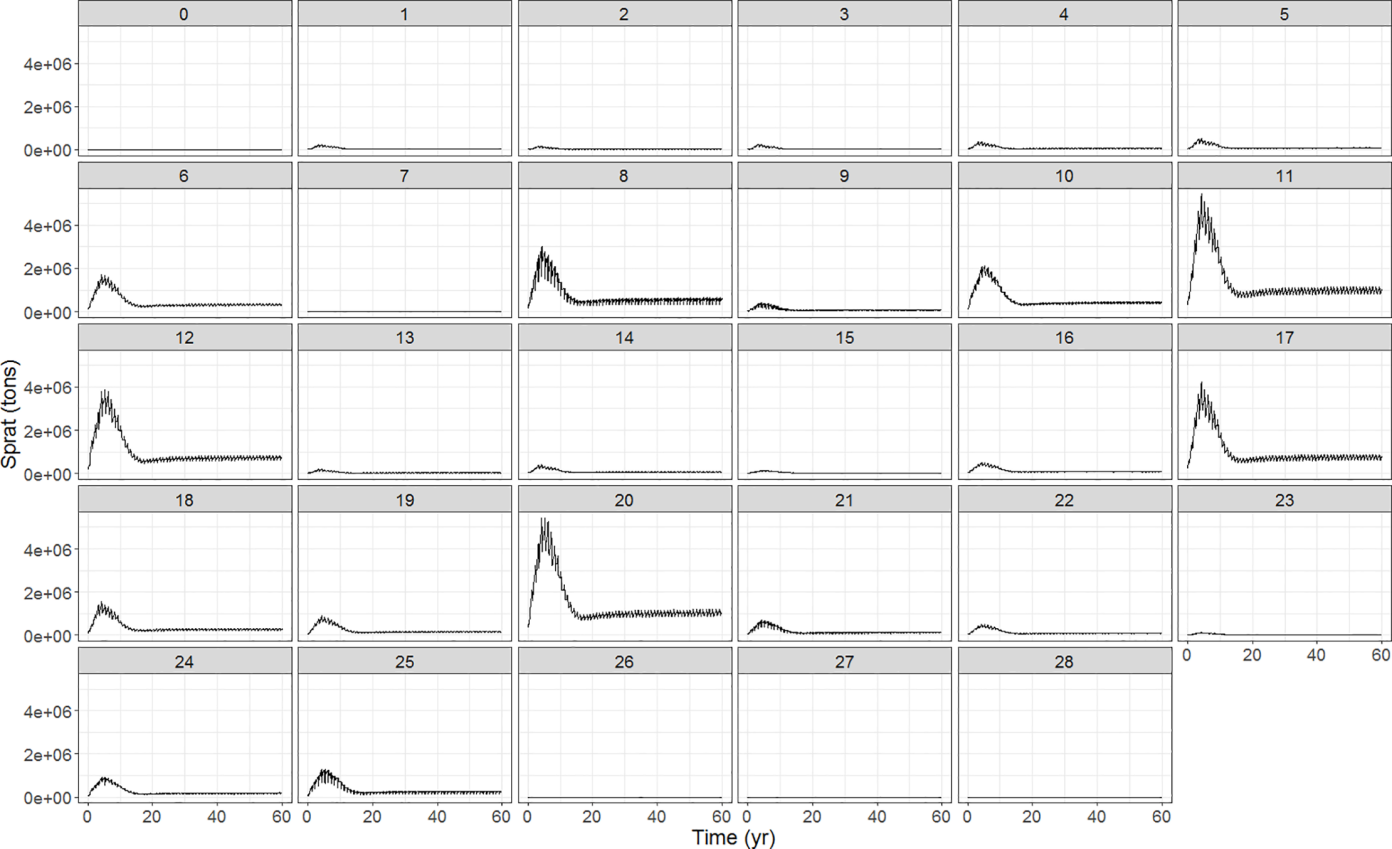
Spatial distribution of Baltic sprat biomass for the 60 year simulation period.

The sprat biomass reaches equilibrium at the end of the model projection for each of the polygons (Fig 8). The spatial distribution of biomass of all the other functional groups can be found in Figure I in S1 File, panels 1–33. It can be seen that most of the groups reach a stable equilibrium (60 and 120 years projection, Fig 5 & Figure B in S1 File) and this equilibrium is spatially consistent, i.e. none of the species with known distribution patterns go extinct in any of the polygons. However, there are some exceptions in which the model can be improved (e.g. mysids, macroalgae, seagrass). Microzooplankton and mesozooplankton as well as large and small phytoplankton are grouped together to zooplankton and phytoplankton respectively, for which combined groups the spatial distribution is given. Phytoplankton spatial distribution is also shown through the distribution of Chl-a (Fig 4C). A pattern appears in which the coastal polygons and the Western Baltic Sea show higher fluctuations than the off-shore ones (Figure I in S1 File, panel 16). Phytoplankton peaks are highest for polygon 24 (Figure I in S1 File, panel 16b), but they still reach a base equilibrium (Figure I in S1 File, panel 16).

**Fig 9:**
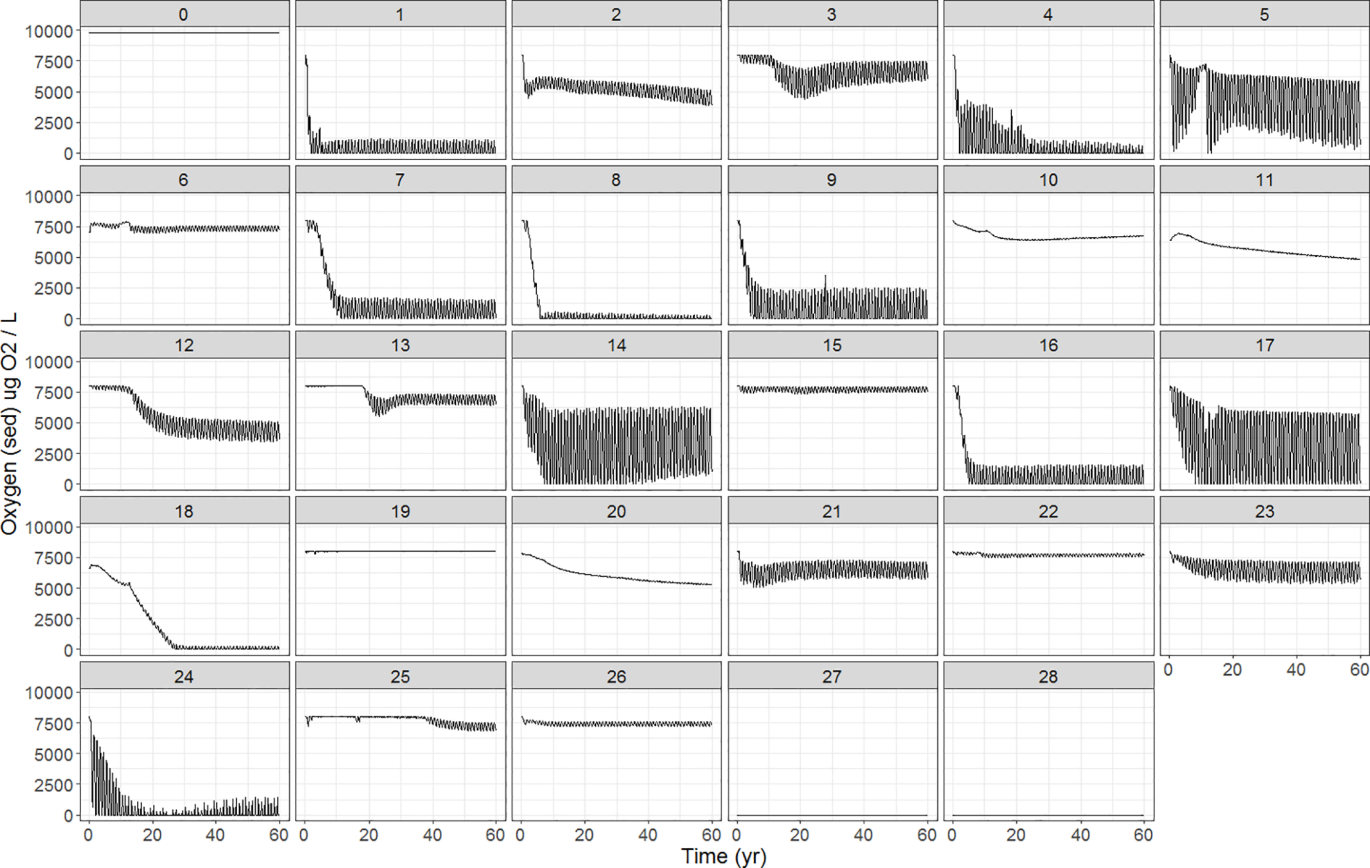
Spatial distribution of bottom oxygen concentration for the Baltic Sea for the 60 year simulation period.

Most polygons show seasonal fluctuations in oxygen levels, with periodic zero values (Fig 9). Oxygen levels for the vertical water column can be found in Figure J in S1 File, panels 1–7.

### Nutrient load reduction scenario results

**Fig 10:**
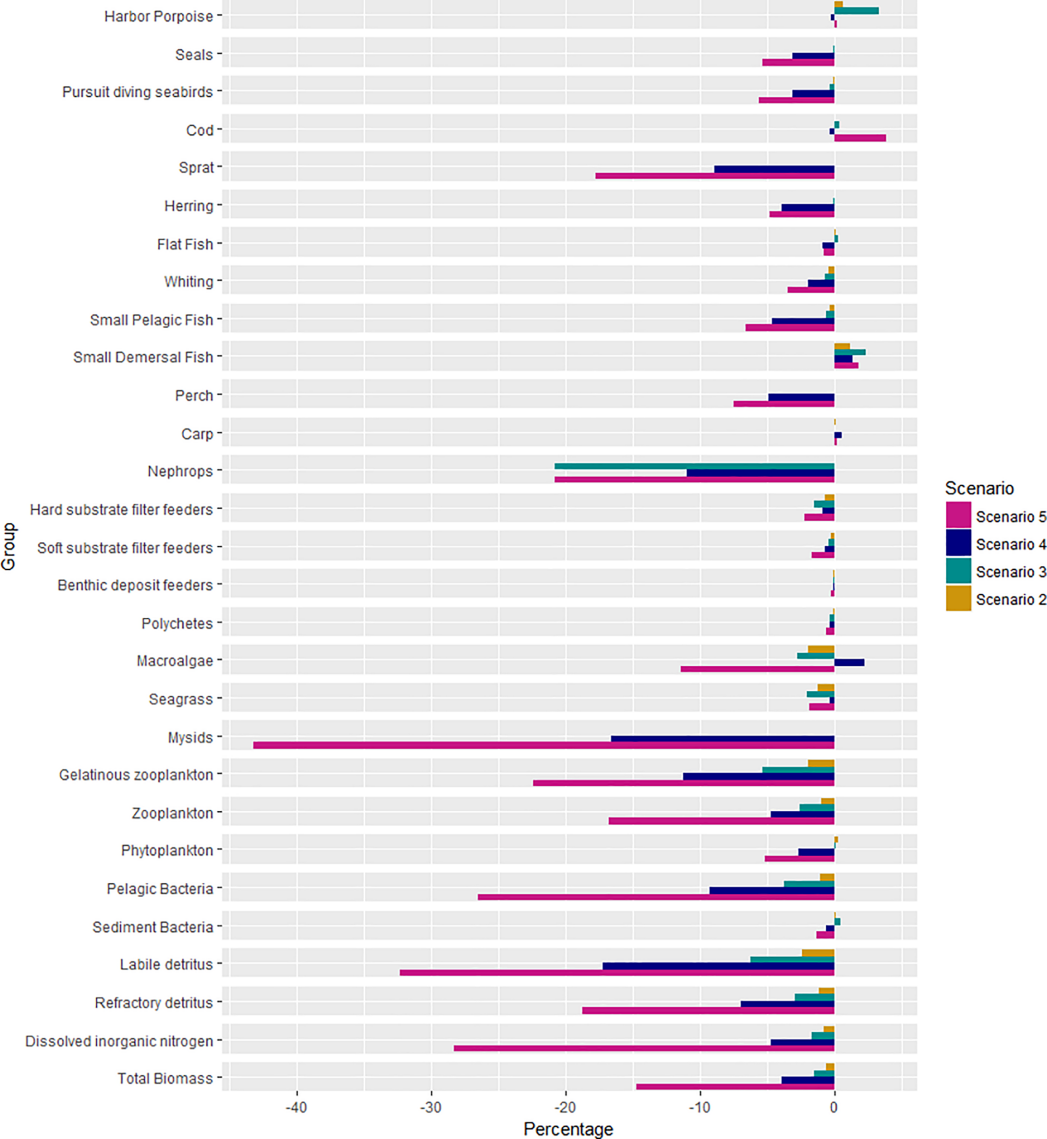
Results of the four river load scenarios. Percentage of change of the biomass for the different biological functional groups compared to the status-quo scenario #1.

From the four nutrient scenarios that were analyzed with the calibrated Baltic Atlantis model, three were also evaluated with the FISHRENT model (i.e. scenario #2–#4). The polygon-based results from those scenarios were converted into stocks and regions consistent with the FISHRENT model structure and associated assumptions, thus providing adequate relative fish biomass scaling indices, applied to scale the constant biomasses in FISHRENT. The indices per scenario for the commercially important species are summarized per area in Table 3. The results for all of the ecosystem functional groups are shown in the form of relative changes in the biomass in Fig 10.

**Table 3:** Relative changes (%) of equilibrium fish and Nephrops total stock biomasses (TSB), averaged over the final simulation year, given the three nutrient load reduction scenarios (#2–#4) evaluated economically relative to the status-quo scenario (#1), of the species groups included in the Baltic Atlantis model.

Species	Scenario #2	Scenario #3	Scenario #4
**COD_KA**	0.04 (0.04)	0.34 (0.35)	-0.34 (0.36)
**COD_WB**	0.04 (0.04)	0.34 (0.34)	-0.34 (0.32)
**SPR_KAWB^1^**	0.01 (0.005)	-0.04 (-0.04)	-8.96 (-8.96)
**HER_KAWB^1^**	-0.01 (-0.01)	-0.13 (-0.15)	-3.9 (-3.88)
**WHI_KAWB^1^**	-0.42 (-0.38)	-0.75 (-0.73)	-1.92 (-1.90)
**FLAT_KAWB^1^**	0.11 (0.11)	0.3 (0.33)	0.88 (0.84)
**NEP_KAWB^2^**	-0.01 (-0.01)	-20.97 (-20.97)	-11.02 (-11.02)

Note

^*1*^For sprat, herring, whiting and flat fish, the box estimates were averaged over to provide one estimate for the entire Kattegat and W Baltic region.

^2^Only values for E. Kattegat.

Note: The values between brackets are calculated with a different way of averaging and summing biomasses according to the different polygons, taking into consideration seasonal migrations. However, it can be seen that there are no differences between the two methods.

KA = Kattegat, WB = Western Baltic Sea, SPR = sprat, HER = herring, WHI = whiting, FLAT = flatfish, NEP = Nephrops.

The biomass changes in scenario #2 are negligible, whereas for the other three scenarios we see an overall decrease in total biomass compared to the baseline. For most of the groups, the decrease in biomass was larger, the stronger the scenario was. The two last scenarios are the ones with the most significant changes in total biomasses, which are observed throughout the whole ecosystem as a bottom up effect, where even the top predators like mammals and seals are either positively or negatively affected. The higher up the trophic level, the lower the difference with the baseline.

Labile detritus, mysids and Nephrops are the most impacted, and their biomass levels decreased according to the magnitude of nutrient reduction in the Kattegat and Western Baltic Sea. Detritus is an important food source for Nephrops (see Figure F in S1 File, panel 13). The decrease of Nephrops biomass was less in scenario #4 given that the nutrient reduction was lower in scenario #4 for Kattegat, only a 15% reduction, because this scenario followed the BSAP plan, whereas the scenario #3 had a 33% reduction by Denmark, Sweden and Germany together.

**Fig 11:**
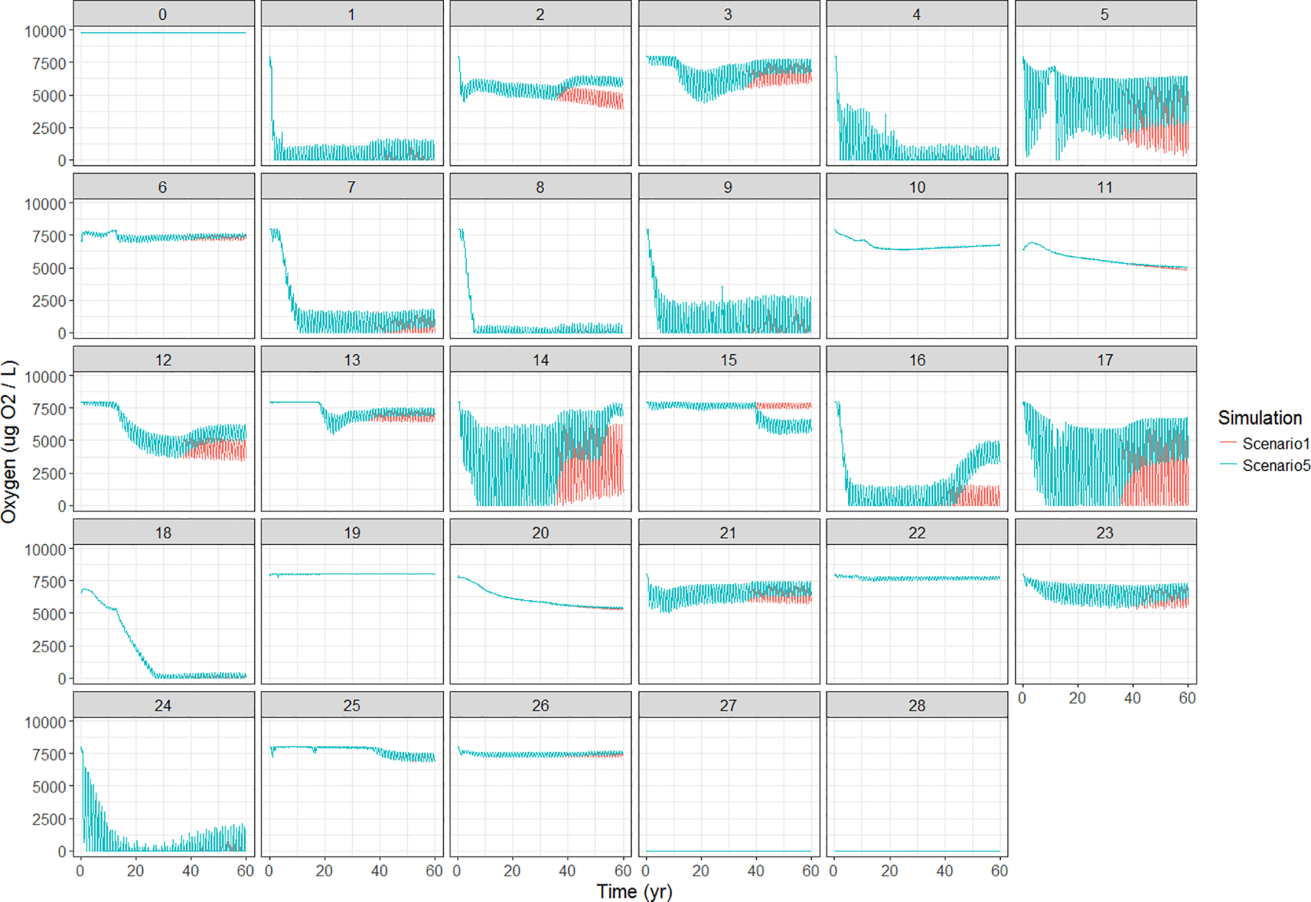
Oxygen concentration of bottom layer: Baseline (scenario #1) vs. scenario #5.

Most of the functional groups biomasses decreased with lower nutrient input (Fig 10). Some groups do not have a consistent pattern over the different scenarios and might increase as well. Cod increased up to 4% in the fifth scenario, where both adult and juvenile cod biomass increased (Figure K in S1 File). The cod dietary pattern changed in scenario #5 with a decrease in sprat for adult cod, while juvenile cod had an increase in prey biomass of the benthic filter and deposit feeders, and of the polychaetes (Figure L in S1 File). Benthic invertebrate biomass decreased for all scenarios, though with a maximum of 2%. For scenario #5, sprat decreased for 18%. Looking at the age structure, it is apparent that mostly the adult sprat decreased. Besides the biological responses, abiotic factors do also react to the nutrient change. Fig 11 shows an increase in oxygen concentration in the bottom water layer for several polygons. The most impacted polygons are the coastal ones in the Western Baltic Sea and Baltic proper. Many of the small changes, caused by the scenarios, would be so small as to be written off as noise in the real world and should be treated with caution given the uncertainty of the model.

### Sensitivity to change in fishing mortality for key fish species

**Fig 12:**
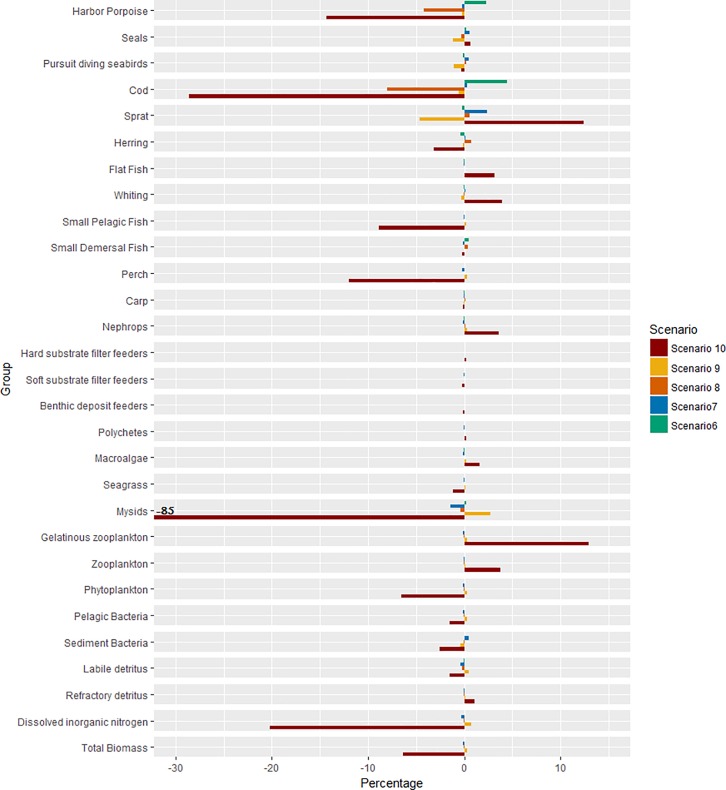
Results of the five fishing mortality sensitivity analyses (scenario #6–#10 respectively). Percentage of change of the biomass for the different biological functional groups compared to the status-quo scenario #1.

When inducing reduced fishing mortalities (Fig 12) for the main pelagic short lived forage fish species like sprat, and for a benthic long lived predatory fish species like cod, the model responds for the fished species or adjacent trophic levels. A 5% increase in cod biomass was observed when halving cod fishing mortality (#6) compared to the 2005 level. Similar, a sprat biomass increase of 2% was observed when halving the fishing mortality (#7). This is consistent with much lower sprat fishing mortality in 2005 than the cod fishing mortality, and that sprat fishing mortality in general is very low. In the last scenario with a more severe increase in fishing mortality, a greater decrease in biomass is simulated throughout the whole ecosystem (Fig 12, scenario 10). The effect goes from a decrease in harbour porpoises, up to -14%, but an increase in sprat and zooplankton, up to 12% and 4% respectively. The largest change is for the mysids, with a decrease in biomass of 85%.

### Fisheries economic evaluations of selected nutrient load reduction scenarios

There are very small decreases in the NPV (Net Present Value) for the total fishery between status quo and the four nitrogen reduction scenarios which are within the uncertainty levels of both models (Table 4). In scenarios #3 and #4, total NPV decreased by 4.1% and 1.7% respectively. The decrease in the Western Baltic is higher than the one in the Kattegat. There is a very small NPV decrease also in Kattegat in scenario 3, but it is on the third digit and therefore not shown in the table. The smaller trawling vessels (12–15 meters) increased their total revenue for both scenarios #3 and #4, while it decreased for the bigger trawlers and the gillnetters (Table T in S1 File). The fuel cost increased for the smaller vessels (12–15 and 15–18 meters) for scenario #3 and #4, for the bigger trawler vessels (18–24 meters) it decreased for scenario #3 but increased for #4, while it decreased slightly for the gillnetters (Table U in S1 File). The total costs for the small trawling vessels increased for scenario #3 and #4, while it decreased slightly or stayed the same for the other vessels (Tables V-Y in the S1 File). This explained the decrease in NPV for scenario #3 and #4. However, the total profit increased for the 12–15 meters trawlers, while it decreased for the others (Table Z in S1 File). Similarly as was shown above, the changes in scenario #2 were so small that also here they didn’t show any differences with the status quo scenario.

**Table 4:** NPV (mill EUR) from 2012 to 2037 for the four fleet segments for the baseline (scenario #1) and the three nutrient scenarios evaluated by FISHRENT (#2–#4). Results based on original 2012 local ICES-based assessed fishing mortality (F) and total stock biomass (TSB) for the groups and areas listed in Table 3, compared to using Atlantis-based F and TSB.

		Original F & TSB		Atlantis F & TSB	
Scenario	Sea	NPV (mill EUR)	Relative change (%)	NPV (mill EUR)	Relative change (%)
**Scenario #1**	**Total**	154.8		182,1	
	**Kattegat**	44.8		0,1	
	**Western Baltic**	36.4		37,9	
**Scenario #2**	**Total**	154.4	-0.26	182,1	0
	**Kattegat**	44.7	-0.22	0,1	0
	**Western Baltic**	36.3	-0.27	37,9	0
**Scenario #3**	**Total**	153.4	-0.90	174,7	-4.1
	**Kattegat**	44.4	-0.89	0,1	0
	**Western Baltic**	36.2	-0.55	37,7	0.5
**Scenario #4**	**Total**	153.3	-0.97	179.0	-1.7
	**Kattegat**	44.3	-1.12	0,1	0
	**Western Baltic**	36.1	-0.82	37,3	-1.6

Total NPV over all waters shown, together with NPV in Kattegat and NPV in Western Baltic.

## Discussion

A Baltic Atlantis model was implemented to integrate and better comprehend the complexity of the marine ecosystem when exploring effects of changes in different pressures on the marine ecosystem. Here we evaluated eutrophication and fishery pressures on the whole ecosystem on a spatial explicit scale. This established model framework will help us to evaluate the performance of alternative management strategies on mitigating human and natural pressures in the long term. We discuss the current model calibration and the realism of the baseline given the model uncertainty, as well as the application to nutrient load reduction scenarios and sensitivity analyses to fishing pressure levels to test the ability of the model to capture the dynamics in the ecosystem according to the changes. As the model is only for strategic use and due to the many assumptions, limitations and overall uncertainty, we only focus on the direction of changes in the relative evaluations rather than short term absolute predictions.

### Model parameterization and initialization

The system perspective supplied by the Baltic Atlantis model makes it a unique and useful tool for the region, enabling investigation of changes in eutrophication pressure while accounting for bio-physical interactions and feedbacks across all important trophic levels in all the basins of the Baltic. Nonetheless, the complexity of the nature of the interacting pressures on the system creates the limitation as it can be very difficult (even in a model) to separate the effects of a specific key factor, agent or process in the ecosystem, and quantitatively estimate its role under a given scenario. While the confounding can be somewhat controlled in the model it can not be completely removed as a faithful representation of the system requires a representation of the many interacting drivers. This issue is exacerbated by the fact that the model parameterization cannot be equally well constrained for all groups and processes. When looking at Nephrops, they could potentially respond to drastic shifts in biomass levels of their main prey, i.e. detritus as well as benthic species. On the other hand, they may also be affected by oxygen depletion with respect to changed mortality or distribution. Although the model seems to capture those interactions, which are evaluated in the scenarios, estimates of Nephrops have showed that their biomass has remained surprisingly constant over the last decade or more [80]. This is however, under the current environmental conditions with relatively high nutrient levels in their habitats. In the long term, Nephrops have most likely increased in Kattegat according to field observations. The parameterization of these interactions and potential feedback effects is complex and difficult to comprehend and evaluate without a well-calibrated ecosystem model. Better constraints on the simulated ecosystem dynamics, while still accounting for complexity, can only be achieved by increasing the input information to the model—including data availability and promoting coordinated monitoring efforts in different regions across the Baltic Sea. See details in section B.2-C in S1 File.

A lack of phosphorus cycling and cyanobacteria in the model also affects the model results in response to scenarios of reduced nutrient loading, especially in the Northern Baltic areas. While code now exists within the model framework for these components, it was not available in a timely manner and so has not yet been implemented for the Baltic. Cyanobacteria are likely to positively respond to significant reductions in nutrient inputs as they gain a relative advantage over diatoms, which primarily rely on the supply of new (non-regenerated) nutrients [16, 21]. Prevalence of blooms of nitrogen-fixing cyanobacteria in the main sub-basins of the Baltic Sea implies that nitrogen fixation is an important process that slows down the recovery from a eutrophic state (e.g. [16, 81]). However, the effects of this underrepresentation of nitrogen fixation is not expected to have an influence on the main commercial species such as cod, sprat and herring in the Baltic proper, Kattegat and Western Baltic areas, where the focus of the current implementation of the model is.

Not only sources but also sinks of nitrogen are very well represented in HBM-ERGOM, and [82] estimated that nitrogen removal by denitrification in sediments varied between 48 and 73% of the external nitrogen inputs delivered via rivers, coastal point sources and atmospheric deposition. In the Baltic Atlantis, in order to avoid a buildup of nutrients in some coastal boxes, especially along the eastern German and western Polish coasts (box #14), we introduced implicit decay terms of labile and refractory detritus to better balance the sources and sinks in the model. These processes are proxies for sediment burial and implicit effects of denitrification, adding to the likely underestimated model explicit denitrification, with overall aggregate sink rates being in line with the rates reported by [82].

Several such aspects limit the model capability to evaluate the results of scenario analysis in absolute terms and in the short term. However, the model is still useful for longer term projections and its set-up enables us to identify key sensitive parameters for which we need more information or need to make the foci of sensitivity analyses and robustness testing. This is a very important capacity of the modelling approach and management evaluation framework applications in general.

### Baltic Atlantis projections and dynamic full feedback mechanisms

#### Biomasses of biological functional groups

The current calibrated version of the Atlantis implementation in the Baltic meets the goal of simulating a stable and balanced marine ecosystem where all biological functional groups survive from one model year to another in a long term equilibrium state with biomass levels close to the best available knowledge (Figure E in S1 File). Despite the increased biomass level for the model output of sprat, it was still accepted as the 2005 sprat assessed abundances was considerably lower than all other years in the assessment year range [83]. In general, the model stability reached after the spin-up period (section B.5 in S1 File), is on a satisfactory level, however it should be noted that the available input data and information on the lower trophic levels are uncertain. Overall, the current calibration of the model can run uninterrupted for at least 120 simulation years, without any groups going extinct from year to year and season to season (Figure B in S1 File).

The biomasses of most of the functional groups exhibit a seasonal oscillation around the same mean biomass level. Planktonic groups exhibit the largest seasonal variability as their biomasses change more rapidly with environmental changes. In our model, benthic organisms exhibit little if any seasonal variability which is in line with model results of [23]. However, for Baltic benthic fauna, field observations reveal significant inter-annual variability in particular in response to prolonged changes in oxygen concentration and predation patterns. Furthermore, seasonal changes in coastal hypoxic conditions may also influence benthic biomass losses as perceived in the evaluation of the nutrient load scenarios (see below). A more complete picture of the interactions and flows between the different groups can be achieved by looking at the production instead of the biomass. This was however not possible in the current setting of the model. We argue though that, achieving equilibrium for the biomass for each of the functional groups, also means that the production is in equilibrium. This is because biomass would most likely not reach equilibrium if production is not in equilibrium.

#### Diets of biological functional groups

The results of the emergent fish dietary pattern are in accordance with our current knowledge. We can compare this to stomach data observations synthesized and modeled for example in the SMS model, previous estimates in mass balance models such as EwE (e.g. [32–33]), or reported diet and consumption estimates in published regional studies. The age-specific diet allows for a different diet for adults versus juveniles. So does the diet of adult cod consists of a greater proportion of larger planktivorous fish, such as sprat and herring (Figure F in S1 File, panel 4), while the diet of juvenile cod constitutes mainly of polychaetes and benthic crustaceans, such as the isopod *Saduria entomon*, which is an important food source for the early life stages of cod [84]. This is in general accordance with observations from stomach sampling programs and from SMS output [85]. The results also indicate that the model is capable of incorporating bentho-pelagic couplings. Although we allow for a cod cannibalism interaction in the model, this process is still underestimated in the current model version. For a relatively small population of harbour porpoise in the Western Baltic, there are accurate data concerning their diet composition [86]. As expected, cod constitute around 80% of the diet composition in the model with an underestimation of whiting, the second most important prey item (Figure F in S1 File, panel 1). The dietary patterns of the grey seal were observed from stomach sampling programs in the Baltic Proper and in the Gulf of Bothnia [87]. Observations show that the majority of their diet consists of herring, with sprat and cod being the second most important prey species, followed by carps and perches. This is similar with the diets modelled with Atlantis, except that sprat is an equally important prey species next to herring for adult seals (Figure F in S1 File, panel 2). However, the study dated from 2010, and the geographical overlap between the species, partly due to change in seal distribution, has very likely changed over time compared to the 2005 situation (and may have changed even more since 2010).

#### Population structure and demography

The distribution of numbers per age group shows a realistic exponential decline for all the age disaggregated functional groups, where the youngest individuals of a population are prone to the highest mortality. The age distribution is most even for seals because they have the most homogeneous survival rates once they survive the first vulnerable year. The abundance of that first age group is determined by the recruitment. Considering that exact mechanisms controlling recruitment are still not fully understood despite abundant data available (e.g. cod; [88]) and many studies on the topic, or are simply not known (small pelagics, small demersals), or are principally mediated by the changing environment anyway (e.g. sprat; [89]), it is difficult to evaluate the potential uncertainty of the fish recruitment dynamics in the Baltic Atlantis.

#### Spatial distribution of biological functional groups

The spatial distribution shows no extinctions in any polygon for all the vertebrates, with biomass fluctuating seasonally. The higher fluctuations of the vertebrates represent migrations between spawning and feeding grounds. This is why for instance cod biomass in certain polygons reaches zero (this type of migration is further discussed in section B.2 in S1 File–Quarterly abundance distribution of vertebrates). The fluctuations of the phytoplankton biomass show a spatial variation in the amplitude of those seasonal biomass peaks. The differences are likely due to the coastal versus off-shore physical conditions. Coastal areas have a continuous high nutrient load due to the riverine inflow all year round and they exhibit the highest variations in temperature with the lowest temperature reaching zero [56]. Off-shore areas also have a continuous N load in the surface areas [50, 90] so primary production and standing stock biomass does not go to zero [56], but they show much less temperature fluctuations, especially with respect to the extreme minima. That is why we do not expect as high temperature driven fluctuations in primary production in the off-shore areas as we see in the coastal ones (Figures D and M in S1 File).

However, it should be noticed that the limitation of finding vertical/horizontal mixing parameters could lead to a lack of adequately representing the nutrient distribution, stratification and so the primary production, despite extensively testing these parameters. On top of that, there exists only limited knowledge on the spatial distribution of the phytoplankton communities in the Baltic Sea due to the limited spatial and/or temporal coverage of previous research [91]. Similar for the Baltic total zooplankton biomass (microzooplankton, mesozooplankton and gelatinous zooplankton), the grazing and its distribution patterns in the Baltic Sea likely remains the least constrained level of the ecosystem. This is partly due to similar challenges in adequately sampling and monitoring, and partly due to the large fluctuations arising from observed patterns that also show high fluctuations on narrow spatial and temporal scales in different water layers. In the Bornholm Basin there are spectacular shifts in observed zooplankton community composition and grazing pressure (e.g. [67]), and although this area is sampled regularly, it still imposes many difficulties and challenges for model attempts in relation to those specific processes. Future improvements in properly constraining this ecosystem bottleneck will be fundamental to the successful evaluation of ecosystem-wide interactions in the Baltic Sea realm.

Another proxy useful for evaluating the geographical distribution of total phytoplankton biomass is the surface Chl-a (Fig 4). The reason why Atlantis seems to overestimate the Chl-a in coastal waters for the Western Baltic, the Gulf of Riga and Gulf of Finland regions, compared to the ERGOM model, could be because of the spatial resolution of the two models, where the ERGOM model has a very fine resolution in comparison with the coarse Atlantis polygon sizes. However, the ERGOM model also tends to underestimate the surface Chl-a concentration when compared to observational data, especially in the offshore Bornholm Basin and Gotland Basin [56] which makes the Atlantis estimates closer to the observations.

In the offshore polygons, Atlantis simulates quite similar surface Chl-a concentrations relative to the HBM-ERGOM output, except for the northern Bothnian Sea where Atlantis has a slightly lower estimate of the Chl-a concentration in the off-shore polygon. This may be explained by the lack of cyanobacteria in the Atlantis in comparison with HBM-ERGOM. The current Baltic Atlantis results are calibrated without considering phosphorus and carbon cycling. Consequently, cyanobacteria are not included in the model simulations since they are mostly distinguished from other pelagic primary producers by a higher phosphorus requirement for growth. In the Baltic Sea, nitrogen-fixing cyanobacteria contribute significantly to primary production, especially during the summer months in the northern Baltic Proper and more Northerly Baltic areas (e.g. [92]).

Lack of cyanobacteria probably does not affect the secondary production in the model, because they are not an important food source for zooplankton. Sedimentation and subsequent degradation of cyanobacteria are also not included in Atlantis and may lead to slight underestimation of hypoxia in the open areas of the model. Not accounting for these phenomena is unlikely to compromise the results of the current analyses because the salinities in the Kattegat, Western Baltic regions and southern Baltic proper regions, except in the immediate vicinity of the coast, are beyond the typical <12 salinity range where cyanobacteria appear in the Baltic [66]. Finally, the attenuation of light in the Baltic Atlantis water column accounts for total phytoplankton biomass but does not explicitly represent attenuation due to colored dissolved organic material (CDOM). The potential overestimation of light levels due to lack of CDOM as well as cloud cover effects on light availability are partially offset by adjusting the light attenuation coefficients of phytoplankton groups.

Furthermore, HBM-ERGOM outputs reveal that in Kattegat and the Western Baltic there exist very sharp gradients between very narrow but highly productive zones and offshore areas with low annual average phytoplankton standing stocks. These features are unlikely to be fully captured in Baltic Atlantis without a more detailed map of time-varying riverine and point source inputs to the model, such as the one offered by the DK-QNP model used to force HBM-ERGOM. Although such high spatial resolution details need not be considered to force the dynamics of fisheries, they are more important in better constraining biogeochemical cycling, and its control on seasonal and inter-annual variability in primary production.

### Nutrient load reduction scenarios

The nutrient load reduction scenarios allowed us to test our newly developed model in its responsiveness to system disturbances. The nutrient load reduction scenarios where chosen because the Baltic ecosystem is already characterized as a highly eutrophic system [10, 16]. The scenarios are purely for testing the model though and do not reflect an actual reduction plan. The four different scenarios gradually increase the reduction of nitrogen in the ecosystem. This is done by including more and more river point sources, linked to certain polygons, which carries a reduced amount of nitrogen. Even though the spatial scale of the Atlantis polygons might, to some extent, limit the resolution of fully representing bio-geochemical and primary production responses to changes in eutrophication pressures, it still allows for the separation of different processes in narrow coastal versus larger off-shore polygons (section B.1 in S1 File). This spatial explicit approach is necessary because the eutrophication issue in the Baltic Sea is on such a large scale. Besides the spatial explicit approach, the interactions of physical with biological processes need to be included to investigate this issue, which is why this end-to-end model is applied.

In the model, it is mainly the dietary interactions that determine the response of planktivorous and piscivorous fish biomass for the analyzed nutrient reduction scenarios. However, the model parameterization could not be equally well constrained for all groups and processes. For instance, the prey-predator interactions involving cod, sprat and herring are well informed using abundant field data and robust model results from the Baltic SMS or EwE. On the other hand, there is little data to quantitatively describe the dietary interactions of many benthic animals, or if locally available, the issue of extrapolating accurately over many types of habitats remains. Benthic organisms, especially in the coastal zones, are known to be susceptible to changes in ambient oxygen conditions [23], strongly affected by riverine nutrient inputs. As a result, the biomass fluctuations in the nutrient load scenarios of these less constrained groups could strongly affect the development of their predators, such as juvenile cod, or other bottom dwelling fish. (See also section B.2 in S1 File–Demographic profiles, mortality rates & reproduction functions).

#### Biomass

For all scenarios, the overall biomass decreases, with a stronger signal when there are large reductions in river load. This is in line with the results from [21] who used a NPDZ model to show that a reduction in nutrients lead to a decrease in biomass for diatoms, flagellates and zooplankton, with a maximum decrease in primary producers. According to the Atlantis results, there is a bottom-up and cascading effect where the change is observed for all trophic levels [93]. However, there is no consistent pattern found for all functional groups because of the complex foodweb interactions that the model comprehends. The amount of change differs also from polygon to polygon with higher changes in the near coastal ones as they are more directly affected by the river inflow, which is also shown in other studies [21]. The changed fish biomass under the scenarios of decreasing fishing mortalities on commercial fish stocks is much smaller than the uncertainty of the model (i.e. difference due to an altered set of initial conditions and uncertainty of the input data). However, the scenario #5 leads to a sprat biomass reduction of 18% and an increased cod biomass levels as was expected. The largest decrease of biomass for scenario #5 was that of the mysids. The mysids react only for the last two scenarios though. This is because of the way they are spatially distributed. Their limited spatial occurrence does not mean that their production is equally limited. Especially because sprat and herring have a broader spatial occurrence and do feed for a large part on the mysids.

Significant changes in Nephrops and detritus biomasses were as expected for the nutrient load reduction scenarios. For scenario #3 and #5, Nephrops decreases around 20% due to a decrease in their main food source, detritus. Labile and refractory detritus are a pool of fixed nutrients from faeces and non-predation mortality products. The decrease of this detritus pool is caused by a decrease of production of the rest of the ecosystem due to the reduced river load. In the scenario following the Baltic action plan, the Nephrops stock is reduced by 11%, less than for other scenarios. Consequently, the model response is proportional to load reduction and shows expected patterns and levels of effects on the different functional groups given current knowledge. This can be seen in context of a general propagation of the effect of the river load reductions through the whole ecosystem. When the small changes in biomass, which are generated in the scenarios, accumulate, then this might lead to a more extensive change of the detritus pool. This in turn can have an effect on those groups feeding on detritus, such as filter feeders, polychaetes, deposit feeders and Nephrops. Those multitudes of interactions is an argument for using an end-to-end ecosystem model to comprehend the more realistic perception of effects of nutrient load reductions taking into consideration the broader and complex biological processes, effects and interactions.

#### Oxygen

Distinct benthic oxygen concentration increases were simulated in many areas according to nutrient load reductions, especially in the Western Baltic and the Baltic proper areas while less distinct in the deeper areas (Fig 11). Oxygen conditions in the bottom water layers improved with up to two to three times the amount of oxygen for scenario #4 and #5 compared with the baseline. Reducing the river load caused a reduced production of biomass which presumably lowered the need for oxygen in order to decompose all the organic matter. It must be emphasized though, that the benthic-pelagic coupling and sediment processes in general require further calibration and validation before we can apply a greater certainty to the above and below model output.

The increase of cod biomass might be linked with the increase in oxygen concentration for two reasons. First of all, benthic invertebrates have a higher survival chance due to the increased oxygen conditions. They are also the main food source for juvenile cod, especially the isopod *Saduria entomon* [84]. Secondly, the improved hydrographical conditions in the bottom water layers may have a positive effect on cod egg survival as it increases the reproductive volume [94]. This is the volume of water in which the cod eggs develop and it is the key driver of its recruitment success. The reproductive volume is delimited by a concentration of oxygen >2mL L-1 and a salinity of >11 psu [94–96]. While we didn’t explicitly represent oxygen effects on the egg and larval stage it would affect new settlers so the model captures some of the potential recruitment effects, but not all.

The simulations mainly show an increase for the cod age-groups of 2–4 and rather than in the 1^st^ age group. This implies that the increase in food sources for the juvenile cod has a larger effect than the higher survival of its eggs (Figure L in S1 File). So, despite an increased production for the benthic organisms, their total biomass decreased due to accentuated predation by juvenile cod.

Anoxic zones have been known in the Baltic for long time and are severely impacting the ecosystem [96–100], because benthic species are unable to escape. Anoxic zones cause severe losses on entire benthic communities, which are seen as one of the causes for changed levels of fish catches since the 1970s combined hydrographical driven fish recruitment patterns [101–105].

### Sensitivity to change in fishing mortality for key fish species

When halving the fishing mortality, we did not see a doubling of the biomasses. This effect could be expected from a single species model, but not from an ecosystem model where the changes in fishing mortality results in changed biological interactions. As such, we for instance see cascading changes in the ecosystem food web which mitigate and buffer the effects. This is another reason that well-informed and evaluated complex ecosystem models are necessary to comprehend and project the full and long-term effects of changed fishing pressure.

The cod and sprat functional groups show expected effects of increased biomass when fishing pressure decrease and vice versa. The smaller reaction of sprat in comparison to cod when halving the fishing mortality is because of the already low fishing mortality for sprat in the Atlantis model, i.e. 0.07 in 2005. When the maximum observed fishing pressure of cod and sprat observed in recent years stock assessments ([80, 83, 85, 106] 1.2 and 0.6 respectively) is applied, then we see a stronger top down effect throughout the whole ecosystem. This stronger effect, propagating over multiple trophic levels, is off course to be expected since there is a large difference between the fishing mortality of scenario #8 and #9 versus #10. However, the different fishing mortalities do not have the purpose of testing different management scenarios, rather trying to see whether the model is reacting to and realistically reflecting changes in fishing pressure, which is shown with this example.

### Fisheries economic evaluations of selected nutrient load reduction scenarios

The NPV decrease is according to the model framework caused by the lowered catch possibilities given the lower biomass when nitrogen load reduction is imposed. However, a biomass decrease does not necessarily leads to a NPV decrease. Lowered catch possibilities can, in some cases, also lead to reduced fishing costs, and thus an increase in NPV. Furthermore, given the reduced catch possibilities which are different for the different species, reallocation of effort among fisheries may actually lead to higher catches overall and thus higher catch values for some fleets [63–64] in such a case the fisheries management system would not constrain the catches with TACs.

Even though the Nephrops is a commercially high valuable species, its 10–20% reduction in biomass did not seem to have a linear response in the FISHRENT output as there was no difference in the NPV for the Kattegat area, where Nephrops is caught. There are two reasons for this. Firstly, Nephrops is a choke species in FISHRENT. This means that a vessel can be forced to stop fishing because it exhausted the quotas for Nephrops, despite underutilized quotas for other species. Accordingly the fishery and its outcomes are not directly dependent on the Nephrops stock biomass as it has a constant quota set, which cannot be exceeded. The likely reason why the decrease is most pronounced in Western Baltic is due to the decrease in the herring stock in scenario 3 as the TAC of herring is not choking. The quotas were kept constant and equal to the values in Table J in S1 File. Secondly, the FISHRENT model does not distinguish the fleets between the Western Baltic and the Kattegat, i.e. it considers the catches of the four fleets over a year in both areas. Thus, if less is caught of Nephrops leading to reduced NPVs for the fleet segments catching Nephrops this decrease is distributed proportionally on Kattegat and the Western Baltic according to the revenue and cost distribution keys.

### Perspectives and future developments within the framework

There are a number of ongoing or planned developments within both Atlantis and the coupled framework. First of all, inclusion of more finely resolved vertical mixing will enable us to better capture the seasonal pattern of the nutrient distributions and processes such as changing mixed layer depth. This will enhance the seasonal cycle of phytoplankton and of oxygen mixing/non-mixing due to stagnation. Secondly, both levels and equilibrium of the production of the different functional groups will be evaluated besides the biomass in Atlantis. Next, the constant linear fishing mortality, applied equally across the whole Baltic Sea will be replaced in Atlantis by a spatial- and fleet specific fishing effort and mortality levels (resolved to the level of the spatial polygons in the model). This will also include information on fisheries economics such as revenue, costs and profit (Gross Value Added, GVA) to enable full fisheries economic analyses within the Atlantis model. Accordingly, the management- and economic module within the Atlantis model will be elaborated to avoid the need for an external bio-economic model and to dynamically model the feedbacks in the underlying processes. Lastly, the model sensitivity of the Atlantis model according to forcing from different bio-geo-chemical and hydrodynamic forcing models will be extensively explored.

## Conclusion

In this paper we have introduced a new calibrated Baltic Atlantis model and an integrated end-to-end modelling framework to examine ecosystem-wide responses under scenarios of human-induced changes in the Kattegat, Western Baltic Sea and southern Baltic proper regions with a focus on eutrophication, nutrient load reductions, and sensitivity to fishing pressure and climate factors. The Baltic Sea Atlantis model presented here for the first time forms the core of the established integrated framework. We have described the current setup of the framework with focus on the sensitivity of the Atlantis model to nutrient load reduction scenarios and fishing pressure changes; this demonstrates how the framework can be used to evaluate ecosystem and economic impacts: HBM-ERGOM, Baltic Atlantis and FISHRENT (noting that the FISHRENT KWB component of the framework only covers the Kattegat and the Western Baltic). Using four hypothetical eutrophication scenarios we demonstrated the capability of Atlantis and the framework to evaluate resulting changes in biological production and their long term biological consequences and economic impacts on the fishing catch sector, also taking into consideration fishing pressure as another anthropogenic impact. In light of the current assumptions and model limitations, we also have discussed the current and envisaged future improvements to the Atlantis model and the framework. The models have the potential to help evaluate conflicting objectives and alternative management strategies for better strategic management of human and natural pressures in relation to impacts on marine resources and in relation to an ecosystem based approach to management taking into consideration a sufficient complexity of processes and biological interactions in the ecosystem and the technical interactions in the fishery. They can also help with identifying knowledge gaps and recommendations for addressing them, e.g. regular benthos monitoring, and integrated data management for Baltic States.

Integrating marine ecosystem components for the purpose of providing integrated ecosystem advice currently relies primarily on an array of indicator-based assessment tools [107]. In the Baltic Sea, ecosystem status is evaluated both as a response to an individual pressure (using HELCOM HEAT 3.0; [16]) or as a cumulative effect of many pressures via an integrated ecosystem assessment (HELCOM HOLAS; [10]). Such a purely statistical basis for advice can neither explain the underlying dynamics, processes, and mechanisms of change, nor can it project future responses to potential scenarios of altered pressures. Our proposed integrated (and in the long term perspective marine spatial planning and cross-sector) modelling platform offers means of an exploratory analysis of future scenarios of ecosystem-state responses to human-induced pressures, here demonstrated on the example of the eutrophication process impacting across fish stocks and fisheries. Further development of the Atlantis model and the modelling framework can be complementary to the indicator-based assessment tools (e.g. HELCOM HEAT and HOLAS tools) and, thus, be of interest to numerous stakeholders and policy makers interested in sustainable ecosystem-based management of marine resources in the Baltic Sea waters. In particular, the Atlantis-based framework can serve as a powerful holistic and long term strategic management strategy evaluation tool for such governing organizations as the ICES Working Group on Integrated Assessments of the Baltic Sea (ICES WGIAB) and their joint efforts with the HELCOM Group for the Implementation of the Ecosystem Approach (HELCOM GEAR), which has been established with the purpose of steering a managerial level process of successful implementation of the HELCOM BSAP. This is in order to help with meeting the ecological objectives and achieving good ecological/ environmental status of the Baltic Sea by 2021 at the latest.

## Supporting information

S1 File Supporting Information A. The Baltic Sea Atlantis:
File A. Input data of the tracers per box for the Baltic Atlantis
File B. Input data of the tracers per box and layer for the Baltic Atlantis
File C. Input data for the fill values for the tracers for the Baltic Atlantis
Figure A. Schematic diagram illustrating the structure of the coupled HBM-ERGOM model system
Figure B. 120 year simulation run
Figure C. The FISHRENT model diagram, here applied to Kattegat and Western Baltic.
Figure D. One-year cycle of Chl-a in the different polygons
Figure E. Relative biomass–initial condition values compared with simulation outcome
Figure F. Diet composition of all predators
Figure G. Biomass per age group over time for all vertebrates
Figure H. Demography distribution for all vertebrates—the number of individuals for each age group
Figure I. Geographical distribution of all functional groups
Figure J. Geographical distribution of oxygen in the different layers. Panel 1 = top layer, panel 7 = bottom layer
Figure K. Total biomass of Cod for scenario 1 (baseline) compared to scenario 5
Figure L. Relative prey biomass for predator cod, baseline compared to scenario 5
Figure M. One-year cycle of nutrients in the different polygons
Table A. Physical and geochemical parameters used to internally force the Baltic Atlantis model.
Table B. Summary of riverine + direct point source waterborne nitrogen loads applied to the Baltic Atlantis grid based on information from the Review of the Fifth Baltic Sea Pollution Load Compilation for the 2013 HELCOM Ministerial Meeting (HELCOM PLC-5.5). Nitrogen fractionation between DIN and DON based on Savchuk et al. (2012). Bioavailable fraction of DON assumed equal to labile DON as in Savchuk and Wolff (2009). Coastal retention fractions from Savchuk and Wolff (2009).
Table C. Summary of key sources used to inform the biological module of Baltic Atlantis in relation to abundance and biomass, demography, prey-predator interaction and other functions.
Table D. Summary of key biological parameters used for vertebrates in the Baltic Atlantis model.
Table E. Maximum potential growth rates and clearance rates per age class of all vertebrate biological groups.
Table F. Key biological rates calibrated for the invertebrate groups.
Table G. Biological functional group structure of the Baltic Atlantis model. Groups are categorized according to the ecosystem level they represent. Key species aggregated within a functional group are listed. The first species in each list is the representative one for that particular functional group. Groups that are currently “turned off”, and which do not play a role in the biological interactions, are marked with an asterisk (*).
Table H. Summary of basic parameters describing the general behavior of biological groups in the model, used as input to the model.
Table I. Relative cover of each polygon with 3 types of bottom abiotic habitats: bedrock, sand, mud, and a fourth abiotic habitat which corresponds to man-made structures such as wind-mill parks, pipelines etc.
Table J. Total Stock Biomass, TSB (tonnes), and natural mortalities (M), target fishing mortalities (F) and TACs (tonnes) in 2012, for the seven species groups included in the FISHRENT model and TSB and F from the Atlantis model, also input for the FISHRENT model.
Table K. Danish fleet segments operating either in the Kattegat (KA), the Western Baltic (WB) or in both areas (KAWB) in 2012.
Table L. Catch value and catch value distribution for the 9 Danish fleet segments operating in the Kattegat and/or the Western Baltic in 2012.The table shows that Netters and liners less than 12 meters, and Trawlers 12–15 meters, 15–18 meters and 18–24 meters cover more than 85% of the total catch value. It has therefore been decided to focus on these four fleet segments in the model evaluations.
Table M. Economic input data for the 4 fleet segments included in the FISHRENT model, based on 2012 data.
Table N. Species prices (1000 €/tonnes) based on 2012 landings data.
Table O. Revenue and effort Fractions in Kattegat and Western Baltic of total Revenue obtained and effort exerted in 2012 in all waters by the four fleet segments included in the FISHRENT model.
Table P. Catch value fraction (%) of species included in the FISHRENT model of total catch value in 2012 for the four fleet segments included in the model.
Table Q. TAC shares for the four fleet segments included in the FISHRENT model for each of the 7 species groups.
Table R. Cobb-Douglas intercept parameters (tonnes) used in the FISHRENT model
Table S. Annual average total Baltic Sea biomass [metric tons] of all active biological groups in the Baltic Atlantis model averaged over the last five years (from a total of year 60). For vertebrates, for which good constraints on abundance/biomass distributions were available, “x initial” factor difference as relative to initial/expected conditions is given as well.
Table T. Total revenue (mill EUR) over the period 2012–2037 for the four fleet segments in each of the four scenarios. Revenue is shown over all waters shown, together with revenue in Kattegat and in the Western Baltic
Table U. Total fuel costs (mill EUR) over the period 2012–2037 for the four fleet segments in each of the four scenarios. Fuel costs are shown over all waters shown, together with fuel costs in Kattegat and in the Western Baltic
Table V. Total crew costs (mill EUR) over the period 2012–2037 for the four fleet segments in each of the four scenarios. Crew costs are shown over all waters shown, together with crew costs in Kattegat and in the Western Baltic
Table W. Total variable costs (mill EUR) over the period 2012–2037 for the four fleet segments in each of the four scenarios. Variable costs are shown over all waters shown, together with variable costs in Kattegat and in the Western Baltic
Table X. Total capital costs (mill EUR) over the period 2012–2037 for the four fleet segments in each of the four scenarios. Capital costs are shown over all waters shown, together with capital costs in Kattegat and in the Western Baltic
Table Y. Total fixed costs (mill EUR) over the period 2012–2037 for the four fleet segments in each of the four scenarios. Fixed costs are shown over all waters shown, together with fixed costs in Kattegat and in the Western Baltic
Table Z. Total profits (1000 EUR) (mill EUR) over the period 2012–2037 for the four fleet segments in each of the four scenarios. 'Profits are shown over all waters shown, together with profit in Kattegat and in the Western Baltic
(ZIP)
