# Supplementary material for: The Baltic Sea Atlantis: An integrated end-to-end modelling framework evaluating ecosystem-wide effects of human-induced pressures
Source: PLoS One. 2018 Jul 20;13(7):e0199168. doi: 10.1371/journal.pone.0199168 (PMC6054375; doi:10.1371/journal.pone.0199168)
Supplement: S1 File — Supporting Information A. The Baltic Sea Atlantis: File A. Input data of the tracers per box for the Baltic Atlantis File B. Input data of the tracers per box and layer for the Baltic Atlantis File C. Input data for the fill values for the tracers for the Baltic Atlantis Figure A. Schematic diagram illustrating the structure of the coupled HBM-ERGOM model system Figure B. 120 year simulation run Figure C. The FISHRENT model diagram, here applied to Kattegat and Western Baltic. Figure D. One-year cycle of Chl-a in the different polygons Figure E. Relative biomass–initial condition values compared with simulation outcome Figure F. Diet composition of all predators Figure G. Biomass per age group over time for all vertebrates Figure H. Demography distribution for all vertebrates—the number of individuals for each age group Figure I. Geographical distribution of all functional groups Figure J. Geographical distribution of oxygen in the different layers. Panel 1 = top layer, panel 7 = bottom layer Figure K. Total biomass of Cod for scenario 1 (baseline) compared to scenario 5 Figure L. Relative prey biomass for predator cod, baseline compared to scenario 5 Figure M. One-year cycle of nutrients in the different polygons Table A. Physical and geochemical parameters used to internally force the Baltic Atlantis model. Table B. Summary of riverine + direct point source waterborne nitrogen loads applied to the Baltic Atlantis grid based on information from the Review of the Fifth Baltic Sea Pollution Load Compilation for the 2013 HELCOM Ministerial Meeting (HELCOM PLC-5.5). Nitrogen fractionation between DIN and DON based on Savchuk et al. (2012). Bioavailable fraction of DON assumed equal to labile DON as in Savchuk and Wolff (2009). Coastal retention fractions from Savchuk and Wolff (2009). Table C. Summary of key sources used to inform the biological module of Baltic Atlantis in relation to abundance and biomass, demography, prey-predator interaction and other functions. Ta [file pone.0199168.s001.zip › FigF.pdf]

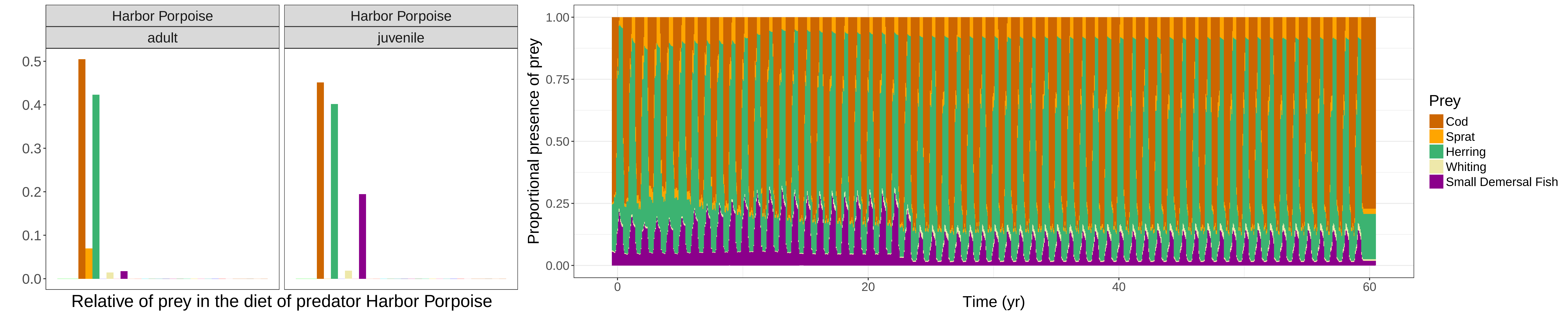

Harbor Porpoise
adult
Harbor Porpoise
juvenile
0.0
0.1
0.2
0.3
0.4
0.5
Relative of prey in the diet of predator Harbor Porpoise
Proportional presence of prey
0.00
0.25
0.50
0.75
1.00
0
20
40
60
Time (yr)
Prey
Cod
Sprat
Herring
Whiting
Small Demersal Fish

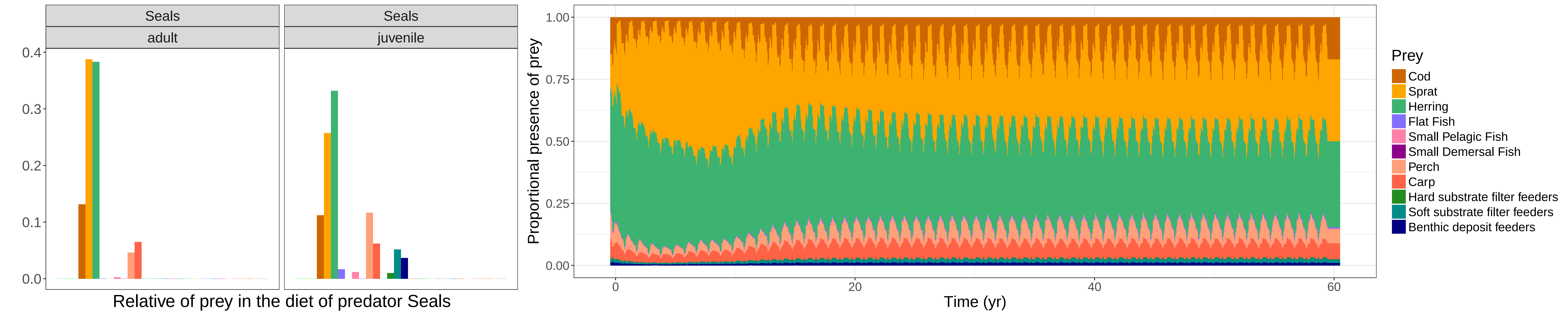
Seals
adult
Seals
juvenile
0.4
0.3
0.2
0.1
0.0
Relative of prey in the diet of predator Seals
1.00
0.75
0.50
0.25
0.00
Proportional presence of prey
0
20
40
60
Time (yr)
Prey
Cod
Sprat
Herring
Flat Fish
Small Pelagic Fish
Small Demersal Fish
Perch
Carp
Hard substrate filter feeders
Soft substrate filter feeders
Benthic deposit feeders

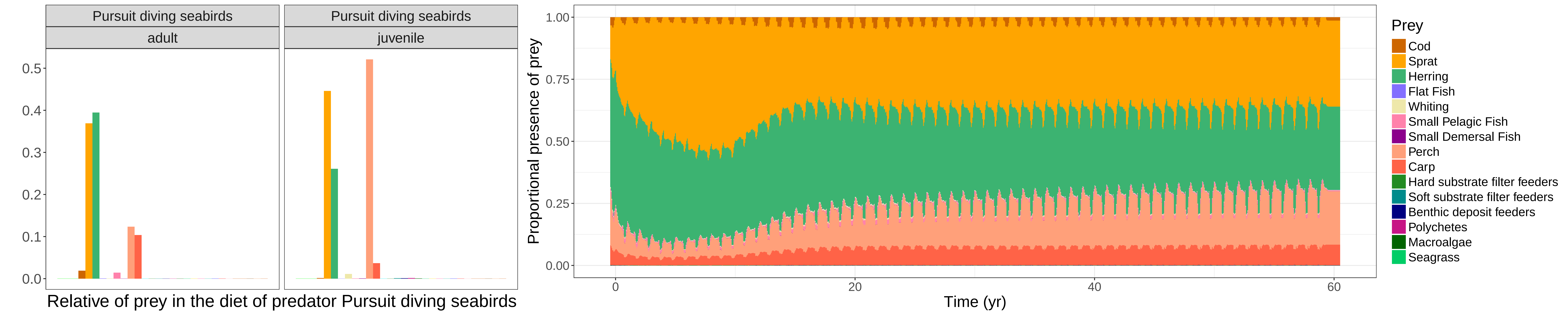
Pursuit diving seabirds
adult
Pursuit diving seabirds
juvenile
0.5
0.4
0.3
0.2
0.1
0.0
Relative of prey in the diet of predator Pursuit diving seabirds
1.00
0.75
0.50
0.25
0.00
Proportional presence of prey
0
20
40
60
Time (yr)
Prey
Cod
Sprat
Herring
Flat Fish
Whiting
Small Pelagic Fish
Small Demersal Fish
Perch
Carp
Hard substrate filter feeders
Soft substrate filter feeders
Benthic deposit feeders
Polychetes
Macroalgae
Seagrass

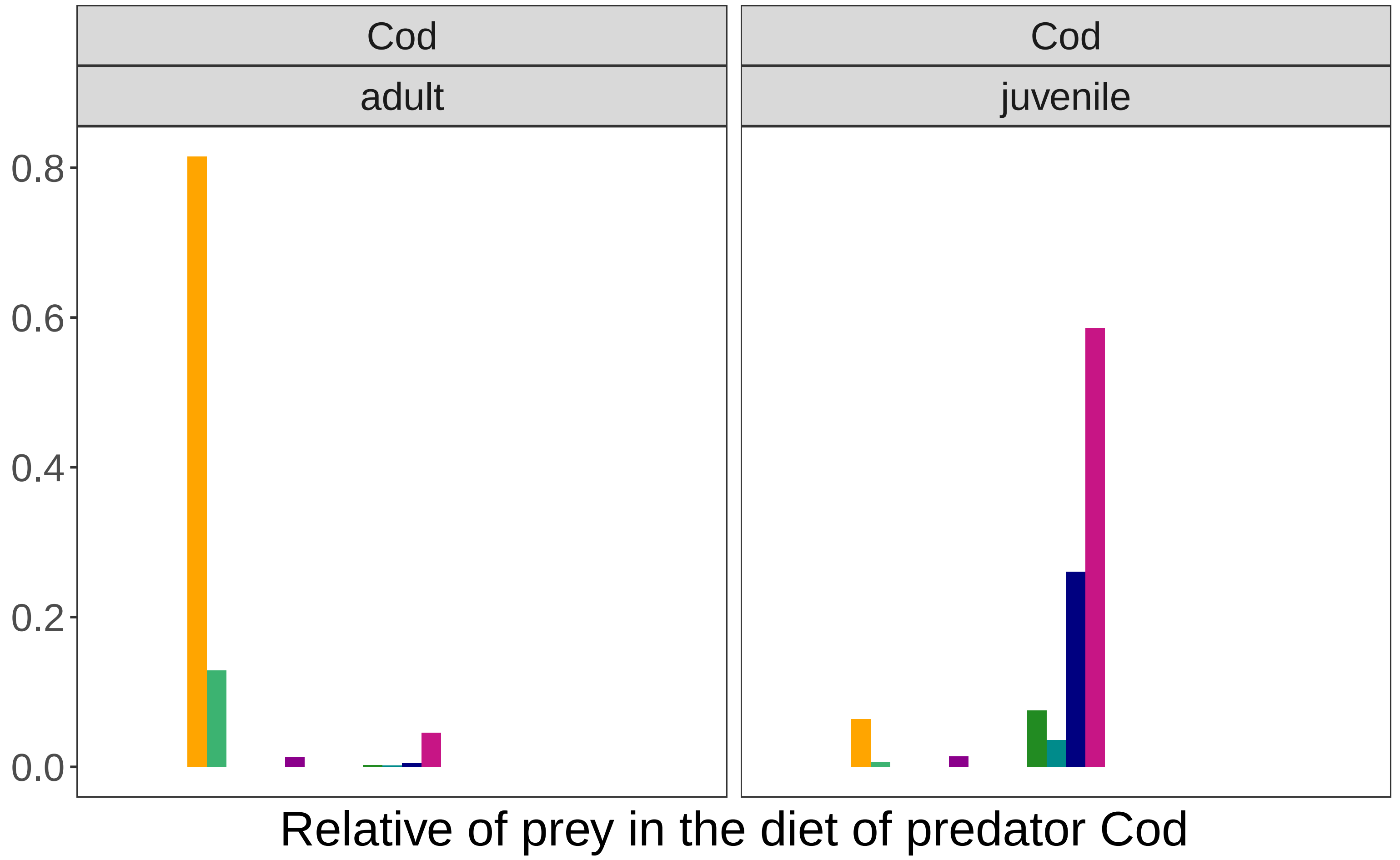
Cod
adult
Cod
juvenile
0.0
0.2
0.4
0.6
0.8
Relative of prey in the diet of predator Cod

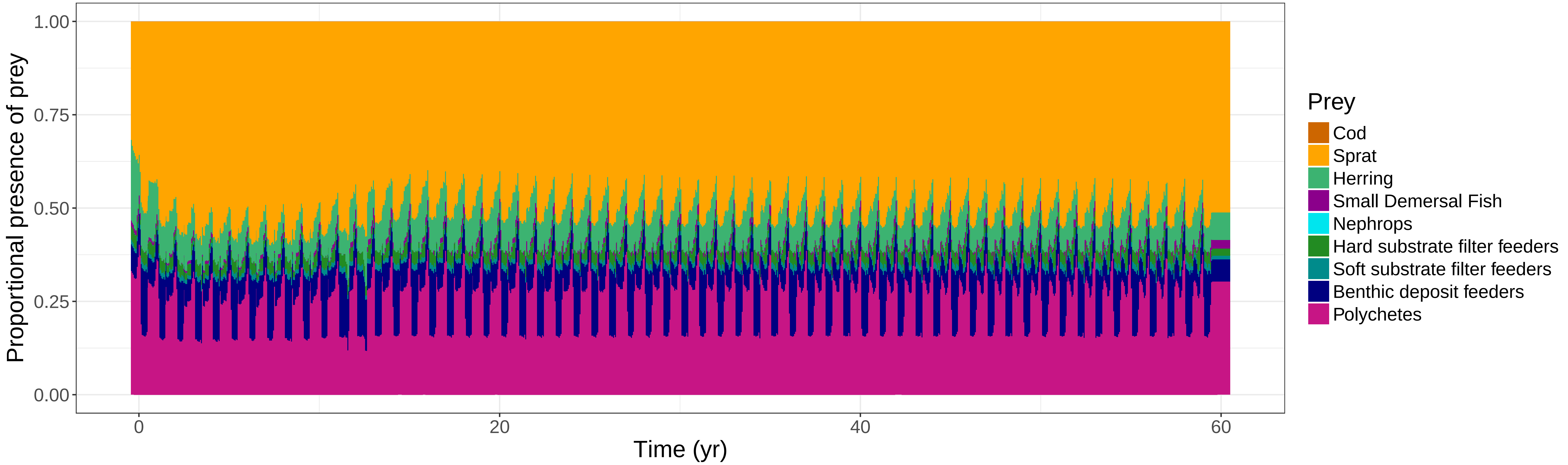
Proportional presence of prey
0.00
0.25
0.50
0.75
1.00
0
20
40
60
Time (yr)
Prey
Cod
Sprat
Herring
Small Demersal Fish
Nephrops
Hard substrate filter feeders
Soft substrate filter feeders
Benthic deposit feeders
Polychetes

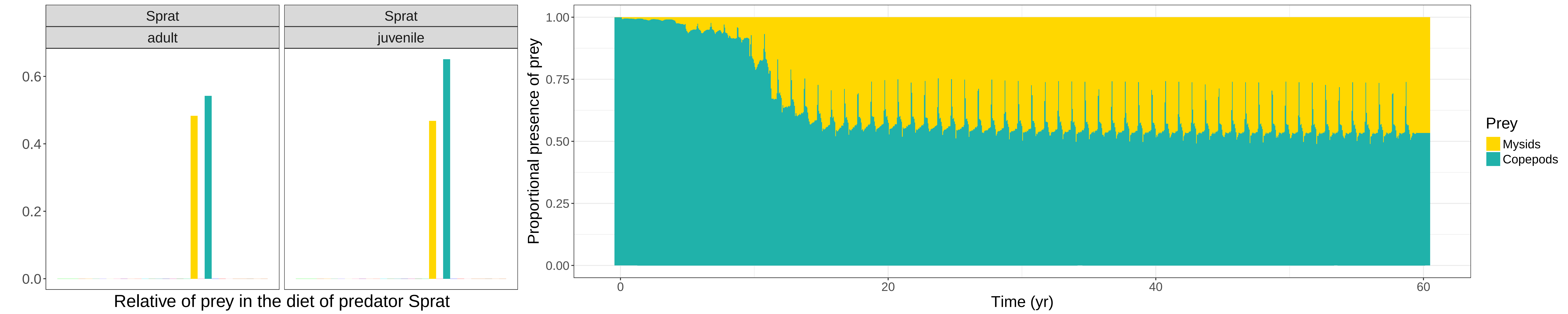
Sprat
adult
Sprat
juvenile
0.0
0.2
0.4
0.6
Relative of prey in the diet of predator Sprat
Proportional presence of prey
1.00
0.75
0.50
0.25
0.00
0
20
40
60
Time (yr)
Prey
Mysids
Copepods

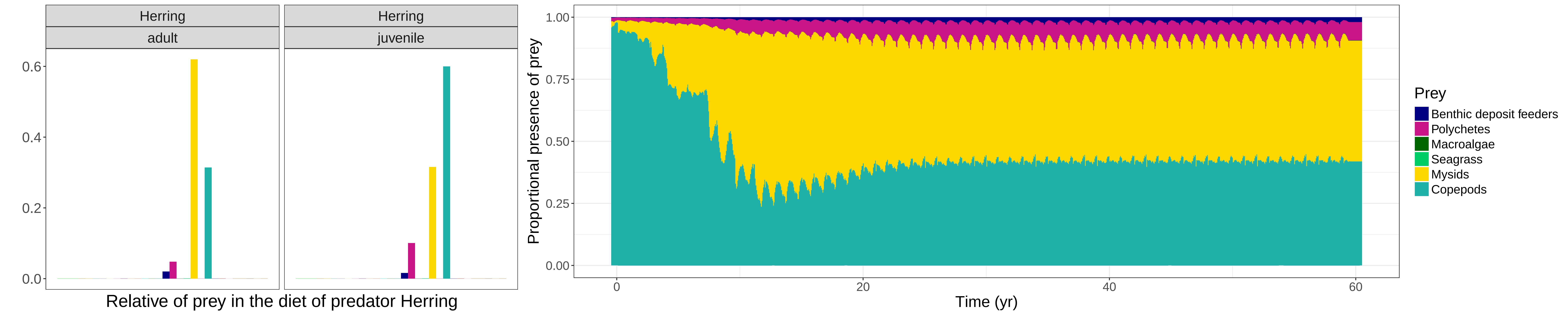
Herring
adult
Herring
juvenile
0.6
0.4
0.2
0.0
Relative of prey in the diet of predator Herring
1.00
0.75
0.50
0.25
0.00
Proportional presence of prey
0
20
40
60
Time (yr)
Prey
Benthic deposit feeders
Polychetes
Macroalgae
Seagrass
Mysids
Copepods

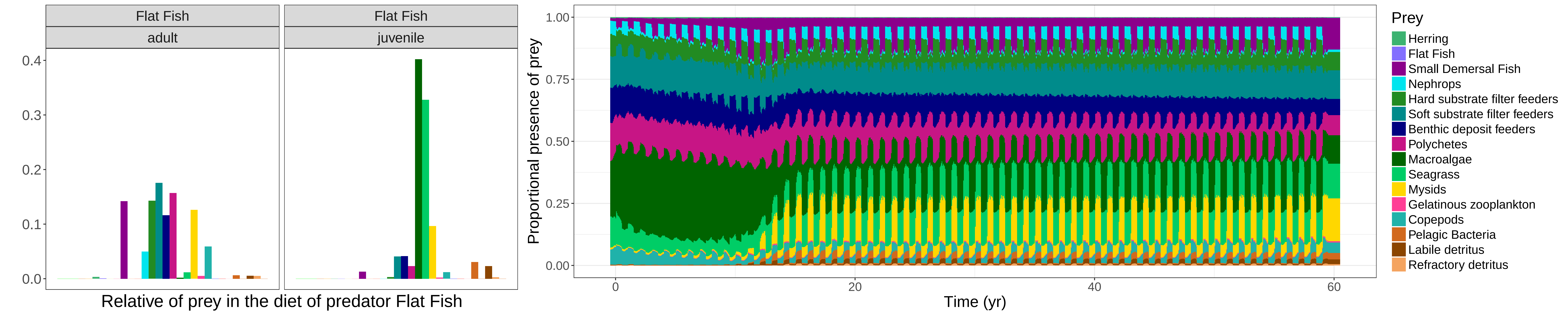
Flat Fish
adult
Flat Fish
juvenile
0.4
0.3
0.2
0.1
0.0
Relative of prey in the diet of predator Flat Fish
1.00
0.75
0.50
0.25
0.00
Proportional presence of prey
0
20
40
60
Time (yr)
Prey
Herring
Flat Fish
Small Demersal Fish
Nephrops
Hard substrate filter feeders
Soft substrate filter feeders
Benthic deposit feeders
Polychetes
Macroalgae
Seagrass
Mysids
Gelatinous zooplankton
Copepods
Pelagic Bacteria
Labile detritus
Refractory detritus

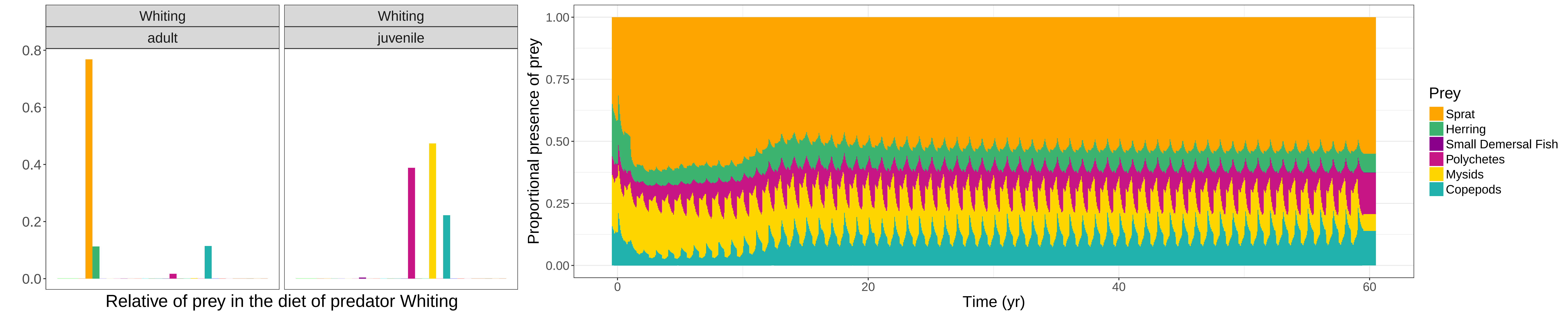

Whiting
adult
Whiting
juvenile
0.0
0.2
0.4
0.6
0.8
Relative of prey in the diet of predator Whiting
1.00
0.75
0.50
0.25
0.00
Proportional presence of prey
0
20
40
60
Time (yr)
Prey
Sprat
Herring
Small Demersal Fish
Polychetes
Mysids
Copepods

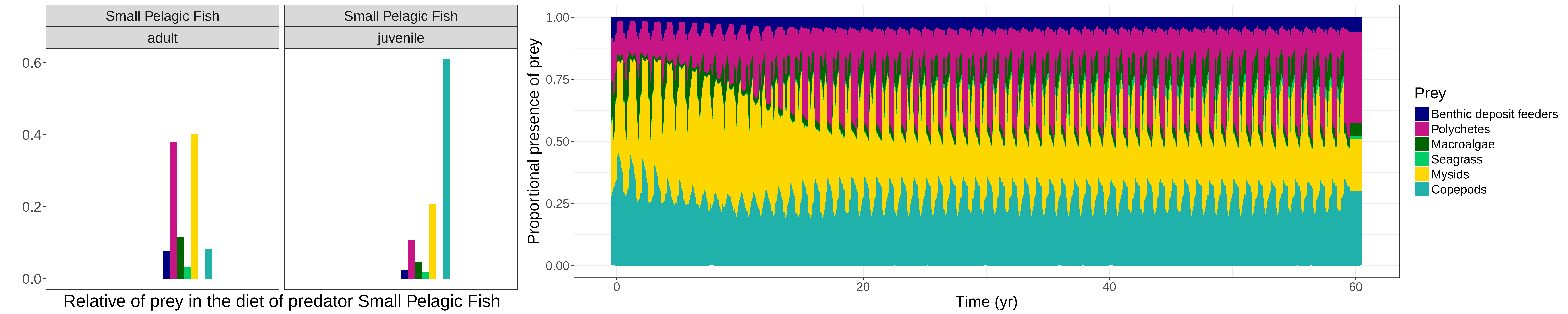

Small Pelagic Fish
adult
Small Pelagic Fish
juvenile
0.6
0.4
0.2
0.0
Relative of prey in the diet of predator Small Pelagic Fish
1.00
0.75
0.50
0.25
0.00
Proportional presence of prey
0
20
40
60
Time (yr)
Prey
Benthic deposit feeders
Polychaetes
Macroalgae
Seagrass
Mysids
Copepods

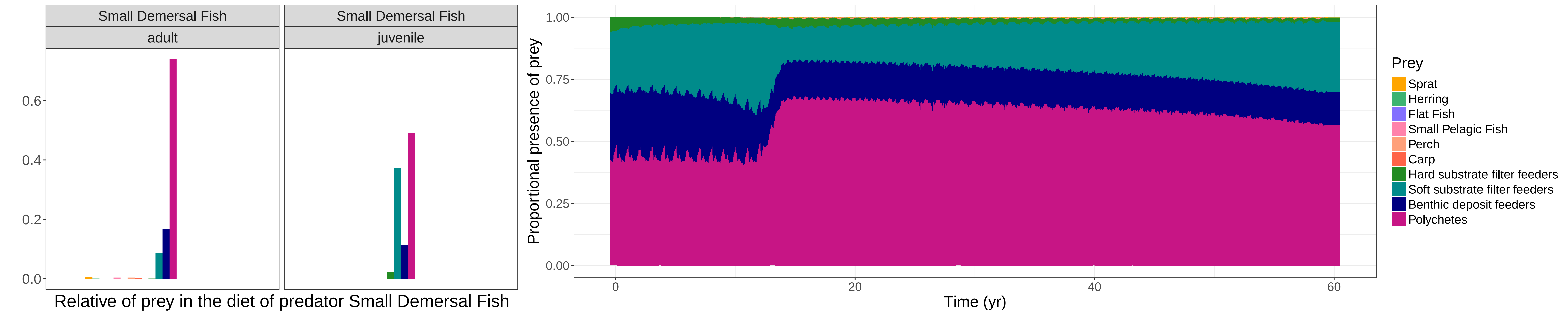

Small Demersal Fish
adult
Small Demersal Fish
juvenile
0.0
0.2
0.4
0.6
Relative of prey in the diet of predator Small Demersal Fish
Proportional presence of prey
1.00
0.75
0.50
0.25
0.00
0
20
40
60
Time (yr)
Prey
Sprat
Herring
Flat Fish
Small Pelagic Fish
Perch
Carp
Hard substrate filter feeders
Soft substrate filter feeders
Benthic deposit feeders
Polychetes

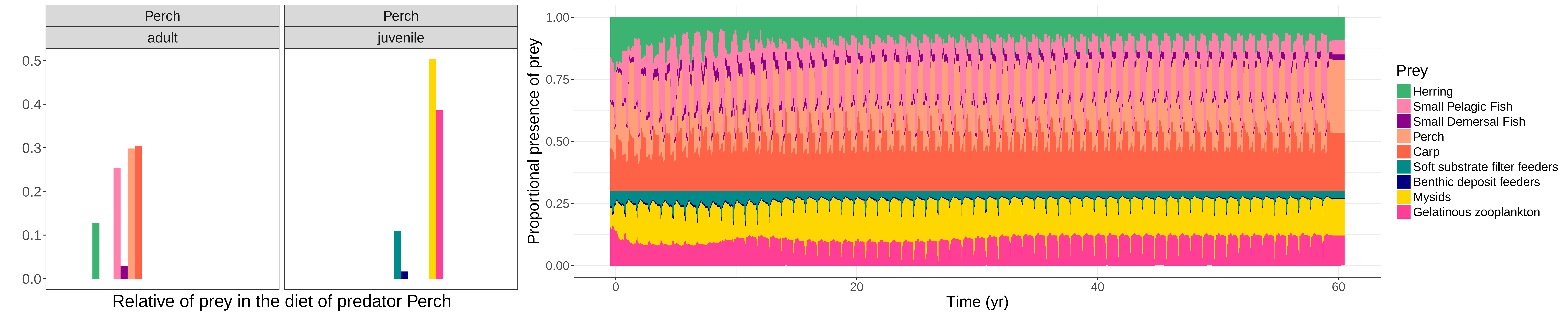
Perch
adult
Perch
juvenile
0.0
0.1
0.2
0.3
0.4
0.5
Relative of prey in the diet of predator Perch
Proportional presence of prey
0.00
0.25
0.50
0.75
1.00
0
20
40
60
Time (yr)
Prey
Herring
Small Pelagic Fish
Small Demersal Fish
Perch
Carp
Soft substrate filter feeders
Benthic deposit feeders
Mysids
Gelatinous zooplankton

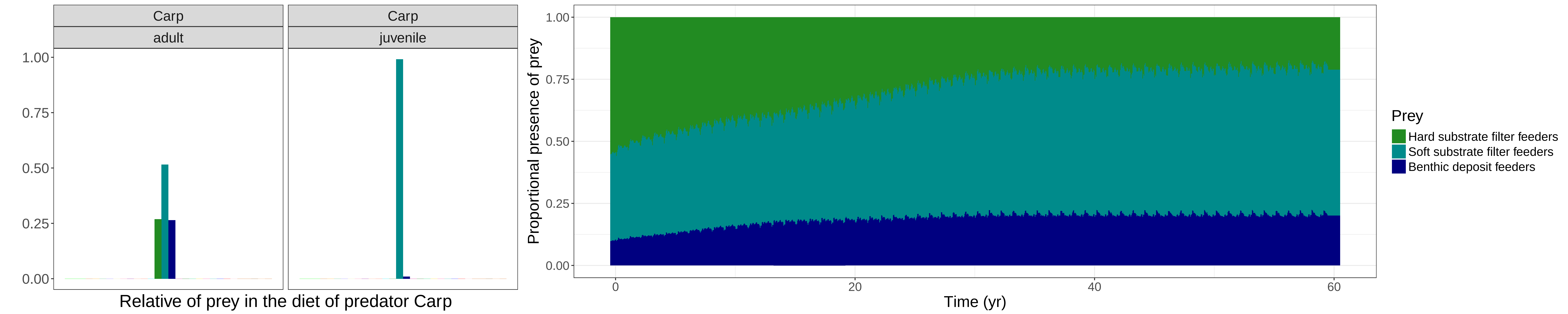
Carp
adult
Carp
juvenile
1.00
0.75
0.50
0.25
0.00
Relative of prey in the diet of predator Carp
Proportional presence of prey
0
20
40
60
Time (yr)
Prey
Hard substrate filter feeders
Soft substrate filter feeders
Benthic deposit feeders

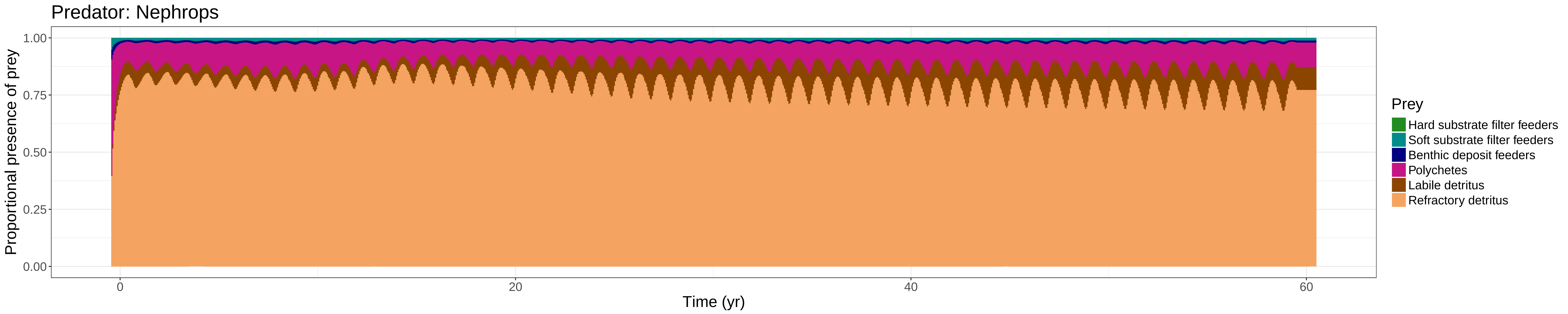

Predator: Nephrops
Proportional presence of prey
1.00
0.75
0.50
0.25
0.00
0
20
40
60
Time (yr)
Prey
Hard substrate filter feeders
Soft substrate filter feeders
Benthic deposit feeders
Polychetes
Labile detritus
Refractory detritus

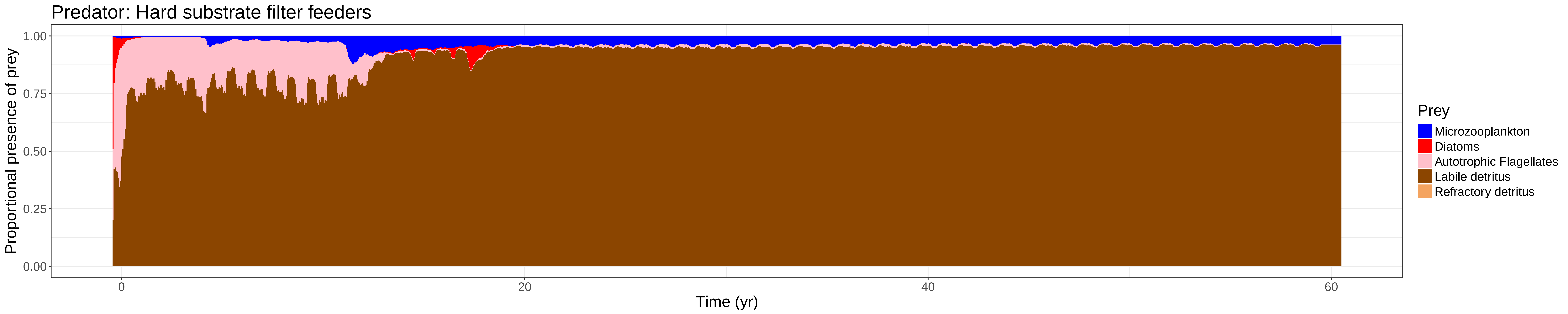
Predator: Hard substrate filter feeders
Proportional presence of prey
1.00
0.75
0.50
0.25
0.00
0
20
40
60
Time (yr)
Prey
Microzooplankton
Diatoms
Autotrophic Flagellates
Labile detritus
Refractory detritus

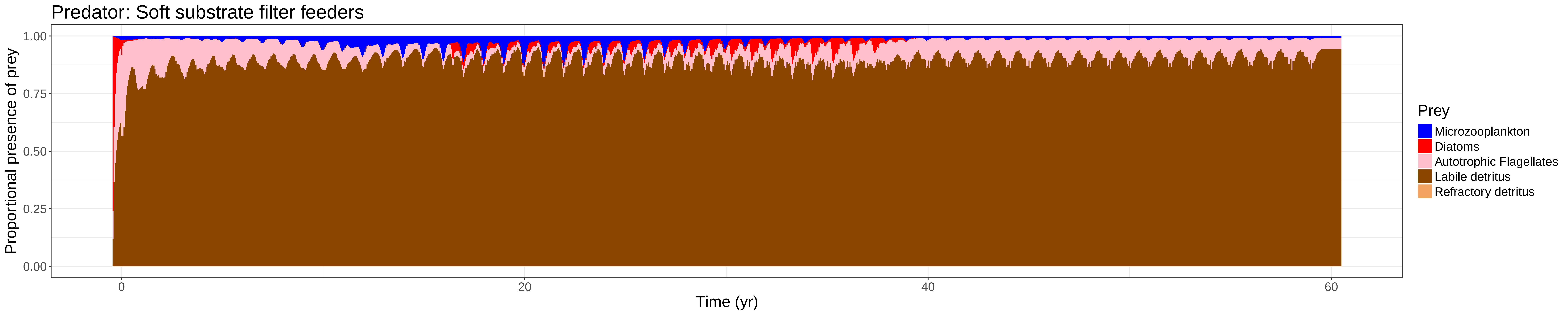
Predator: Soft substrate filter feeders
Proportional presence of prey
1.00
0.75
0.50
0.25
0.00
0
20
40
60
Time (yr)
Prey
Microzooplankton
Diatoms
Autotrophic Flagellates
Labile detritus
Refractory detritus

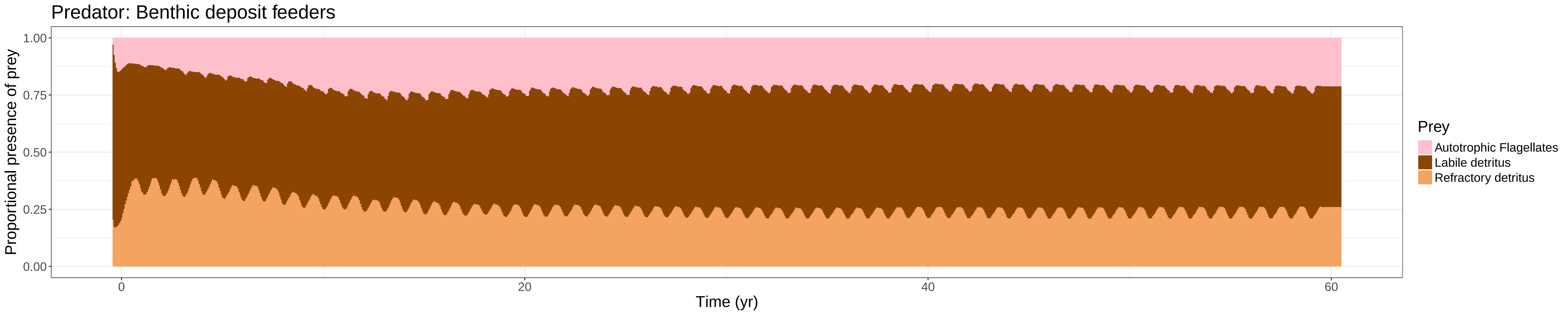

Predator: Benthic deposit feeders
Proportional presence of prey
1.00
0.75
0.50
0.25
0.00
0
20
40
60
Time (yr)
Prey
Autotrophic Flagellates
Labile detritus
Refractory detritus

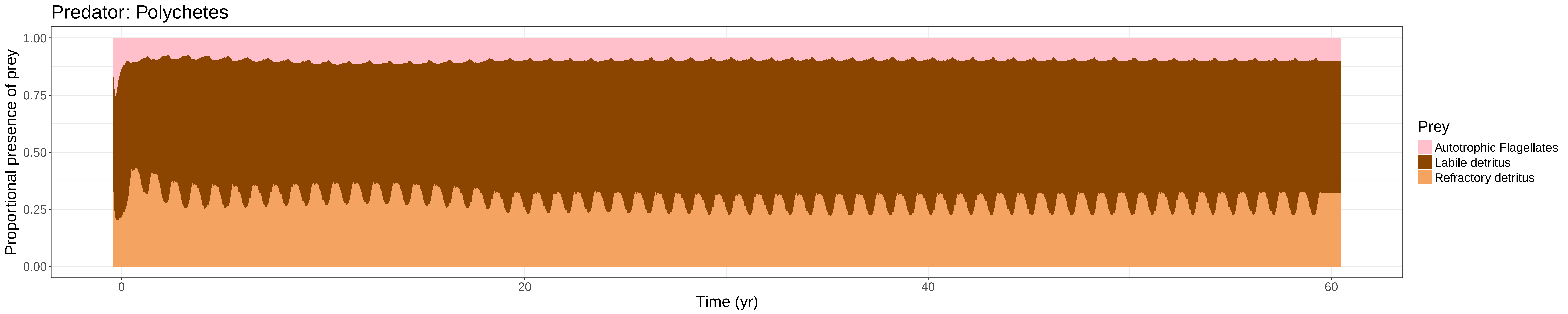

Predator: Polychetes
Proportional presence of prey
1.00
0.75
0.50
0.25
0.00
0
20
40
60
Time (yr)
Prey
Autotrophic Flagellates
Labile detritus
Refractory detritus

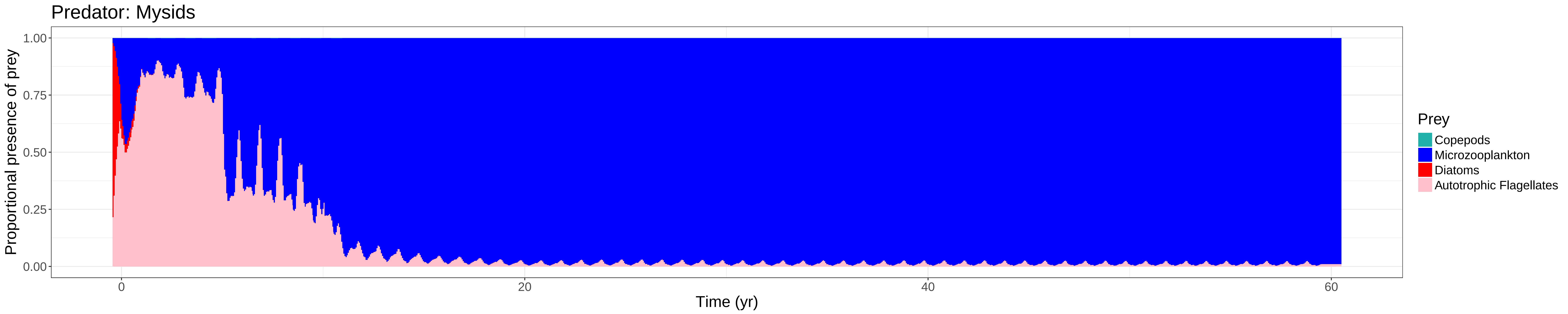

Predator: Mysids
Proportional presence of prey
1.00
0.75
0.50
0.25
0.00
0
20
40
60
Time (yr)
Prey
Copepods
Microzooplankton
Diatoms
Autotrophic Flagellates

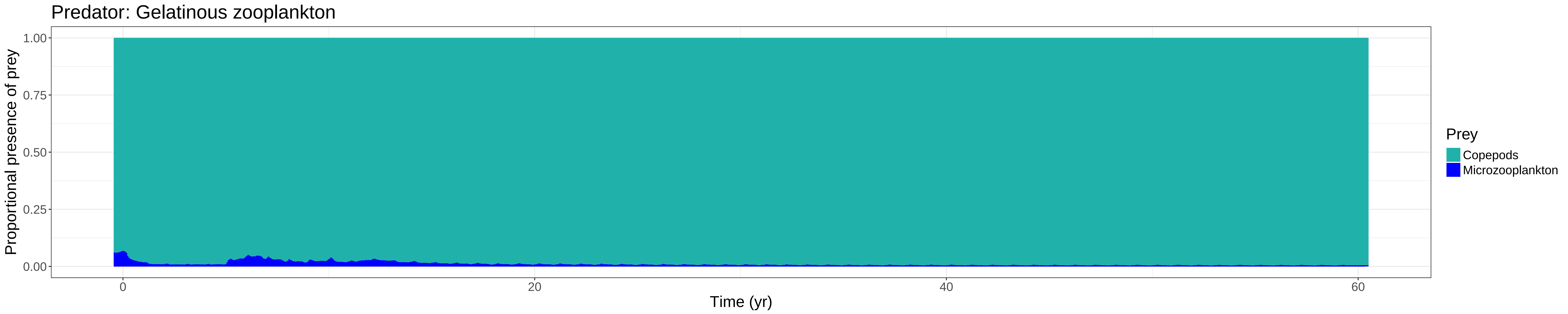
Predator: Gelatinous zooplankton
Proportional presence of prey
1.00
0.75
0.50
0.25
0.00
0
20
40
60
Time (yr)
Prey
Copepods
Microzooplankton

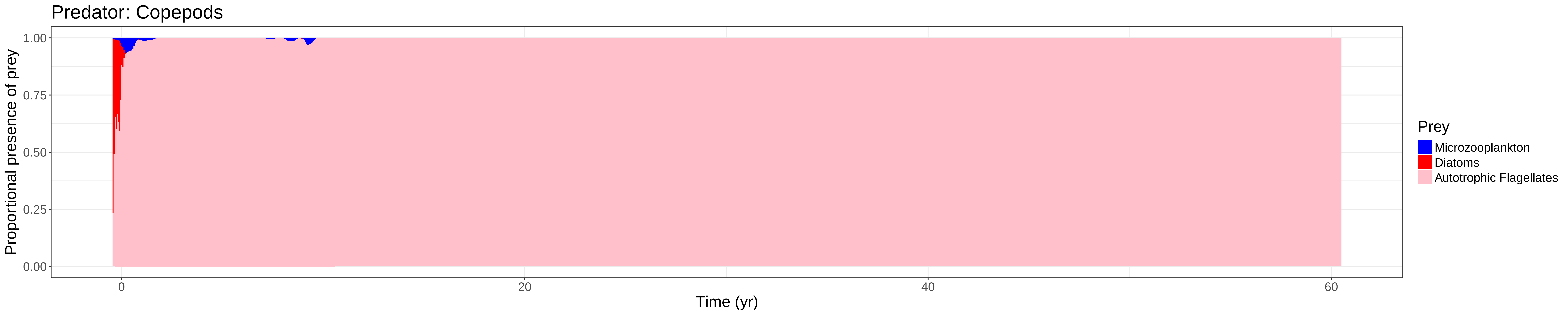

Predator: Copepods
Proportional presence of prey
1.00
0.75
0.50
0.25
0.00
0
20
40
60
Time (yr)
Prey
Microzooplankton
Diatoms
Autotrophic Flagellates

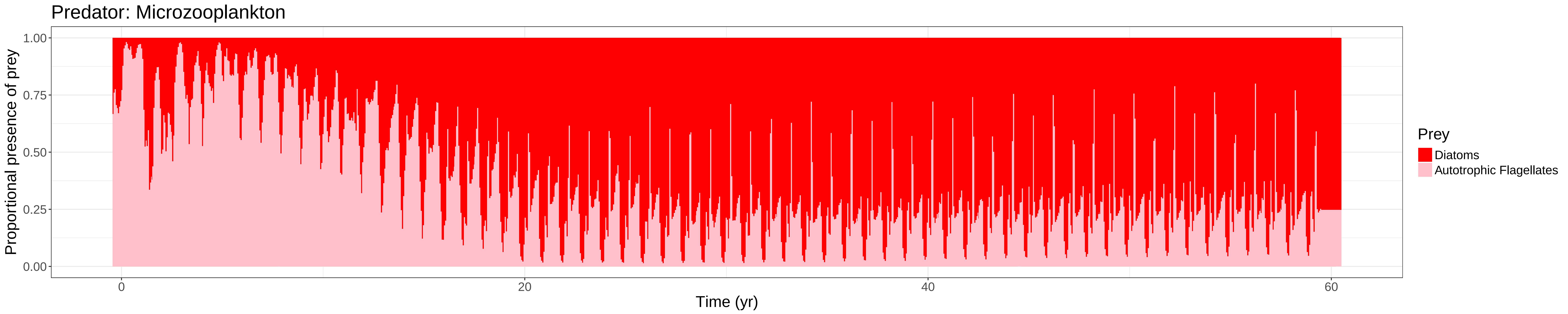
Predator: Microzooplankton
Proportional presence of prey
1.00
0.75
0.50
0.25
0.00
0
20
40
60
Time (yr)
Prey
Diatoms
Autotrophic Flagellates
