# Supplementary material for: The Baltic Sea Atlantis: An integrated end-to-end modelling framework evaluating ecosystem-wide effects of human-induced pressures
Source: PLoS One. 2018 Jul 20;13(7):e0199168. doi: 10.1371/journal.pone.0199168 (PMC6054375; doi:10.1371/journal.pone.0199168)
Supplement: S1 File — Supporting Information A. The Baltic Sea Atlantis: File A. Input data of the tracers per box for the Baltic Atlantis File B. Input data of the tracers per box and layer for the Baltic Atlantis File C. Input data for the fill values for the tracers for the Baltic Atlantis Figure A. Schematic diagram illustrating the structure of the coupled HBM-ERGOM model system Figure B. 120 year simulation run Figure C. The FISHRENT model diagram, here applied to Kattegat and Western Baltic. Figure D. One-year cycle of Chl-a in the different polygons Figure E. Relative biomass–initial condition values compared with simulation outcome Figure F. Diet composition of all predators Figure G. Biomass per age group over time for all vertebrates Figure H. Demography distribution for all vertebrates—the number of individuals for each age group Figure I. Geographical distribution of all functional groups Figure J. Geographical distribution of oxygen in the different layers. Panel 1 = top layer, panel 7 = bottom layer Figure K. Total biomass of Cod for scenario 1 (baseline) compared to scenario 5 Figure L. Relative prey biomass for predator cod, baseline compared to scenario 5 Figure M. One-year cycle of nutrients in the different polygons Table A. Physical and geochemical parameters used to internally force the Baltic Atlantis model. Table B. Summary of riverine + direct point source waterborne nitrogen loads applied to the Baltic Atlantis grid based on information from the Review of the Fifth Baltic Sea Pollution Load Compilation for the 2013 HELCOM Ministerial Meeting (HELCOM PLC-5.5). Nitrogen fractionation between DIN and DON based on Savchuk et al. (2012). Bioavailable fraction of DON assumed equal to labile DON as in Savchuk and Wolff (2009). Coastal retention fractions from Savchuk and Wolff (2009). Table C. Summary of key sources used to inform the biological module of Baltic Atlantis in relation to abundance and biomass, demography, prey-predator interaction and other functions. Ta [file pone.0199168.s001.zip › S1_File.docx]

**Supporting Information – The Baltic Sea Atlantis**

**A. HBM-ERGOM Model**

**1 HBM-ERGOM**

HBM is a further development of the BSHcmod model (Dick et al., 2001), which has been used operationally at the Danish Meteorological Institute (DMI) since 2001 (Berg et al., 2012; She et al., 2007). HBM has a two-way nested setup of the Danish Straits transition areas due to the complex bathymetry and hydrography with an inflow of high salinity bottom water into the Baltic Sea. The horizontal resolution is generally 6 nm, but 1 nm in the Danish transition area. The model has spherical coordinates horizontally and z coordinates vertically with 50 layers and barotropic time-steps of 30 seconds. The meteorolo­gical forcing is based on the operational DMI weather model HIRLAM (Sass et al., 2002).

The HBM-ERGOM model output time series applied in this context is the 2001-2009 hindcast. The initial fields for biogeochemistry were set to winter mean values (2001-2009) from 16 monitoring stations available from the International Council for the Exploration of the Sea (ICES; www.ices.dk). Climatologically conditions along the open lateral boundary of inorganic nutrients (NO3, PO4 and SiO2) and dissolved O2 were obtained from World Ocean Atlas (WOA05) (Conkright & Levitus, 2002). Monthly lateral boundary values were obtained from surface observations by the satelliteborne SeaWiFS (Sea-viewingWideField-of-viewSensor) and extrapolated with depth according to Morel and Berthon (1989). Chl-a concentrations were converted to phytoplankton nitrogen biomass using a conversion factor of 0.5 and assumed to be evenly distributed between diatoms and flagellates (from Maar et al., 2011). Cyanobacteria are not present in the North Sea due to the high salinity limiting growth and were therefore set to zero at the open boundary. They are however present in the model for the Baltic Sea. CPR data from 2004 to 2005 (Johns, 2009) was used to generate model initial fields and boundary data of bulk zooplankton biomass (Maar et al., 2012). In the current version, microzooplankton was assumed to follow the biomass of phytoplankton, but with a 5 times lower biomass. The variable detritus was set to zero. The effect of zero open boundary conditions only locally affected the model performance near the boundaries in the North Sea, but not the Baltic Sea because the importance of internal dynamics of the model increased with distance from the open boundary.

The performance of HBM-ERGOM has been evaluated extensively. In particular, (Wan et al., 2012) presented a careful assessment of this model for operational service in the Baltic Sea. The most recent version of HBM-ERGOM was validated in (Maar et al., 2016).

**Fig A.** Schematic diagram illustrating the structure of the coupled HBM-ERGOM model system

**2 HBM-ERGOM & Baltic Atlantis**

The volume exchange (currents), temperature and salinity fields and fluxes are not modeled internally by the Baltic Atlantis. Therefore, the model is forced externally with a time series of these quantities derived from HBM-ERGOM. The three variables are updated offline (i.e. using information from previously stored, completed HBM-ERGOM runs) on a 12-hourly time step basis, which is the time step used inside the Atlantis model. Due to a large difference in spatial resolution between the two models, there is significant reprocessing required to fit the HBM-ERGOM results onto the lower resolution Atlantis horizontal and vertical grid. For each box (B) and face (F) of the Atlantis native polygon grid, two types of forcing need to be provided: box averaged temperature and salinity, and face fluxes of currents. Since the Atlantis grid is not coherent with the underlying regular longitude-latitude mesh of HBM-ERGOM, data from this grid had to be recast to the Atlantis grid. Subgrid interpolation was performed by trilinear interpolation of grid data, omitting dry vertices for interpolations close to the coast line or sea bed. Atlantis box averages were evaluated by averaging data and interpolating on a regular longitude-latitude submesh with 2 km horizontal resolution, 5 vertical sub-layers and 1h temporal resolution. Fluxes were estimated by sampling cell faces with a coherent face-sub-mesh with 2 km horizontal resolution and 5 vertical sub-layers and 1h temporal resolution. Mass conservation for Atlantis cells had to be reinforced when recasting data from the HBM-ERGOM, because the sub-grid interpolation fields are not exactly rotation free. The procedure applied was to renormalize the vertical fluxes cell by cell and to remove the excess flux through the bottom layer of the dynamic boundary (for record keeping purposes these fluxes are stored in the non-dynamic boundary Box 0)

In addition to the time series hydrodynamic forcing, HBM-ERGOM provides three types of other information to the Baltic Atlantis. First, horizontal and vertical distribution of nutrients, detritus and phytoplankton from HBM-ERGOM are recalculated onto the Baltic Atlantis polygon grid to provide initial condition fields of these quantities (see section above). Second, time series of nutrient and oxygen fluxes through the boundary Box 0 are also obtained from HBM-ERGOM. This represents the inflow of North Sea water into the Baltic Sea basin. Third, many of the biological parameters, e.g. nutrient uptake, light affinity, mortality rates of functionally similar phytoplankton groups are borrowed from HBM-ERGOM in an attempt to make these components of the two models as closely compatible as possible.

**B. The Baltic Atlantis model**

The Atlantis source code is hosted in a subversion repository. To become an Atlantis user you need to register (for free) with the developer Dr. Beth Fulton ([beth.fulton@csiro.au](mailto:beth.fulton@csiro.au)). Once registered you can join the Atlantis wiki ([https://confluence.csiro.au/](https://confluence.csiro.au/login.action?os_destination=%2Fspaces%2Fviewspace.action%3Fkey%3DAtlantis&permissionViolation=true)) where you’ll get access to the SVN repository. The input data specific to the Baltic Atlantis is given in the three excel documents within the Supporting Information material (File A - C), other specific parameters are given in the tables below.

**File A**. Input data of the tracers per box for the Baltic Atlantis

**File B**. Input data of the tracers per box and layer for the Baltic Atlantis

**File C**. Input data for the fill values for the tracers for the Baltic Atlantis

**1. Forcing**

*Table A: Physical and geochemical parameters used to internally force the Baltic Atlantis model.*

| **Parameter name** | **Value** | **Unit** | **Source** |
| --- | --- | --- | --- |
| Atmospheric deposition of nitrogen (NH4) | 0.42 | mg-N m-2 d-1 | HBM-ERGOM |
| Redfield ratio of O:N | 16 | mg-O2 (mg-N)-1 | Atlantis standard setting |
| Redfield ratio of C:N | 5.7 | mg-C (mg-N)-1 | Atlantis standard setting |
| Redfield ratio of Chl-*a*:N | 7 | mg-Chl-*a* (mg-N)-1 | Atlantis standard setting |
| Coefficient of background light absorption | 0.03 | m-1 | HBM-ERGOM |
| Coefficient of light absorption due to particulate matter | 0.0035 | m2 mg-N-1 | HBM-ERGOM |
| Coefficient of light absorption due to dissolved organic nitrogen (DON) | 0.0009 | m2 mg-N-1 | HBM-ERGOM |
| Coefficient of light absorption due to labile detritus | 0.0038 | m2 mg-N-1 | HBM-ERGOM |
| Basic coefficient of light absorption | 0.05 | - | HBM-ERGOM |
| Diffusion coefficient for bio-irrigation of sediments at water-sediment interface, scaled by the amount of biological activity in the sediment | 1e-7 | m2 s-1 per animal per m2 | HBM-ERGOM |
| Exchange rate for bio-irrigation of sediments at water-sediment interface, scaled by the amount of biological activity in the sediment | 1e-6 | m s-1 per animal per m2 | HBM-ERGOM |
| Diffusion coefficient for bio-turbation of sediments at water-sediment interface, scaled by the amount of biological activity in the sediment | 1e-8 | m2 s-1 per animal per m2 | HBM-ERGOM |
| Exchange rate for bio-turbation of sediments at water-sediment interface, scaled by the amount of biological activity in the sediment | 1e-8 | m s-1 per animal per m2 | Atlantis standard setting |
| Decay rate of labile detritus (to a sink, not accessible by the model) | 1e-10 | d-1 | Described in calibration |
| Decay rate of refractory detritus (to a sink, not accessible by the model) | 1e-10 | d-1 | Described in calibration |
|  |  |  |  |

Rates of nitrogen external loads used to force Baltic Atlantis are summarized in Table B.

*Table B: Summary of riverine + direct point source waterborne nitrogen loads applied to the Baltic Atlantis grid based on information from the Review of the Fifth Baltic Sea Pollution Load Compilation for the 2013 HELCOM Ministerial Meeting (HELCOM PLC-5.5). Nitrogen fractionation between DIN and DON based on Savchuk et al. (2012). Bioavailable fraction of DON assumed equal to labile DON as in Savchuk and Wolff (2009). Coastal retention fractions from Savchuk and Wolff (2009).*

| **Box #** | **2005 Total N point source (tonnes/year)** | **Fraction DIN (NH3+NO3)** | **Fraction DON** | **Fraction bioavailable DIN** | **Fraction bioavailable DON** | **Fraction coastal retention (bioavail DIN + bioavail DON)** | **Fraction transported & box # receiving** | **2005 supply rate as NO3** | **2005 supply rate as DON** |
| --- | --- | --- | --- | --- | --- | --- | --- | --- | --- |
| 0 |  |  |  |  |  |  |  |  |  |
| 1 | 5340.56 | 0.76 | 0.24 | 1 | 0.45 | 0.08 |  | 118408.14 | 16826.42 |
| 2 | 9435.64 | 0.76 | 0.24 | 1 | 0.45 | 0.09 |  | 206928.13 | 29405.58 |
| 3 | 26744.95 | 0.76 | 0.24 | 1 | 0.45 | 0.09 |  | 586529.91 | 83348.99 |
| 4 | 36915.50 | 0.76 | 0.24 | 1 | 0.45 | 0.08 |  | 818471.51 | 116309.11 |
| 5 | 1497.27 | 0.76 | 0.24 | 1 | 0.45 | 0.1 |  | 73628.52 | 16960.92 |
| 6 |  |  |  |  |  |  | 0.3-12 | 96024.95 | 28807.48 |
| 7 | 58908.89 | 0.5 | 0.5 | 1 | 0.2 | 0 | 0.3-26 | 653796.06 | 130759.21 |
| 8 | 74661.83 | 0.5 | 0.5 | 1 | 0.35 | 0 |  | 1183755.52 | 414314.43 |
| 9 | 111613.77 | 0.5 | 0.5 | 1 | 0.25 | 0.1 |  | 1592662.29 | 398165.57 |
| 10 |  |  |  |  |  |  |  |  |  |
| 11 |  |  |  |  |  |  | 0.3-20 | 110452.50 | 33135.75 |
| 12 | 15322.14 | 0.5 | 0.5 | 1 | 0.3 | 0.1 | 0.15-6 | 185842.16 | 55752.65 |
| 13 | 1094.44 | 0.5 | 0.5 | 1 | 0.3 | 0.1 |  | 15616.99 | 4685.10 |
| 14 | 43746.99 | 0.76 | 0.24 | 1 | 0.45 | 0.1 | 0.1-16 | 853965.12 | 121352.94 |
| 15 | 5472.19 | 0.5 | 0.5 | 1 | 0.3 | 0.1 |  | 78084.94 | 23425.48 |
| 16 | 73151.48 | 0.5 | 0.5 | 1 | 0.3 | 0.1 | 0.1-6 0.25-18 | 773373.37 | 217030.17 |
| 17 | 103774.49 | 0.5 | 0.5 | 1 | 0.3 | 0.1 | 0.25-18 | 1110600.23 | 333180.07 |
| 18 |  |  |  |  |  |  | 0.25-11 | 473367.85 | 142010.36 |
| 19 | 1048.89 | 0.5 | 0.5 | 1 | 0.3 | 0.1 |  | 14966.98 | 4490.09 |
| 20 |  |  |  |  |  |  |  | 47336.79 | 14201.04 |
| 21 | 6995.00 | 0.5 | 0.5 | 1 | 0.25 | 0.04 |  | 106468.80 | 26617.20 |
| 22 |  |  |  |  |  |  |  |  |  |
| 23 |  |  |  |  |  |  |  |  |  |
| 24 | 28334.66 | 0.5 | 0.5 | 1 | 0.25 | 0.04 |  | 431273.44 | 107818.36 |
| 25 | 20023.80 | 0.5 | 0.5 | 1 | 0.25 | 0.04 |  | 304776.26 | 76194.06 |
| 26 |  |  |  |  |  |  |  | 280198.31 | 56039.66 |
| 27 |  |  |  |  |  |  |  |  |  |
| 28 |  |  |  |  |  |  |  |  |  |

Denitrification is the only explicit biogeochemical nutrient sink in the Baltic Atlantis model. The parameterization of this process strongly relies on the successful calibration of nutrient and oxygen profiles. In Atlantis, denitrification is a function of bacterial activity which is being modeled explicitly. This is an important difference compared to the ERGOM model for instance. The calibration process of Atlantis confirms the expected difficulties in obtaining an accurate simulation of denitrification. Unless one is using a very high spatial resolution, coupled physical-biological models in the Baltic are known to struggle to capture the strong vertical oxygen gradient which results in lower denitrification and a general nutrient build-up in the model. This observation is confirmed by labile detritus exhibiting a continuous increase in time in response to a very high external source term (riverine inflows) and a very low sink term. In order to correct for the excess detritus build-up in Baltic Atlantis, we introduced a detritus decay rate in the model – a proxy for sediment burial and other potential unaccounted sink terms of detritus in the Baltic. Burial rates published in Deutsch et al. (2010) show a vast potential range that is difficult to constrain due to so many uncertainties, for example related to the unknown magnitude of the buffer effect of nutrients in the sediments in the deep Baltic basins. We adopted rates at an order of 1E-10 per day for both labile and refractory detritus, which are within the published range (Deutsch et al., 2010) and which enable total detritus in the Baltic Sea to reach a quasi-equilibrium.

Despite the fact that the Baltic Atlantis is forced with outputs from the high resolution HBM-ERGOM model system, the model framework may, to some extent, be limited in its ability to fully represent bio-geo-chemical and primary production responses to changes in eutrophication pressures with very high resolution. On one hand, the separation of narrow coastal from larger offshore polygons in the Baltic Atlantis has the potential to resolve processes at a higher spatial resolution than other box ecosystem models such as BALTSEM (Savchuk et al., 2012). This enables Atlantis to better account for strong gradients in bathymetry, salinity, nutrients and biotic habitat distribution as well as better coupling between hydrographical and biological processes. On the other hand, fields and fluxes derived from HBM-ERGOM, together with riverine and point sources of nutrients, when translated into the Atlantis polygon grid represent only box averages which have spatial limitations in simulating the full patchiness and coastal gradients in nutrients and primary production.

**2. Initial conditions**

While reliable accounts of the spatial distribution patterns of all vertebrate groups are found up to year 2012, there is no coincident coverage of published distribution data on the majority of invertebrate groups. A comprehensive review of the contents of publicly available databases, reports and publications reveals 2005 as the most data rich year and, thus, the optimum reference year for the model information process. The decision to parameterize the model to a narrow time period is a conscious one, albeit in contrast to the long-term time period average approach applied in many previous end-2-end model set ups. The Baltic Sea ecosystem undergoes frequent and significant regime shifts during which large structural and functional ecosystem changes are detected both in the physical-chemical environment and in the biological environments and communities. One might argue that a longer than 10 year time period average of abundance/biomass distributions would, thus, produce a desirable mean representation of the ecosystem. However, there is also in this respect the danger that the rendered patterns, especially of vertebrate distributions, could not in such a case be interpreted in a physically meaningful way, thus extending the long model spin-up time even further. This is because the state of a given group would not necessarily be related to a particular pressure or state of another functional group that is a key prey or predator for a given group. Based on expert knowledge of the integrated ecosystem state in 2005 (e.g.: ICES WGIAM), that year is also described as quite typical year for the Baltic Sea in the recent period, that is without any significant anomalies in the physical or biological states.

Quarterly abundance distribution of vertebrates

Quarterly abundance/biomass distribution patterns from 2005 are used to inform the Baltic Atlantis model in two ways. Firstly, absolute numbers per age group from the first quarter or semester are used to determine the initial conditions for the model simulations. Secondly, the relative distribution patterns of juvenile and adult biomass are used to drive the forced seasonal migration. The latter allows for the redistribution of the current juvenile and adult biomass for each season according to a set fraction for each of the polygons, in order to mimic the movement of the vertebrates during model calibration. Spatial distribution plots will therefore often show much stronger seasonal fluctuations compared to the aggregated biomass time series. This is because the oscillations represent the migration between spawning and feeding grounds, on top of their natural biomass oscillations. The prescribed seasonal migration within the model domain is eventually replaced with density and forage-dependent movements, where the different functional groups will move according to physical conditions and/or seasonal changes in forage fields.

Quarterly estimates of the numbers at age of MHP (harbour porpoise), SEA (seals) and SBD (persuit-diving seabirds) groups in Baltic Atlantis polygons come from long-term annual monitoring programs performed by various institutions across the Baltic countries. Regional MHP abundance estimates are converted into polygon abundances through a relative surface area scaling. Numbers of SBD breeding pairs come from the HELCOM database and additional publications. Abundances reported from coastal sites by country are translated onto the Atlantis polygon grid by comparing lengths of national coastlines with lengths of polygon borders within country's borders. The majority of the numbers of SEA pups and adults in the Western and Northern Baltic come from the Swedish Meteorological and Hydrological Institute (SMHI). Details of the sources of raw population numbers are listed in Table C.

The primary sources for calculating fish abundance and/or fish biomass at age by polygon were: ICES IBAM acoustic surveys, ICES BITS trawl surveys, ICES stock assessments (www.ices.dk), national coastal fish surveys from Baltic coastal countries, and HELCOM coastal fish surveys, and from other models (e.g. EwE). While acoustic surveys give estimates of absolute numbers of fish on a spatially explicit scale, the BITS and coastal fish surveys only give estimates of relative density and distribution patterns on a spatially explicit scale, which then have to be applied to absolute stock abundances/biomasses using information from stock assessments or other sources if available, or using very gross assumptions. In general for the ICES assessed fish stocks and species (cod, herring, sprat, some flatfish) the abundances/biomasses have been obtained from the assessments (for 2005), and subsequently the abundances/biomasses have been spatially re-distributed based on the survey estimates. Abundance/biomass data available per ICES area was translated onto the Atlantis polygon grid. This is done using a precise area correction based on overlap of surface areas calculated in GIS. For demersal species, BITS Catch per Unit Effort (CPUE), expressed in numbers per trawl effort time, are applied to stock assessment population numbers/biomass, or in absence hereof converted into abundance estimates by using gear-specific swept areas based on wing spread. The procedures adopted to calculate distributions for each vertebrate group are briefly specified below. An example is the perch and roach distribution, derived from fish survey abundance level categories from catch indices from HELCOM (<http://bio.helcom.fi/apex/f?p=108:11>, <http://bio.helcom.fi/apex/f?p=108:8>, HELCOM 2017a, HELCOM 2017b).

*Table C: Summary of key sources used to inform the biological module of Baltic Atlantis in relation to abundance and biomass, demography, prey-predator interaction and other functions.*

| **Parameters / Group code** | **Abundance/biomass/concentration/flux** | **Demography, reproduction, mortality** | **Dietary interactions** |
| --- | --- | --- | --- |
| MHP | Hammond et al. (2013) | Koschinski (2001) | Andreasen (pers. comm.) |
| SEA | SMHI; Harkonen et al. (2007); Vanhatalo et al. (2014) and sources therein; Ahola (pers. comm.) | Kauhala et al. (2012) | Lundström (2010); Tomczak et al. (2009) and sources therein |
| SBD | HELCOM; Hentati-Sundberg (2011) | Törnlund (2013) | Törnlund (2013); Tomczak et al. (2009) and sources therein |
| FCD | ICES Assesssments; ICES BITS surveys | ICES; Bastardie et al. (2014) | Tomczak et al. (2012) and sources therein; SMS |
| FHR | ICES Acoustic surveys; ICES Assessments | Acoustic surveys; ICES; Bastardie et al. (2014); SMS | Tomczak et al. (2012) and sources therein; Möllmann et al. (2004) |
| FSR | ICES Acoustic surveys; ICES Assessments | Acoustic surveys; ICES; Bastardie et al. (2014); SMS | Tomczak et al. (2012) and sources therein; Möllmann et al. (2004) |
| FFL | ICES Assessments | ICES; Bastardie et al. (2014) | Tomczak et al. (2012) and sources therein; |
| FWH | ICES BITS surveys | Bastardie et al. (2014) | Lappalainen et al. (2001) |
| FCP | Coastal fish surveys; HELCOM | FISHBASE | Tomczak et al. (2009) and sources therein; |
| FPR | Coastal fish surveys; HELCOM | FISHBASE | Tomczak et al. (2009) and sources therein; Lappalainen et al. (2001) |
| FSD | HELCOM | FISHBASE | Bubinas and Ložys (2000); Ehrenberg et al. (2005) |
| FSP | ICES BITS surveys | FISHBASE | Peltonen et al. (2004) |
| NE | ICES Assessments | ICES | Johnson et al. (2013) |

MHP (harbour porpoise), SEA (seals), SBD (pursuit-diving seabirds), FCD (cod), FSR (sprat), FHR (herring), FFL (flat fish), FWH (whiting), FCP (cyprinids), FPR (perch), FSD (small demersals), FSP (small pelagics), NE (Nephrops)

For the FHR and FSR groups, absolute abundances in numbers at age by polygon based on the area corrected acoustic survey abundance data are calculated. For FCD, population number by age by polygon is calculated by distributing the summed number by age for all 3 ICES cod assessments in the Baltic area (Cod SD21, Cod SD22-24, Cod SD25-32 in 2005 extracted from the 2013 assessment) according to the BITS survey relative spatial distributions. Consequently, the summed biomasses are redistributed geographically according to survey information on relative catch rate indices, i.e. biomasses are multiplied by the relative CPUE by age by polygon (relative to total CPUE by age for all polygons) from the BITS survey data. Assessment data are extracted from the ICES WGBFAS based on the XSA, SAM and SMS model assessments. Swept area estimates and CPUE by polygon by age from BITS (for 2005) are used to calculate FFL numbers by area by polygon. Because the key species in this group, flounder, is known to be distributed beyond the northern limit of the BITS survey, additional population number estimates from the WGBFAS reports are used to cover those areas. Distributions of FWH are obtained solely from the BITS survey data, converted to numbers per polygon using swept area information.

Abundance of coastal freshwater fish in FCP and FPR groups require a more complicated calculation procedure involving much higher uncertainty because these groups are not detected representatively in the standard ICES surveys and their distribution areas are not covered in those surveys. Due to a lack of knowledge of the swept area in the coastal fish survey catch values (often non-standardized trap or gillnet surveys with only very limited data registration and with no effort estimate involved), the catch indices reported in HELCOM are for those groups scaled with independent but geographically approximate fish biomass density measurements. Assuming an average weight of an adult individual, biomass per unit area is first converted into density estimates. Dividing the catch index by the density estimate, a scaling factor is then obtained and applied uniformly to all available catch indices from all coastal fish surveys. Application of a uniform scaling factor (given effort and fishing method is very different) is a big assumption which carries along a large uncertainty on the total fish abundance estimates. While it is impossible to state whether these calculations are over- or underestimates of the true population, or even within an correct order of magnitude estimate in agreement with the actual population numbers in the Baltic, it is still important to include the group as this severely understudied and yet ecologically is anecdotally known to be a very significant ecosystem component. An order of magnitude agreement is found for this group in relation to an independent regional biomass calculation performed in the ECOPATH models, providing a first order validation of the basin-wide calculations made for the Baltic Atlantis implementation. The calculations were limited to coastal polygons and none of the FCP and FPR fish were allowed to distribute beyond their reported salinity tolerance range.

Even more uncertain are the initial estimates of the FSD and FSP groups. Small demersals comprising mostly gobies, are assigned to an average density of 2 specimens per m2 for the majority of coastal polygons where species have been observed both in the HELCOM surveys and in the BITS surveys, and to a density of 0.2 individuals per square meter in the more offshore polygons where species have been observed in the BITS surveys. Highest abundances of FSD are reported in the area around the Lolland island (polygon 4) and in the south-eastern Baltic coastal waters (polygon 17). Sticklebacks from the FSP group are known to occur with high abundance in offshore waters but migrate inshore during spawning season. Only a few site-specific estimates of their abundance are available in the Baltic and scaling the population densities based on these accounts is highly uncertain. As a consequence, the initial conditions specified for numbers at age for FSD and FSP are poor reference points. Only an order of magnitude agreement is expected from the calibrated model outputs.

Biomass distribution patterns of invertebrates

Biomass distribution patterns of all benthic invertebrate groups were obtained from a compilation of many sources of data. The most comprehensive and consistent in time and space samplings were recorded in: the Swedish Meterological and Hydrological Institute database (SMHI; http://www.smhi.se/en), Danish National Environmental Research Institute (DMU) National Database for Marine Data (MADS; http://www.dmu.dk/vand/havmiljoe/mads/), Finnish Environmental Institute (SYKE) national Hertta database (wwwp2.ymparisto.fi/scripts/oiva.asp) and the ICES database (http://ecosystemdata.ices.dk/).

The majority of measurements were performed in units of abundance that needed to be converted into units of biomass. This task is achieved by applying an average weight per individual conversion rate which in most cases was available in the literature on a per species basis, but which sometimes has to be inferred from the dataset itself (provided that at least one coincident biomass estimate was available). All data points from the relevant time period are plotted on top of the Atlantis polygon grid in QGIS. A spatial average is calculated per polygon. In the frequent case that no estimates are available for a given polygon, and that polygon is not outside the known range of distribution for that group, a value from the ecologically most similar polygon is copied onto the empty polygon.

Abundance/biomass model input files

Complete information on initial abundance and/or biomass of all biological groups can be found in the three spreadsheet files attached to Supporting Information:

1. File A
   1. Biomass of benthic invertebrate groups per box in mg-N/m2 written in as: [Group Long Name]_N
   2. Percentage cover per box of biotic habitats (i.e. occupied by benthic biological groups)
   3. Fraction of box surface area covered with an abiotic habitat (see SI 2.2 for the list of habitats)
   4. Other parameters given per box: number of wet depth layers (numlayers), depth of sediment penetration by biota (sedbiodepth), depth of sediment penetration by detritus (seddetdepth), depth of sediment oxygen profile (sedoxdepth), maximum density of biotic groups in the sediment (setbiodens), sediment irrigation enhancement rate (sedirrgenh), sediment bioturbation enhancement rate (sedturbenh), erosion rate (erosion_rate), coefficient of eddy mixing (eddy).
2. File B
   1. Vertebrate group abundance in numbers of individuals: [Group Long Name][Age Class]_Nums
   2. Vertebrate group structural/reserve biomass per individual per box per layer in mg-N: [Group Long Name][Age Class]_StructN/ResN
   3. Total vertebrate group population biomass per box per layer in mg-N/m-3: [Group Long Name]_N[Age Class]
   4. Total pelagic and epibenthic invertebrate group population biomass per box per layer in mg-N/m3: [Group Long Name]_N[Age Class]
3. File C – Values used to fill all the parameters listed in the two files above whenever no initial conditions value was specified.

Physiological and life-history traits

Length-weight parameters, maximum potential growth rate, and length at infinity are the four key parameters taken from ICES data or model compilations, or from FISHBASE, to estimate age-specific weight, growth rate, and clearance rates assuming scaling laws from metabolic theory of ecology. For fish and other HTL groups, the maximum growth rate (Gmax) is derived by utilizing the weight-consumption relationship from fish bioenergetics (Hanson 1997).

Gmax = CA × Weight ^ CB

Weight estimates from von Bertalanffy curves are used to obtain maximum consumption for an average individual. The constants are generalized across functional groups, setting CA equal to 0.3 and CB equal to 0.7. Growth efficiency is assumed to be 10% (Pauly & Christensen 1995).

Consumption rates from this equation represent daily averages, and assuming that individuals generally operate at about 30% of their potential maximum, the resulting Gmax is multiplied by three to obtain theoretical maxima. Similar scaling arguments are assumed to derive clearance rates for all age-structured vertebrates.

The majority of maximum growth and clearance rates for pelagic invertebrates are taken from a recent data compilation performed by Kiørboe and Hirst (2014) who provide the largest contribution to the model parameterization in this context. In the case of benthic invertebrates, rates calibrated for a previous benthic model in the Baltic are used (Timmermann et al. 2012). Nutrient and light acquisition half-saturation constants, maximum growth rates mortalities terms for all phytoplankton groups are taken from the ERGOM model (Maar et al. 2011).

It should be noted that during calibration these rates were altered significantly, especially for vertebrates, sometimes by more than an order of magnitude. In the end, while the distribution of relative growth and consumption rates at age was maintained, the absolute values were adjusted freely in order to optimize the emerging structural and reserves weight of individual vertebrates on which there are well-defined observational constraints available (e.g. Figs. 5 and 7).

Demographic profiles, mortality rates & reproduction functions

Although Atlantis is not an agent-based model because it does not model true individuals, it still enables us to simulate temporally dynamic age groups, with dynamic size-age relationships, where all individuals are identical for a certain age group and polygon cell at a given time. For mammals and seabirds literature-based survival rates are used to constrain natural and predator mortalities and to estimate the initial demographic structures of the populations (sources listed in Table E). It should be noted that, in reality, there are very high inter-annual differences in mortality rates, which are also quite site specific. In order to have a density dependent reproduction, the reproduction of these groups was also modelled using the Beverton-Holt function instead of the constant reproductive rate of pups/calves/hatchings.

The majority of the data on the 2005 initial population structure of fish groups comes from the ICES stock assessments. The SMS multi-species assessment model provides reliable estimates of natural, total predation and fishing mortality rates for the FCD, FSR and FHR groups. Other fish groups which are not assessed require a theoretical approach to mortality and demographic structure estimation. The calculations are based on species-specific von Bertalanffy growth parameters and stock-recruitment relationship parameter values compiled from other models or found in FISHBASE. For an overview of main sources and references used to collect these data see Table C.

There are two types of reproduction functions currently used in the Baltic Atlantis model. For invertebrates, which are all single biomass pool groups, there is the simple growth rate which determines their division rate per day. For marine mammals, seabirds and fish groups recruitment processes are described in Baltic Atlantis using a standard Beverton-Holt function with parameters calibrated during this study. There is, on top of that, an effect of environmental change considered in the vertebrate recruitment, which scales the recruitment according to the environmental factors.

The effect of temperature on recruitment is firstly scaled with the parameter, which follows a simple q10 principle (Audzijonyte et al., 2017). Secondly the model checks whether the temperature and salinity conditions in the cell are within the minimum and maximum spawn temperature and salinity ranges (Table D). If not, the scalar will accordingly be set to zero and there won’t be any recruitment in that cell. On top of the temperature and salinity dependencies, recruitment is also sensitive to the oxygen level. Recruits will die if they arrive into cells with oxygen concentrations lower than the set minimum.

Besides recruitment, oxygen levels also affect the distribution of the species, their feeding rate and a stress related linear mortality. Two types of limitations are active, the ambient oxygen limitation and the depth based limitation. The first one uses the ambient oxygen levels (), the lethal oxygen concentration () and the limiting oxygen concentration () to calculate the oxygen scalar (Audzijonyte et al., 2017):

The scalar is then applied to the oxygen mortality , with an increased linear mortality when oxygen concentration levels are below the minimum oxygen level. Species that are able to move however, will contract to areas above the minimum oxygen level. Although if no suitable habitat is found (i.e. if the entire area was to become anoxic) they would be completely lost from the model.

The second one, the depth based limitation, calculates the oxygen scalar based on the sediment depth of the half oxygen mortality parameter and the depth of the oxygenated sediment layer (Audzijonyte et al., 2017):

The depth based oxygen limitation is only applied to the clearance rate as it will affect the feeding rate for the epibenthic groups that dig into the sediment.

Tables D and E provide a summary of key biological parameters used for vertebrates in the Baltic Atlantis model, and Table F for invertebrates.

*Table D: Summary of key biological parameters used for vertebrates in the Baltic Atlantis model.*

| **Code** | **Linf [cm]** | **k** | **a** | **b** | **Age Class First Mature** | **Age Max [yrs]** | **Age+ Class** | **BHa** | **BHb** |
| --- | --- | --- | --- | --- | --- | --- | --- | --- | --- |
| MHP | 150 | 0.95 | 0.081 | 2.67 | 3 | 24 | 10 | 3.0E+5 | 3.0E+08 |
| SEA | 220 | 0.4 | 0.001 | 3.63 | 3 | 25 | 10 | 5.0E+4 | 3.0E+08 |
| SBD | 87 | 0.63 | 0.0345 | 3 | 3 | 20 | 10 | 10.0E+8 | 3.0E+08 |
| FCD | 112,5 | 0.1 | 0.0099 | 2.965 | 4 | 25 | 10 | 8.0E+9 | 3.0E+08 |
| FSR | 13.6 | 0.6 | 0.0041 | 3.22 | 3 | 10 | 10 | 11.2E+11 | 3.0E+08 |
| FHR | 20 | 0.34 | 0.0062 | 3.019 | 4 | 25 | 10 | 9.1E+11 | 3.0E+08 |
| FFL | 37 | 0.35 | 0.0079 | 3.089 | 4 | 16 | 10 | 1.0E+9 | 3.0E+08 |
| FWH | 37.8 | 0.458 | 0.0089 | 2.926 | 3 | 20 | 10 | 4.0E+8 | 3.0E+08 |
| FSP | 6.7 | 0.6 | 0.0068 | 3.28 | 2 | 8 | 5 | 141.6E+11 | 3.0E+08 |
| FSD | 13.3 | 0.4 | 0.0174 | 2.96 | 3 | 6 | 5 | 9.0E+11 | 3.0E+08 |
| FPR | 31.1 | 0.28 | 0.011 | 3.11 | 3 | 22 | 10 | 9.97E+10 | 3.0E+08 |
| FCP | 35.5 | 0.2 | 0.0074 | 3.21 | 2 | 15 | 10 | 2.0E+11 | 3.0E+08 |

*Continue Table D*

| **Code** | **Minimum spawning temperature [°C]** | **Maximum spawning temperature [°C]** | **Minimum spawning salinity [psu]** | **Maximum spawning salinity [psu]** | **Minimum tolerated oxygen [mg-O2]** |
| --- | --- | --- | --- | --- | --- |
| MHP | NA | NA | NA | NA | 0 |
| SEA | NA | NA | NA | NA | 0 |
| SBD | 2 | 25 | 0 | 40 | 32 |
| FCD | 0.5 | 8.5 | 6 | 33 | 0 |
| FSR | 2 | 25 | 0 | 40 | 0 |
| FHR | 2 | 25 | 0 | 10 | 0 |
| FFL | 3 | 25 | 0 | 40 | 0 |
| FWH | 2 | 25 | 0 | 40 | 0 |
| FSP | 2 | 25 | 0 | 10 | 0 |
| FSD | 2 | 25 | 0 | 40 | 0 |
| FPR | 2 | 25 | 0 | 10 | 0 |
| FCP | 2 | 25 | 0 | 10 | 0 |

*Table E: Maximum potential growth rates and clearance rates per age class of all vertebrate biological groups.*

| **Code** | **Growth (Gmax)**  **[mg N d-1] & clearance C [mg3(mg N)-1 d-1]** | | **Age Class** | | | | | | | | | | | | | | | | |
| --- | --- | --- | --- | --- | --- | --- | --- | --- | --- | --- | --- | --- | --- | --- | --- | --- | --- | --- | --- |
|  |  |  | **1** | **2** | **3** | | **4** | **5** | | **6** | | **7** | | **8** | | **9** | | **10** | |
| MHP | Gmax | 6204 | | 3204 | | 4300 | 6900 | | 8900 | | 9900 | | 15900 | | 21900 | | 21900 | | 21900 |
|  | C | 4360 | | 4480 | | 4480 | 5480 | | 5480 | | 5480 | | 5480 | | 5480 | | 5480 | | 5480 |
| SEA | Gmax | 10500 | | 8900 | | 9900 | 9900 | | 10000 | | 11900 | | 12900 | | 18900 | | 25900 | | 25900 |
|  | C | 4000 | | 4480 | | 4480 | 4480 | | 5060 | | 5060 | | 5060 | | 5060 | | 5060 | | 5060 |
| SBD | Gmax | 180 | | 420 | | 520 | 1020 | | 1120 | | 2020 | | 2220 | | 2220 | | 3020 | | 3020 |
|  | C | 240 | | 560 | | 1160 | 1460 | | 1660 | | 1660 | | 1660 | | 2060 | | 2060 | | 2060 |
| FCD | Gmax | 3 | | 18 | | 35 | 65 | | 107 | | 129 | | 142 | | 200 | | 220 | | 230 |
|  | C | 32 | | 63 | | 113 | 213 | | 340 | | 340 | | 340 | | 350 | | 350 | | 350 |
| FSR | Gmax | 0,52 | | 2,50 | | 3,50 | 5,70 | | 5,90 | | 5,95 | | 5,95 | | 5,95 | | 5,95 | | 5,95 |
|  | C | 25,00 | | 55,00 | | 75,00 | 105,00 | | 105,00 | | 110,00 | | 110,00 | | 95,00 | | 95,00 | | 95,00 |
| FHR | Gmax | 0.46 | | 1.708 | | 28.329 | 3.0 | | 3.5 | | 3.5 | | 3.5 | | 3.5 | | 3.5 | | 3.5 |
|  | C | 20,00 | | 48,00 | | 66,00 | 86,00 | | 86,00 | | 94,00 | | 94,00 | | 90,00 | | 90,00 | | 90,00 |
| FFL | Gmax | 0,58 | | 2,20 | | 2,93 | 3,10 | | 4,40 | | 53,20 | | 53,20 | | 53,30 | | 53,40 | | 53,40 |
|  | C | 30,00 | | 115,00 | | 115,00 | 150,00 | | 170,00 | | 170,00 | | 170,00 | | 170,00 | | 155,00 | | 155,00 |
| FWH | Gmax | 0,70 | | 1,20 | | 2,30 | 5,90 | | 10,60 | | 10,70 | | 10,70 | | 10,70 | | 10,70 | | 10,70 |
|  | C | 12,00 | | 13,00 | | 13,00 | 13,00 | | 80,00 | | 80,00 | | 80,00 | | 80,00 | | 80,00 | | 80,00 |
| FSP | Gmax | 0,07 | | 0,10 | | 0,16 | 0,16 | | 0,16 | |  | |  | |  | |  | |  |
|  | C | 2,50 | | 3,00 | | 4,00 | 5,50 | | 7,00 | |  | |  | |  | |  | |  |
| FSD | Gmax | 0,19 | | 0,51 | | 0,81 | 0,90 | | 0,92 | |  | |  | |  | |  | |  |
|  | C | 5,00 | | 13,00 | | 18,00 | 24,00 | | 30,00 | |  | |  | |  | |  | |  |
| FPR | Gmax | 0,96 | | 3,50 | | 7,10 | 12,60 | | 15,30 | | 16,80 | | 21,30 | | 23,60 | | 24,00 | | 24,10 |
|  | C | 15,00 | | 55,00 | | 70,00 | 100,00 | | 180,00 | | 180,00 | | 180,00 | | 180,00 | | 180,00 | | 180,00 |
| FCP | Gmax | 0,96 | | 4,50 | | 13,00 | 17,50 | | 23,30 | | 31,80 | | 37,30 | | 38,00 | | 38,00 | | 38,00 |
|  | C | 10,00 | | 40,00 | | 68,00 | 76,00 | | 74,00 | | 110,00 | | 114,00 | | 114,00 | | 114,00 | | 114,00 |

*Table F: Key biological rates calibrated for the invertebrate groups.*

| **Code** | **Growth rate [d-1]** | **Clearance rate** | **Linear mortality / lysis rate** | **Quadratic mortality** | **Light half-saturation constant [Wm-2]** | | **Nitrogen half-saturation constant  [mg N m-3 ]** |
| --- | --- | --- | --- | --- | --- | --- | --- |
| SF | 1.8 | 1.2 | 0.0000001 | 1.0e-9 |  |  | |
| HF | 0.15 | 0.3 | 0.0000001 | 1.0e-9 |  |  | |
| BF | 4.1 | 1.9 | 0.0000001 | 1.0e-11 |  |  | |
| PO | 2.0 | 0.8 | 0.0000001 | 1.0e-11 |  |  | |
| NE | 0.8 | 1.05 | 0.000196 | 1.0e-14 |  |  | |
| MA | 0.1 |  | 0.01 |  | 5 | 4 | |
| SG | 0.025 |  | 0.002 |  | 20 | 2 | |
| MS | 0.07 | 0.055 | 0.00001 | 1.0e-5 |  |  | |
| ZG | 0.06 | 0.045 | 0.0001 | 1.0e-4 |  |  | |
| ZM | 1.0 | 0.08 | 0.001 | 1.0e-5 |  |  | |
| ZS | 1.3 | 0.18 | 0.015 | 1.0e-4 |  |  | |
| PL | 1.85 |  | 0.00001 |  | 35 | 21 | |
| PS | 1.52 |  | 0.000001 |  | 45 | 28.5 | |

*Continue Table F*

| **Code** | **Lethal oxygen level**  **(KO2)**  **[mg O2 m-3]** | **Minimum oxygen concentration (KO2LIM) [mg O2 m-3]** | **Half oxygen mortality depth**  **[m]** | **Oxygen dependent mortality**  **[d-1]** |
| --- | --- | --- | --- | --- |
| SF | 3.0 | 50 | 0.001 | 0.00001 |
| HF | 3.0 | 50 | 0.001 | 0.00001 |
| BF | 1.0 | 10 | 0.005 | 0.0001 |
| PO | 1.0 | 10 | 0.005 | 0.0001 |
| NE | 1.0 | 10 | 0.005 | 0.0001 |
| MA | NA | 10 | NA | NA |
| SG | NA | 10 | NA | NA |
| MS | NA | 10 | NA | NA |
| ZG | NA | 10 | NA | NA |
| ZM | NA | 10 | NA | NA |
| ZS | NA | 10 | NA | NA |
| PL | 1.0 | 10 | 1.0 | 0 |
| PS | 1.0 | 10 | 1.0 | 0 |

Dietary interactions

The EwE models have a region-specific data-driven parameterization of fixed diet proportions, summing up to 1 per predator. The diet composition of the Baltic Atlantis model however, is an emergent property. This means it is not a priori determined. The amount of prey consumed by a predator depends on a number of factors such as functional response type, growth and consumption rate, gape size, assimilation efficiency and the so-called availability parameter which can not be directly measured and is somewhat akin (though not identical) to the vulnerability term in Ecopath with Ecosim. This parameter, ranging from 0 to 1, describes the fraction of total prey biomass available to a given predator, i.e. it does not alone determine how much of the prey is consumed. Although there are means of converting stomach content data into the availability parameter, they were shown to require significant recalibrations. The initial values of the availability parameters, specific to juvenile and adult groups, are selected on an order of magnitude level corresponding to a very general classification of potential prey-predator interaction strength. Based on previous Atlantis model experience and data and expert knowledge from the Baltic system, the following scheme is adopted when specifying the availability parameter: >0.3 – very strong interaction, 0.05-0.3 – strong interaction, 0.005-0.05 medium interaction, 0.0001-0.005 – weak interaction, 0.0 – no interaction. In order to maximize the flexibility in biological interactions, a wide envelope of interactions is allowed for by assigning for weak interactions whenever there is evidence of possible dietary interaction. While such a diet formulation was more challenging to parameterize and calibrate, its flexibility offered a unique opportunity to capture for example potential shifts in cod diet when available benthic food decreases – taking into account that cod is an opportunistic predator. The resulting diets remain flexible and this is part of the reason that the calibrated model does not project large scales shifts in total biomass for many functional groups, as they shift diets as needed when prey fluctuate. This is to make the model sensitive to possible new ecological states that might evolve under new environmental regimes favoring one biological group over another, investigating changed cascading dynamics or to including abrupt changes to bottleneck species.

The primary source of information to validate the emerging dietary patterns (e.g. Fig. 6) comes from stomach content analysis. Much of the relevant data and expert knowledge has been assembled while developing the SMS and regional ECOPATH models. Very detailed regional information on diets of both juvenile and adult vertebrates is available for most fish and HTL groups. On the other hand, parameterization of some groups with a higher internal diversity of species, such as the FPR group, was hindered by aggregating species with mutually exclusive diets. In this case, maximum model flexibility was given preference over tuning towards only the most representative species.

Due to a largely ubiquitous spatial distribution of preferred prey items, feeding patterns of benthic and pelagic invertebrates are much simpler to parameterize compared to vertebrate groups. Initial values for the key parameters such as availability of phytoplankton groups to zooplankton groups are taken from previous Atlantis applications as a first order constrain.

**3. Species aggregation into biological functional groups**

Although grouping different species into a single biological functional group, e.g. flounder, plaice, dab and turbot into the flatfish group carries a trade-off of introducing a bias in choosing the representative growth, maturity and recruitment, and dietary parameters, this had to be done to reduce the complexity of the model. Species are aggregated into biological functional groups using the following criteria: (i) they are an important component of the food-web, (ii) they are known to possess similar physiological traits (e.g. size, growth rate), (iii) they primarily predate on and are prey to organisms from the same trophic level, and (iv) they share common life-history traits (life expectancy, reproductive mode). Aggregating species into functional groups is a common pragmatic approach to modeling complexity of ecosystem model interactions, and is a common practice in contemporary ecological models (e.g. Atlantis, satellite PFT algorithms, trait-based models). Potential overlap with biological structures of other ecosystem models in the Baltic is considered to foster the linking and potential future coupling capability. Models such as ERGOM (e.g. Maar et al., 2011), ERSEM (e.g. Vichi et al., 2004), the Stochastic Multispecies Model (SMS; Lewy & Vinther, 2004), the mass-balance ECOPATH (Tomczak et al., 2009; Tomczak et al., 2012) have the potential to provide regional information for initial conditions and/or biological parameters for those biological groups for which insufficient field information was available and where there is functional overlap between groups in the different models (e.g. small phytoplankton, cyanobacteria, seagrass). The same models also provide first order evaluation of the robustness of the Baltic Atlantis model parameterization.

*Table G: Biological functional group structure of the Baltic Atlantis model. Groups are categorized according to the ecosystem level they represent. Key species aggregated within a functional group are listed. The first species in each list is the representative one for that particular functional group. Groups that are currently “turned off”, and which do not play a role in the biological interactions, are marked with an asterisk (*).*

| **Ecosystem level** | **Group name & model code** | **Species names** |
| --- | --- | --- |
| Marine mammals | Harbour porpoise (MHP) | *Phocoena phocoena (Harbour porpoise)* |
|  | Seals (SEA) | *Halichoerus grypus* (Grey seal)*, Pusa hispida botnica* (Ringed seal)*, Phoca vitulina* (Harbour (common) seal) |
| Seabirds | Pursuit-diving seabirds (SBD) | *Phalacrocoracidae* (Cormorants), *Cepphus grylle grylle* (Black guillemot), *Alca torda torda* (Razorbill), *Uria aalge* (Common guillemot) |
|  | *Surface-feeding seabirds (SBS) | *Laridae* (Gulls), *Sternidae* (Terns), and many other |
| Fish | Cod (FCD) | *Gadus morhua* (Atlantic cod) |
|  | Sprat (FSR) | *Sprattus sprattus* (European sprat) |
|  | Herring (FHR) | *Clupea harengus membras* (Baltic herring) |
|  | Whiting (FWH) | *Merlangius merlangus* (Whiting) |
|  | Flat fish (FFL) | *Platichtys flesus* (Flounder), *Pleuronectes platessa* (Plaice), *Limanda limanda* (Dab), *Psetta maxima* (Turbot), *Solea solea* (Sole), *Scopthalmus rhombus* (Brill), *Microstomus kitt* (Lemon sole) |
|  | Perch (FPR) | *Perca fluviatilis* (Perch), *Sander lucioperca (Stizostedion lucioperca;* Pikeperch (sander/zander)*), Esox lucius* (Northern pike), *Gymocephalus certuus* (Ruffe) |
|  | Cyprinids (FCP) | *Rutilus rutilus (*Roach), *Alburnus alburnus (*Bleak), *Leuciscu idus (*Ide), *Blicca bjoerkna (*White (silver) bream), *Abramis brama (*Bream (common bream)), *Vimba vimba (*Vimba (vimba bream)), *Scardinius erythropthalmus (*Rudd), *Leuciscus leuciscu (*Dace), *Carassius carassius (*Crussian carp), *Tinca tinca (*Tench), *Carassius gibelio (*Prussian carp), *Cyprinous carpio (*Carp), *Abramis ballerus (*Blue bream) |
|  | Small demersal fish (FSD) | *Neogobius melanostomus (*Round goby), *Gobius niger (Black goby), Pomatoschistus microps (*Common goby), *Promatoschistus minutus (*Sand goby), *Cottus gobio (Bullhead)* |
|  | Small pelagic fish (FSP) | *Gasterosteus aceluatus (*Three-spined stickleback), *Pungitus pungitus (*Nine-spined stickleback), and other sticklebacks |
| Benthic invertebrates | Nephrops (NE) | *Nephrops norvegicus* (Norway lobster) |
|  | Soft substrate filter feeders (SF) | *Macoma balthica, Mya arenaria, Mya truncata, Astarte borealis, Arctica islandica, Cerastoderma glaucum, Thyasira flexuosa, Musculus niger, Mysella bidentata*, and many other bivales |
|  | Hard substrate filter feeder (HF) | *Mytilus edulis, Mytilus trossulus, Modiolus modiolus* |
|  | Polychaetes (PO) | *Marenzelleria spp., Bylgides (Harmothoe) sarsi, Hediste diversicolor, Scoloplos armiger, Pholoe baltica, Polydora, Heteromastus filiformis, Lagis koreni (*trumpet worm), *Pygospio elegans, Terebellides stroemii* |
|  | Benthic deposit feeders (DF) | Amphipods, isopods and shrimp: *Monoporeia affinis, Pontoporeia femorata, Leucothoe spp., Monoculodes, Diastylis spp., Corophium volutator, Caprellidae, Saduria entomon, Ideotea baltica, Idotea chelipes, Jaera albifrons, Asellus, aquaticus, Cyathura carinata, Bathyporeia pilosa, Lekanesphaera hookeri, Crangon crangon, Crangon allmani, Palaemon adspersus, Palaemon elegans, Palaemon serratus* |
| Benthic primary producers | *Microphytobenthos (MB) |  |
|  | Macroalgae (MA) | *Fucus vesiculosus, Fucus radicans, Fucus serratus, Ascophyllum nodosum, Furcellaria lumbricalis* |
|  | Seagrass (SG) | *Zostera marina* (Eel grass)*, Zostera noltii* |
| Pelagic invertebrates | Mysids (MS) | *Neomysis integer, Mysis mixta, Mysis relicta, Mesopodopsis slabberi, Praunus flexuosus* |
|  | Gelatinous zooplankton (ZG) | *Aurelia aurita, Cyanea capillata, Pleurobrachia pileus, Mertensia ovum, Mniemiopsis leidyi* |
|  | Mesozooplankton (ZM) | *Acartia tonsa, Temora longicoris, Cyclopidae, Limnocalanus, Pseudocalanus, Paracalanus, Oncaea, Corycaeidae, Centropages, Cladocera, Rotifera* |
|  | Microzooplankton (ZS) | heterotrophic flagellates, cilliates |
| Pelagic primary producers | Large phytoplankton (PL) | diatoms |
|  | Small phytoplankton (PS) | autotrophic flagellates |
|  | *Cyanobacteria (PC) |  |
| Bacteria | Pelagic Bacteria (BP) |  |
|  | Sediment Bacteria (BB) |  |
| Detritus | Labile detritus (DL) |  |
|  | Refractory detritus (DR) |  |
|  | Carrion (DC) |  |

Table H provides a summary of the key general parameters assigned to each of the biological groups defined in the Baltic Atlantis model. For explanations of the functional behavior of each of the group types, i.e. FISH, SED_EP_FF, SM_PHY, etc., please refer to previous Atlantis publications (e.g. the Atlantis manual (Audzijonyte et al., 2017)). Mammals, seabirds and fish are age structured with a set amount of age groups (cohorts) and annual age classes within the age groups. Mammals, seabirds and whiting have two age classes within each age group, so for example age group 2 consists of age class 3 and 4. This is done to simplify the ecological processes. There is a maximum of 10 age groups (20 age classes). All fish, except whiting, only have one age class within each age group, with a maximum of 10 age groups (10 age classes). The current setting of the model allows for senescence, where the final age class dies into the following calendar year.

*Table H: Summary of basic parameters describing the general behavior of biological groups in the model, used as input to the model.*

| **Code** | **Index** | **Is**  **Turned**  **On** | **Name** | **Long**  **Name** | **Num**  **Cohorts** | **Num**  **Gene**  **Types** | **Num**  **Stages** | **Num**  **Spawns** | **Num**  **AgeClass**  **Size** | **Num**  **Stocks** | **Vertically**  **Migrates** |  |
| --- | --- | --- | --- | --- | --- | --- | --- | --- | --- | --- | --- | --- |
| MHP | 0 | 1 | Har_Porp | Harbor Porpoise | 10 | 1 | 2 | 1 | 2 | 1 | 1 |  |
| SEA | 1 | 1 | Seal | Seals | 10 | 1 | 2 | 1 | 2 | 1 | 1 |  |
| SBD | 2 | 1 | Diving_seabird | Pursuit diving seabirds | 10 | 1 | 2 | 1 | 2 | 1 | 1 |  |
| SBS | 3 | 0 | Surface_seabird | Surface feeding seabirds | 10 | 1 | 2 | 1 | 2 | 1 | 1 |  |
| FCD | 4 | 1 | Cod | Cod | 10 | 1 | 2 | 1 | 1 | 1 | 1 |  |
| FSR | 5 | 1 | Sprat | Sprat | 10 | 1 | 2 | 1 | 1 | 1 | 1 |  |
| FHR | 6 | 1 | Herring | Herring | 10 | 1 | 2 | 1 | 1 | 1 | 1 |  |
| FFL | 7 | 1 | Flat_Fish | Flat Fish | 10 | 1 | 2 | 1 | 1 | 1 | 1 |  |
| FWH | 8 | 1 | Whiting | Whiting | 10 | 1 | 2 | 1 | 2 | 1 | 1 |  |
| FSP | 9 | 1 | Small_Pel | Small Pelagic Fish | 5 | 1 | 2 | 1 | 1 | 1 | 1 |  |
| FSD | 10 | 1 | Small_Dem | Small Demersal Fish | 5 | 1 | 2 | 1 | 1 | 1 | 1 |  |
| FPR | 11 | 1 | Perch | Perch | 10 | 1 | 2 | 1 | 1 | 1 | 1 |  |
| FCP | 12 | 1 | Carp | Carp | 10 | 1 | 2 | 1 | 1 | 1 | 1 |  |
| NE | 13 | 1 | Nephrop | Nephrops | 1 | 1 | 1 | 1 | 1 | 1 | 0 |  |
| HF | 14 | 1 | Filter_Hard | Hard substrate filter feeders | 1 | 1 | 1 | 1 | 1 | 1 | 0 |  |
| SF | 15 | 1 | Filter_Soft | Soft substrate filter feeders | 1 | 1 | 1 | 1 | 1 | 1 | 0 |  |
| BF | 16 | 1 | Dep_Feed | Benthic deposit feeders | 1 | 1 | 1 | 1 | 1 | 1 | 0 |  |
| PO | 17 | 1 | Polych | Polychetes | 1 | 1 | 1 | 1 | 1 | 1 | 0 |  |
| MB | 18 | 0 | MicroPB | Microphtybenthos | 1 | 1 | 1 | 1 | 1 | 1 | 0 |  |
| MA | 19 | 1 | Macroalgae | Macroalgae | 1 | 1 | 1 | 1 | 1 | 1 | 0 |  |
| SG | 20 | 1 | Seagrass | Seagrass | 1 | 1 | 1 | 1 | 1 | 1 | 0 |  |
| MS | 21 | 1 | Mysid | Mysids | 1 | 1 | 1 | 1 | 1 | 1 | 1 |  |
| ZG | 22 | 1 | Gelat_Zoo | Gelatinous zooplankton | 1 | 1 | 1 | 1 | 1 | 1 | 1 |  |
| ZM | 23 | 1 | MesoZoo | Copepods | 1 | 1 | 1 | 1 | 1 | 1 | 1 |  |
| ZS | 24 | 1 | MicroZoo | Microzooplankton | 1 | 1 | 1 | 1 | 1 | 1 | 1 |  |
| PL | 25 | 1 | Diatom | Diatoms | 1 | 1 | 1 | 1 | 1 | 1 | 0 |  |
| PS | 26 | 1 | Flag | Autotrophic Flagellates | 1 | 1 | 1 | 1 | 1 | 1 | 0 |  |
| PC | 27 | 0 | Cyanos | Cyanobacteria | 1 | 1 | 1 | 1 | 1 | 1 | 0 |  |
| BP | 28 | 1 | Pelag_Bact | Pelagic Bacteria | 1 | 1 | 1 | 1 | 1 | 1 | 0 |  |
| BB | 29 | 1 | Sed_Bact | Sediment Bacteria | 1 | 1 | 1 | 1 | 1 | 1 | 0 |  |
| DL | 30 | 1 | Lab_Det | Labile detritus | 1 | 1 | 1 | 1 | 1 | 1 | 0 |  |
| DR | 31 | 1 | Ref_Det | Refractory detritus | 1 | 1 | 1 | 1 | 1 | 1 | 0 |  |
| DC | 32 | 1 | Carrion | Carrion | 1 | 1 | 1 | 1 | 1 | 1 | 0 |  |

*Table H: Continued.*

| **Horizontally**  **Migrates** | **Is**  **Fished** | **Is**  **Impacted** | **Is**  **TAC** | **Group**  **Type** | **Is**  **Predator** | **Is**  **Cover** | **Is**  **Silicon**  **Dep** | **Is**  **Assessed** | **Is**  **Catch**  **Grazer** | **Is**  **Over**  **Winters** | **Is**  **Cultured** | **Is**  **Hab**  **Depend** | **Num**  **Move**  **Seaons** |
| --- | --- | --- | --- | --- | --- | --- | --- | --- | --- | --- | --- | --- | --- |
| 1 | 1 | 1 | 0 | MAMMAL | 1 | 0 | 0 | 1 | 0 | 0 | 0 | 0 | 4 |
| 1 | 1 | 1 | 0 | MAMMAL | 1 | 0 | 0 | 1 | 0 | 0 | 0 | 0 | 4 |
| 1 | 1 | 1 | 0 | BIRD | 1 | 0 | 0 | 1 | 0 | 0 | 0 | 0 | 4 |
| 1 | 1 | 1 | 0 | BIRD | 1 | 0 | 0 | 1 | 0 | 0 | 0 | 0 | 4 |
| 1 | 1 | 1 | 1 | FISH | 1 | 0 | 0 | 1 | 0 | 0 | 0 | 0 | 4 |
| 1 | 1 | 1 | 1 | FISH | 1 | 0 | 0 | 1 | 0 | 0 | 0 | 0 | 4 |
| 1 | 1 | 1 | 1 | FISH | 1 | 0 | 0 | 1 | 0 | 0 | 0 | 0 | 4 |
| 1 | 1 | 1 | 1 | FISH | 1 | 0 | 0 | 1 | 0 | 0 | 0 | 0 | 4 |
| 1 | 1 | 1 | 1 | FISH | 1 | 0 | 0 | 1 | 0 | 0 | 0 | 0 | 4 |
| 1 | 1 | 1 | 1 | FISH | 1 | 0 | 0 | 1 | 0 | 0 | 0 | 0 | 4 |
| 1 | 1 | 1 | 1 | FISH | 1 | 0 | 0 | 1 | 0 | 0 | 0 | 0 | 4 |
| 1 | 1 | 1 | 1 | FISH | 1 | 0 | 0 | 1 | 0 | 0 | 0 | 0 | 4 |
| 1 | 1 | 1 | 1 | FISH | 1 | 0 | 0 | 1 | 0 | 0 | 0 | 0 | 4 |
| 0 | 1 | 1 | 0 | SED_EP_OTHER | 1 | 0 | 0 | 1 | 0 | 0 | 0 | 1 | 4 |
| 0 | 0 | 1 | 0 | SED_EP_FF | 1 | 1 | 0 | 1 | 0 | 0 | 0 | 1 | 4 |
| 0 | 0 | 1 | 0 | SED_EP_FF | 1 | 1 | 0 | 1 | 0 | 0 | 0 | 1 | 4 |
| 0 | 0 | 1 | 0 | SED_EP_OTHER | 1 | 0 | 0 | 1 | 0 | 0 | 0 | 1 | 4 |
| 0 | 0 | 1 | 0 | SED_EP_OTHER | 1 | 0 | 0 | 1 | 0 | 0 | 0 | 1 | 4 |
| 0 | 0 | 1 | 1 | MICRO-PHTYBENTHOS | 0 | 1 | 1 | 1 | 0 | 0 | 0 | 1 | 4 |
| 0 | 0 | 1 | 0 | PHYTOBEN | 0 | 1 | 0 | 1 | 0 | 0 | 0 | 1 | 4 |
| 0 | 0 | 1 | 0 | SEAGRASS | 0 | 1 | 0 | 1 | 0 | 0 | 0 | 1 | 4 |
| 0 | 0 | 0 | 0 | LG_ZOO | 1 | 0 | 0 | 1 | 0 | 0 | 0 | 1 | 4 |
| 0 | 0 | 0 | 0 | LG_ZOO | 1 | 0 | 0 | 1 | 0 | 0 | 0 | 0 | 4 |
| 0 | 0 | 0 | 0 | MED_ZOO | 1 | 0 | 0 | 1 | 0 | 0 | 0 | 0 | 4 |
| 0 | 0 | 0 | 0 | SM_ZOO | 1 | 0 | 0 | 1 | 0 | 0 | 0 | 0 | 4 |
| 0 | 0 | 0 | 0 | LG_PHY | 0 | 0 | 1 | 1 | 0 | 0 | 0 | 0 | 4 |
| 0 | 0 | 0 | 0 | SM_PHY | 0 | 0 | 0 | 1 | 0 | 0 | 0 | 0 | 4 |
| 0 | 0 | 0 | 0 | SM_PHY | 0 | 0 | 0 | 1 | 0 | 0 | 0 | 0 | 4 |
| 0 | 0 | 0 | 0 | PL_BACT | 0 | 0 | 0 | 0 | 0 | 0 | 0 | 0 | 4 |
| 0 | 0 | 0 | 0 | SED_BACT | 0 | 0 | 0 | 0 | 0 | 0 | 0 | 0 | 4 |
| 0 | 0 | 0 | 0 | LAB_DET | 0 | 0 | 0 | 1 | 0 | 0 | 0 | 0 | 4 |
| 0 | 0 | 0 | 0 | REF_DET | 0 | 0 | 0 | 1 | 0 | 0 | 0 | 0 | 4 |
| 0 | 0 | 0 | 0 | CARRION | 0 | 0 | 0 | 1 | 0 | 0 | 0 | 0 | 4 |

**4. Habitat structure**

In the Baltic Atlantic model five substrate types identified and mapped by HELCOM (http://helcom.fi/baltic-sea-trends/data-maps/biodiversity/balance) are assigned to three default model abiotic habitat types according to the similarity in ecosystem function. The bedrock substrate is made equivalent to model's "reef" habitat, hard bottom complex and hard clay correspond to model's "flat" habitat, while mud and sand are assigned to model's "soft" habitat class. Additionally, we consider the surface area covered by man-made structures (e.g. wind-mill parks, other large marine constructions) as equivalent of the model's "canyon" class. Table I lists the relative percentage cover by substrate per polygon. There are also dynamic biotic habitat types formed by five benthic biological groups: seagrass, macroalgae, microphytobenthos, soft-substrate filter feeders and hard-substrate filter feeders. All habitat classes are defined as % cover of each polygon, with the three basic abiotic adding up to 100% but canyon and biotic being calculated independently. Biotic habitat cover changes in time as a function of total biomass per box. Each biological group in the model is assigned a basic affinity (presence vs absence) to all habitats.

*Table I: Relative cover of each polygon with 3 types of bottom abiotic habitats: bedrock, sand, mud, and a fourth abiotic habitat which corresponds to man-made structures such as wind-mill parks, pipelines etc.*

| **Box_ID** | **bedrock** | **sand** | **mud** | **man-made** |  | **Box_ID** | **bedrock** | **sand** | **mud** | **man-made** |
| --- | --- | --- | --- | --- | --- | --- | --- | --- | --- | --- |
| **0** | 0.04 | 0.05 | 0.91 | 0.00 |  | **15** | 0.00 | 0.65 | 0.35 | 0.00 |
| **1** | 0.02 | 0.26 | 0.72 | 0.00 |  | **16** | 0.00 | 0.17 | 0.83 | 0.00 |
| **2** | 0.00 | 0.17 | 0.83 | 0.00 |  | **17** | 0.00 | 0.30 | 0.70 | 0.00 |
| **3** | 0.00 | 0.14 | 0.85 | 0.00 |  | **18** | 0.00 | 0.14 | 0.86 | 0.00 |
| **4** | 0.00 | 0.31 | 0.69 | 0.00 |  | **19** | 0.00 | 0.64 | 0.36 | 0.00 |
| **5** | 0.01 | 0.21 | 0.77 | 0.00 |  | **20** | 0.03 | 0.35 | 0.62 | 0.00 |
| **6** | 0.00 | 0.35 | 0.65 | 0.00 |  | **21** | 0.26 | 0.23 | 0.51 | 0.00 |
| **7** | 0.00 | 0.51 | 0.49 | 0.00 |  | **22** | 0.12 | 0.38 | 0.50 | 0.00 |
| **8** | 0.00 | 0.07 | 0.93 | 0.00 |  | **23** | 0.01 | 0.53 | 0.46 | 0.00 |
| **9** | 0.08 | 0.49 | 0.43 | 0.00 |  | **24** | 0.01 | 0.76 | 0.22 | 0.00 |
| **10** | 0.01 | 0.64 | 0.35 | 0.00 |  | **25** | 0.07 | 0.88 | 0.05 | 0.00 |
| **11** | 0.00 | 0.33 | 0.67 | 0.00 |  | **26** | 0.00 | 0.31 | 0.69 | 0.00 |
| **12** | 0.20 | 0.46 | 0.34 | 0.00 |  | **27** | 0.00 | 0.28 | 0.72 | 0.00 |
| **13** | 0.00 | 0.22 | 0.78 | 0.00 |  | **28** | 0.00 | 0.29 | 0.71 | 0.00 |
| **14** | 0.00 | 0.07 | 0.93 | 0.00 |  |  |  |  |  |  |

**5. Model calibration: spin-up and stability**

The time required to reach the state of equilibrium for the fish species reflects the time of moving one cohort through all the age groups, i.e. 10 years. The spin-up period of the lower trophic levels and the detritus groups was longest, this is despite their fast turnover times. Even though it is normally the higher trophic levels that determine the length of the spin-up, this is still a reflection of their food web role, where many groups depend on them, and the way in which they are influenced by many processes; together this means it takes some time for the aggregated conditions to stabilize (Fig 5).

It is concluded that the comprehensive data collection effort resulted in a very robust first set of parameters which did not need to be altered significantly throughout the calibration process. The parameters which were initially described with very little confidence levels require time-consuming balancing. These key parameters - growth rate, mortality rates, consumption rate – constitute the typically most sensitive areas of the parameters space regardless of the complexity and model structure.

Calibrating the availability parameter presented the biggest obstacle in obtaining a balanced ecosystem state. During the calibration process these values were frequently and drastically adjusted, sometimes by orders of magnitude. Due to lack of a measurable equivalent of the availability parameters, the criterion for making these adjustments was subjective and required a trial and error approach. The iterative changes aimed at preventing a group from becoming extinct due to overconsumption, or to bring the emergent diet to a state matching the extensive in-house expert knowledge on the subject or published field and modeling records in other cases. It should be noted that in the absence of objective multi-parameter optimization tools available, there are many possible alternative parameterization sets that would enable a balanced and steady ecosystem. Therefore, we cannot consider the current parameterization optimum. For instance, the same levels of primary production in the water column could be achieved by decreasing the availability of phytoplankton to zooplankton while simultaneously increasing natural mortality or decreasing nutrient and light acquisition parameter values. These rates are virtually non-measurable in the field and their published values are typically a product of mass balance calculations in NPZ type models. The majority of phytoplankton rate parameters we adopted from the ERGOM model and opted to keep them relatively unchanged, while focusing on adjusting the more arbitrary availability parameter.

Another source of great uncertainty, and thus an area of intense parameter tuning, was that of growth and consumption rates of vertebrate groups. While the initial guesses were fairly well constrained using size-based scaling laws of metabolic theory, up to an order of magnitude adjustments had to be made for some groups. The two criteria used to adjust these rates were: (i) obtaining an expected (= initial) structural and reserve weight per age group and (ii) an expected diet composition which is a nonlinear product of availability, growth rate, consumption rate and assimilation rate among other less important parameters.

Another difficulty is that there are likely several alternative sets of parameters which could describe the growth and reproduction dynamics of many biological groups.

*Table S: Annual average total Baltic Sea biomass [metric tons] of all active biological groups in the Baltic Atlantis model averaged over the last five years (from a total of year 60). For vertebrates, for which good constraints on abundance/biomass distributions were available, “x initial” factor difference as relative to initial/expected conditions is given as well.*

| **Group code** | **Biomass** | |  | **Group code** | **Biomass** |  |
| --- | --- | --- | --- | --- | --- | --- |
|  | **metric tons** | **x initial** |  |  | **metric tons** |  |
| MHP | 1628 | -0.05 |  | BF | 52624296 |  |
| SEA | 6976 | 0.00 |  | PO | 152441990 |  |
| SBD | 21544 | 0.26 |  | MA | 8825772 |  |
| FCD | 182379 | 0.14 |  | SG | 885008 |  |
| FSR | 7672327 | 2.72 |  | MS | 13289790 |  |
| FHR | 1709495 | -0.05 |  | ZG | 5987087 |  |
| FFL | 78867 | -0.16 |  | ZM | 73190896 |  |
| FWH | 133408 | -0.02 |  | ZS | 13918001 |  |
| FSP | 420132 | 0.27 |  | PL | 25904686 |  |
| FSD | 1030842 | 0.05 |  | PS | 14534864 |  |
| FPR | 1186766 | 0.15 |  | BP | 13093200 |  |
| FCP | 773445 | 0.05 |  | BB | 5016428 |  |
| NE | 75633 |  |  | DL | 7638727 |  |
| HF | 5840067 |  |  | DR | 10859636 |  |
| SF | 27103291 |  |  | DIN | 193152692 |  |
|  |  |  |  | Total Biomass | 444447181 |  |

**6. Result graphs**

**Fig B**. 120 year simulation run

**Fig D**. One-year cycle of Chl-a in the different polygons

**Fig E**. Relative biomass – initial condition values compared with simulation outcome

**Fig F**. Diet composition of all predators

**Fig G**. Biomass per age group over time for all vertebrates

**Fig H**. Demography distribution for all vertebrates - the number of individuals for each age group

**Fig I**. Geographical distribution of all functional groups

**Fig J**. Geographical distribution of oxygen in the different layers. Panel 1 = top layer, panel 7 = bottom layer

**Fig K**. Total biomass of Cod for scenario 1 (baseline) compared to scenario 5

**Fig L**. Relative prey biomass for predator cod, baseline compared to scenario 5

**Fig M**. One-year cycle of nutrients in the different polygons

# C. The FISHRENT model

FISHRENT is a robust bio-economic model with a long history of development (Cobb & Douglas, 1928; Salz et al., 2010). The model is a deterministic bio-economical model applying dynamic feedback between fisheries and exploited fish stocks in the sense that year-on-year changes observed for e.g. total fish stock biomass in the main stock areas (see below) are fed back into the model to update fishing opportunities, while at the same time year-to-year changes in fishing opportunities (represented by fishing capacity) will feed back into the model and affect stock development.

The biological module in FISHRENT is founded on ICES published data ([www.ices.dk](file:///C:\Users\artur\Documents\Research\BalticAtlantis\papers\IMAGE\July_2016\www.ices.dk)) on exploited fish stock status, i.e. biomass (www.ices.dk) for the key stocks where production, growth and recruitment parameters are estimated externally to populate the necessary biological functions in the model that tracks the status of the fish stocks. The input of the different fish stocks is not spatially explicit according to main stock area, i.e. in the present context, the model covers only one stock unit per species per main area covering the Kattegat and the western Baltic Sea, respectively. FISHRENT uses a Cobb-Douglas production function [105] to estimate catch value year-on-year. A detailed outline of the equations included in the model is given below.

**Fig C.** The FISHRENT model diagram, here applied to Kattegat and Western Baltic.

**1. FISHRENT KWB**

Economic models are usually constructed as normative (thought) models i.e. models that recommend the best possible solutions from an economic point of view (also named what’s best), or as models that project what may happen when exogenous limitations are imposed onto the model (also named what if). Some economic models are positivistic i.e. what things are (Hausman, 1989). Therefore, economic projections are usually carried out and compared with a base line case. Validation of such models is therefore based on coherence with economic theory rather than correspondence with real life data.

(Frost et al., 2011) aimed to validate FISHRENT in terms of coherence with economic theory, which in normative models may be of greater importance than empirical evidence. However, the latter must not be disregarded in real life assessments. In the EU-FP7 project MYFISH, the FISHRENT has been calibrated using real-life data and the base year output validated against real life data, with good correspondence.

Arnason (2000) classifies fisheries economic models into three groups: 1) Analytical with no or little empirical content 2) Empirical with an empirical description of a fishery 3) Numerical and generally solved by numerical methods using computers.

FISHRENT, in its basic version, covers all three model types of which type 3 is the most difficult to construct and operate. FISHRENT KWB is a combination of a type 2 and a type 3 model. An empirical/numerical model in the sense that it needs numerically estimated values for a large number of the input parameters, while some input parameters must be estimated empirically due to lack of data. A model like this is sensitive to initial data input and requires continuous data collection and data validation. As such the value of using FISHRENT KWB lies in relative rather than absolute outputs.

**2. Calibration of FISHRENT I: Danish fleet dat**a

The FISHRENT model has been calibrated with 2012 fisheries dynamics parameters as the initial year for forward projections, given nutrient load scenarios. This calibration year is chosen because adequate data for fisheries dynamics parameters to inform the FISHRENT model are not available for 2005 but only for the period from 2010 and onwards. Furthermore, significant changes in fisheries management and regulations were implemented for the Danish fishery in 2007-2008 with the introduction of the individual vessel based quotas for demersal stocks and fisheries similar to existing individual quota regulation for pelagic stocks and fisheries. Consequently, this regulation changed the fishing behavior significantly from 2007 and in the following years for main fisheries in the Kattegat and western Baltic Sea area. Accordingly, 2012 gives a more realistic and precise picture of the fishing patterns in the 60-year projection period compared to the 2005 fishing pattern. In addition, the compliance to regulations increased with introduction of the individual quotas in general which overall improved the quality of the fishery data. Finally, the river run-off of nutrients was at the same level in 2012 as in 2005, while for the intermediate years such as 2009-2010 the nutrient river runoff significantly deviated from this level. The implications for choosing 2012 instead of 2005 as calibration year are described further below, as the initial biomass and fishing mortality will be different.

Danish vessels that operated in 2012, in the Kattegat and/or in the Western Baltic, including the Sound and the Great Belt, have been extracted from the Danish logbook register (obtained from the Danish AgriFish Agencies database; [www.naturerhverv.dk/fiskeri/erhvervsfiskeri/indberetning-og-foering-af-logbog](http://www.naturerhverv.dk/fiskeri/erhvervsfiskeri/indberetning-og-foering-af-logbog)). In total, 259 vessels were selected and distributed on gear type and operating area as displayed in Table J. This covered 123 vessels that operated solely in the Kattegat and areas outside the western Baltic, 93 vessels that operated solely in the Western Baltic and areas outside Kattegat, and 43 vessels operated in both areas and other areas in 2012. Overall, 9 vessel segments are represented, defined by gear and vessel length. Total catch value and catch value distribution by segment in 2012, including catches in other areas than the Kattegat and the Western Baltic, are given in Table K. Of these 9 segments four (Netters and liners less than 12 meters, and Trawlers 12-15 meters, 15-18 meters and 18-24 meters) cover more than 85% of the total catch value. It was therefore decided to focus on these four fleet segments in the model evaluations.

Table M displays the economic input data used to initialize the model (used in equations a4 to a9 in the model description below). The data is based on the 2012 account statistics data for the Danish fishing fleet. The ‘Revenue scaling’ parameter is the amount the revenue obtained from catching the 7 target species in the model is scaled up to get the full revenue of the fleet segment from catching all species in all waters the fleet operates in (corresponding to the *OsFf*  parameter in equation a4 below). This number is based on landings values (from the logbook register) obtained by each fleet in 2012.

Table N displays the prices used to evaluate revenue in the model (cf. equation a4 below). These are also based on 2012 logbook data for catch weight and value for each included fleet segment and species.

Output from FISHRENT comprises total revenues, costs, profits and the Net Present Values (NPVs) resulting from operating in all waters that the included fleets are active in, and not only in Kattegat and the Western Baltic. To evaluate an estimate of the revenues, costs and profits resulting from operating in the Kattegat and the Western Baltic alone, revenues and revenue-based costs are scaled down by revenue fractions in the Kattegat and the Western Baltic for each fleet, while effort dependent costs are scaled down by effort fractions in the Kattegat and the Western Baltic. Both the revenue and effort fractions, displayed in Table O, are based on 2012 data.

**3. Calibration of FISHRENT II: Biological data**

In the model evaluations, focus is on cod caught in the Kattegat (COD_KA), cod caught in the Western Baltic (COD_WB), and on Sprat, Herring, Whiting, Flatfish (represented by plaice and flounder) and Nephrops, all groups are aggregated over the Kattegat and the Western Baltic (SPR_KAWB, HER_KAWB, WHI_KAWB, FLAT_KAWB, NEP_KAWB). These species represent the most abundant, ecological important and commercially valuable species in the WBS. Table L shows how much the catch value of each of these species constituted of the total catch value for each of the four included fleet segments in 2012. Cod caught in the Western Baltic and Nephrops are the most important species followed by Sprat and Flatfish.

Table J displays the initial biomasses used in FISHRENT (the ‘TSB Atlantis’ column), used to evaluate catches (cf. equation a10 below). These are equal to the equilibrium biomasses that have been estimated by Atlantis (details of the calculation of the biomass levels in 2005 for ATLANTIS are given in section B.2.), together with the long term relative changes in these for the main fish stocks given the nutrient load scenarios. These relative changes in biomass have accordingly been applied to the initial biomasses used in FISHRENT. To limit the effects of bias due to differences in the two model calibrations, we explicitly use relative and not absolute changes in fish biomass as inputs to economic scenarios evaluated in FISHRENT, and as such the linking of the models are based on the relative changes in fish biomass estimated by ATLANTIS given eutrophication levels which accordingly are applied as similar relative changes in FISHRENT and projected forwards. Consequently, the presented analysis cannot be viewed as time series forecast but rather as a time slice scenario exploration approach.

Table J moreover displays total allowable catches (TAC) in 2012 used to initialise the FISHRENT model for the seven species groups included in the model. The TACs are used in the catch constraint of the FISHRENT optimization (equation a2 below).

It must be noticed that the initial levels of TAC for the most important fish stocks both in the ecosystem and for the fisheries main fish stocks are both in Atlantis and FISHRENT models held constant throughout the projection period, and equal to the values given in Table J. Moreover the TSBs are held constant in FISHRENT and equal to the ‘TSB Atlantis’ values. This has been done to distinguish and evaluate effects of different nutrient load scenarios which are not potentially confounded with effects of changes in fisheries dynamics, fisheries management, and fishing mortality. For the same reason recruitment has been set equal to zero in FISHRENT throughout the projection period.

**Table J. Total Stock Biomass, TSB (tonnes), and natural mortalities (M), target fishing mortalities (F) and TACs (tonnes) in 2012, for the seven species groups included in the FISHRENT model and TSB and F from the Atlantis model, also input for the FISHRENT model.**

| **Species** | **TSB (2005 ICES/Atlantis)** | | | **M** | **Target F (2005 ICES/Atlantis)** | | **TAC** |
| --- | --- | --- | --- | --- | --- | --- | --- |
| **COD_KA1** | | 6625 (4823/6155) | 0.266 | | | 0.4 (1.0/0.32) | 133 |
| **COD_WB2** | | 58454 (48874/47608) | 0.2 | | | 0.6 (1.1/0.32) | 21300 |
| **SPR_KAWB3** | | 108368 (NA/422444) | 0.34 | | | 0.3 (0.5/0.07) | 44341 |
| **HER_KAWB4** | | 141883 (215654/118916) | 0.244 | | | 0.26 (0.6/0.12) | 25358 |
| **WHI_KAWB5** | | 7169 (NA/130682) | 0.7 | | | 0.3 (0.3/0.10) | 500 |
| **FLAT_KAWB6** | | 16545 (NA/33158) | 0.15 | | | 0.25 (0.6/0.60) | 2000 |
| **NEP_KAWB7** | | 44307 (NA/75633) | 0.266 | | | 0.1 (0.1/0.07) | 2766 |

The corresponding values for 2005 is given in brackets originating from the same sources (where available) and used as inputs to Baltic Atlantis (either from ICES or based on the SMS model). For the F’s it is observed F’s rather than target F according to long term management plans. Sources for the data are given in table footers. M values are constant over years. KA = Kattegat, WB = Western Baltic Sea, SPR = sprat, HER = herring, WHI = whiting, FLAT = flatfish, NEP = Nephrops.

**1** [82], Cod in division IIIaS (Kattegat)

2 [83]

3 [84]

4 [84]

5 [85]

6 [86]

7 [87]

Finally, Table Q displays the TAC shares used in the model, i.e. the share each fleet segment has of the total TAC of each of the species groups (used in equation a2 below).

**4. Calibration of the Cobb Douglas Catch equations**

Landings are evaluated in FISHRENT using the Cobb-Douglas functions (Cobb & Douglas, 1928) (cf. equation a10 below). Constant returns to scale is assumed, i.e. that the sum of the effort and biomass elasticities is 1. This assumption is supported by a study of the Iberian-Atlantic hake fishery, where the catch-effort (alpha) and catch-stock coefficients (beta) in the Cobb-Douglas function are estimated at 0.59 and 0.24, respectively, for the trawl fleet and 0.14 and 0.74 for the gill net fleet (Garza-Gil et al., 2003). Others find increasing returns to scale (IRS). (Eide et al., 2003) e.g. estimate alpha and beta to be 1.23 and 0.42, respectively, for the Norwegian bottom trawlers targeting cod in a model with technical progress, and (Kronbak & Lindroos, 2005) estimates alpha and beta to be 0.75 and 0.64, respectively, for bottom trawlers in the Baltic Sea. It is reasonable to expect that the catch-effort elasticity is higher for trawlers than for gill netters and vice versa for the catch-stock elasticity. Therefore, the catch-effort elasticity alpha has been set to 0.6 for the trawlers and 0.4 for the netters in the present context, while the catch-stock elasticity beta has been set to 0.4 for the trawlers and 0.6 for the netters. The corresponding intercepts, i.e. the value of the Cobb-Douglas functions when both biomass and effort are unity, are given in Table R. These are based on average logbook effort and landings data for the included fleet segments in 2012.

**5. Calibration of FISHRENT vs. Calibration of Atlantis**

As outlined above the FISHRENT model is initiated with the long run equilibrium biomasses estimated from Atlantis, rather than the original 2012 biomasses (also listed in the Table J[[1]](#footnote-1)), even though the model is calibrated for 2012 for the fishing fleets. Although the 2012 biological conditions are not the same as the 2005 conditions used to calibrate the Baltic Atlantis, the differences in TSBs for most fish groups are not very large, i.e. orders of magnitude. These values cover the main fish stocks being most important both in ecosystem and fisheries context in the Kattegat and the Western Baltic area and the values are generally in the same order of magnitude and at comparable levels as the biomasses in 2005 used in the initialization of the ATLANTIS model (shown in brackets in Table J). However, there are exceptions with respect to whiting and flatfish whose biomasses appear to be higher in 2012, and sprat whose biomass appears to be lower in 2012 compared to 2005, in the initial inputs to the Baltic Atlantis model. In general, ICES assessments estimate extensive yearly variability in total stock biomasses for those stocks because of high recruitment variability and relatively few year classes in the cohorts (see references in Table J).

The above are naturally quite strong assumptions, but for the present purpose of assessing how well the

**6. FISHRENT and linking to Atlantis**

An important limitation with using FISHRENT is the fact that there is no cyclic feedback between the economic and fisheries structural changes simulated by FISHRENT resulting in changing fishing mortality and the biological production evaluated by the Baltic Atlantis. The current configuration of the integrated modeling framework does not therefore allow for time series forecast simulations. Rather we demonstrate the capability of exploring time-slice long term scenario economic consequences in an ecosystem brought to a state of a new equilibrium under strong human-induced perturbations. Naturally, a time series forecast would require very different assumptions, both in terms of economic and biological feedbacks.

More specifically, relative (%) change in annual average biomass of the selected commercially harvested biological groups per polygon is converted into relative biomass change of these groups in the two regions: the Kattegat and the Western Baltic, providing for exploratory bio-economic scenario analysis of effects on the fishery due to changed eutrophication. Consequently, there is at present no dynamic feedback between changes in the FISHRENT fishing and fleet economics back into the biological productivity simulated by Baltic Atlantis. The constant fishing pressure applied in Baltic Atlantis is not necessarily equivalent to the fishing pressure modeled in FISHRENT. To illustrate an example of the potential ecosystem impacts of using different F values, we analysed the sensitivity of the model using different F values on cod and sprat. Details of this sensitivity study are presented in the main paper (Fig 12).

**7. Model equations**

In the optimization scenarios run with the FISHRENT KWB model, the NPV of the total fishery represented by the four fleets, over a period of 25 years, is maximized, given the restriction that the total catches of target species are kept below the set TACs each year. Thus, the optimization scenarios can be characterized by Maximum Economic Yield (MEY) (Gordon, 1954; Scott, 1955) given long term sustainability of the included stocks.

Thus, in the present context FISHRENT maximizes the total net present value (NPV) of the fishery (the objective function) over a given time period (set to 25 years in the present context):

(a1)

Where is the profit obtained by fleet *f* (the sum over *f* being over the fleet segments included in the model, 4 in all in the present context, cf. the discussion of calibration below) in year *y*, and the discount rate, which is in the present context set to 3.5%. The independent variables in the maximization are the number of vessels in fleet segment *f* in year *y* and the number of days at sea per vessel exerted by fleet segment *f* in year *y*. Thus, the actors in FISHRENT are the fishers, or more specifically fleet segments, choosing to use their DAS per vessel, and invest/disinvest in number of vessels, in an economically optimal way over the simulation period. It is assumed that the number of vessels of a given fleet segment can only change by ±4%-10% per year. Thus that a fleet cannot ‘sell out’ all vessels from one year to the next.

In the model optimizations, total catches of each target species species are restricted under the TACs. ‘Total catches’ refers both to the sum of the catches taken by the four fleet segments, and the catches taken by all other fleets. ‘Target species’ include all species, except for the cod in Kattegat (COD_KA) and the whiting in both Kattegat and the Western Baltic Sea (WHI_KAWB), seeing that these are not in the present context considered target species for the included fleets (a target species is currently defined in the model as a species for which the value share of the total fleet catch value in 2012 is more than 2%), and as such should not act as choke species (i.e. one particular species for which you finished your quota and prevents you from fishing any further out of risk to exceed the quota).

Thus, the maximization given in equation (a1) is performed subject to the constraint that the total catch taken of target species *s* in year *y* by the total Danish fleet operating in Kattegat and the Western Baltic is less than the Danish quotas in these areas:

(a2)

Where is the catch, less high-grading[[2]](#footnote-2), of target species *s* taken by fleet segment *f* in year *y* (see equation a10), is the TAC of species *s* in year *y* (cf. Table J). This is assumed constant over the simulation period in the present context, as explained above. is the share of the TAC of species *s* historically taken by fleet segment *s* (cf. Table Q). The sum in the nominator on the left side of the equation is the catch of species *s* taken by the fleet segments included in the model, while the sum in the denominator is the historical fraction these segments takes of the TAC of species *s*, which is used to scale the catches determined by the model up to the full catches of species *s* in year *y*.

The profit of fleet *f* in year *y* is given by:

(a3)

Where is the revenue, the fuel costs, the crew costs, the other variable costs, the fixed costs and the capital costs of fleet *f* in year *y*.

The revenue is given by:

(a4)

Where is the price (cf. Table N) obtained by fleet *f* for species *s* in all years (prices are in the present context assumed constant over the simulation period).

The fuel costs are given by:

(a5)

Where are the fuel costs per sea day (cf. Table M), and is the total effort (number of sea days) exerted by fleet segment *f* in year *y*

The crew costs are given by:

(a6)

Where is the fraction of the revenue used as wages (cf. Table M).

The variable costs are given by:

(a7)

Where is the fraction that the variable costs will constitute of the revenue (cf. Table M).

The fixed costs are given by:

(a8)

Where is the fixed cost per vessel in fleet *f* (cf. Table M).

The capital costs are given by:

(a9)

Where is the capital costs per vessel in fleet segment *f* (cf. Table M).

The catch, less highgrading, of species *s* taken by fleet *f* in year *y* is given by the Cobb-Douglas form:

(a10)

where , and are the parameters of the Cobb-Douglas function (a0 given in Table R, a1 and a2 discussed in the section describing calibration above). is the total stock biomass of species *s* in year *y*, which is in the present context assumed constant over the simulation period (‘TSB Atlantis’ in Table J).

**8. Tables**

*Table K. Danish fleet segments operating either in the Kattegat (KA), the Western Baltic (WB) or in both areas (KAWB) in 2012.*

| **Fleet Segment** | **Area** | **Number of vessels** |
| --- | --- | --- |
| **GK1215m** | WB | 2 |
| **GK1518m** | KA | 1 |
| **GK1518m** | WB | 3 |
| **GKu12m** | KA | 12 |
| **GKu12m** | WB | 42 |
| **GKu12m** | KAWB | 11 |
| **JOLRUSu12m** | KA | 3 |
| **JOLRUSu12m** | WB | 21 |
| **JOLRUSu12m** | KAWB | 1 |
| **SNV1824m** | WB | 4 |
| **TRA1215m** | KA | 36 |
| **TRA1215m** | WB | 10 |
| **TRA1215m** | KAWB | 14 |
| **TRA1518m** | KA | 41 |
| **TRA1518m** | WB | 7 |
| **TRA1518m** | KAWB | 13 |
| **TRA1824m** | KA | 22 |
| **TRA1824m** | WB | 1 |
| **TRA1824m** | KAWB | 2 |
| **TRAu12m** | KA | 8 |
| **TRAu12m** | WB | 3 |
| **TRAu12m** | KAWB | 2 |

Note: ‘GK1215m’=Netters and Liners 12-15 meters, ‘GK1518m’=Netters and Liners 15-18 meters, ‘GKu12m’=Netters and Liners below 12 meters, ‘JOLRUSu12m’=Dinghies below 12 meters, ‘SNV1824m’=Danish Seine 18-24 meters, ‘TRA1215m’=Trawlers 12-15 meters, ‘TRA1518m’=Trawlers 15-18 meters, ‘TRA1824m’=Trawlers 18-24 meters, ‘TRAu12m’=Trawlers below 12 meters.

*Table L. Catch value and catch value distribution for the 9 Danish fleet segments operating in the Kattegat and/or the Western Baltic in 2012.****The table shows that Netters and liners less than 12 meters, and Trawlers 12-15 meters, 15-18 meters and 18-24 meters cover more than 85% of the total catch value. It has therefore been decided to focus on these four fleet segments in the model evaluations.***

| **Fleet segment** | | **Catch Value (1000 €)** | **Catch value distribution (%)** | |  |
| --- | --- | --- | --- | --- | --- |
| **GK1215m** | 276 | | | 0.44 | |
| **GK1518m** | 2053 | | | 3.28 | |
| **GKu12m** | 6113 | | | 9.75 | |
| **JOLRUSu12m** | 1984 | | | 3.17 | |
| **SNV1824m** | 1971 | | | 3.14 | |
| **TRA1215m** | 11675 | | | 18.62 | |
| **TRA1518m** | 21666 | | | 34.56 | |
| **TRA1824m** | 15525 | | | 24.76 | |
| **TRAu12m** | 1427 | | | 2.28 | |

Note: ‘GK1215m’=Netters and Liners 12-15 meters, ‘GK1518m’=Netters and Liners 15-18 meters, ‘GKu12m’=Netters and Liners below 12 meters, ‘JOLRUSu12m’=Dinghies below 12 meters, ‘SNV1824m’=Danish Seine 18-24 meters, ‘TRA1215m’=Trawlers 12-15 meters, ‘TRA1518m’=Trawlers 15-18 meters, ‘TRA1824m’=Trawlers 18-24 meters, ‘TRAu12m’=Trawlers below 12 meters.

*Table M. Economic input data for the 4 fleet segments included in the FISHRENT model, based on 2012 data.*

|  | **GKu12m** | **TRA1215m** | **TRA1518m** | **TRA1824m** |
| --- | --- | --- | --- | --- |
| **Fuel cost per Day (1000 €)** | 0.0575 | 0.2975 | 0.4686 | 0.8129 |
| **Crew Share of revenue** | 0.4963 | 0.3938 | 0.3711 | 0.3334 |
| **Variable cost share of revenue** | 0.1484 | 0.0913 | 0.0858 | 0.1008 |
| **Fixed cost per vessel (1000 €)** | 29.80 | 56.24 | 93.56 | 166.28 |
| **Capital cost per vessel (1000 €)** | 15.56 | 31.81 | 66.95 | 141.34 |
| **Investment price (1000 €)** | 76.9 | 198.5 | 492.4 | 956.0 |
| **Revenue scaling** | 1.76 | 1.72 | 2.11 | 3.14 |

*Table N. Species prices (1000 €/tonnes*) based on 2012 landings data.

| **Species** | **GKu12m** | | **TRA1215m** | | **TRA1518m** | | **TRA1824m** | |  |
| --- | --- | --- | --- | --- | --- | --- | --- | --- | --- |
| **COD_KA** | | 3.23 | | 2.71 | | 2.67 | | 3.38 | |
| **COD_WB** | | 2.46 | | 1.65 | | 1.48 | | 1.84 | |
| **SPR_KAWB** | | 0.00 | | 0.26 | | 0.26 | | 0.26 | |
| **HER_KAWB** | | 0.57 | | 0.65 | | 0.50 | | 0.60 | |
| **WHI_KAWB** | | 0.75 | | 0.75 | | 0.81 | | 0.81 | |
| **FLAT_KAWB** | | 1.12 | | 0.85 | | 0.80 | | 0.88 | |
| **NEP_KAWB** | | 18.10 | | 8.52 | | 8.37 | | 8.58 | |

*Table O. Revenue and effort Fractions in Kattegat and Western Baltic of total Revenue obtained and effort exerted in 2012 in all waters by the four fleet segments included in the FISHRENT model.*

|  | **Kattegat** | | **Western Baltic** | |
| --- | --- | --- | --- | --- |
|  | **Rev fraction** | **Effort fraction** | **Rev fraction** | **Effort fraction** |
| **GKu12m** | 0.08 | 0.06 | 0.62 | 0.61 |
| **TRA1215m** | 0.44 | 0.58 | 0.21 | 0.11 |
| **TRA1518m** | 0.32 | 0.49 | 0.20 | 0.08 |
| **TRA1824m** | 0.28 | 0.16 | 0.02 | 0.005 |

*Table P. Catch value fraction (%) of species included in the FISHRENT model of total catch value in 2012 for the four fleet segments included in the model.*

|  | **GKu12m** | | **TRA1215m** | | **TRA1518m** | | **TRA1824m** | |  |
| --- | --- | --- | --- | --- | --- | --- | --- | --- | --- |
| **COD_KA** | | 0.1 | | 0.2 | | 0.2 | | 0.2 | |
| **COD_WB** | | 44.62 | | 13.54 | | 9.23 | | 0.63 | |
| **SPR_KAWB** | | 0.00 | | 4.04 | | 7.14 | | 14.75 | |
| **HER_KAWB** | | 0.41 | | 0.58 | | 2.86 | | 7.18 | |
| **WHI_KAWB** | | 0.01 | | 0.06 | | 0.08 | | 0.00 | |
| **FLAT_KAWB** | | 11.38 | | 3.71 | | 2.92 | | 0.34 | |
| **NEP_KAWB** | | 0.02 | | 35.83 | | 25.02 | | 8.75 | |
| **Total** | | 57.65 | | 65.37 | | 53.19 | | 32.00 | |

*Table Q. TAC shares for the four fleet segments included in the FISHRENT model for each of the 7 species groups.*

| **Species** | **GKu12m** | **TRA1215m** | **TRA1518m** | **TRA1824m** |
| --- | --- | --- | --- | --- |
| **COD_KA** | 0.02 | 0.09 | 0.14 | 0.08 |
| **COD_WB** | 0.05 | 0.06 | 0.00 | 0.05 |
| **SPR_KAWB** | 0.04 | 0.13 | 0.20 | 0.00 |
| **HER_KAWB** | 0.00 | 0.05 | 0.07 | 0.00 |
| **WHI_KAWB** | 0.02 | 0.04 | 0.00 | 0.00 |
| **FLAT_KAWB** | 0.25 | 0.40 | 0.03 | 0.31 |
| **NEP_KAWB** | 0.18 | 0.23 | 0.06 | 0.00 |

*Table R. Cobb-Douglas intercept parameters (tonnes) used in the FISHRENT model*

| **Species** | **GKu12m** | **TRA1215m** | **TRA1518m** | **TRA1824m** |
| --- | --- | --- | --- | --- |
| **COD_KA** | 0.0006 | 0.0022 | 0.0028 | 0.0020 |
| **COD_WB** | 0.0653 | 0.0732 | 0.0843 | 0.0045 |
| **SPR_KAWB** | 0 | 0.1087 | 0.2891 | 0.5923 |
| **HER_KAWB** | 0.0015 | 0.0055 | 0.0548 | 0.1110 |
| **WHI_KAWB** | 0.0001 | 0.0017 | 0.0029 | 0.0001 |
| **FLAT_KAWB** | 0.0778 | 0.0463 | 0.0817 | 0.0085 |
| **NEP_KAWB** | 0 | 0.0419 | 0.0451 | 0.0151 |

**9. *FISHRENT results:***

*Table T. Total revenue (mill EUR) over the period 2012-2037 for the four fleet segments in each of the four scenarios. Revenue is shown over all waters shown, together with revenue in Kattegat and in the Western Baltic*

| **Scenario** | **Sea** | **TRA1215m** | **TRA1518m** | **TRA1824m** | **GKu12m** |
| --- | --- | --- | --- | --- | --- |
| **Scenario 1** | **Total** | 216,2 | 541,0 | 325,2 | 147,4 |
|  | **Kattegat** | 95,1 | 173,1 | 91,0 | 11,8 |
|  | **Western Baltic** | 45,4 | 108,2 | 6,5 | 91,4 |
| **Scenario 2** | **Total** | 216,3 | 540,8 | 325,2 | 147,4 |
|  | **Kattegat** | 95,2 | 173,1 | 91,0 | 11,8 |
|  | **Western Baltic** | 45,4 | 108,2 | 6,5 | 91,4 |
| **Scenario 3** | **Total** | 259,0 | 527,1 | 294,1 | 139,4 |
|  | **Kattegat** | 113,9 | 168,7 | 82,3 | 11,2 |
|  | **Western Baltic** | 54,4 | 105,4 | 5,9 | 86,4 |
| **Scenario 4** | **Total** | 227,6 | 535,2 | 326,0 | 143,4 |
|  | **Kattegat** | 100,2 | 171,3 | 91,3 | 11,5 |
|  | **Western Baltic** | 47,8 | 107,0 | 6,5 | 88,9 |

*Table U. Total fuel costs (mill EUR) over the period 2012-2037 for the four fleet segments in each of the four scenarios. Fuel costs are shown over all waters shown, together with fuel costs in Kattegat and in the Western Baltic*

| **Scenario** | **Sea** | **TRA1215m** | **TRA1518m** | **TRA1824m** | **GKu12m** |
| --- | --- | --- | --- | --- | --- |
| **Scenario 1** | **Total** | 11,4 | 40,0 | 31,8 | 3,1 |
|  | **Kattegat** | 6,6 | 19,6 | 5,1 | 0,2 |
|  | **Western Baltic** | 1,3 | 3,2 | 0,2 | 1,9 |
| **Scenario 2** | **Total** | 11,4 | 40,0 | 31,8 | 3,1 |
|  | **Kattegat** | 6,6 | 19,6 | 5,1 | 0,2 |
|  | **Western Baltic** | 1,3 | 3,2 | 0,2 | 1,9 |
| **Scenario 3** | **Total** | 16,5 | 41,7 | 28,4 | 2,7 |
|  | **Kattegat** | 9,6 | 20,4 | 4,5 | 0,2 |
|  | **Western Baltic** | 1,8 | 3,3 | 0,1 | 1,6 |
| **Scenario 4** | **Total** | 12,9 | 41,4 | 33,7 | 2,9 |
|  | **Kattegat** | 7,5 | 20,3 | 5,4 | 0,2 |
|  | **Western Baltic** | 1,4 | 3,3 | 0,2 | 1,8 |

*Table V. Total crew costs (mill EUR) over the period 2012-2037 for the four fleet segments in each of the four scenarios. Crew costs are shown over all waters shown, together with crew costs in Kattegat and in the Western Baltic*

| **Scenario** | **Sea** | **TRA1215m** | **TRA1518m** | **TRA1824m** | **GKu12m** |
| --- | --- | --- | --- | --- | --- |
| **Scenario 1** | **Total** | 85,1 | 200,8 | 108,4 | 73,1 |
|  | **Kattegat** | 37,5 | 64,2 | 30,4 | 5,9 |
|  | **Western Baltic** | 17,9 | 40,2 | 2,2 | 45,3 |
| **Scenario 2** | **Total** | 85,2 | 200,7 | 108,4 | 73,1 |
|  | **Kattegat** | 37,5 | 64,2 | 30,4 | 5,9 |
|  | **Western Baltic** | 17,9 | 40,1 | 2,2 | 45,3 |
| **Scenario 3** | **Total** | 102,0 | 195,6 | 98,0 | 69,2 |
|  | **Kattegat** | 44,9 | 62,6 | 27,5 | 5,5 |
|  | **Western Baltic** | 21,4 | 39,1 | 2,0 | 42,9 |
| **Scenario 4** | **Total** | 89,6 | 198,6 | 108,7 | 71,1 |
|  | **Kattegat** | 39,4 | 63,6 | 30,4 | 5,7 |
|  | **Western Baltic** | 18,8 | 39,7 | 2,2 | 44,1 |

*Table W. Total variable costs (mill EUR) over the period 2012-2037 for the four fleet segments in each of the four scenarios. Variable costs are shown over all waters shown, together with variable costs in Kattegat and in the Western Baltic*

| **Scenario** | **Sea** | **TRA1215m** | **TRA1518m** | **TRA1824m** | **GKu12m** |
| --- | --- | --- | --- | --- | --- |
| **Scenario 1** | **Total** | 19,7 | 46,4 | 32,8 | 21,9 |
|  | **Kattegat** | 8,7 | 14,8 | 9,2 | 1,7 |
|  | **Western Baltic** | 4,1 | 9,3 | 0,7 | 13,6 |
| **Scenario 2** | **Total** | 19,8 | 46,3 | 32,8 | 21,9 |
|  | **Kattegat** | 8,7 | 14,8 | 9,2 | 1,7 |
|  | **Western Baltic** | 4,1 | 9,3 | 0,7 | 13,6 |
| **Scenario 3** | **Total** | 23,6 | 45,2 | 29,6 | 20,7 |
|  | **Kattegat** | 10,4 | 14,5 | 8,3 | 1,7 |
|  | **Western Baltic** | 5,0 | 9,0 | 0,6 | 12,8 |
| **Scenario 4** | **Total** | 20,8 | 45,9 | 32,9 | 21,3 |
|  | **Kattegat** | 9,1 | 14,7 | 9,2 | 1,7 |
|  | **Western Baltic** | 4,4 | 9,2 | 0,7 | 13,2 |

*Table X. Total capital costs (mill EUR) over the period 2012-2037 for the four fleet segments in each of the four scenarios. Capital costs are shown over all waters shown, together with capital costs in Kattegat and in the Western Baltic*

| **Scenario** | **Sea** | **TRA1215m** | **TRA1518m** | **TRA1824m** | **GKu12m** |
| --- | --- | --- | --- | --- | --- |
| **Scenario 1** | **Total** | 18,0 | 40,0 | 34,5 | 10,3 |
|  | **Kattegat** | 7,9 | 12,8 | 9,7 | 0,8 |
|  | **Western Baltic** | 3,8 | 8,0 | 0,7 | 6,4 |
| **Scenario 2** | **Total** | 18,0 | 40,0 | 34,5 | 10,3 |
|  | **Kattegat** | 7,9 | 12,8 | 9,7 | 0,8 |
|  | **Western Baltic** | 3,8 | 8,0 | 0,7 | 6,4 |
| **Scenario 3** | **Total** | 18,6 | 40,6 | 33,8 | 10,3 |
|  | **Kattegat** | 8,2 | 13,0 | 9,5 | 0,8 |
|  | **Western Baltic** | 3,9 | 8,1 | 0,7 | 6,4 |
| **Scenario 4** | **Total** | 18,1 | 40,5 | 34,8 | 10,3 |
|  | **Kattegat** | 8,0 | 12,9 | 9,7 | 0,8 |
|  | **Western Baltic** | 3,8 | 8,1 | 0,7 | 6,4 |

*Table Y. Total fixed costs (mill EUR) over the period 2012-2037 for the four fleet segments in each of the four scenarios. Fixed costs are shown over all waters shown, together with fixed costs in Kattegat and in the Western Baltic*

| **Scenario** | **Sea** | **TRA1215m** | **TRA1518m** | **TRA1824m** | **GKu12m** |
| --- | --- | --- | --- | --- | --- |
| **Scenario 1** | **Total** | 31,7 | 55,8 | 40,6 | 19,8 |
|  | **Kattegat** | 14,0 | 17,9 | 11,4 | 1,6 |
|  | **Western Baltic** | 6,7 | 11,2 | 0,8 | 12,3 |
| **Scenario 2** | **Total** | 31,7 | 55,8 | 40,6 | 19,8 |
|  | **Kattegat** | 14,0 | 17,9 | 11,4 | 1,6 |
|  | **Western Baltic** | 6,7 | 11,2 | 0,8 | 12,3 |
| **Scenario 3** | **Total** | 33,0 | 56,8 | 39,8 | 19,8 |
|  | **Kattegat** | 14,5 | 18,2 | 11,1 | 1,6 |
|  | **Western Baltic** | 6,9 | 11,4 | 0,8 | 12,3 |
| **Scenario 4** | **Total** | 32,0 | 56,5 | 40,9 | 19,8 |
|  | **Kattegat** | 14,1 | 18,1 | 11,4 | 1,6 |
|  | **Western Baltic** | 6,7 | 11,3 | 0,8 | 12,3 |

*Table Z. Total profits (1000 EUR) (mill EUR) over the period 2012-2037 for the four fleet segments in each of the four scenarios. ´Profits are shown over all waters shown, together with profit in Kattegat and in the Western Baltic*

| **Scenario** | **Sea** | **TRA1215m** | **TRA1518m** | **TRA1824m** | **GKu12m** |
| --- | --- | --- | --- | --- | --- |
| **Scenario 1** | **Total** | 50,3 | 158,0 | 77,1 | 19,1 |
|  | **Kattegat** | 20,5 | 43,8 | 25,4 | 1,6 |
|  | **Western Baltic** | 11,7 | 36,4 | 2,0 | 11,9 |
| **Scenario 2** | **Total** | 50,3 | 158,0 | 77,2 | 19,1 |
|  | **Kattegat** | 20,5 | 43,8 | 25,4 | 1,6 |
|  | **Western Baltic** | 11,7 | 36,4 | 2,0 | 11,9 |
| **Scenario 3** | **Total** | 65,2 | 147,2 | 64,4 | 16,7 |
|  | **Kattegat** | 26,4 | 40,0 | 21,4 | 1,4 |
|  | **Western Baltic** | 15,4 | 34,4 | 1,7 | 10,4 |
| **Scenario 4** | **Total** | 54,2 | 152,3 | 75,1 | 17,9 |
|  | **Kattegat** | 22,0 | 41,7 | 25,1 | 1,5 |
|  | **Western Baltic** | 12,7 | 35,4 | 2,0 | 11,2 |

**References**

Arnason R. Economic instruments for achieving ecosystem objectives in fisheries management. ICES Journal of Marine Science: Journal du Conseil. 2000; 57(3): 742-51.

Audzijonyte A, Gorton R, Kaplan I, Fulton EA. Atlantis User’s Guide Part I: General Overview, Physics & Ecology. CSIRO living document; 2017; Available from: <https://research.csiro.au/atlantis/home/useful-references/>

Bastardie F, Nielsen JR, Miethe T. DISPLACE: a Dynamic, individual-based model for spatial fishing planning and effort displacement – integrating underlying fish population models. Can. J. Fish. Aquat. Sci. 2014; 71: 1-21. doi: 10.1139/cjfas-2013-0126.

Berg P, Poulsen JW. Implementation details for HBM. DMI Technical Report 12-11, DMI, Copenhagen, pp. 147; 2012.

Bubinas A, Ložys L. The Nutrition of Fish in the Curonian Lagoon and the Coastal Zone of the Baltic Sea. Acta Zoologica Lituanica. 2000; 10(4): 56-67.

Cobb CW, Douglas PH. A theory of production. The American Economic Review. 1928; 18(1): 139-65.

Conkright ME, Levitus S. World Ocean Atlas 2001. Volume 4, Nutrients. 2002.

Deutsch B, Forster S, Wilhelm M, Dippner JW, Voss M. Denitrification in sediments as a major nitrogen sink in the Baltic Sea: an extrapolation using sediment characteristics. Biogeosciences. 2010; 7(10): 3259-71.

Dick S, Kleine E, Mueller-Navarra S, Kleine H, Komo H. The operational circulation model of BSH (BSHcmod) - model description and validation. Berichte des BSH 29/2001Bundesamt für Seeschifffart und Hydrographie. 2001: 241.

Ehrenberg SZ, Hansson S, Elmgren R. Sublittoral abundance and food consumption of Baltic gobies. Journal of Fish Biology. 2005; 67(4): 1083-1093.

Eide A, Skjold F, Olsen F, Flaaten O. Harvest functions: the Norwegian bottom trawl cod fisheries. Marine Resource Economics. 2003: 81-93.

Frost H, Andersen P, Hoff A. An Application of Fisheries Economic Theory—100 Years after Warming’s Paper:“Rent of Fishing Grounds.”. Nationaløkonomisk Tidsskrift (Danish Economic Journal). 2011; 149: 55-84.

Garza-Gil MD, Varela-Lafuente MM, Iglesias-Malvido C. Spain's North Atlantic swordfish fishery. Marine Policy. 2003; 27(1): 31-7.

Gordon HS. The economic theory of a common-property resource: the fishery. In Classic Papers in Natural Resource Economics. Palgrave Macmillan UK. 1954: 178-203.

Hammond PS, Macleod K, Berggren P, Borchers DL, Burt L, Caсadas A, et al. Cetacean abundance and distribution in European Atlantic shelf waters to inform conservation and management. Biological Conservation. 2013; 164: 107-122.

Hausman DM. Economic methodology in a nutshell. The Journal of Economic Perspectives. 1989; 3(2): 115-27.

Härkönen T, Brasseur S, Teilmann J, Vincent C, Dietz R, Abt K, Reijnders P. Status of grey seals along mainland Europe from the Southwestern Baltic to France. NAMMCO Scientific Publications. 2007; 6: 57-68.

HELCOM. Guidelines for coastal fish monitoring sampling methods of HELCOM. 2017a. Available from: <http://www.helcom.fi/action-areas/monitoring-and-assessment/manuals-and-guidelines/coastal-fish-guidelines>

HELCOM. Abundance of coastal key fish species. HELCOM core indicator report. 2017b. Available from: <http://www.helcom.fi/baltic-sea-trends/indicators/abundance-of-key-coastal-fish-species>

Hentati-Sundberg J. Arctic Seabirds Breeding in the African-Eurasian Waterbird Agreement (AEWA) Area: Status and Trends. 2011. Available from: <http://library.arcticportal.org/id/eprint/1600>

ICES. Stock Summaries Advice June 2012 – North Sea, Cod in Division IIIa (Kattegat), Advice for 2013. 2012.

ICES. Baltic Sea, Cod in Subdivision 22-24 (Western Baltic Sea), Advice May 2013. 2013a.

ICES. North Sea, Sprat in Division IIIa (Skagerrak – Kattegat), Advice May 2013. 2013b.

ICES. North Sea, Herring in Division IIIA and Subdivision 22-24 (western Baltic spring spawners), Advice May 2013. 2013c.

ICES. North Sea, Whiting in Division IIIa (Skagerrak – Kattegat), Advice June 2013. 2013d.

ICES. Baltic Sea, Plaice in Subdivision 21-23 (Kattegat, Belts and Sound), Advice 2013. 2013e.

ICES. ICES WGNSSK report 2013: 3. Nephrops in Subareas IIIa and IV, 2013f.

Johns D. Monthly Averaged Data for Zoopankton (48–63N, 10W–12E) 1946-2007 as Recorded by the Continuous Plankton Recorder. Sir Alister Hardy Foundation for Ocean Science, Plymouth, UK. 2009.

Johnson MP, Lordan C, Power AM. Chapter Two - Habitat and Ecology of Nephrops norvegicus , in Magnus L. Johnson & Mark P. Johnson, ed.,'The Ecology and Biology of Nephrops norvegicus, Academic Press. 2013: 27-63.

Kauhala K, Ahola MP, Kunnasranta M. Demographic structure and mortality rate of a Baltic grey seal population at different stages of population change, judged on the basis of the hunting bag in Finland. Annales Zoologici Fennici. 2012: 287-305.

Koschinski S. Current knowledge on harbour porpoises (Phocoena phocoena) in the Baltic Sea. Ophelia. 2001; 55(3): 167-197.

Kronbak LG, Lindroos M. Sharing rules and stability in coalition games with externalities: the case of the Baltic Sea cod fishery. Environmental Economics. 2005; 22: 137-54.

Lappalainen A, Rask M, Koponen H, Vesala S. Relative abundance, diet and growth of perch(Perca fluviatilis) and roach(Rutilus rutilus) at Tvaerminne, northern Baltic Sea, in 1975 and 1997: responses to eutrophication? Boreal Environment Research. 2001; 6(2): 107-118.

Lewy P, Vinther M. A stochastic age-length-structured multispecies model applied to North Sea stocks. ICES CM. 2004: 33.

Lundström K, Hjerne O, Lunneryd SV, Karlsson O. Understanding the diet composition of marine mammals: grey seals (Halichoerus grypus) in the Baltic Sea. ICES Journal of Marine Science, 2010; 67(6): 1230–1239. doi: 10.1093/icesjms/fsq022.

Maar M, Møller EF, Larsen J, Madsen KS, Wan Z, She J, et al. Ecosystem modelling across a salinity gradient from the North Sea to the Baltic Sea. Ecological Modelling. 2011; 222(10): 1696-711.

Maar M, Møller EF, Gürkan Z, Jónasdóttir SH, Nielsen TG. Sensitivity of Calanus spp. copepods to environmental changes in the North Sea using life-stage structured models. Progress in Oceanography. 2012; 111: 24-37.

Maar M, Markager SS, Madsen KS, Windolf J, Lyngsgaard MM, Andersen HE, et al. The importance of local versus external nutrient loads for Chl a and primary production in the Western Baltic Sea. Ecological Modelling. 2016; 320: 258-272.

Möllmann C, Kornilovs G, Fetter M, Köster FW. Feeding ecology of central Baltic Sea herring and sprat. Journal of Fish Biology. 2004; 65(6): 1563-1581.

Morel A, Berthon JF. Surface pigments, algal biomass profiles, and potential production of the euphotic layer: Relationships reinvestigated in view of remote‐sensing applications. Limnology and oceanography. 1989; 34(8): 1545-1562.

Peltonen H, Vinni M, Lappalainen A, Pönni J. Spatial feeding patterns of herring (Clupea harengus L.), sprat (Sprattus sprattus L.), and the three-spined stickleback (Gasterosteus aculeatus L.) in the Gulf of Finland, Baltic Sea. ICES Journal of Marine Science: Journal du Conseil. 2004; 61(6): 966-971.

Salz P, Buisman E, Frost H, Accadia P, Prellezo R, Soma K. Study on the remuneration of spawning stock biomass. Final Report, Framian. 2010.

Sass BH, Nielsen NW, Jørgensen JW, Amstrup B, Kmit M, Mogensen KS. The operational DMI+HIRLAM model system+2002 version. In: DMI Technical Reports 02-05. Danish Meteorological Institute, Copenhagen, pp. 58; 2002.

Savchuk OP, Wulff, FV. Long-term modeling of large-scale nutrient cycles in the entire Baltic Sea. Hydrobiologia. 2009; 629:209–224; doi: 10.1007/s10750-009-9775-z.

Savchuk OP, Gustafsson BG, Rodriguez Medina M, Sokolov AV, Wulff FV. External nutrient loads to the Baltic Sea, Technical Report No. 5, Baltic Nest Institute; 2012.

Scott A. The fishery: the objectives of sole ownership. The Journal of Political Economy. 1955 Apr 1: 116-24.

She J, Berg P, Berg J. Bathymetry impacts on water exchange modelling through the Danish Straits. Journal of Marine Systems. 2007; 65: 450-459.

Tomczak MT, Müller-Karulis B, Järv L, Kotta J, Martin G, Minde A et al. Analysis of trophic networks and carbon flows in south-eastern Baltic coastal ecosystems. Progress in Oceanography. 2009; 81(1): 111-31.

Tomczak MT, Niiranen S, Hjerne O, Blenckner T. Ecosystem flow dynamics in the Baltic Proper—Using a multi-trophic dataset as a basis for food–web modelling. Ecological Modelling. 2012; 230: 123-47.

Törnlund L. Consumption of fish by Baltic seabirds. Master's thesis, Dep. of System Ecology, Stockholm University. 2013.

Vanhatalo J, Vetemaa M, Herrero A, Aho T, Tiilikainen R. By-Catch of Grey Seals (Halichoerus grypus) in Baltic Fisheries—A Bayesian Analysis of Interview Survey. PLoS ONE. 2014; 9(11): e113836. doi: 10.1371/journal.pone.0113836.

Vichi M, Ruardij P, Baretta J. Link or sink: a modelling interpretation of the open Baltic biogeochemistry. Biogeosciences Discussions. 2004; 1(1): 219-274.

Wan Z, She J, Maar M, Jonasson L, Baasch-Larsen J. Assessment of a physical-biogeochemical coupled model system for operational service in the Baltic Sea. Ocean Science. 2012; 8(4): 683.

1. The majority of the 2012 TSBs displayed in Table J are estimates based on ICES stock assessment data [74; 77; 79; 112-114] for larger stock areas than the Kattegat and the Western Baltic, as stock estimates for these areas alone in most cases do not exist. Stock sizes have been scaled according to relative subarea sizes compared to the larger stock distribution and assessment areas (to match how initial stock biomasses in Atlantis have been re-distributed according to subareas). The natural mortalities and target fishing mortalities displayed in Table J are all based on ICES Working group estimates [74; 77; 79; 112-114], and, as for the TSB values, are for most species groups based on estimates for areas larger than Kattegat and the Western Baltic. [↑](#footnote-ref-1)
2. In the present context there will only be high-grading discard of fish below minimum landings size, and no overquota discards, as it is, given the maximization of the NPV, assumed that the fishers comply with the Danish quotas. [↑](#footnote-ref-2)
