# Supplementary figures and images for: The Baltic Sea Atlantis: An integrated end-to-end modelling framework evaluating ecosystem-wide effects of human-induced pressures
Source: PLoS One. 2018 Jul 20;13(7):e0199168. doi: 10.1371/journal.pone.0199168 (PMC6054375; doi:10.1371/journal.pone.0199168)

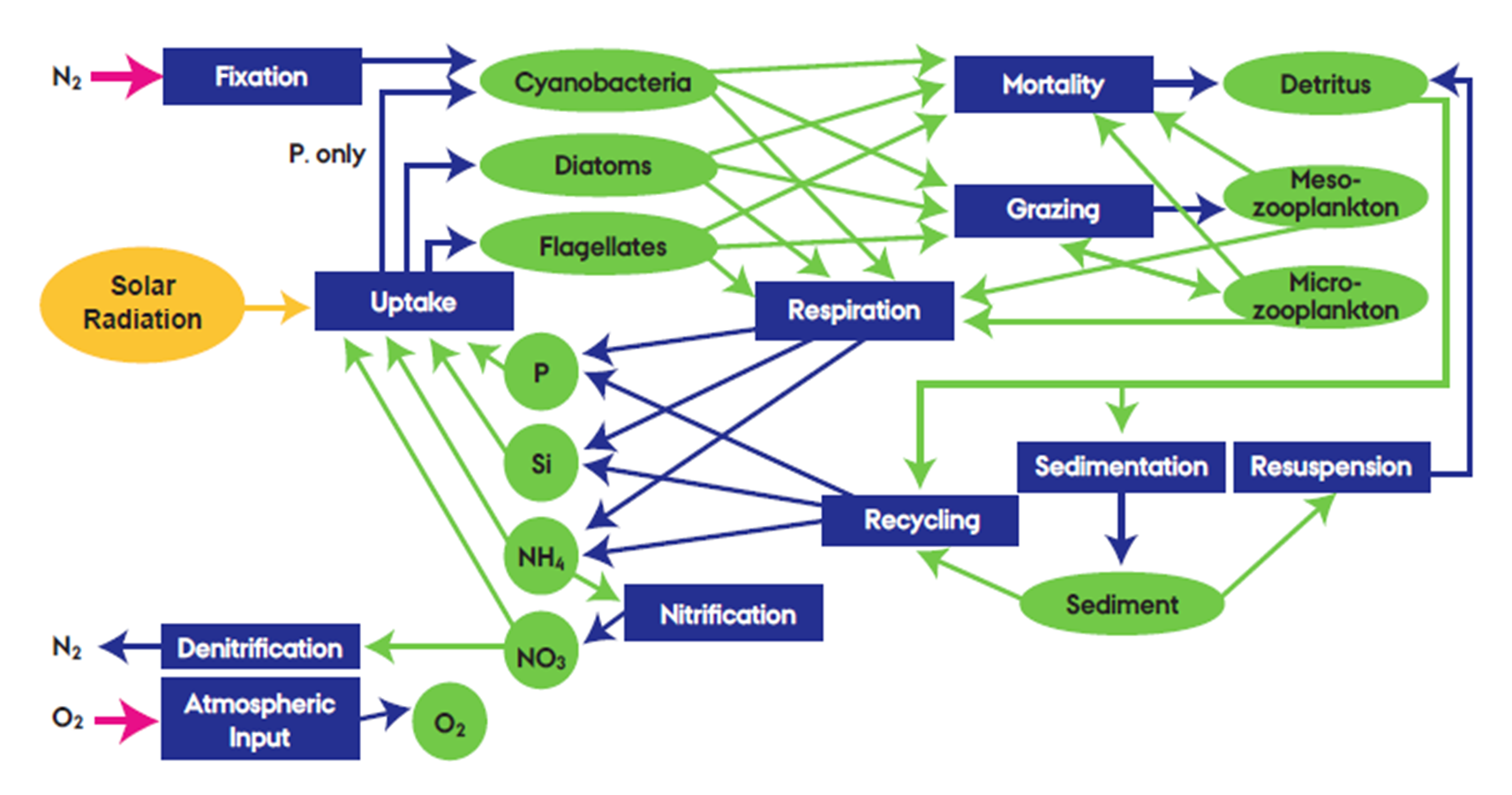

Supplement: S1 File — Supporting Information A. The Baltic Sea Atlantis: File A. Input data of the tracers per box for the Baltic Atlantis File B. Input data of the tracers per box and layer for the Baltic Atlantis File C. Input data for the fill values for the tracers for the Baltic Atlantis Figure A. Schematic diagram illustrating the structure of the coupled HBM-ERGOM model system Figure B. 120 year simulation run Figure C. The FISHRENT model diagram, here applied to Kattegat and Western Baltic. Figure D. One-year cycle of Chl-a in the different polygons Figure E. Relative biomass–initial condition values compared with simulation outcome Figure F. Diet composition of all predators Figure G. Biomass per age group over time for all vertebrates Figure H. Demography distribution for all vertebrates—the number of individuals for each age group Figure I. Geographical distribution of all functional groups Figure J. Geographical distribution of oxygen in the different layers. Panel 1 = top layer, panel 7 = bottom layer Figure K. Total biomass of Cod for scenario 1 (baseline) compared to scenario 5 Figure L. Relative prey biomass for predator cod, baseline compared to scenario 5 Figure M. One-year cycle of nutrients in the different polygons Table A. Physical and geochemical parameters used to internally force the Baltic Atlantis model. Table B. Summary of riverine + direct point source waterborne nitrogen loads applied to the Baltic Atlantis grid based on information from the Review of the Fifth Baltic Sea Pollution Load Compilation for the 2013 HELCOM Ministerial Meeting (HELCOM PLC-5.5). Nitrogen fractionation between DIN and DON based on Savchuk et al. (2012). Bioavailable fraction of DON assumed equal to labile DON as in Savchuk and Wolff (2009). Coastal retention fractions from Savchuk and Wolff (2009). Table C. Summary of key sources used to inform the biological module of Baltic Atlantis in relation to abundance and biomass, demography, prey-predator interaction and other functions. Ta [file pone.0199168.s001.zip › FigA.tif]

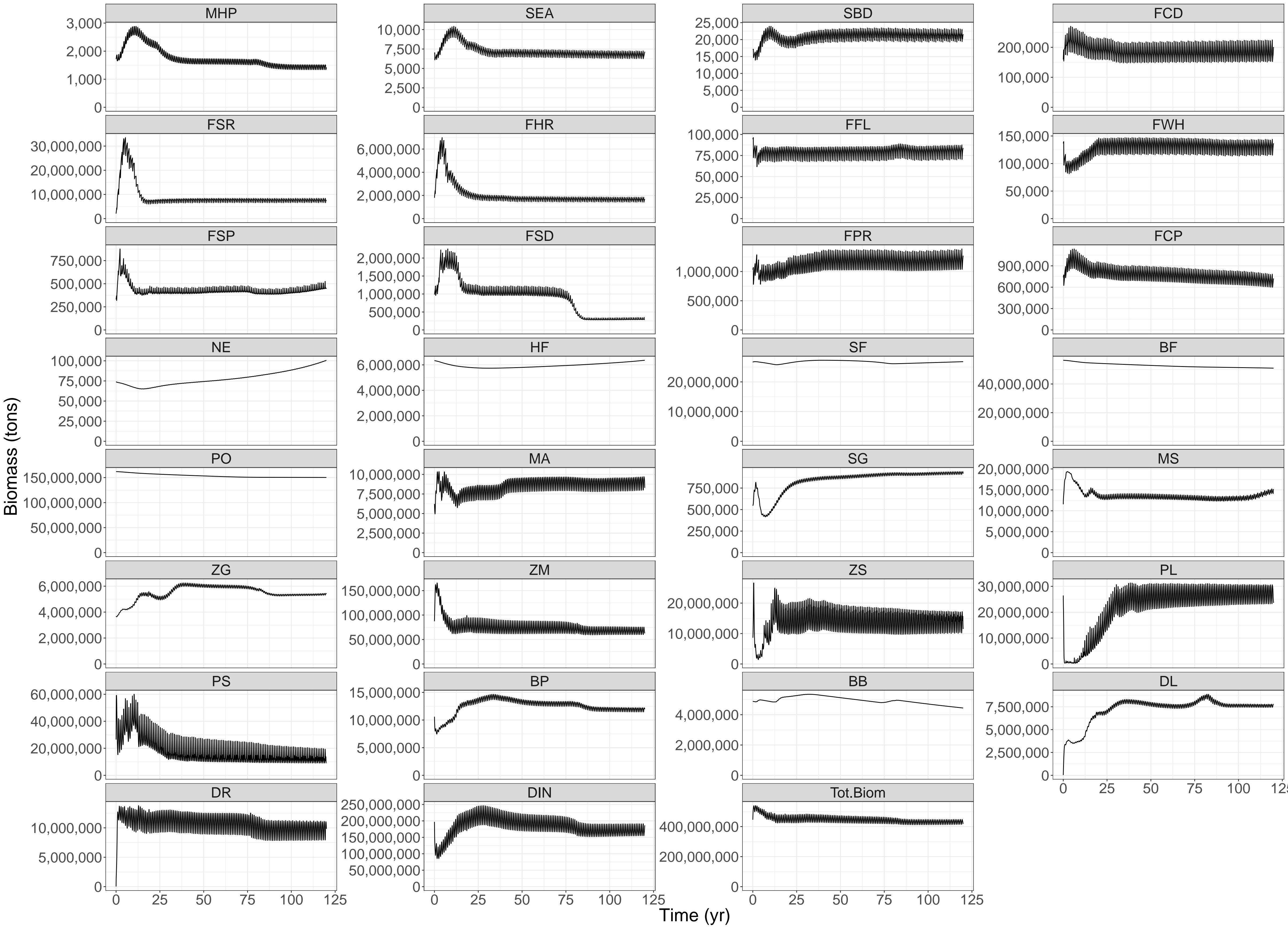

Supplement: S1 File — Supporting Information A. The Baltic Sea Atlantis: File A. Input data of the tracers per box for the Baltic Atlantis File B. Input data of the tracers per box and layer for the Baltic Atlantis File C. Input data for the fill values for the tracers for the Baltic Atlantis Figure A. Schematic diagram illustrating the structure of the coupled HBM-ERGOM model system Figure B. 120 year simulation run Figure C. The FISHRENT model diagram, here applied to Kattegat and Western Baltic. Figure D. One-year cycle of Chl-a in the different polygons Figure E. Relative biomass–initial condition values compared with simulation outcome Figure F. Diet composition of all predators Figure G. Biomass per age group over time for all vertebrates Figure H. Demography distribution for all vertebrates—the number of individuals for each age group Figure I. Geographical distribution of all functional groups Figure J. Geographical distribution of oxygen in the different layers. Panel 1 = top layer, panel 7 = bottom layer Figure K. Total biomass of Cod for scenario 1 (baseline) compared to scenario 5 Figure L. Relative prey biomass for predator cod, baseline compared to scenario 5 Figure M. One-year cycle of nutrients in the different polygons Table A. Physical and geochemical parameters used to internally force the Baltic Atlantis model. Table B. Summary of riverine + direct point source waterborne nitrogen loads applied to the Baltic Atlantis grid based on information from the Review of the Fifth Baltic Sea Pollution Load Compilation for the 2013 HELCOM Ministerial Meeting (HELCOM PLC-5.5). Nitrogen fractionation between DIN and DON based on Savchuk et al. (2012). Bioavailable fraction of DON assumed equal to labile DON as in Savchuk and Wolff (2009). Coastal retention fractions from Savchuk and Wolff (2009). Table C. Summary of key sources used to inform the biological module of Baltic Atlantis in relation to abundance and biomass, demography, prey-predator interaction and other functions. Ta [file pone.0199168.s001.zip › FigB.pdf]

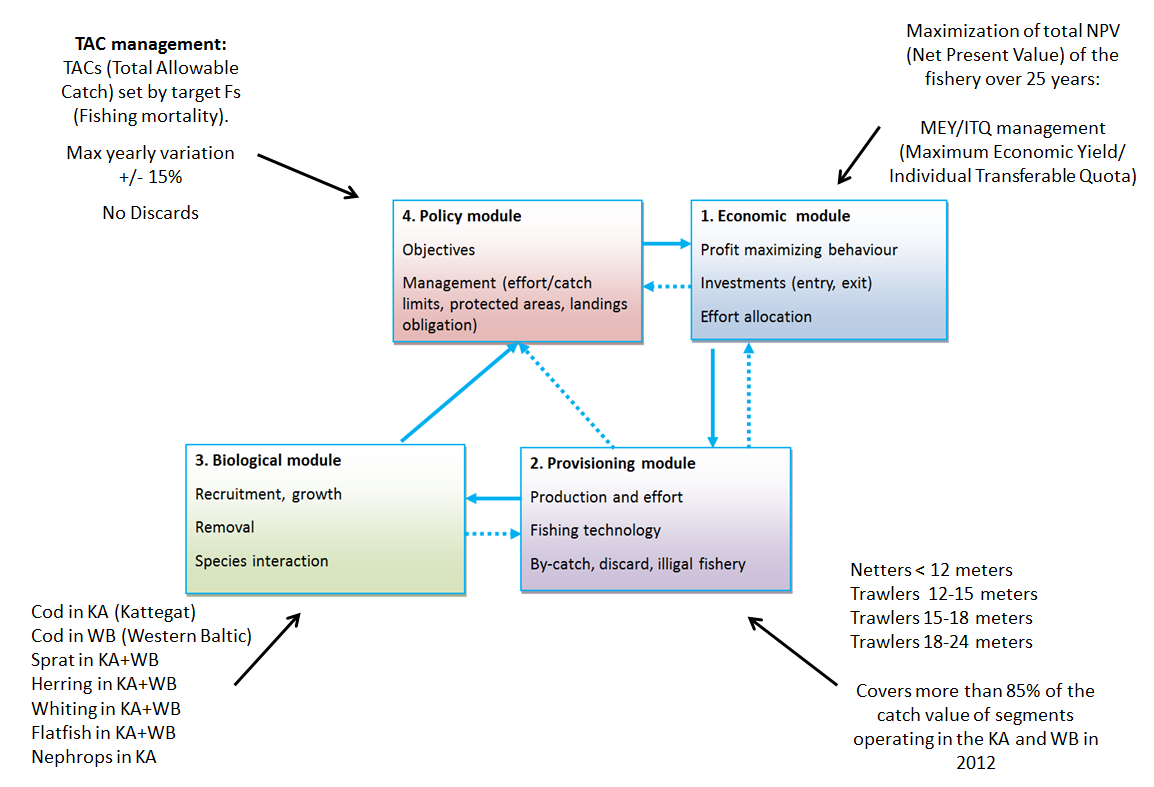

Supplement: S1 File — Supporting Information A. The Baltic Sea Atlantis: File A. Input data of the tracers per box for the Baltic Atlantis File B. Input data of the tracers per box and layer for the Baltic Atlantis File C. Input data for the fill values for the tracers for the Baltic Atlantis Figure A. Schematic diagram illustrating the structure of the coupled HBM-ERGOM model system Figure B. 120 year simulation run Figure C. The FISHRENT model diagram, here applied to Kattegat and Western Baltic. Figure D. One-year cycle of Chl-a in the different polygons Figure E. Relative biomass–initial condition values compared with simulation outcome Figure F. Diet composition of all predators Figure G. Biomass per age group over time for all vertebrates Figure H. Demography distribution for all vertebrates—the number of individuals for each age group Figure I. Geographical distribution of all functional groups Figure J. Geographical distribution of oxygen in the different layers. Panel 1 = top layer, panel 7 = bottom layer Figure K. Total biomass of Cod for scenario 1 (baseline) compared to scenario 5 Figure L. Relative prey biomass for predator cod, baseline compared to scenario 5 Figure M. One-year cycle of nutrients in the different polygons Table A. Physical and geochemical parameters used to internally force the Baltic Atlantis model. Table B. Summary of riverine + direct point source waterborne nitrogen loads applied to the Baltic Atlantis grid based on information from the Review of the Fifth Baltic Sea Pollution Load Compilation for the 2013 HELCOM Ministerial Meeting (HELCOM PLC-5.5). Nitrogen fractionation between DIN and DON based on Savchuk et al. (2012). Bioavailable fraction of DON assumed equal to labile DON as in Savchuk and Wolff (2009). Coastal retention fractions from Savchuk and Wolff (2009). Table C. Summary of key sources used to inform the biological module of Baltic Atlantis in relation to abundance and biomass, demography, prey-predator interaction and other functions. Ta [file pone.0199168.s001.zip › FigC.tif]

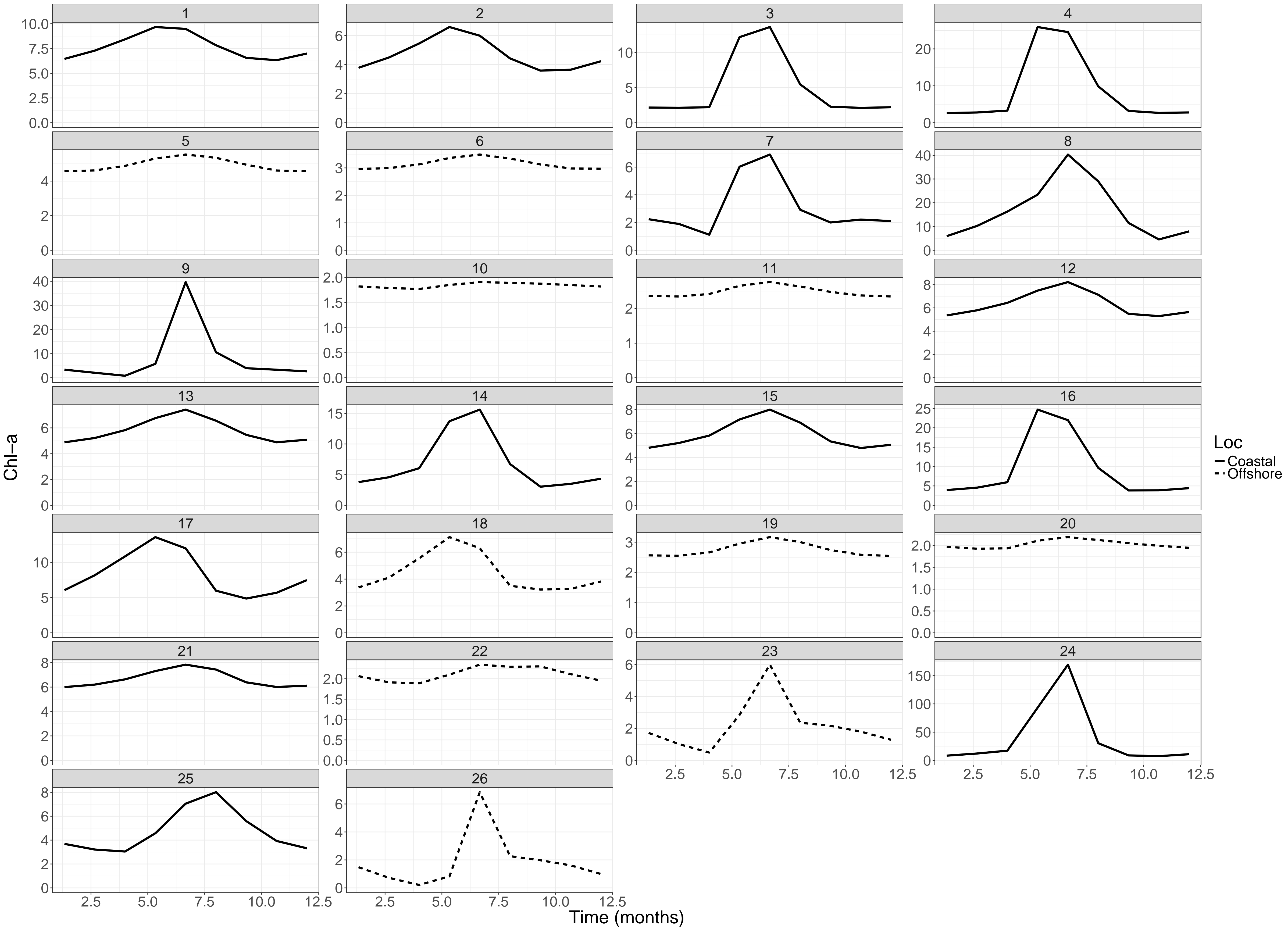

Supplement: S1 File — Supporting Information A. The Baltic Sea Atlantis: File A. Input data of the tracers per box for the Baltic Atlantis File B. Input data of the tracers per box and layer for the Baltic Atlantis File C. Input data for the fill values for the tracers for the Baltic Atlantis Figure A. Schematic diagram illustrating the structure of the coupled HBM-ERGOM model system Figure B. 120 year simulation run Figure C. The FISHRENT model diagram, here applied to Kattegat and Western Baltic. Figure D. One-year cycle of Chl-a in the different polygons Figure E. Relative biomass–initial condition values compared with simulation outcome Figure F. Diet composition of all predators Figure G. Biomass per age group over time for all vertebrates Figure H. Demography distribution for all vertebrates—the number of individuals for each age group Figure I. Geographical distribution of all functional groups Figure J. Geographical distribution of oxygen in the different layers. Panel 1 = top layer, panel 7 = bottom layer Figure K. Total biomass of Cod for scenario 1 (baseline) compared to scenario 5 Figure L. Relative prey biomass for predator cod, baseline compared to scenario 5 Figure M. One-year cycle of nutrients in the different polygons Table A. Physical and geochemical parameters used to internally force the Baltic Atlantis model. Table B. Summary of riverine + direct point source waterborne nitrogen loads applied to the Baltic Atlantis grid based on information from the Review of the Fifth Baltic Sea Pollution Load Compilation for the 2013 HELCOM Ministerial Meeting (HELCOM PLC-5.5). Nitrogen fractionation between DIN and DON based on Savchuk et al. (2012). Bioavailable fraction of DON assumed equal to labile DON as in Savchuk and Wolff (2009). Coastal retention fractions from Savchuk and Wolff (2009). Table C. Summary of key sources used to inform the biological module of Baltic Atlantis in relation to abundance and biomass, demography, prey-predator interaction and other functions. Ta [file pone.0199168.s001.zip › FigD.pdf]

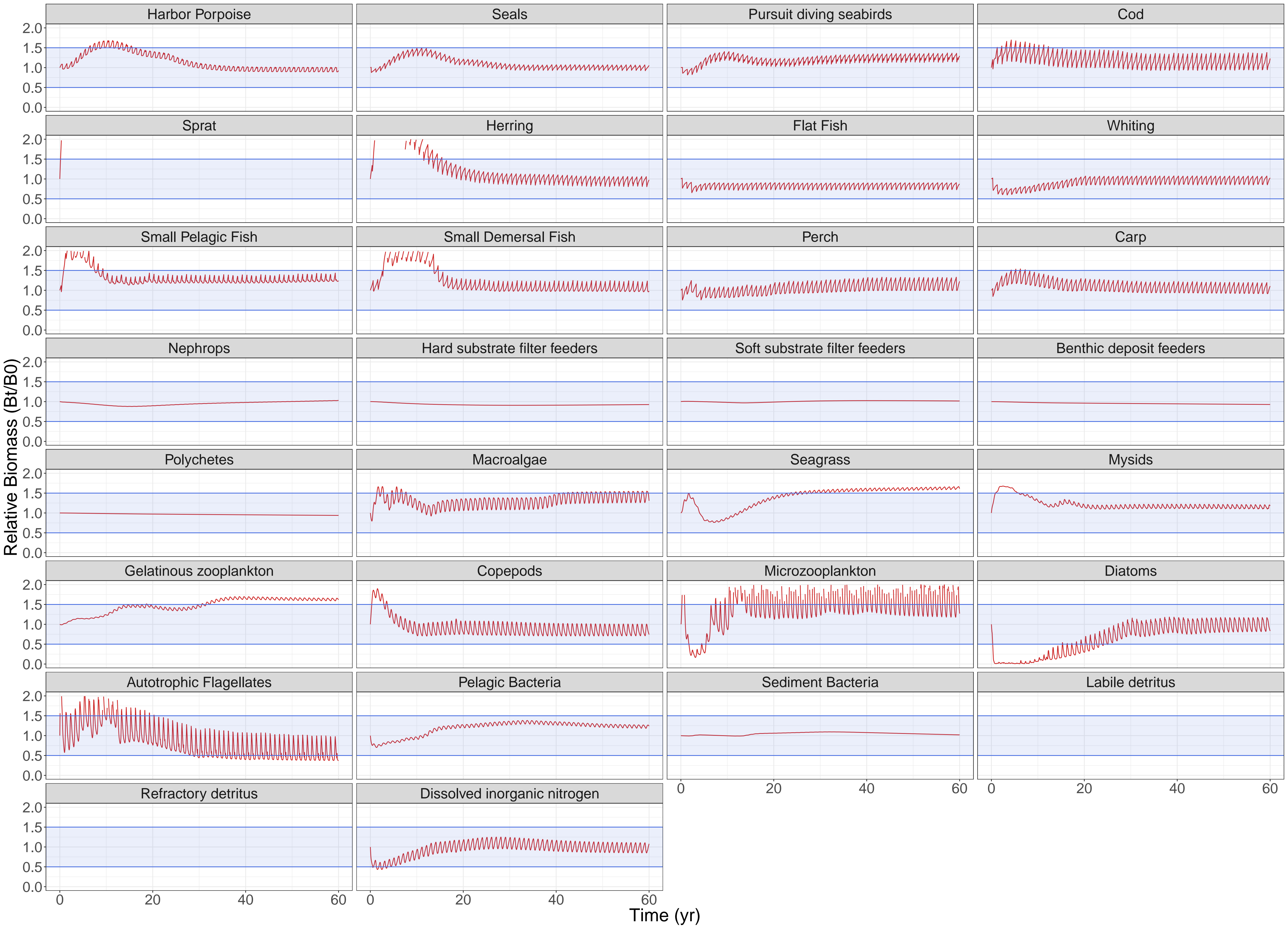

Supplement: S1 File — Supporting Information A. The Baltic Sea Atlantis: File A. Input data of the tracers per box for the Baltic Atlantis File B. Input data of the tracers per box and layer for the Baltic Atlantis File C. Input data for the fill values for the tracers for the Baltic Atlantis Figure A. Schematic diagram illustrating the structure of the coupled HBM-ERGOM model system Figure B. 120 year simulation run Figure C. The FISHRENT model diagram, here applied to Kattegat and Western Baltic. Figure D. One-year cycle of Chl-a in the different polygons Figure E. Relative biomass–initial condition values compared with simulation outcome Figure F. Diet composition of all predators Figure G. Biomass per age group over time for all vertebrates Figure H. Demography distribution for all vertebrates—the number of individuals for each age group Figure I. Geographical distribution of all functional groups Figure J. Geographical distribution of oxygen in the different layers. Panel 1 = top layer, panel 7 = bottom layer Figure K. Total biomass of Cod for scenario 1 (baseline) compared to scenario 5 Figure L. Relative prey biomass for predator cod, baseline compared to scenario 5 Figure M. One-year cycle of nutrients in the different polygons Table A. Physical and geochemical parameters used to internally force the Baltic Atlantis model. Table B. Summary of riverine + direct point source waterborne nitrogen loads applied to the Baltic Atlantis grid based on information from the Review of the Fifth Baltic Sea Pollution Load Compilation for the 2013 HELCOM Ministerial Meeting (HELCOM PLC-5.5). Nitrogen fractionation between DIN and DON based on Savchuk et al. (2012). Bioavailable fraction of DON assumed equal to labile DON as in Savchuk and Wolff (2009). Coastal retention fractions from Savchuk and Wolff (2009). Table C. Summary of key sources used to inform the biological module of Baltic Atlantis in relation to abundance and biomass, demography, prey-predator interaction and other functions. Ta [file pone.0199168.s001.zip › FigE.pdf]

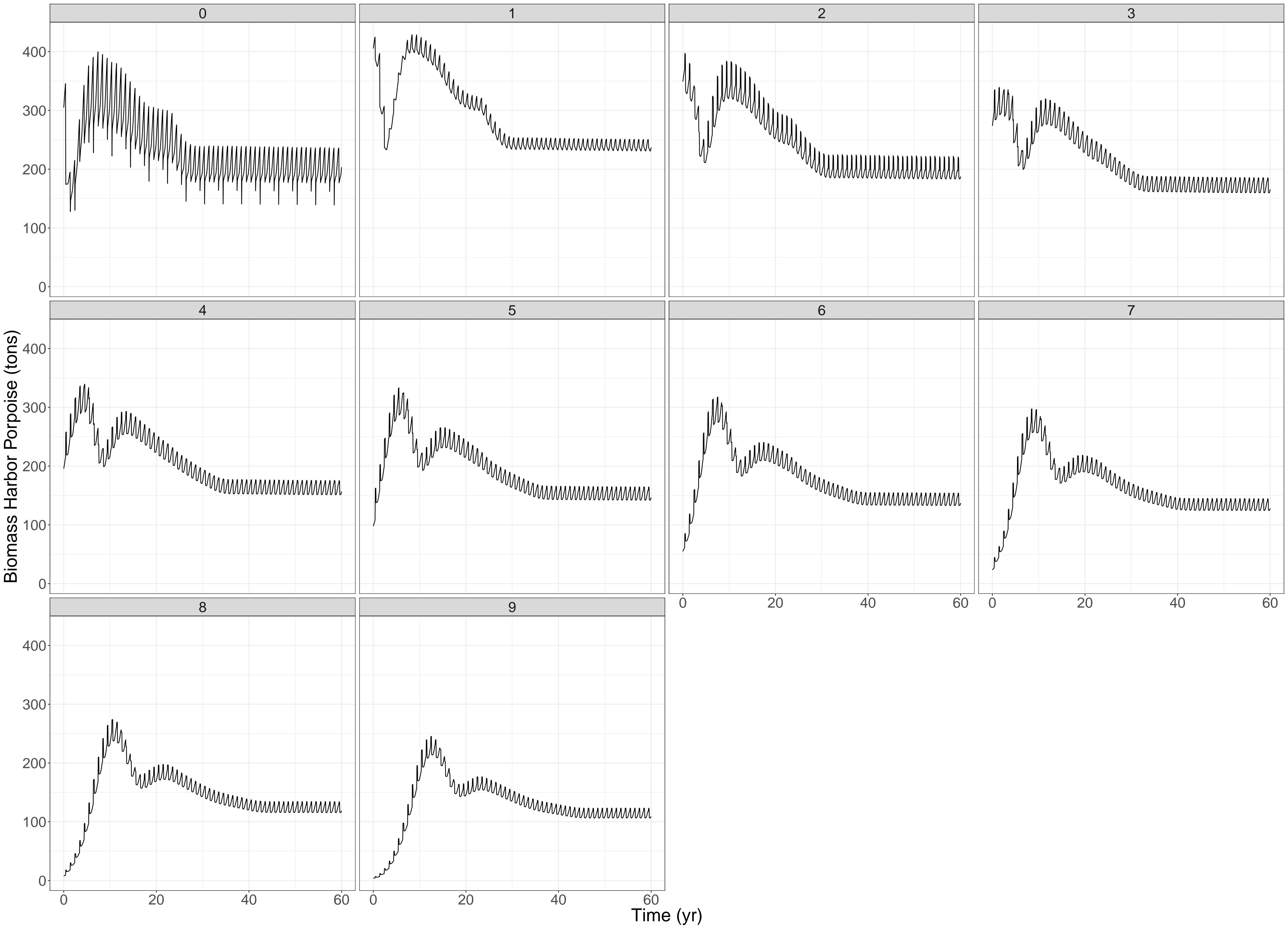

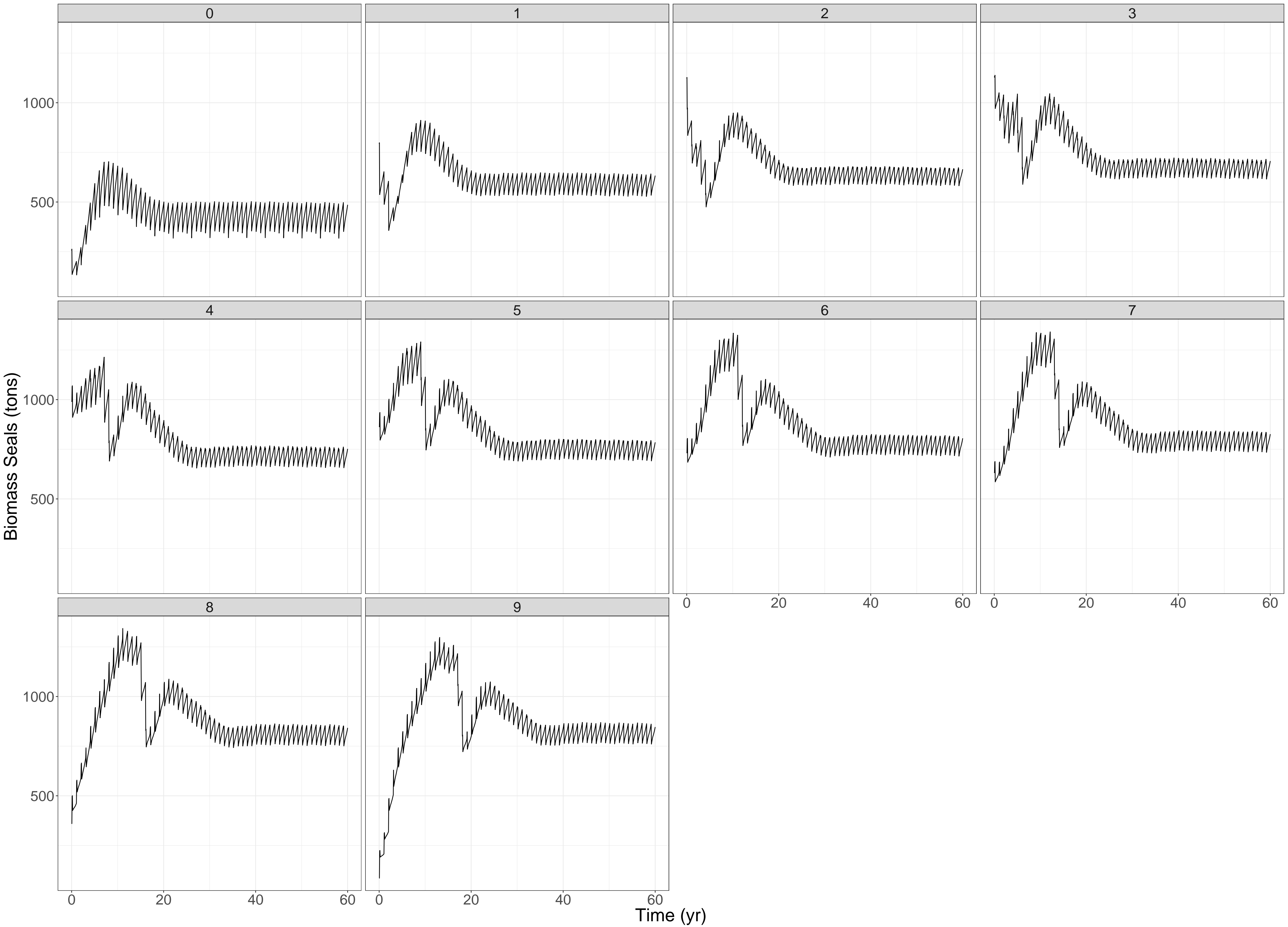

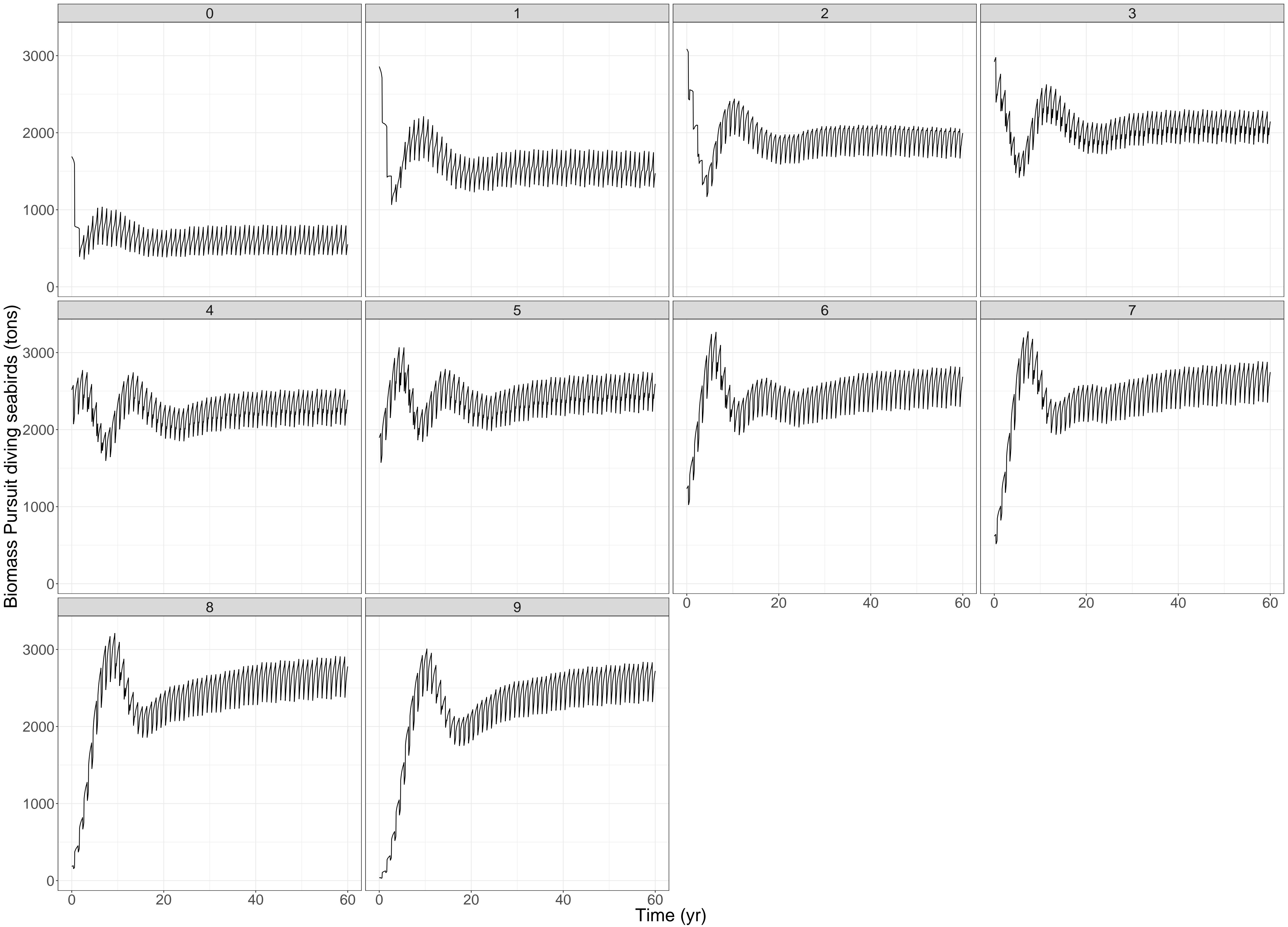

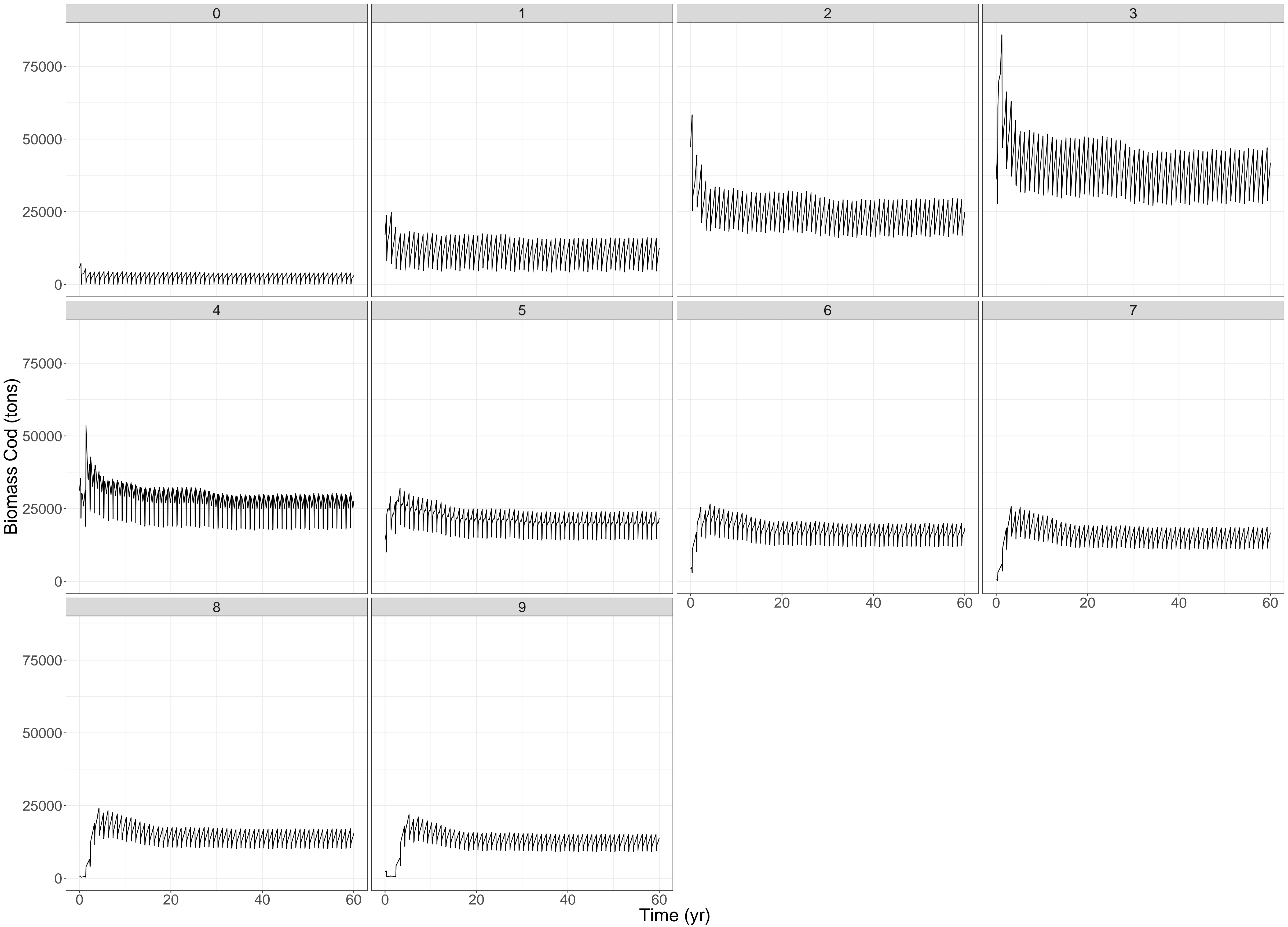

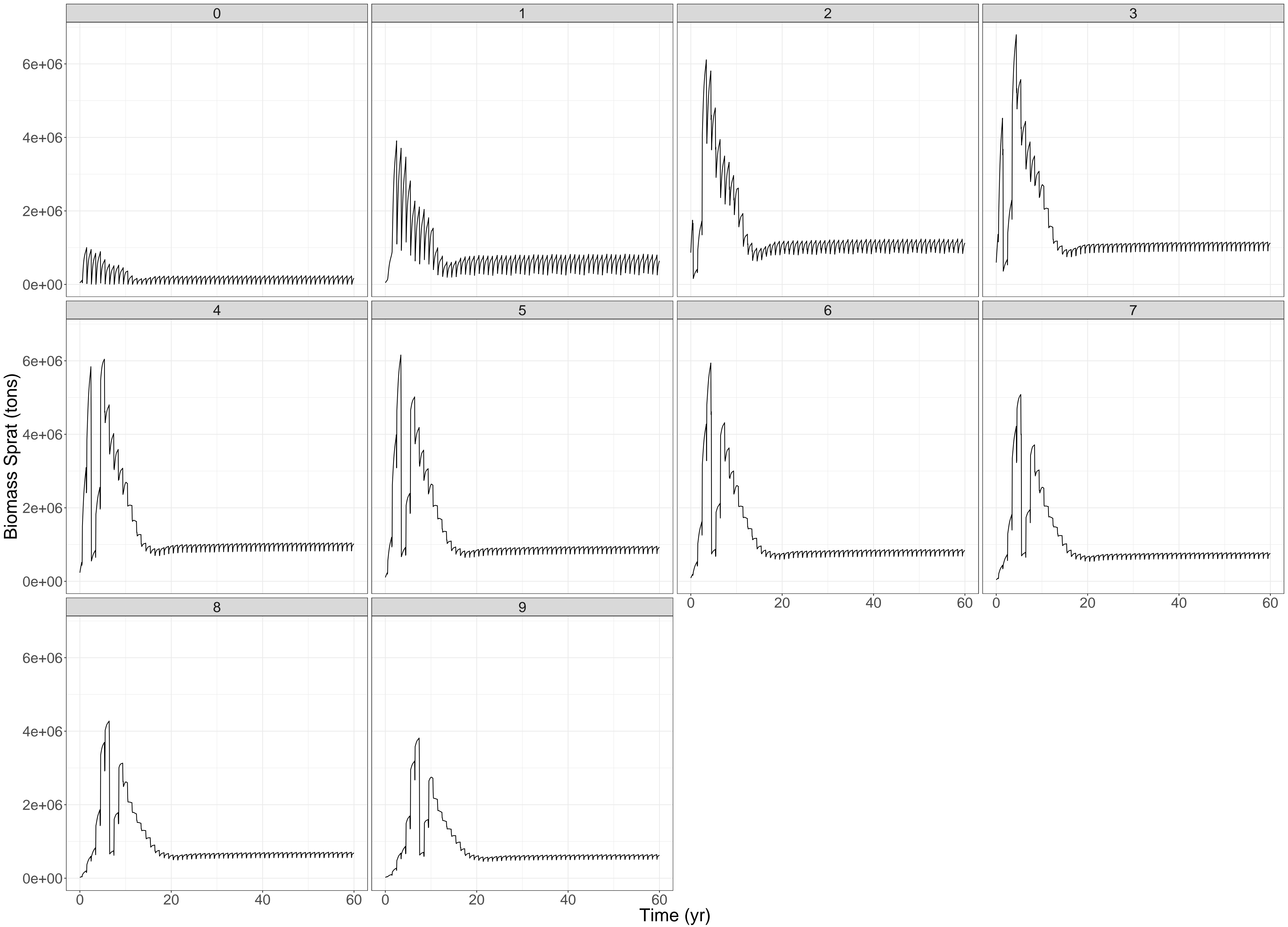

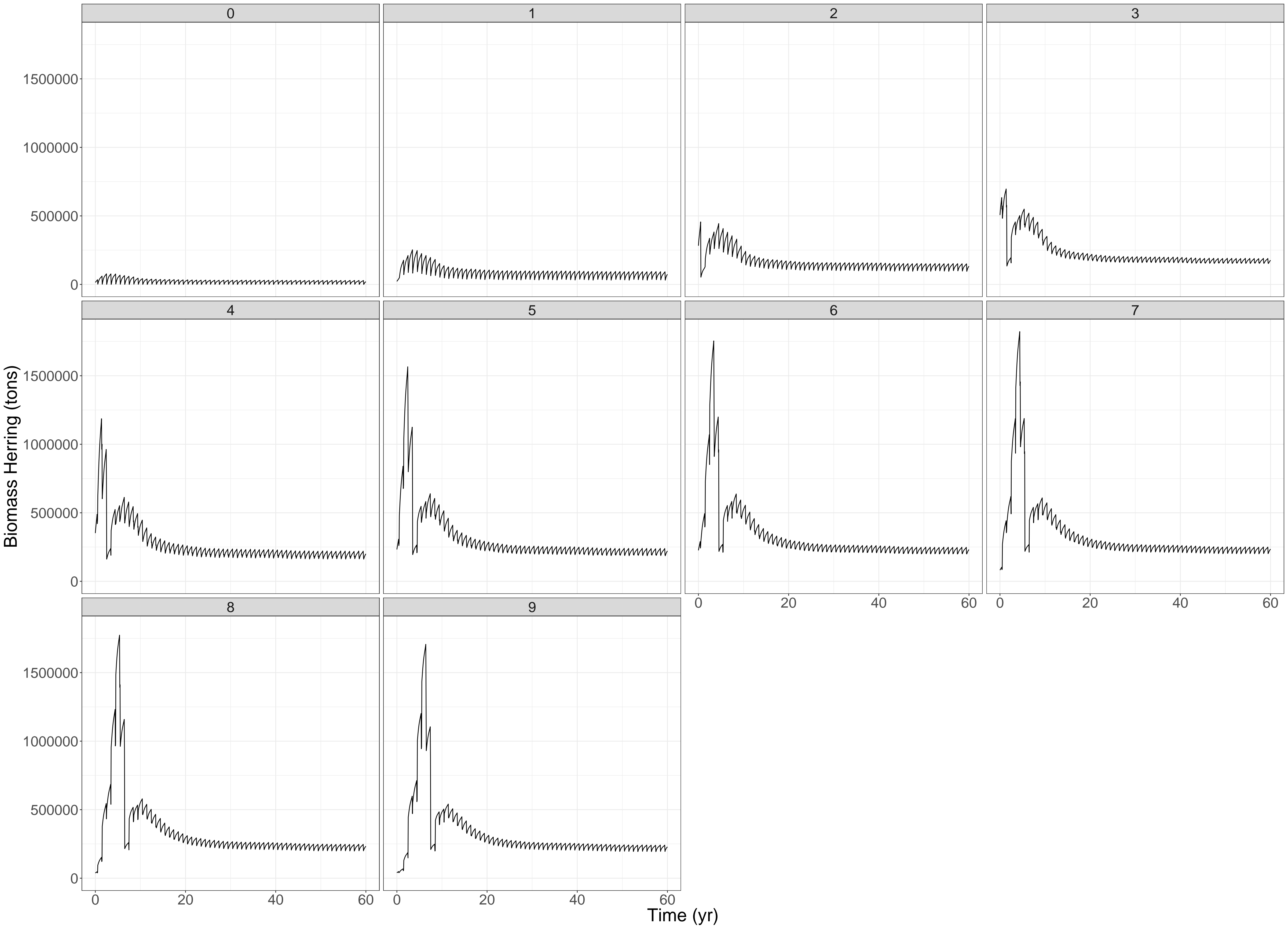

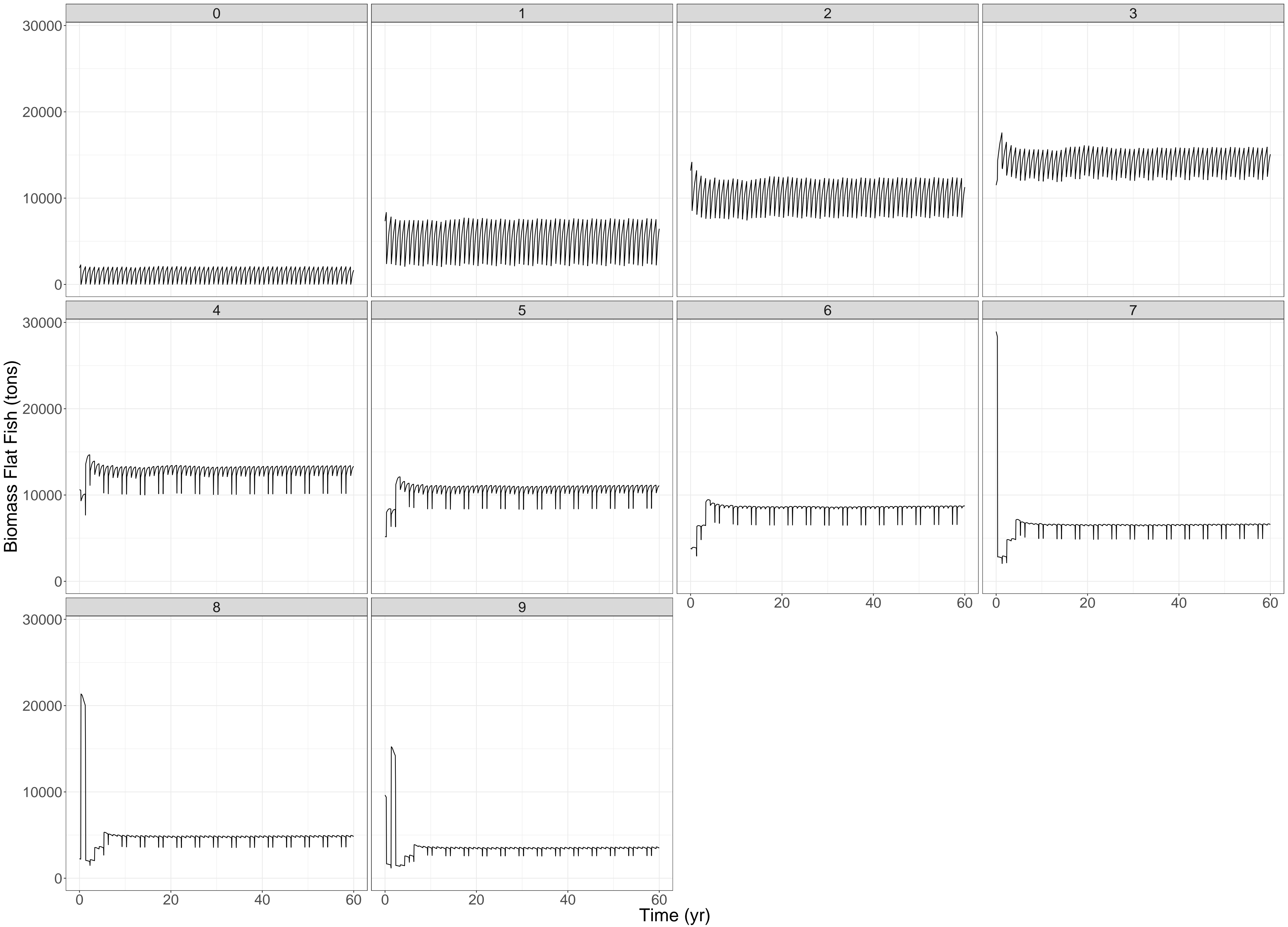

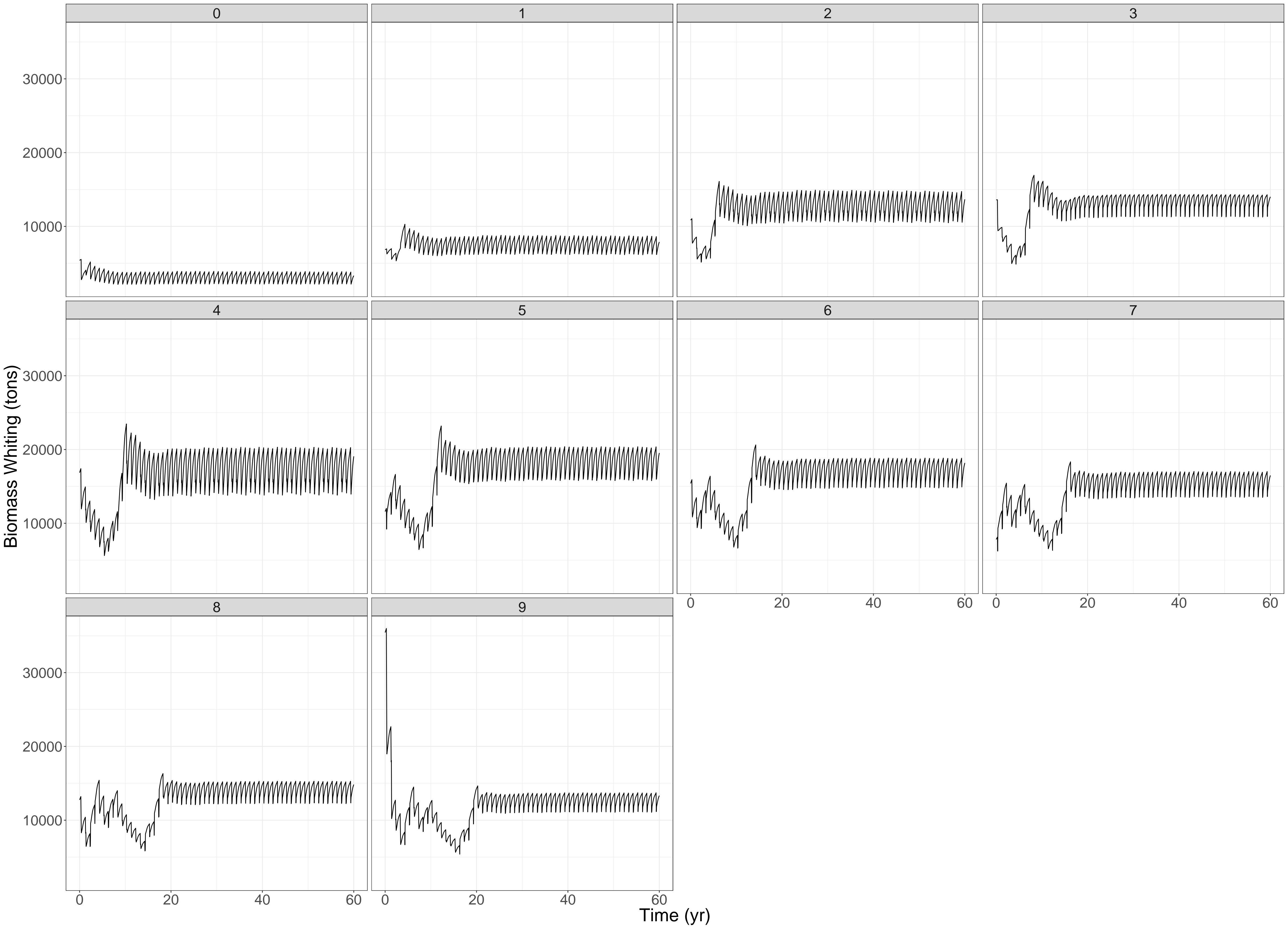

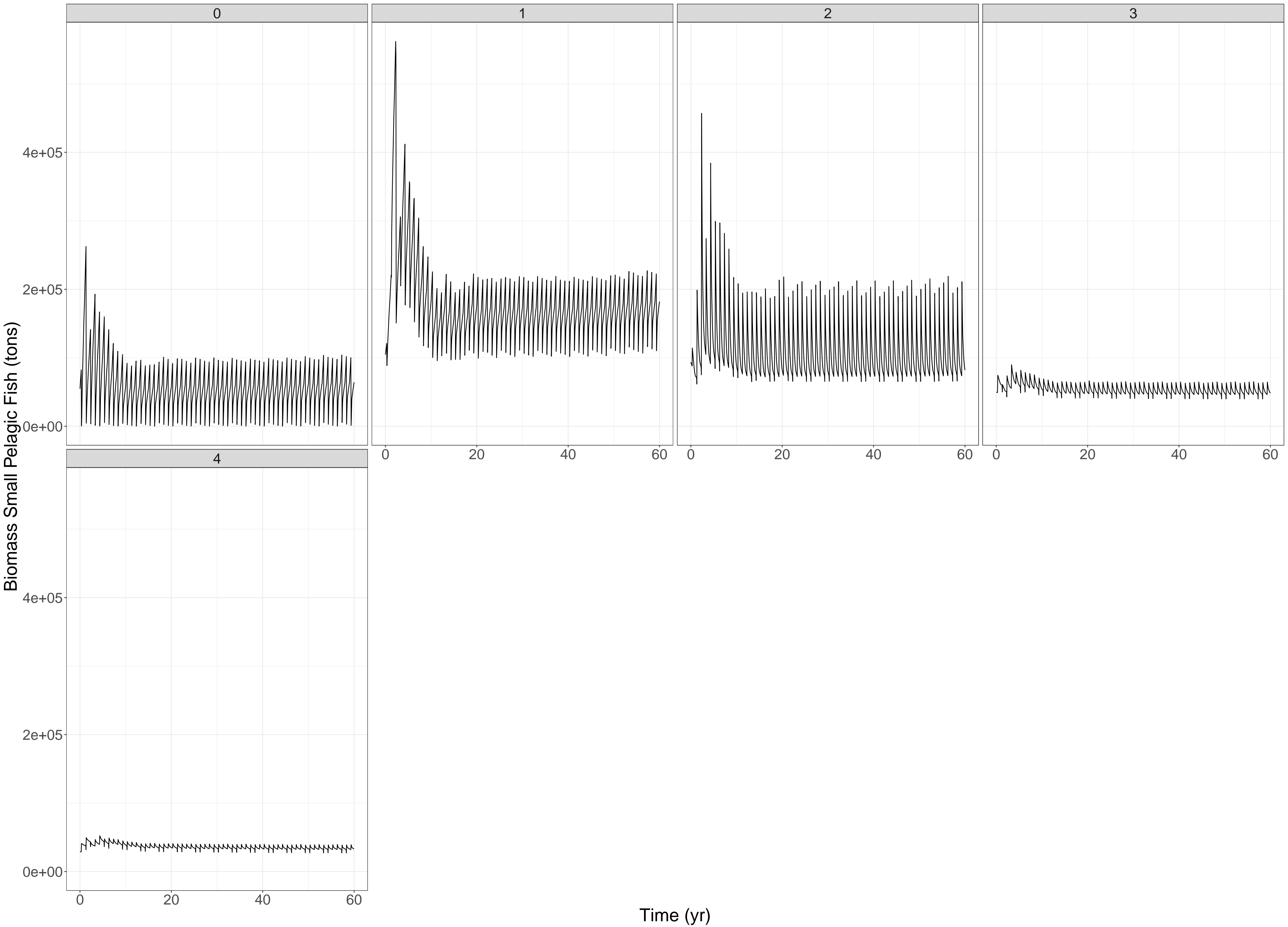

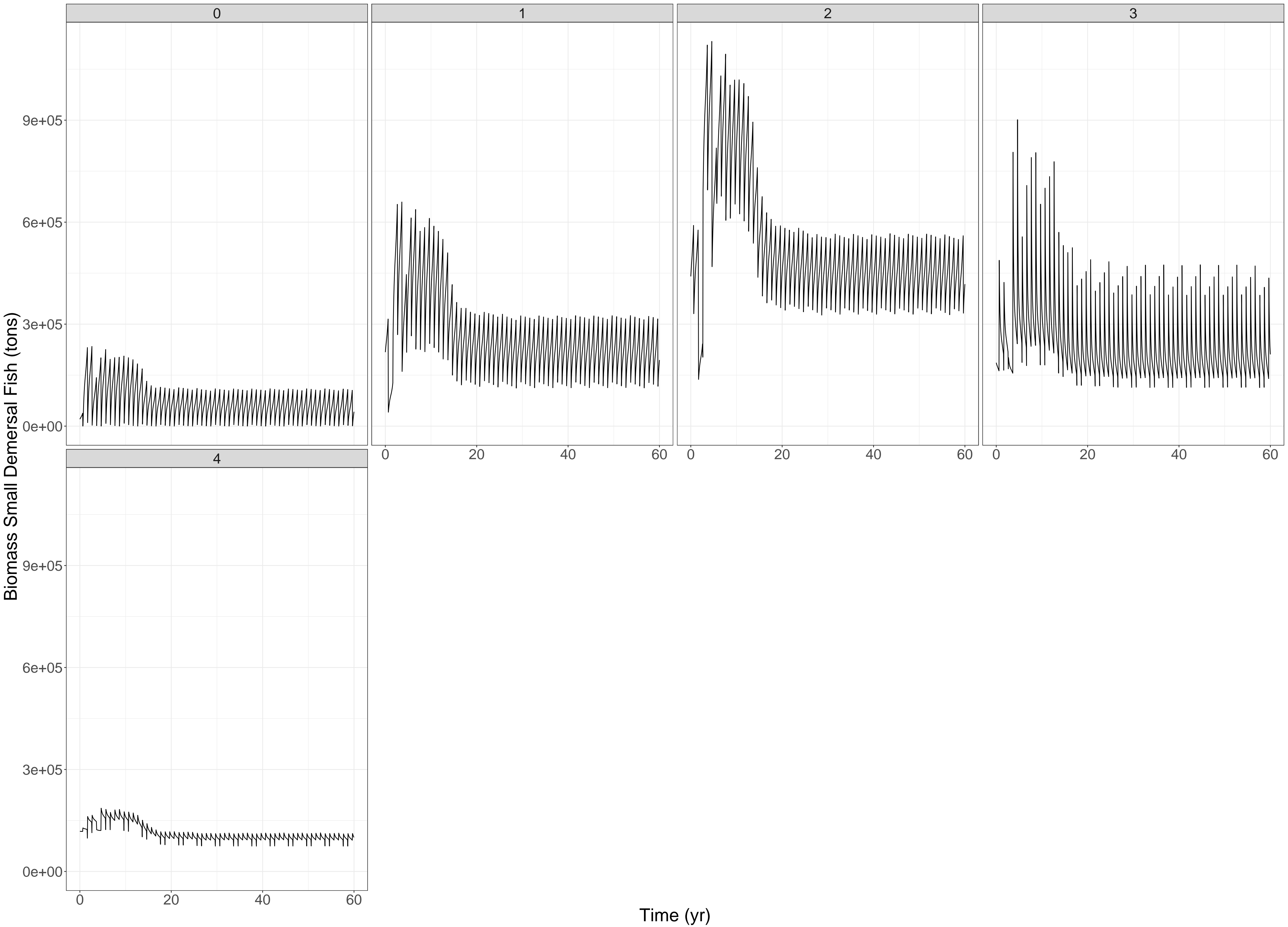

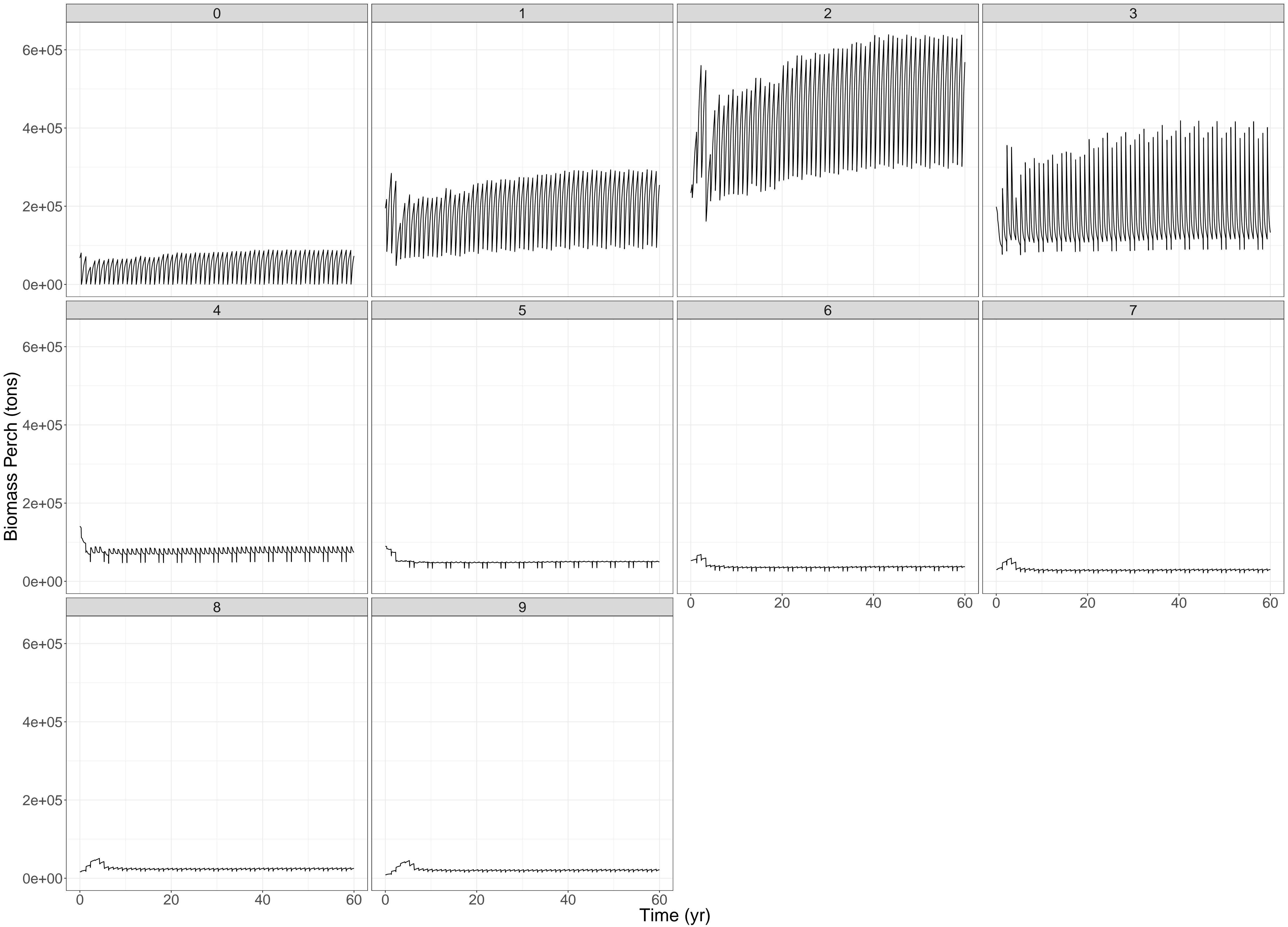

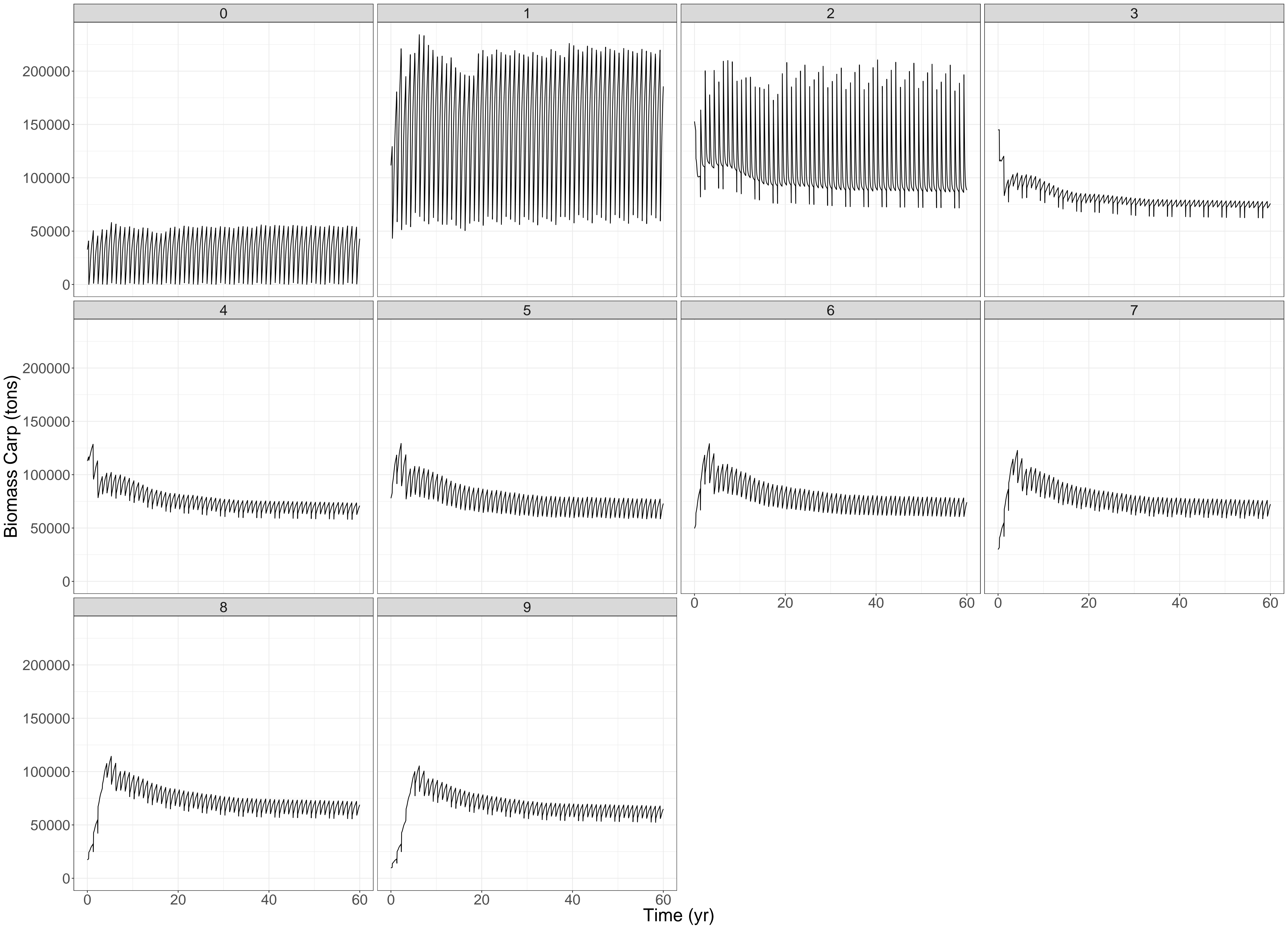

Supplement: S1 File — Supporting Information A. The Baltic Sea Atlantis: File A. Input data of the tracers per box for the Baltic Atlantis File B. Input data of the tracers per box and layer for the Baltic Atlantis File C. Input data for the fill values for the tracers for the Baltic Atlantis Figure A. Schematic diagram illustrating the structure of the coupled HBM-ERGOM model system Figure B. 120 year simulation run Figure C. The FISHRENT model diagram, here applied to Kattegat and Western Baltic. Figure D. One-year cycle of Chl-a in the different polygons Figure E. Relative biomass–initial condition values compared with simulation outcome Figure F. Diet composition of all predators Figure G. Biomass per age group over time for all vertebrates Figure H. Demography distribution for all vertebrates—the number of individuals for each age group Figure I. Geographical distribution of all functional groups Figure J. Geographical distribution of oxygen in the different layers. Panel 1 = top layer, panel 7 = bottom layer Figure K. Total biomass of Cod for scenario 1 (baseline) compared to scenario 5 Figure L. Relative prey biomass for predator cod, baseline compared to scenario 5 Figure M. One-year cycle of nutrients in the different polygons Table A. Physical and geochemical parameters used to internally force the Baltic Atlantis model. Table B. Summary of riverine + direct point source waterborne nitrogen loads applied to the Baltic Atlantis grid based on information from the Review of the Fifth Baltic Sea Pollution Load Compilation for the 2013 HELCOM Ministerial Meeting (HELCOM PLC-5.5). Nitrogen fractionation between DIN and DON based on Savchuk et al. (2012). Bioavailable fraction of DON assumed equal to labile DON as in Savchuk and Wolff (2009). Coastal retention fractions from Savchuk and Wolff (2009). Table C. Summary of key sources used to inform the biological module of Baltic Atlantis in relation to abundance and biomass, demography, prey-predator interaction and other functions. Ta [file pone.0199168.s001.zip › FigG.pdf]

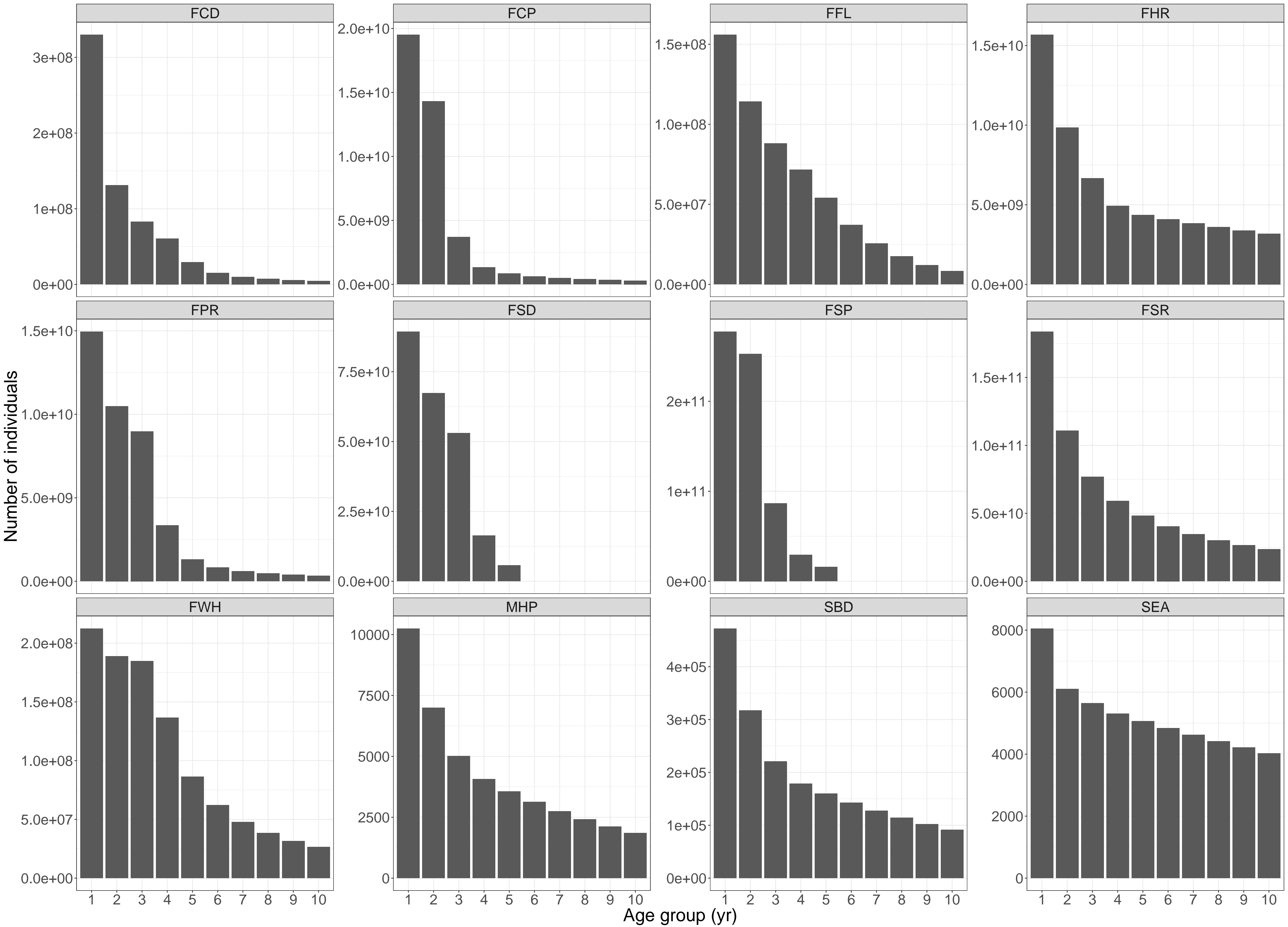

Supplement: S1 File — Supporting Information A. The Baltic Sea Atlantis: File A. Input data of the tracers per box for the Baltic Atlantis File B. Input data of the tracers per box and layer for the Baltic Atlantis File C. Input data for the fill values for the tracers for the Baltic Atlantis Figure A. Schematic diagram illustrating the structure of the coupled HBM-ERGOM model system Figure B. 120 year simulation run Figure C. The FISHRENT model diagram, here applied to Kattegat and Western Baltic. Figure D. One-year cycle of Chl-a in the different polygons Figure E. Relative biomass–initial condition values compared with simulation outcome Figure F. Diet composition of all predators Figure G. Biomass per age group over time for all vertebrates Figure H. Demography distribution for all vertebrates—the number of individuals for each age group Figure I. Geographical distribution of all functional groups Figure J. Geographical distribution of oxygen in the different layers. Panel 1 = top layer, panel 7 = bottom layer Figure K. Total biomass of Cod for scenario 1 (baseline) compared to scenario 5 Figure L. Relative prey biomass for predator cod, baseline compared to scenario 5 Figure M. One-year cycle of nutrients in the different polygons Table A. Physical and geochemical parameters used to internally force the Baltic Atlantis model. Table B. Summary of riverine + direct point source waterborne nitrogen loads applied to the Baltic Atlantis grid based on information from the Review of the Fifth Baltic Sea Pollution Load Compilation for the 2013 HELCOM Ministerial Meeting (HELCOM PLC-5.5). Nitrogen fractionation between DIN and DON based on Savchuk et al. (2012). Bioavailable fraction of DON assumed equal to labile DON as in Savchuk and Wolff (2009). Coastal retention fractions from Savchuk and Wolff (2009). Table C. Summary of key sources used to inform the biological module of Baltic Atlantis in relation to abundance and biomass, demography, prey-predator interaction and other functions. Ta [file pone.0199168.s001.zip › FigH.pdf]

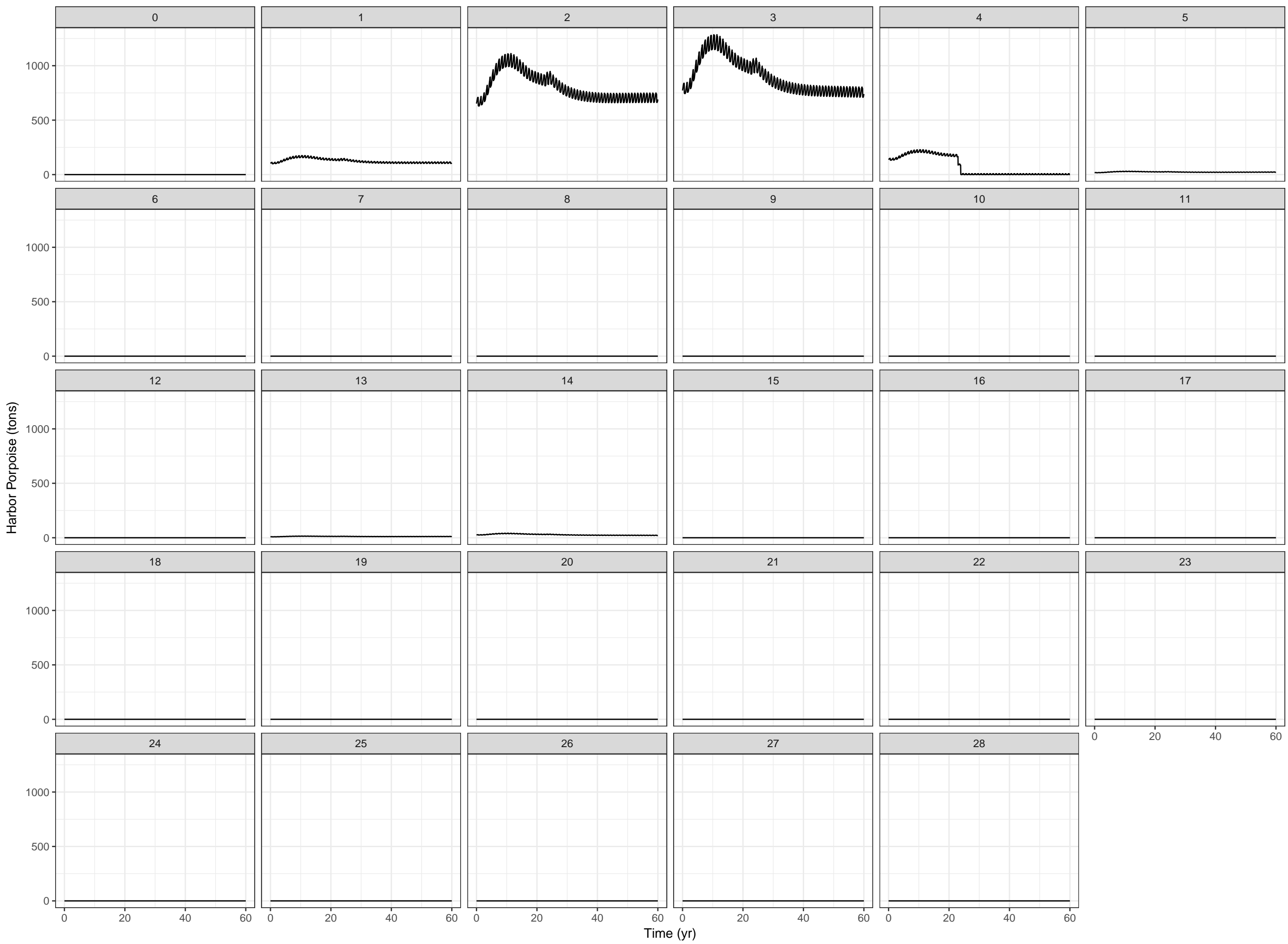

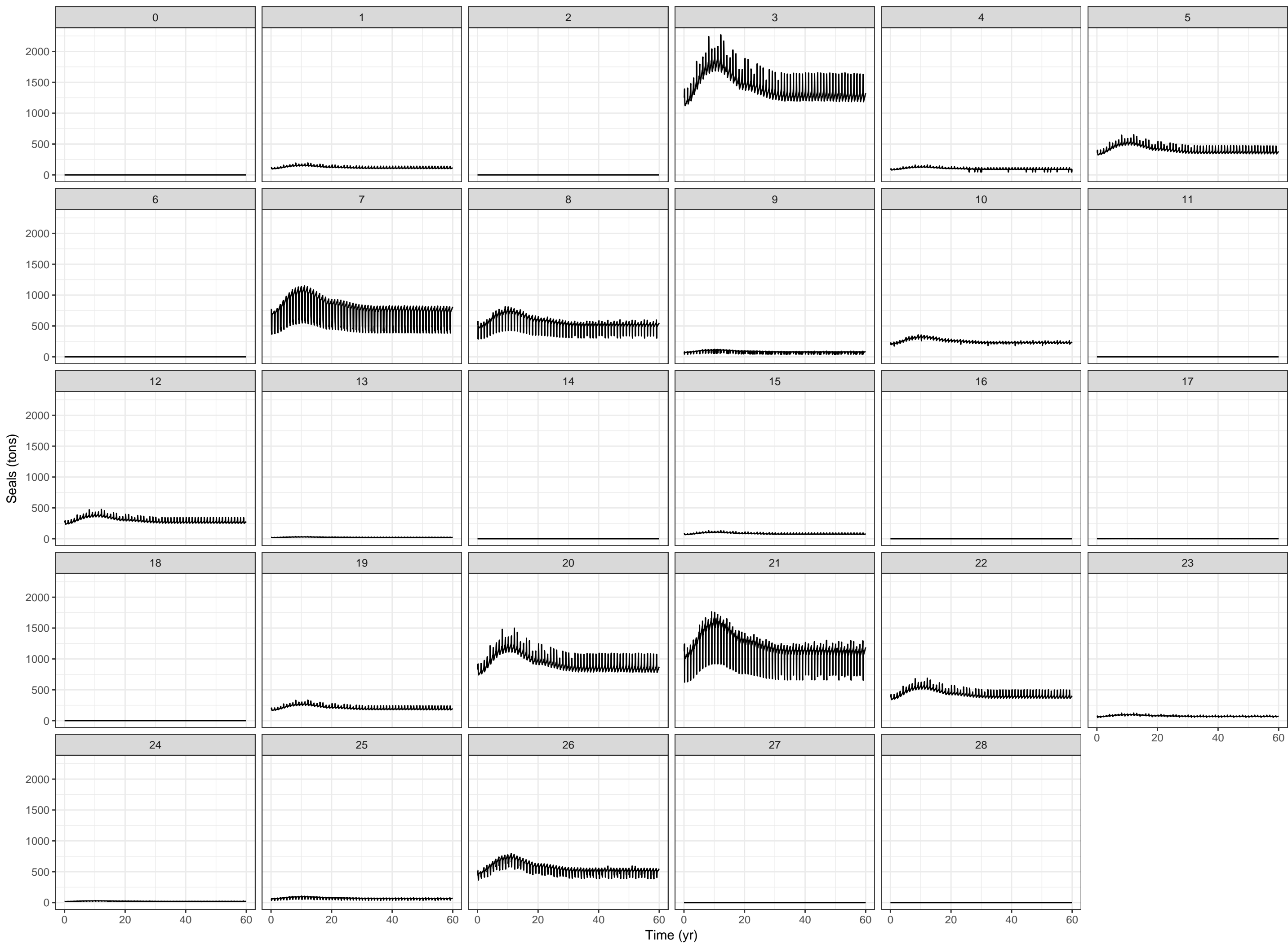

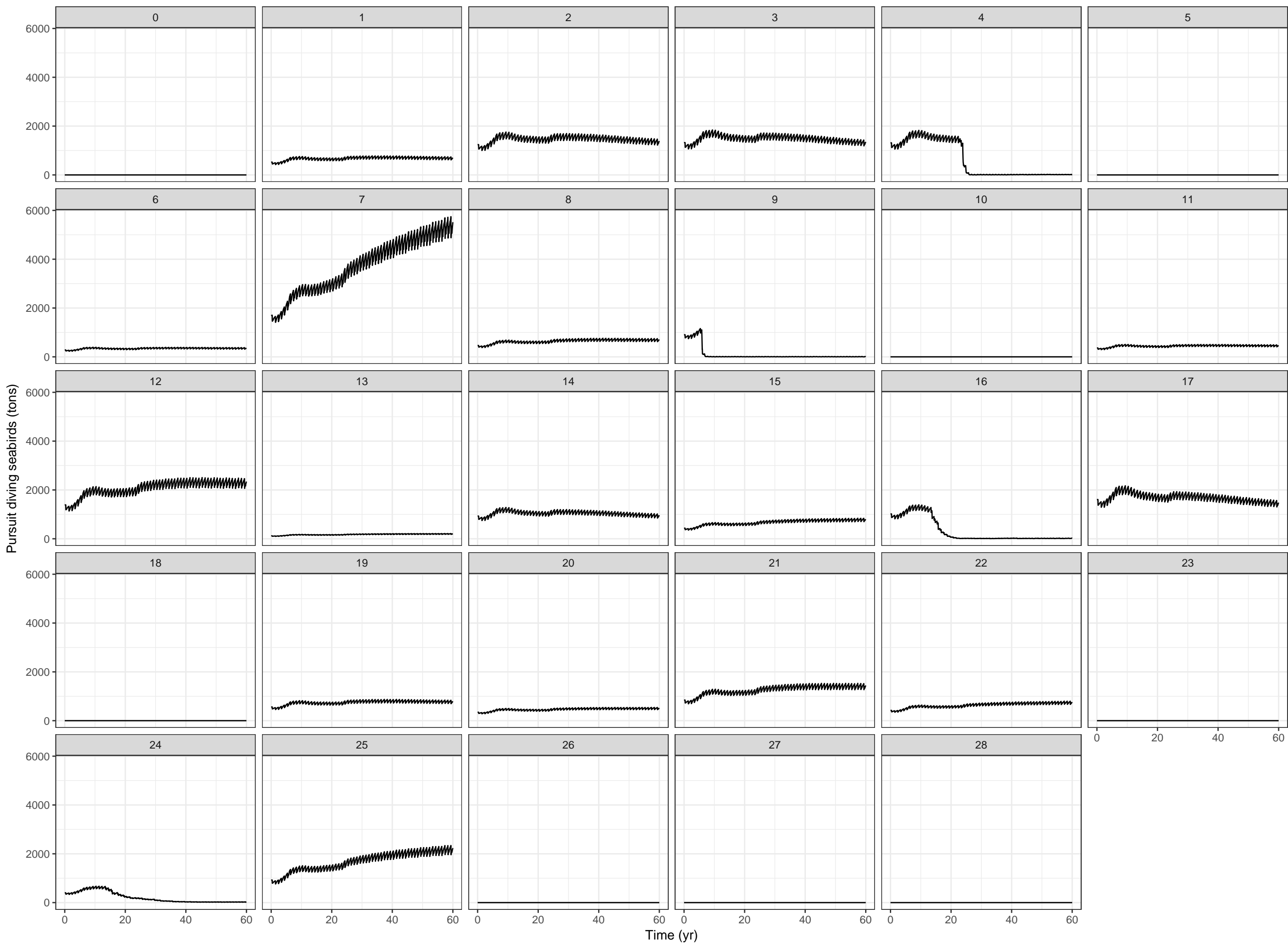

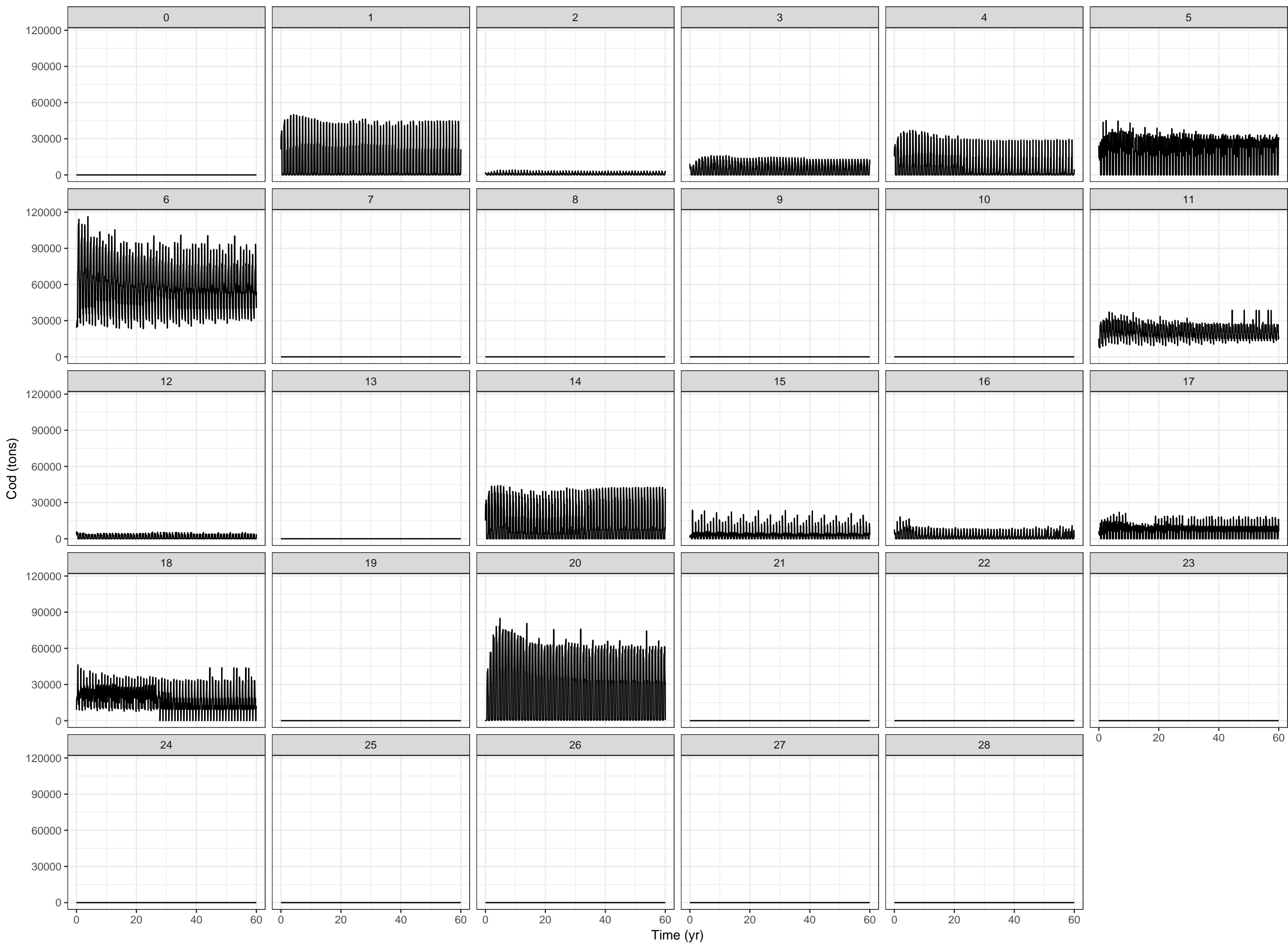

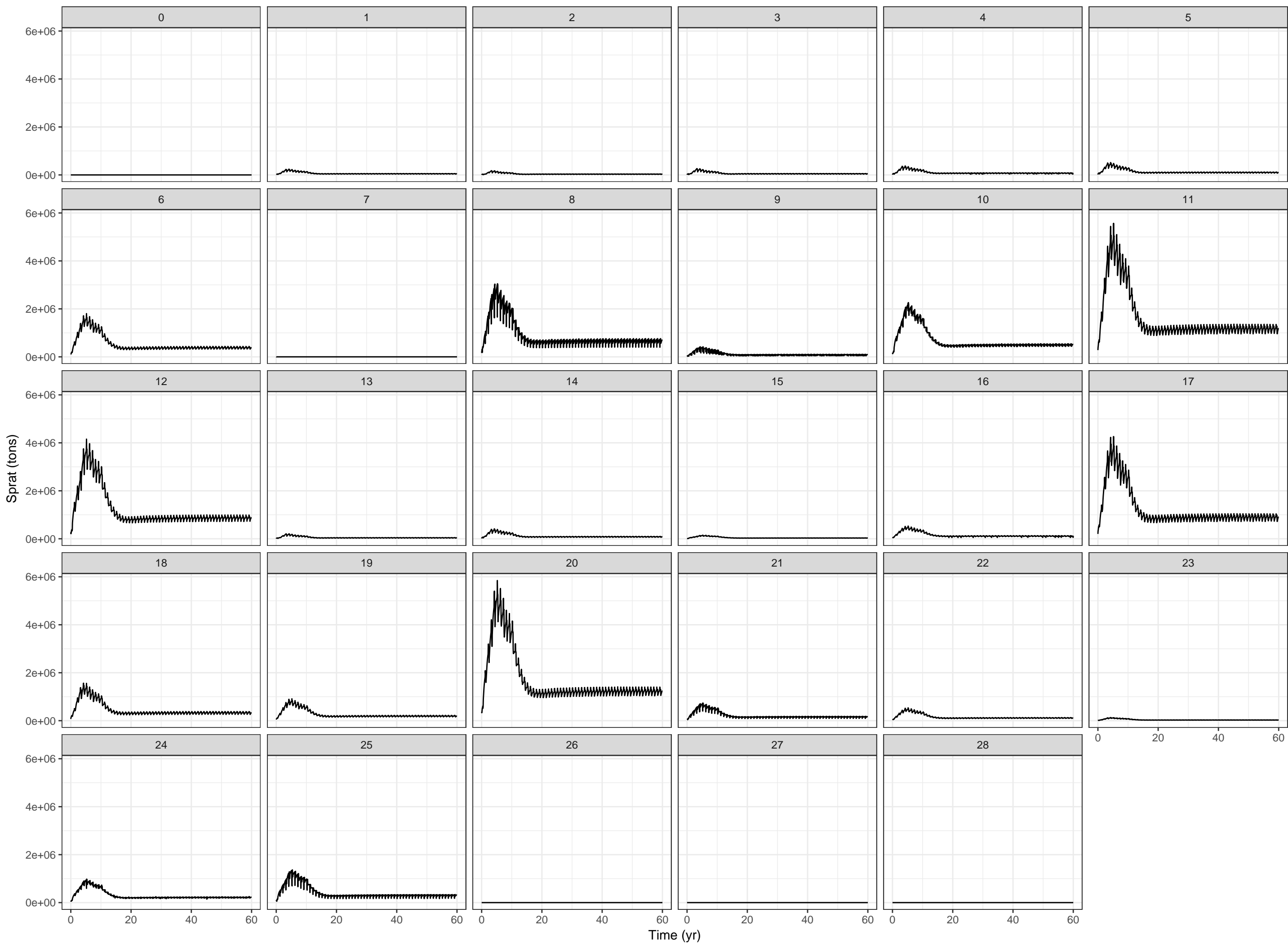

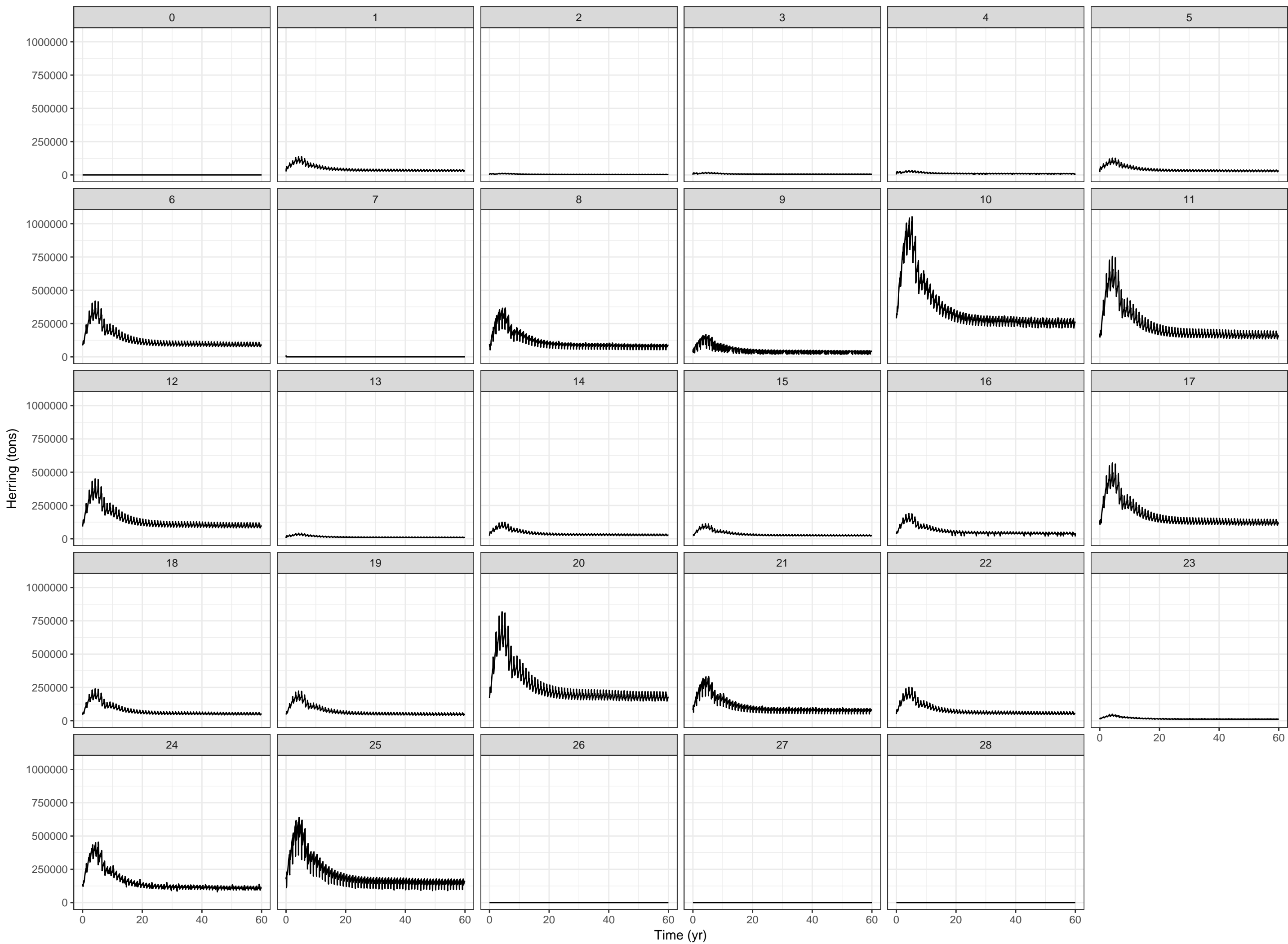

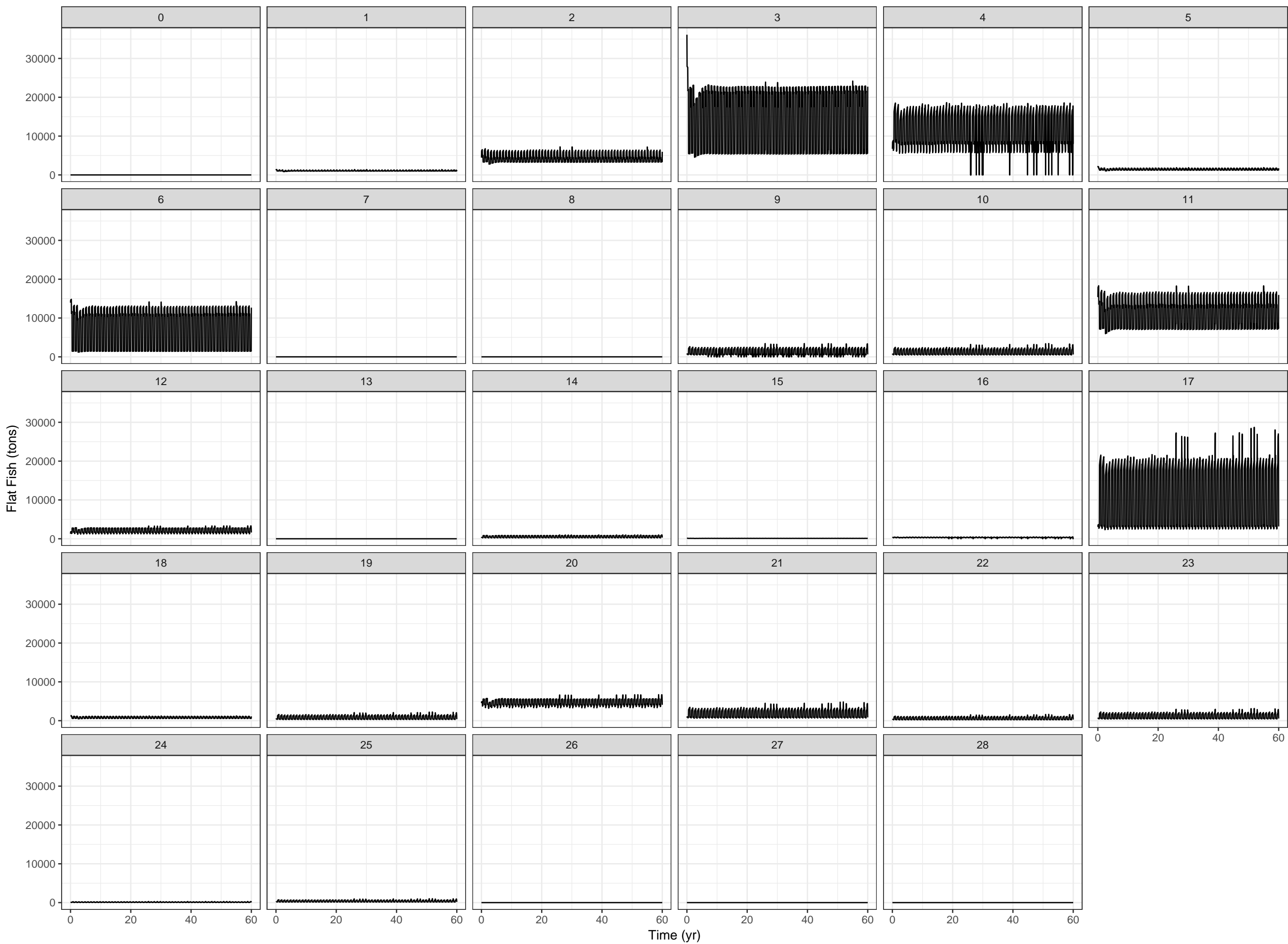

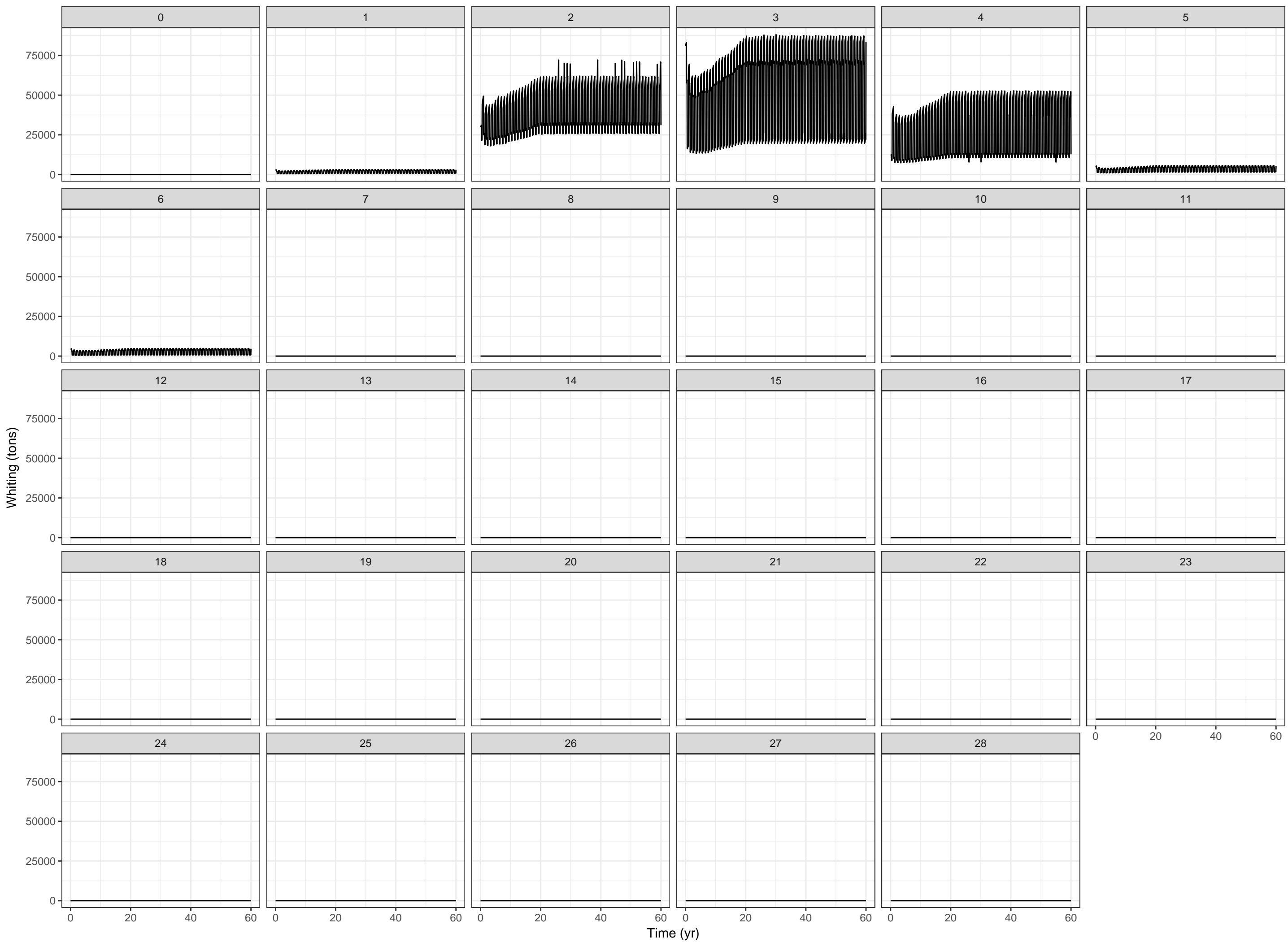

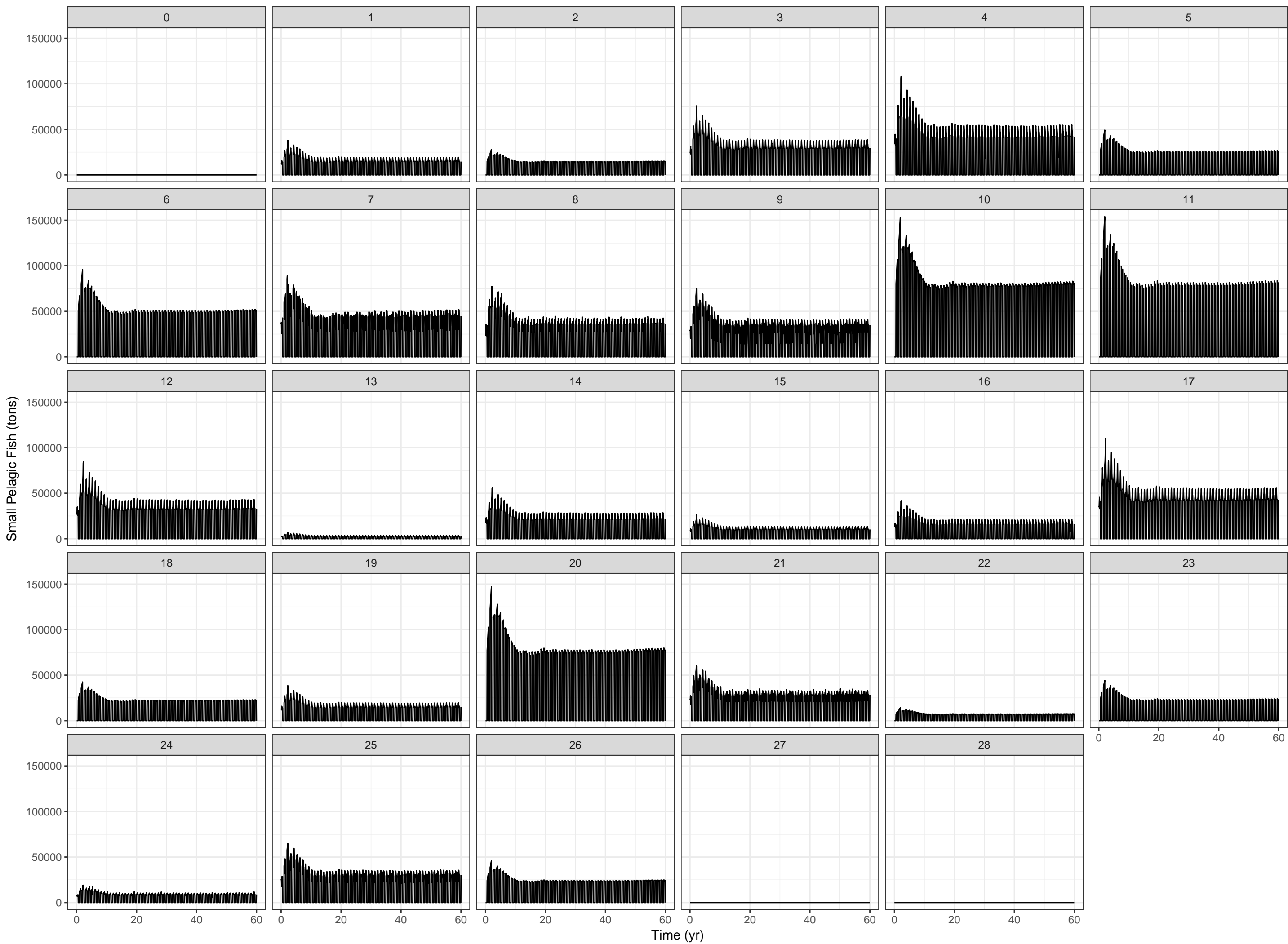

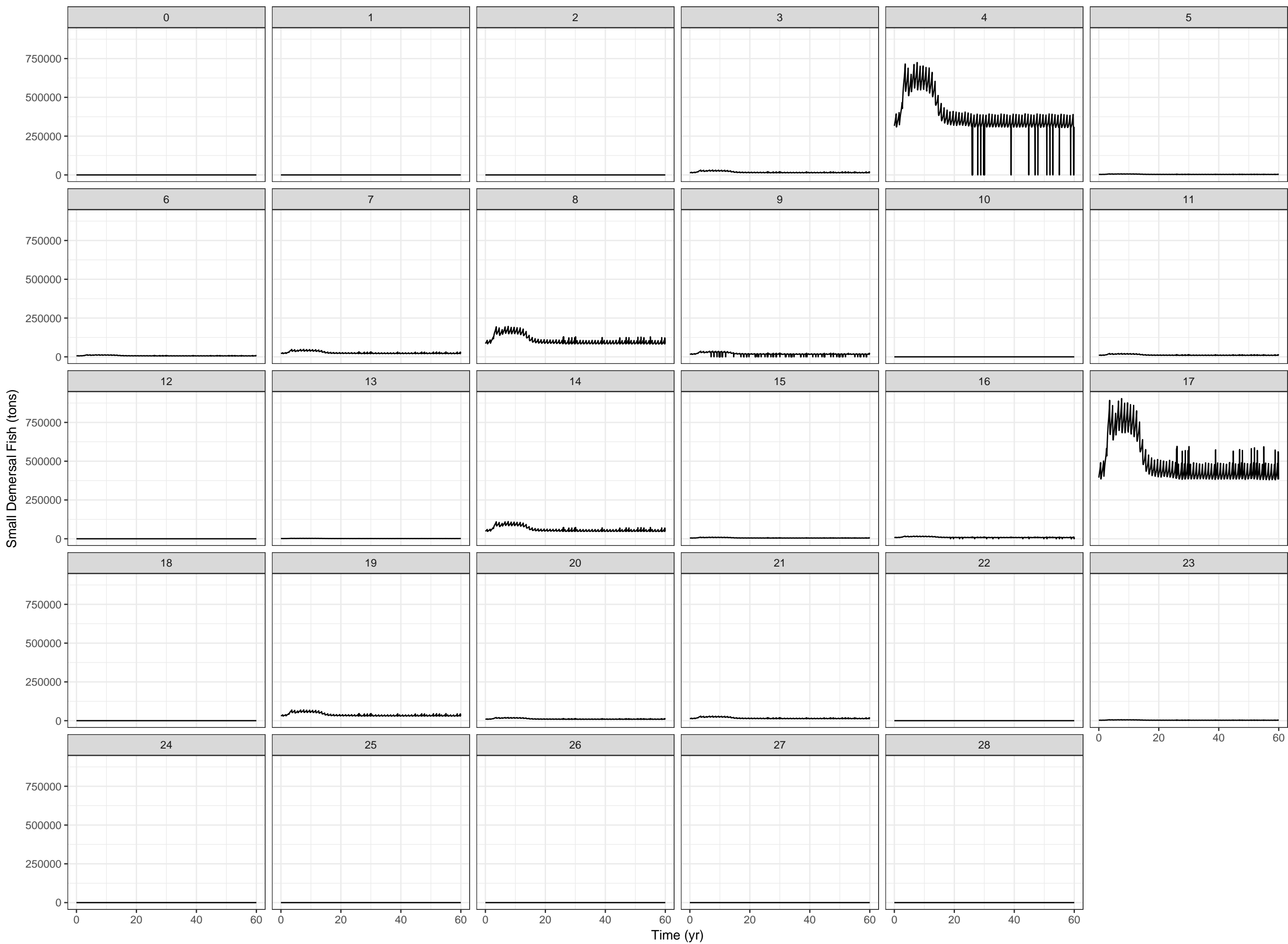

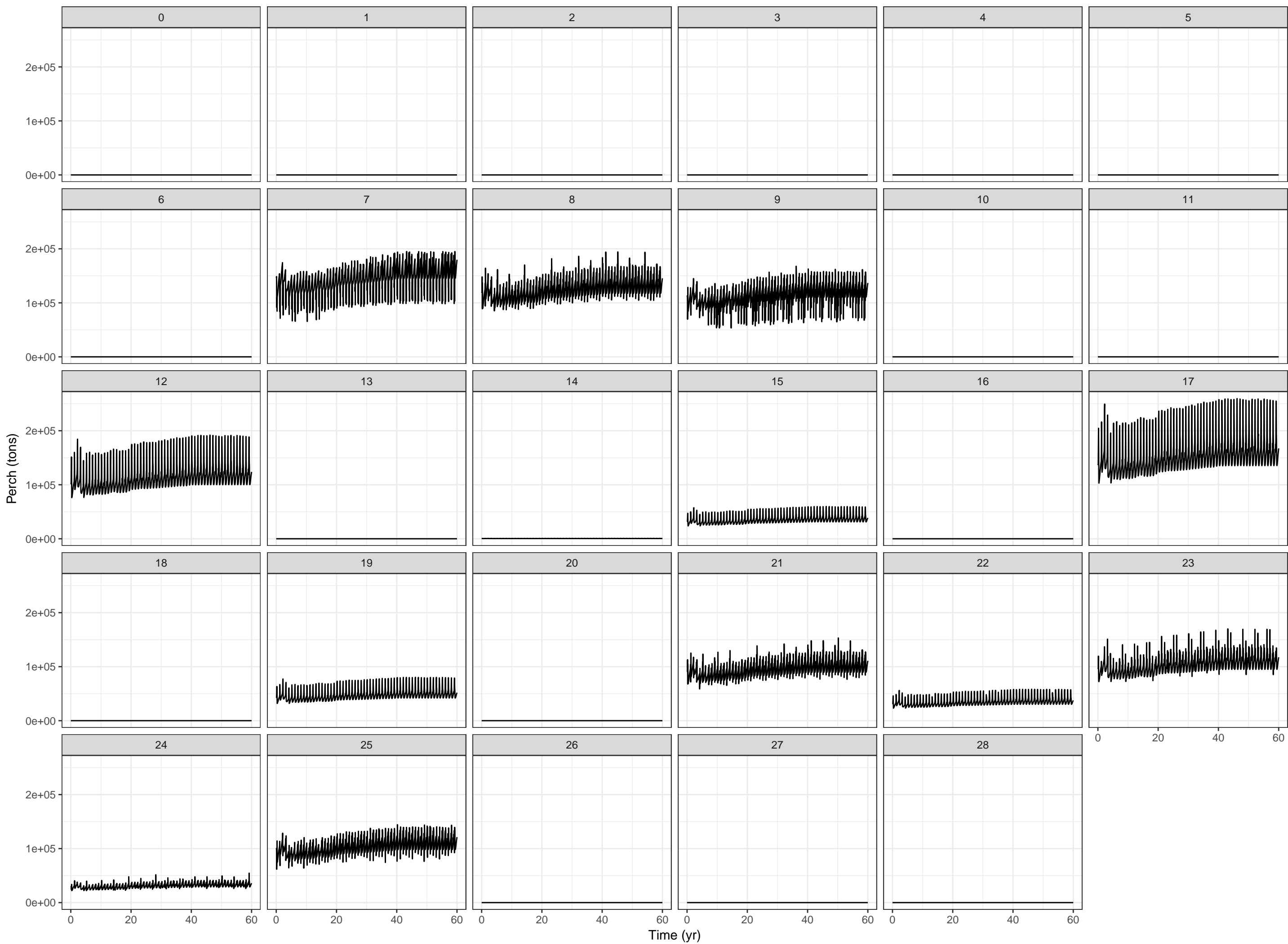

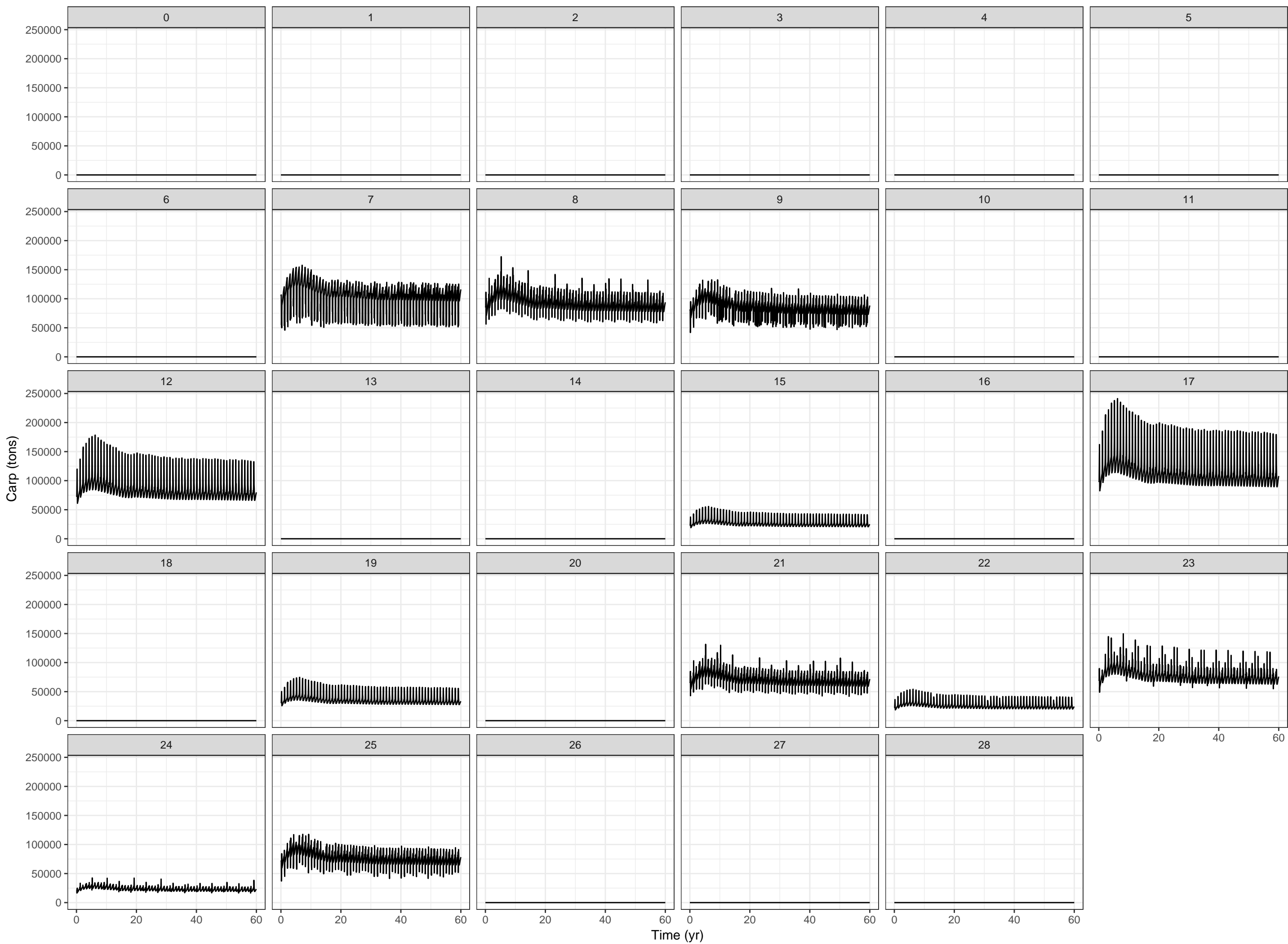

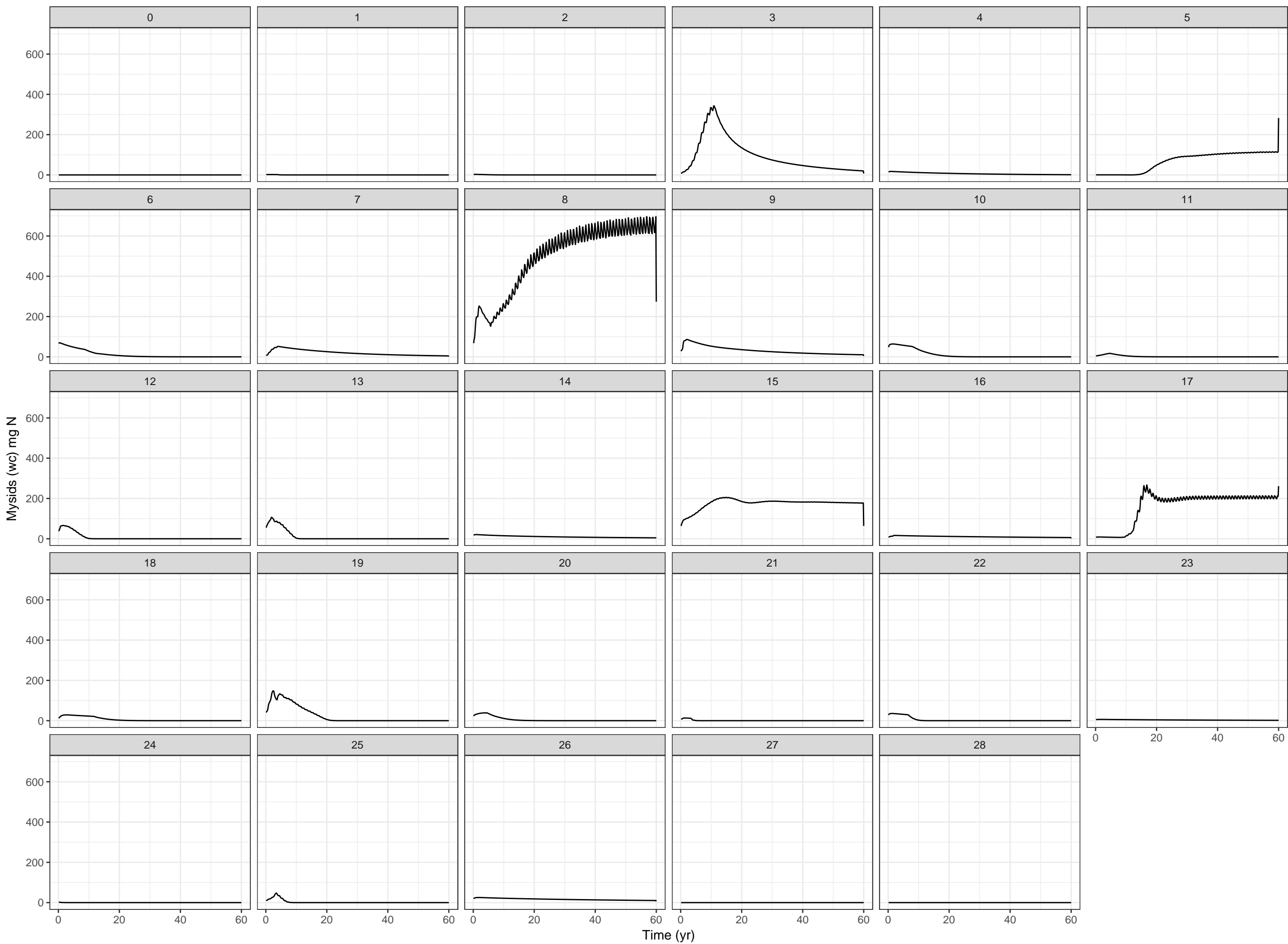

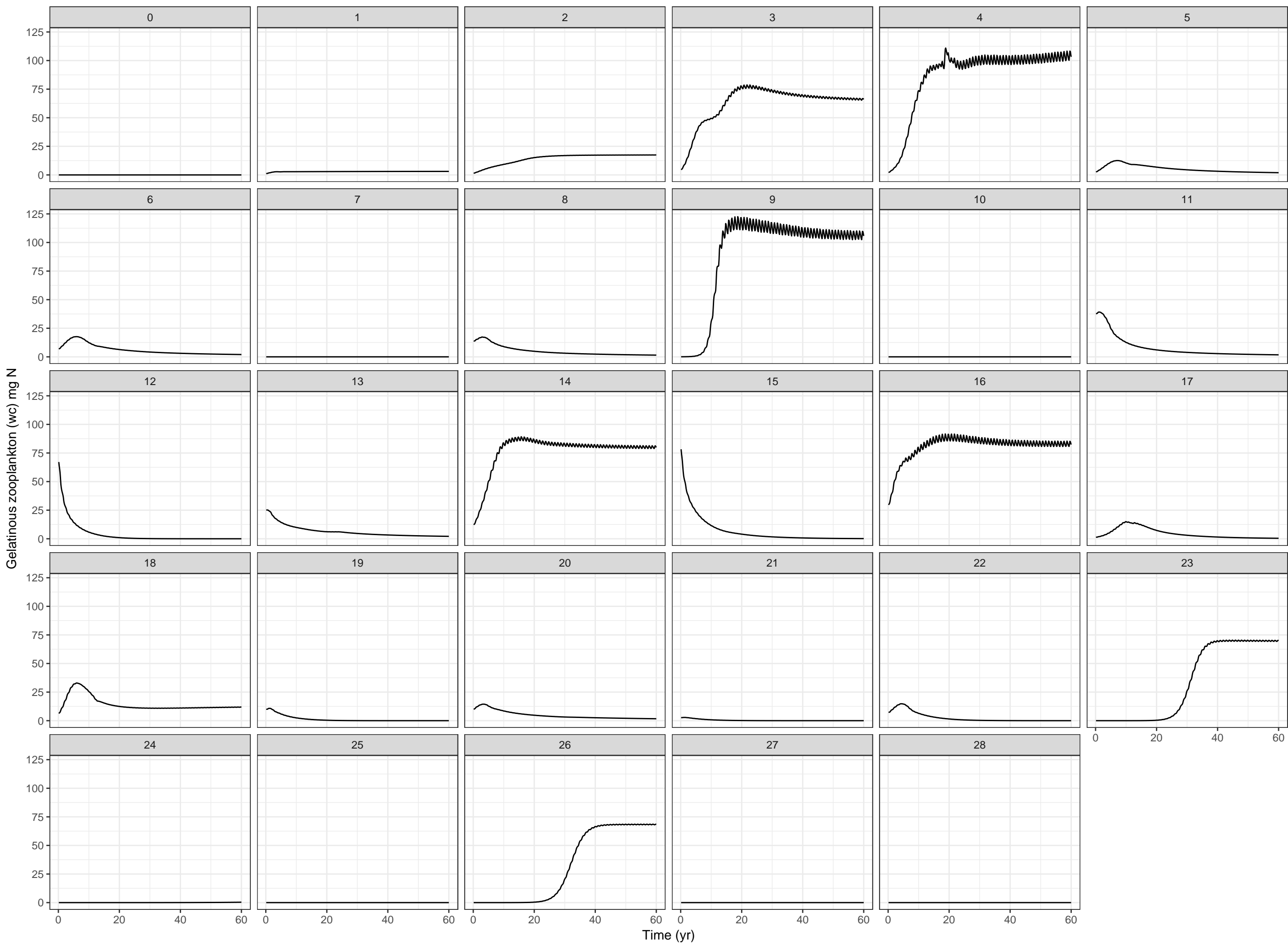

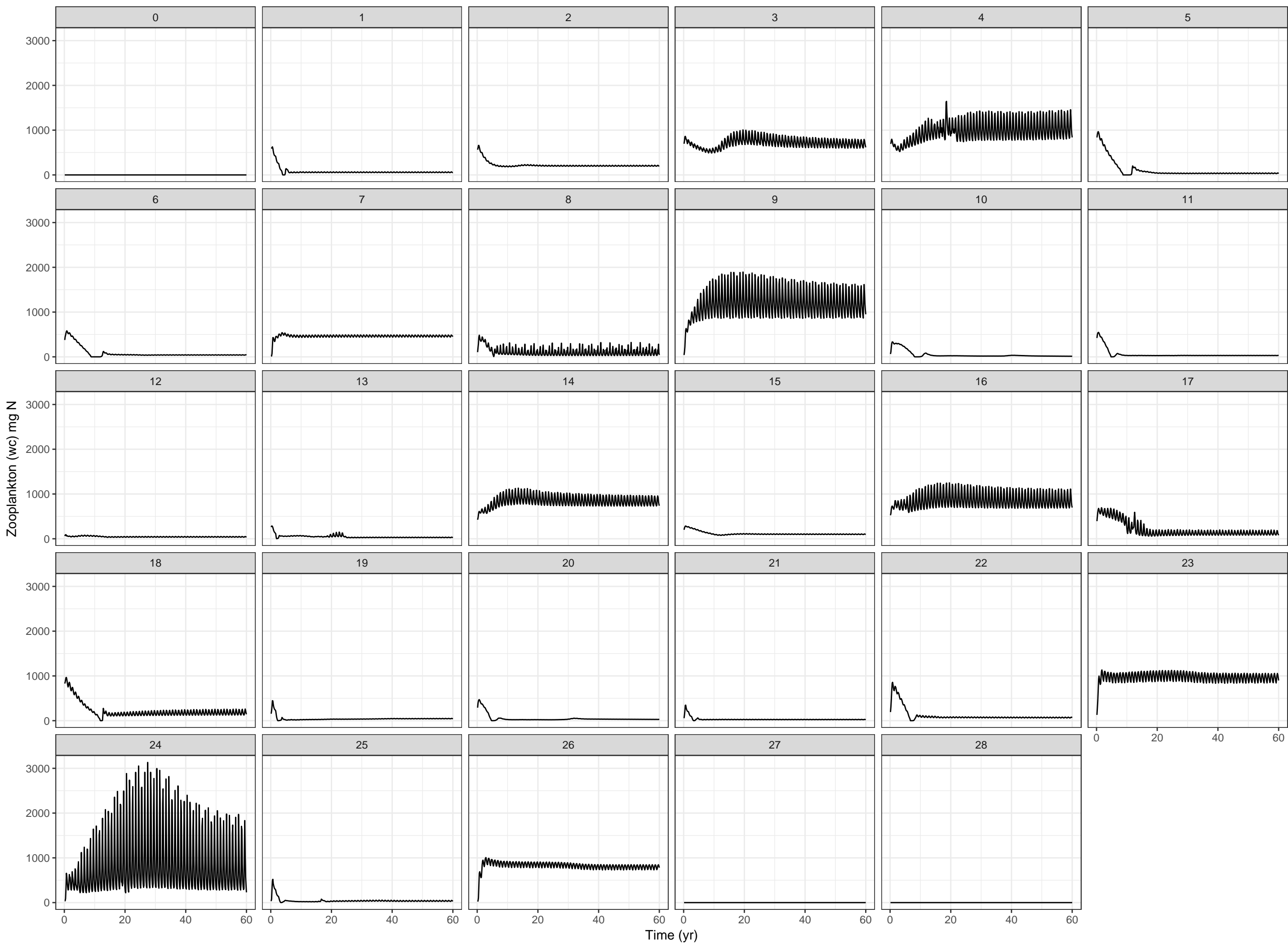

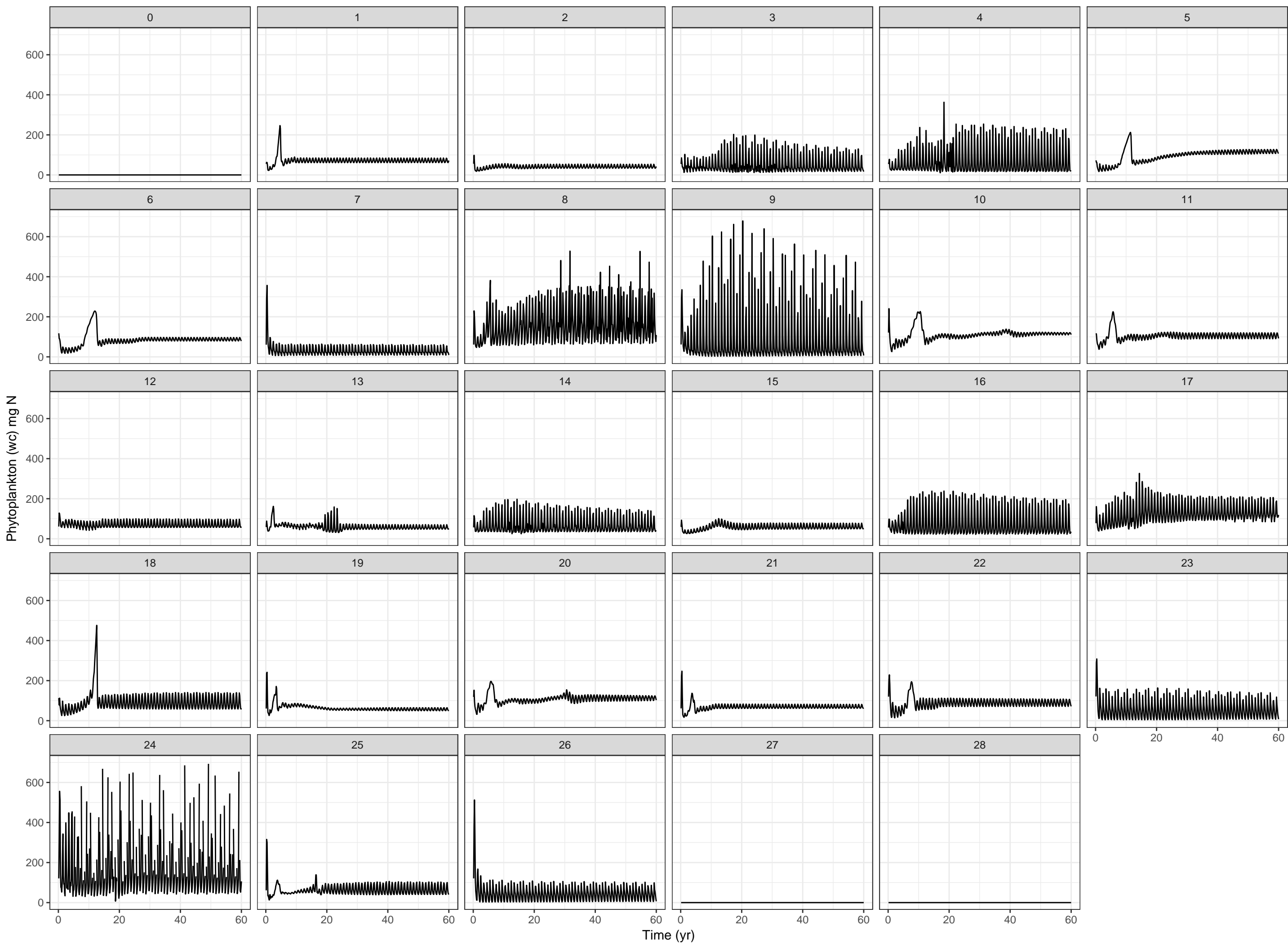

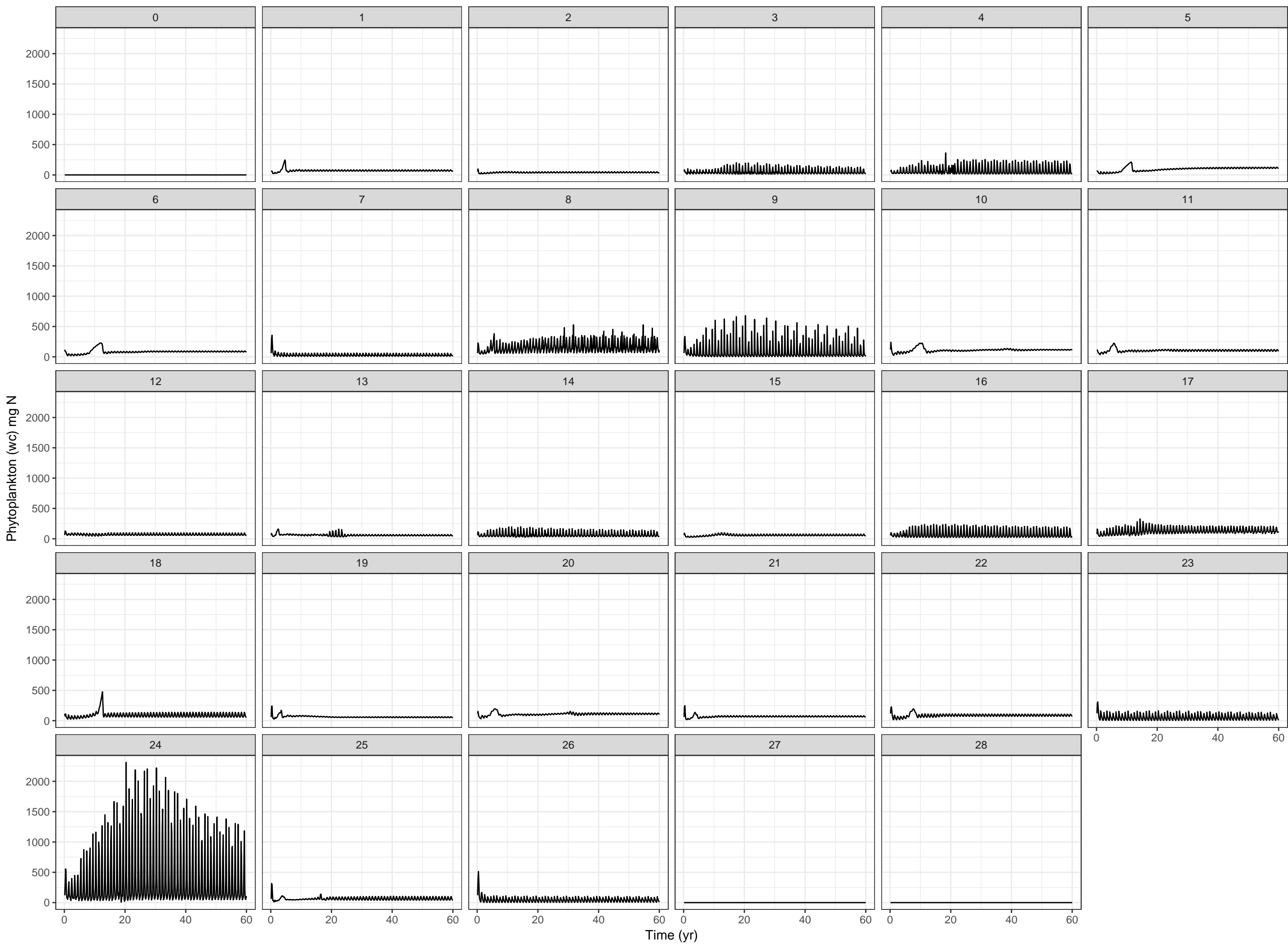

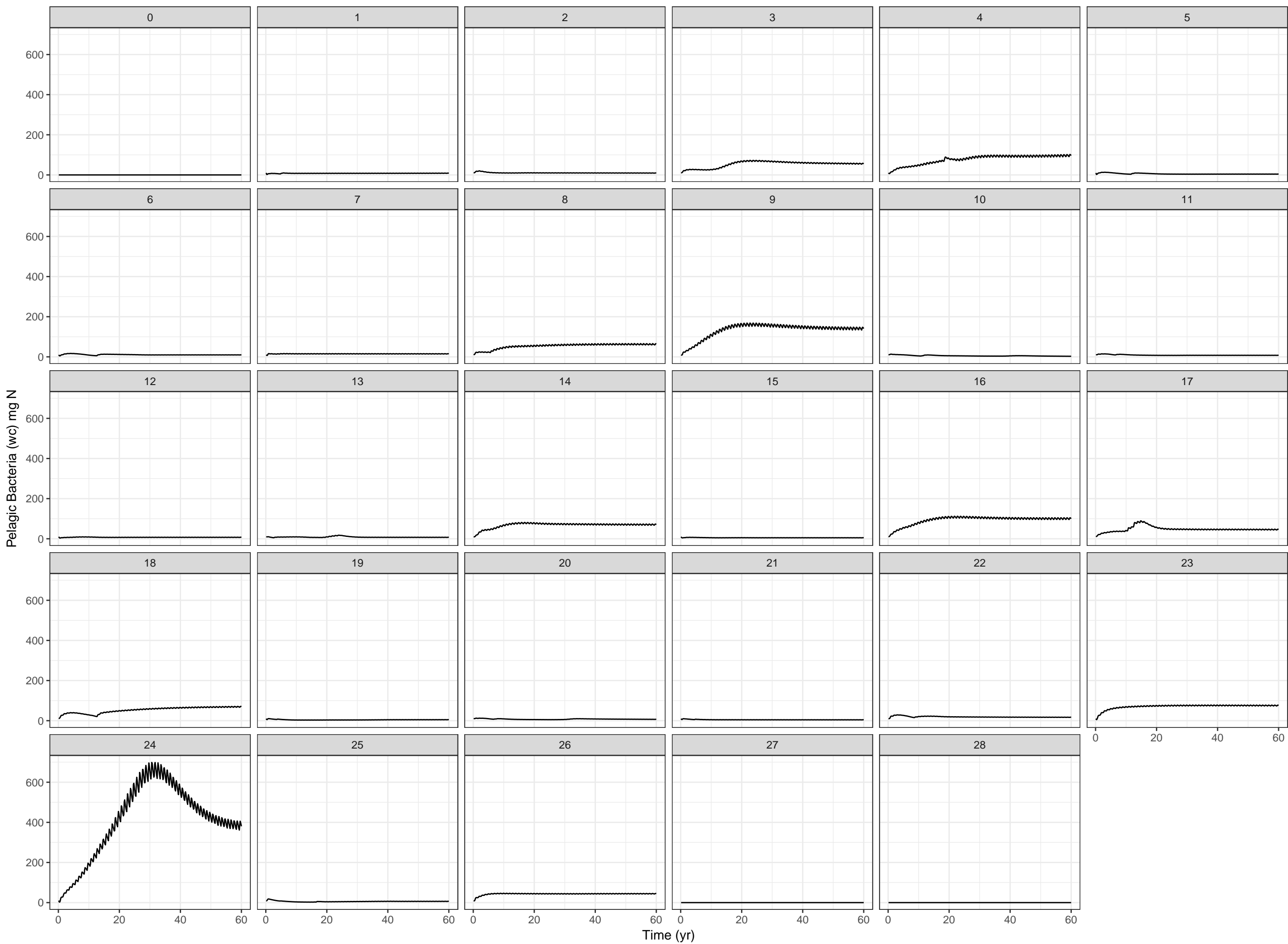

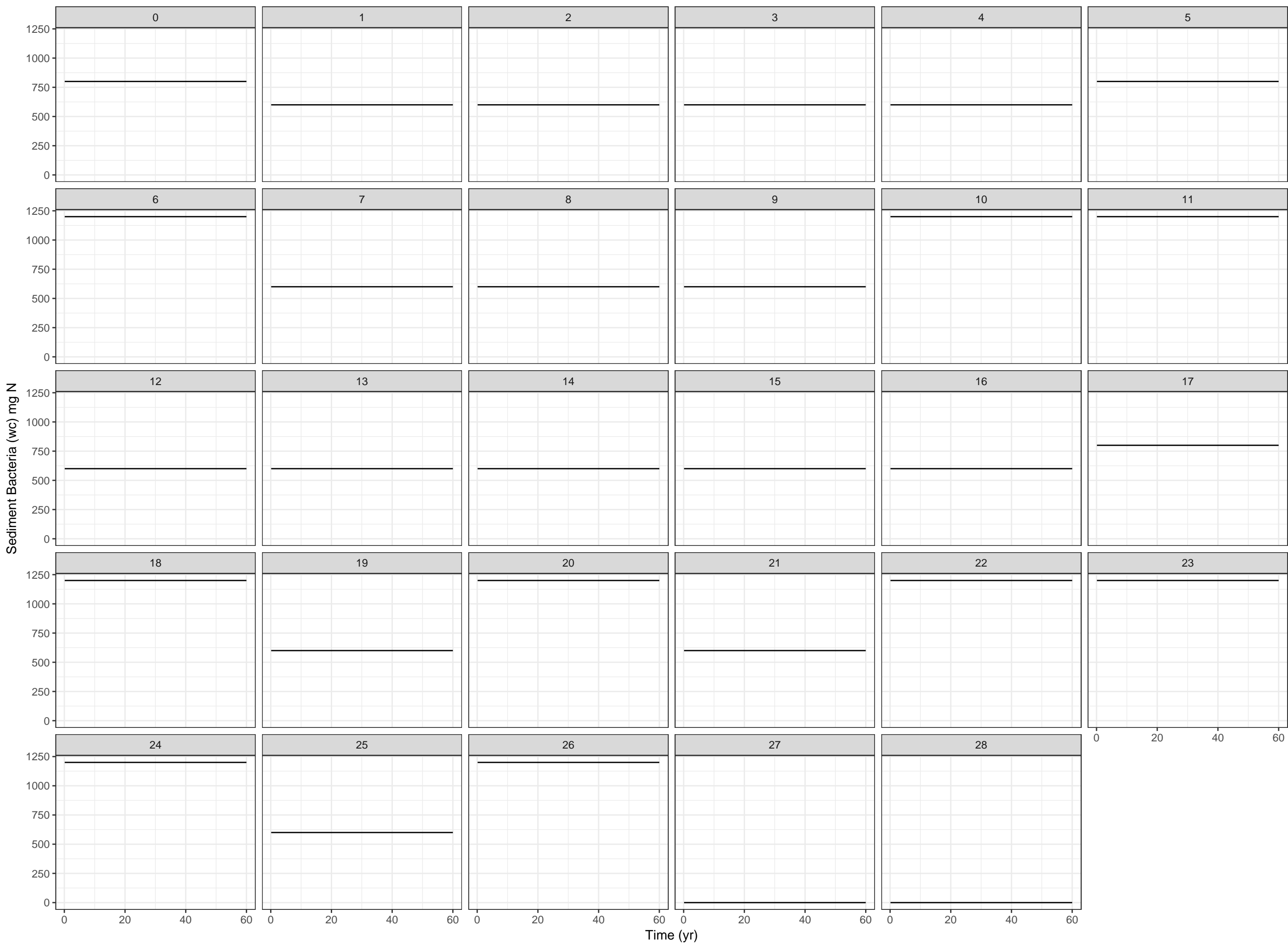

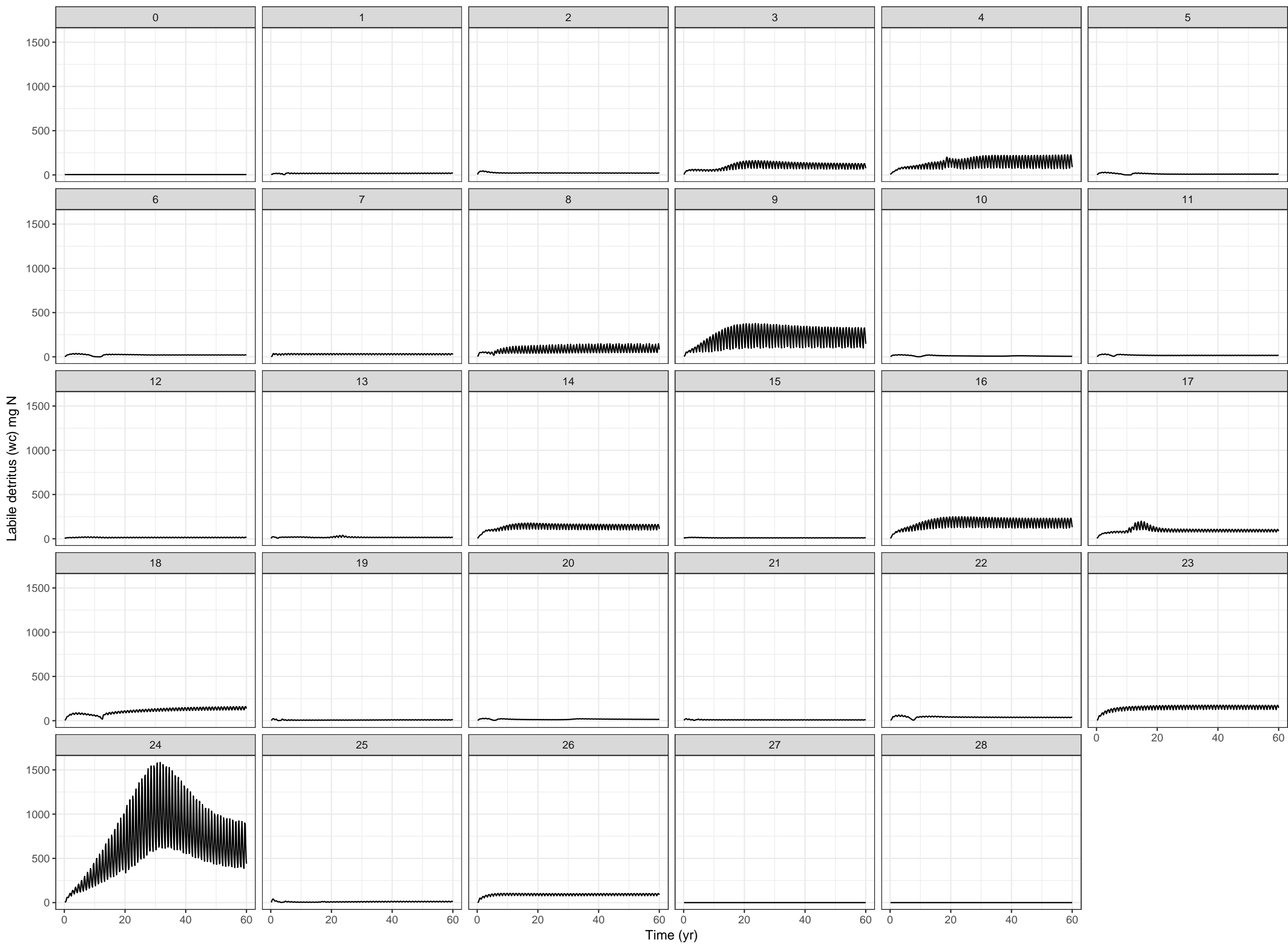

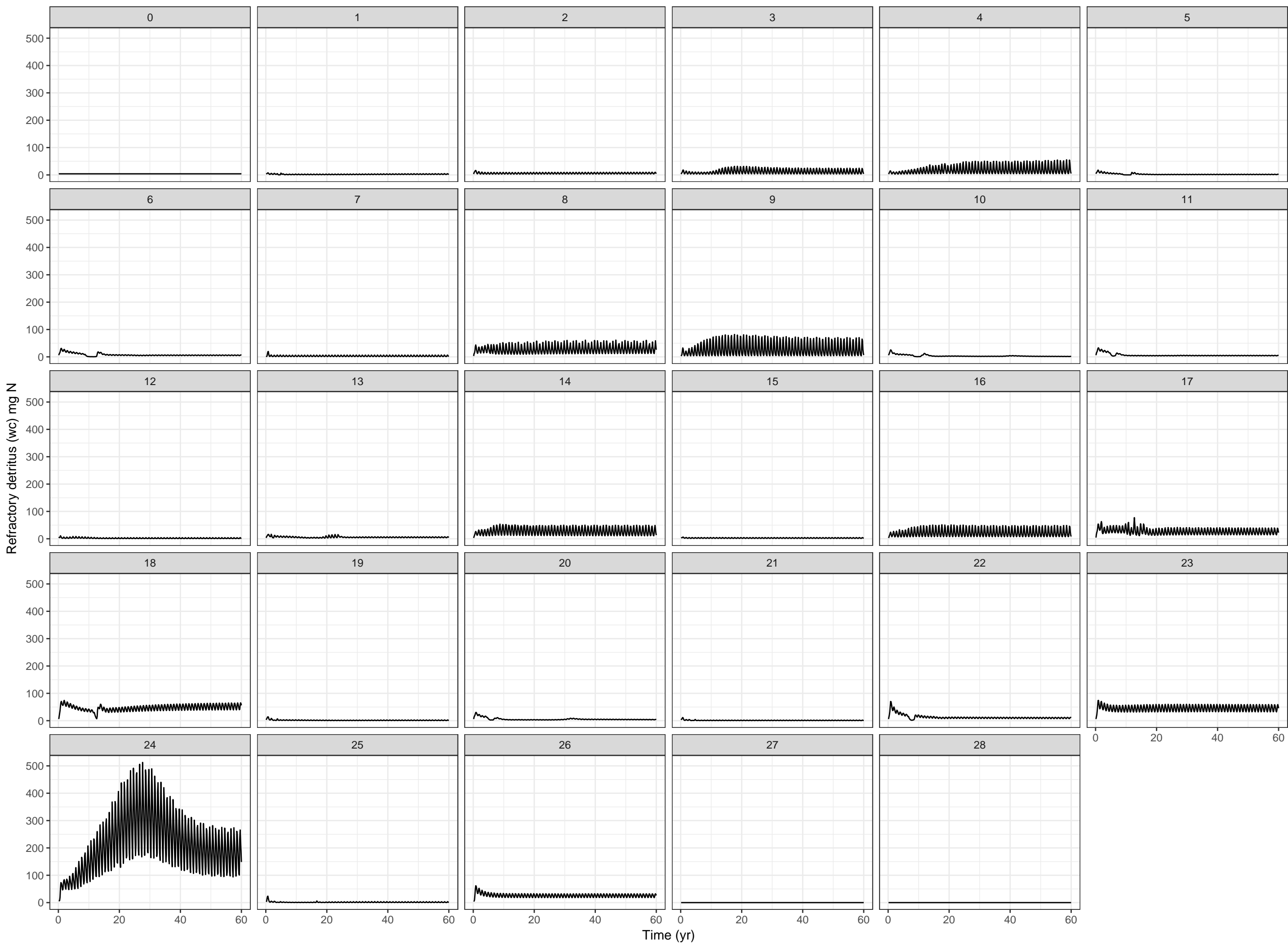

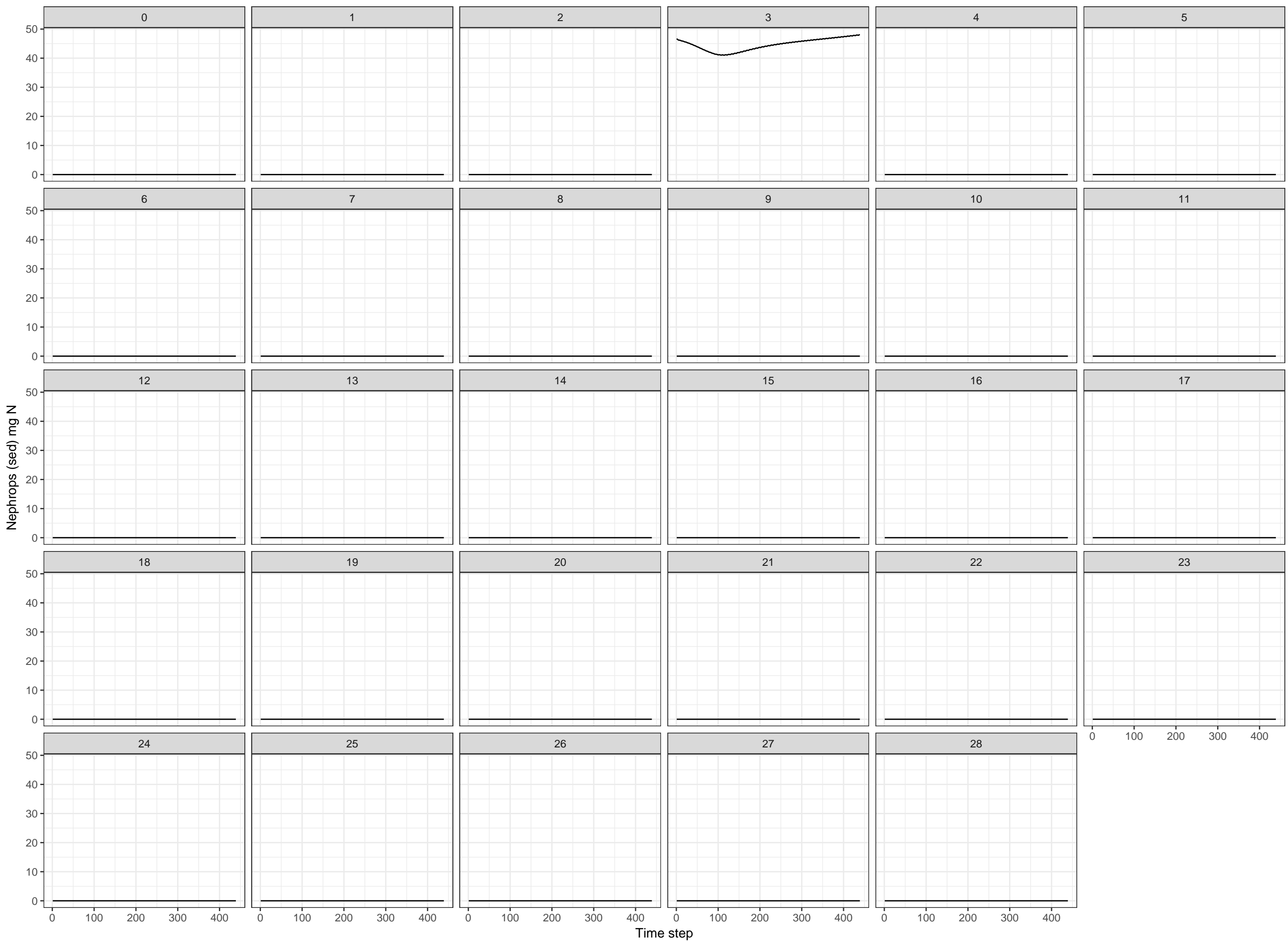

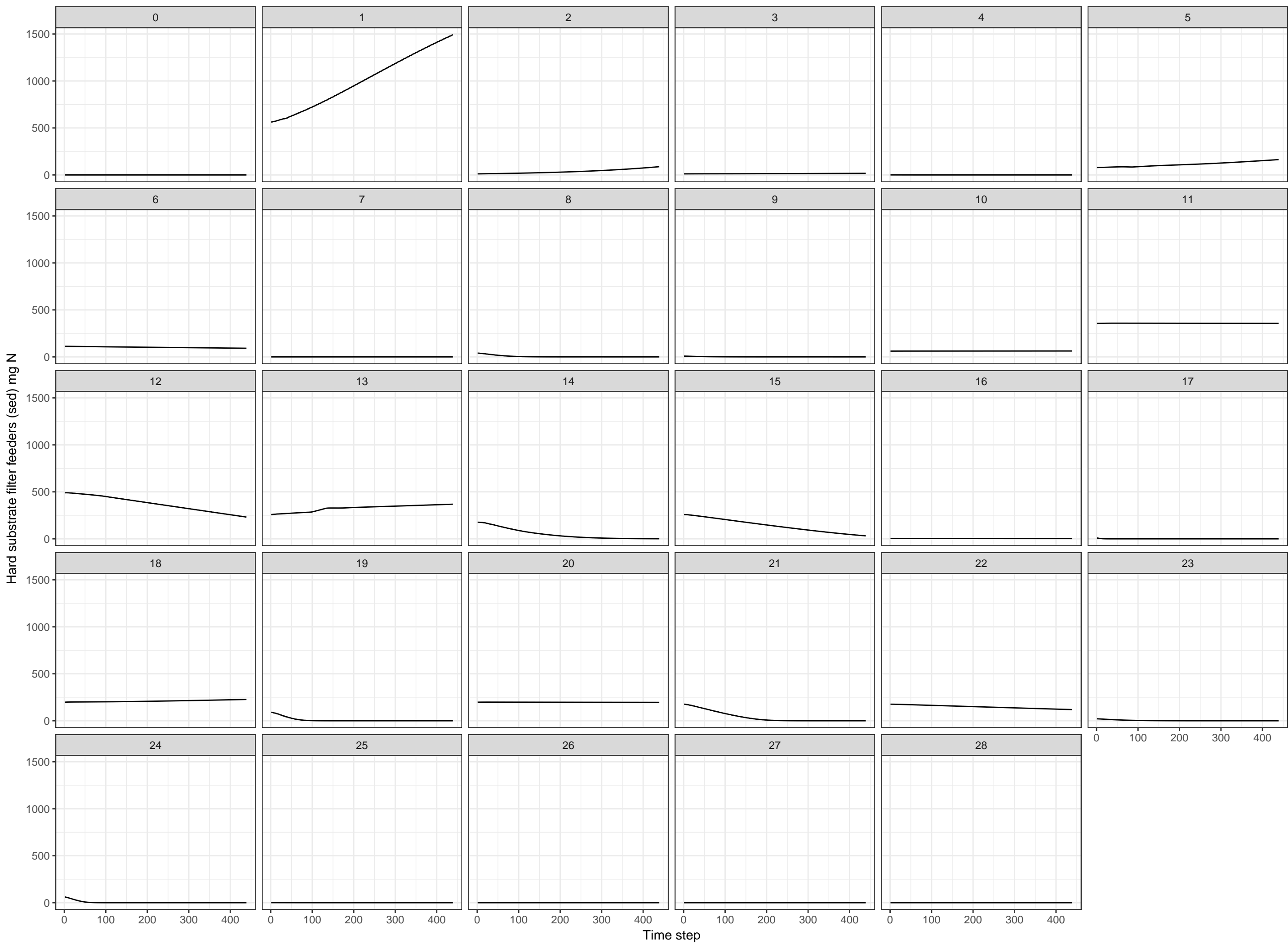

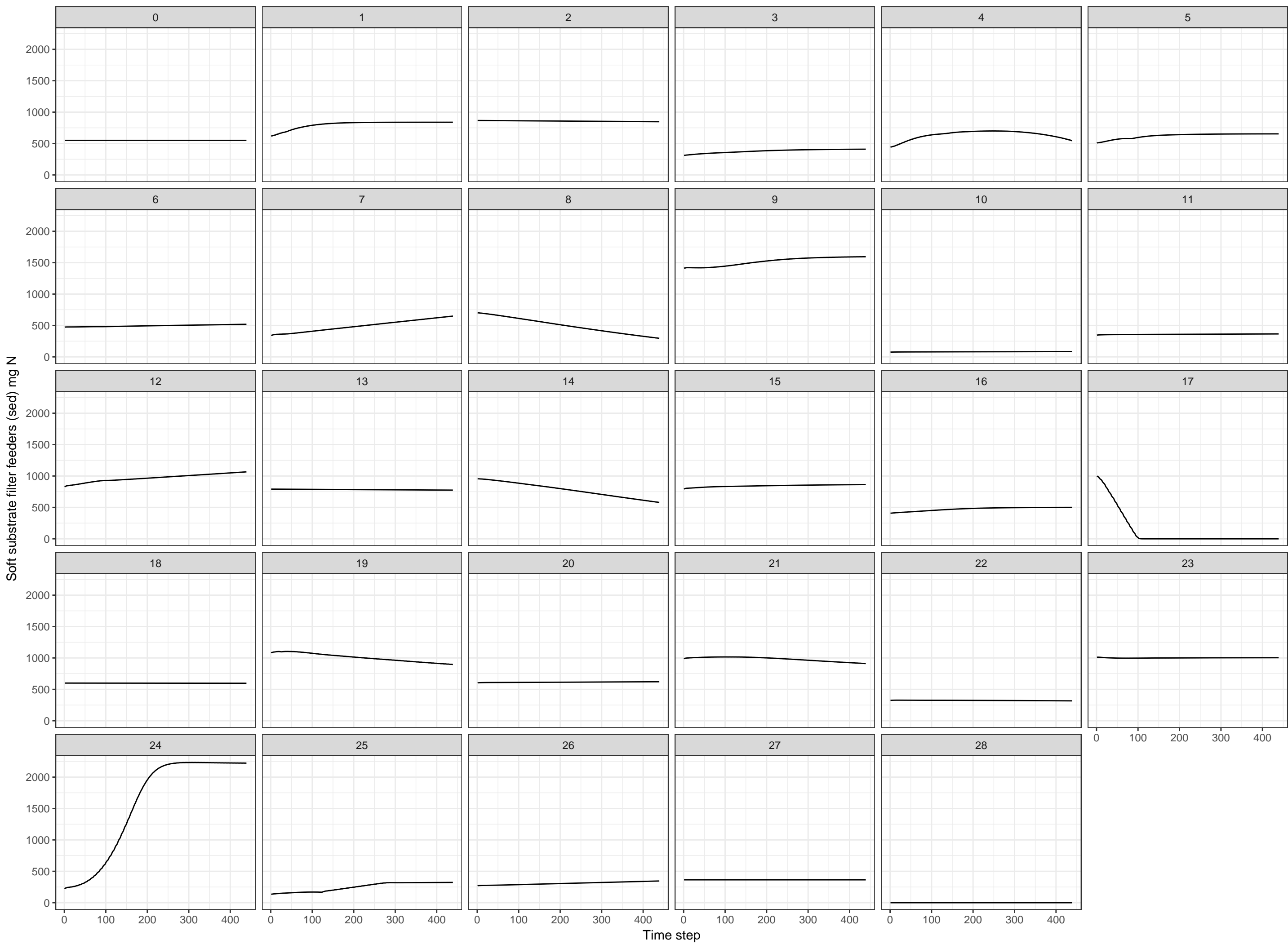

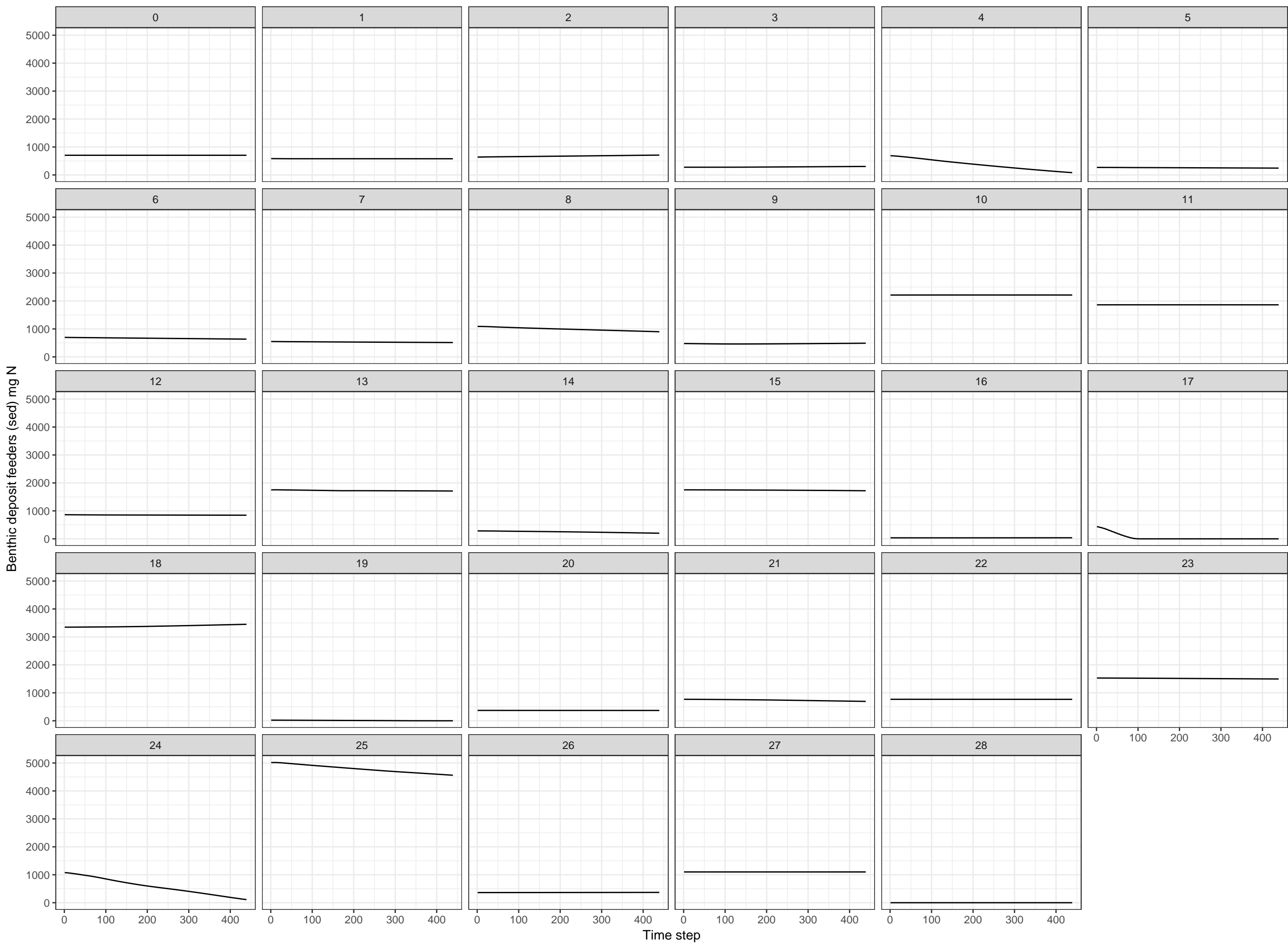

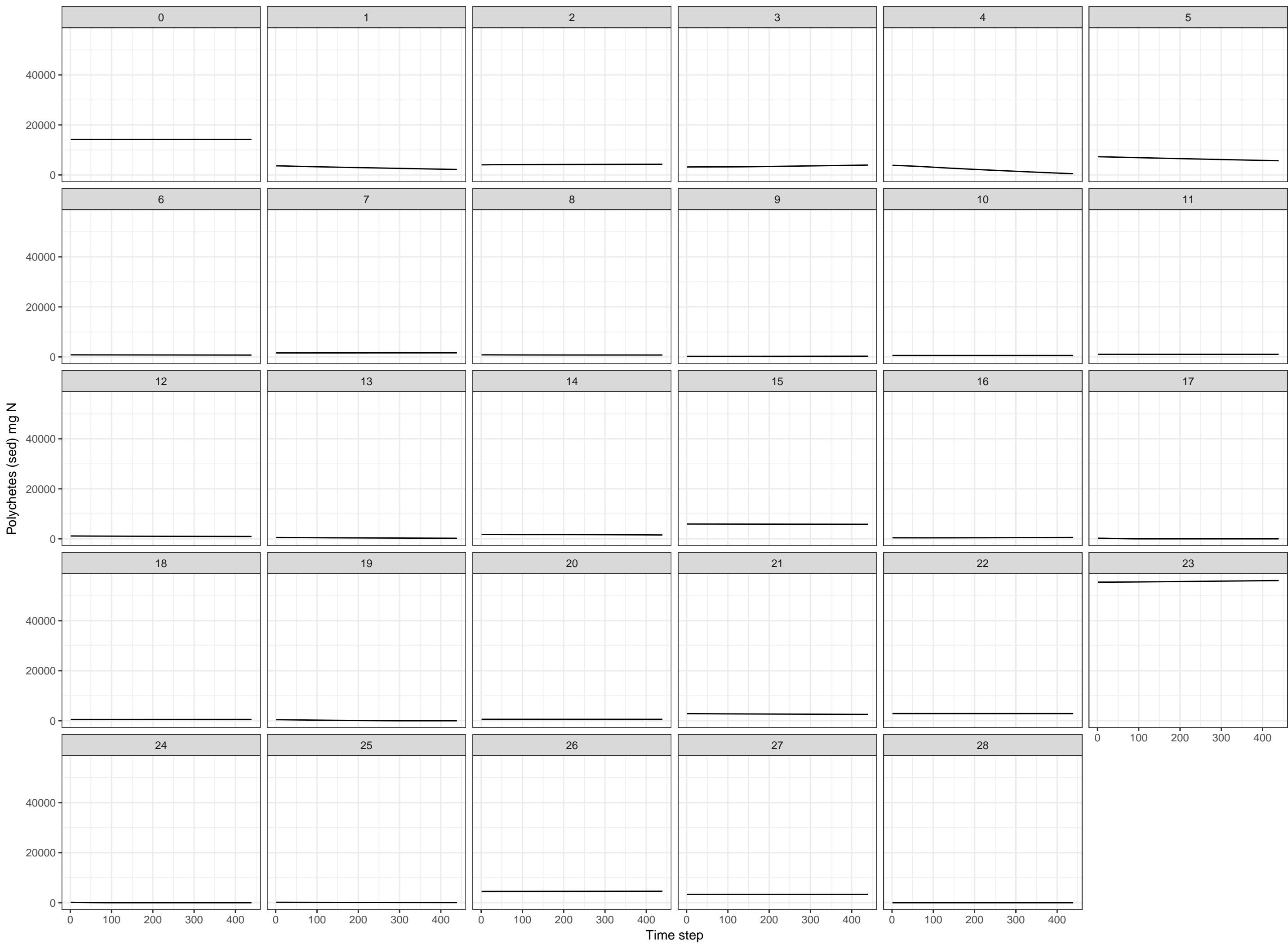

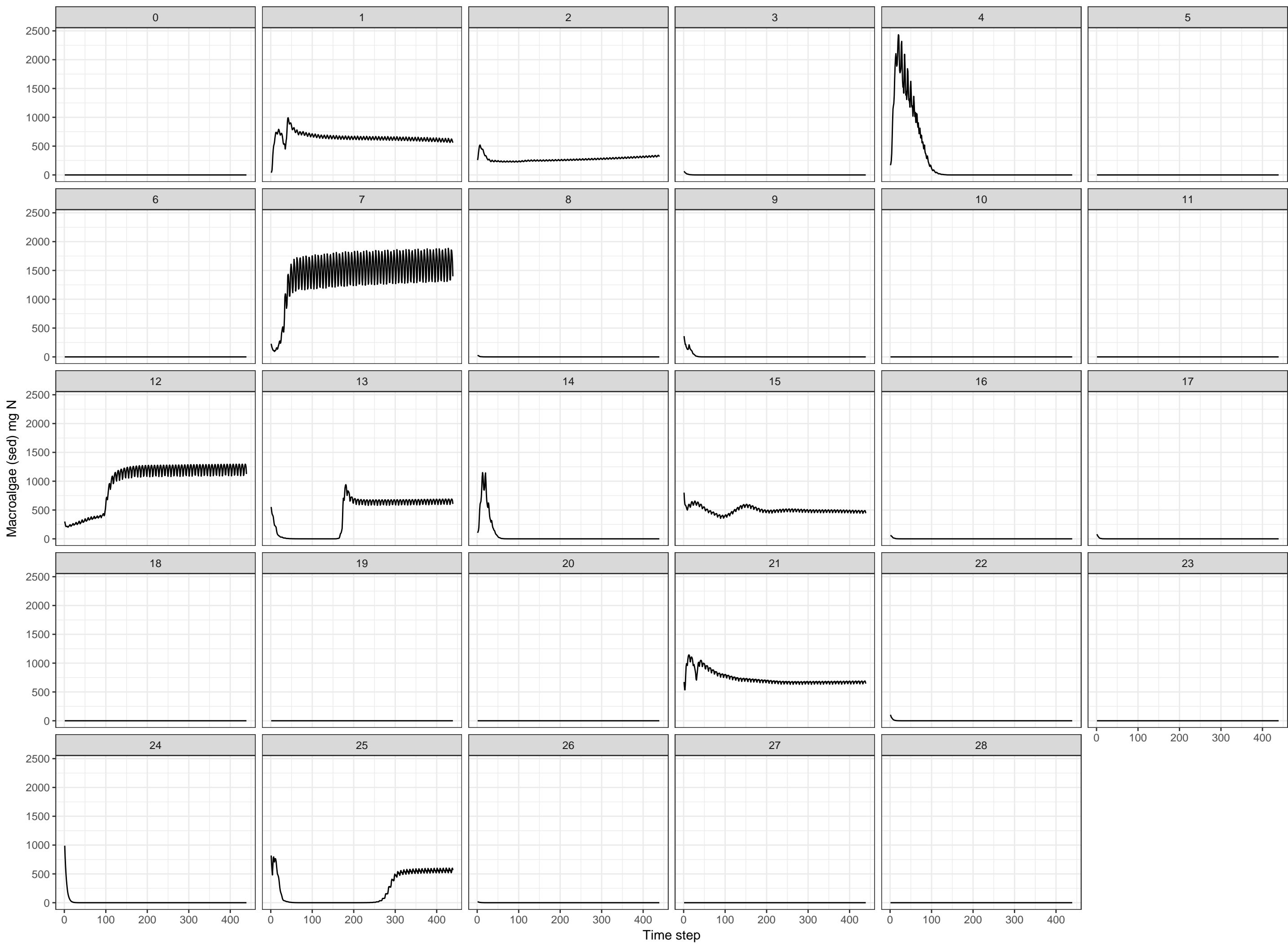

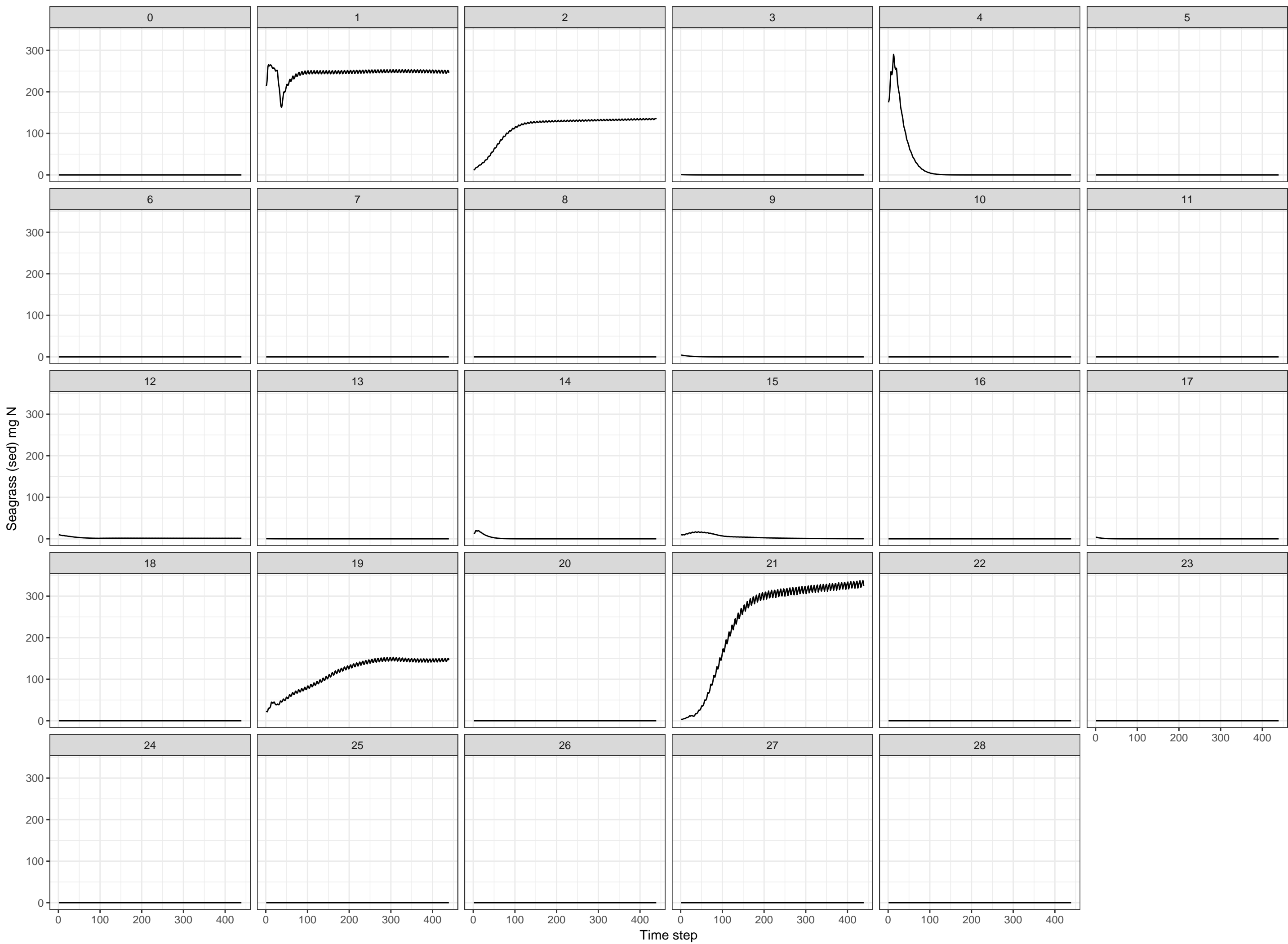

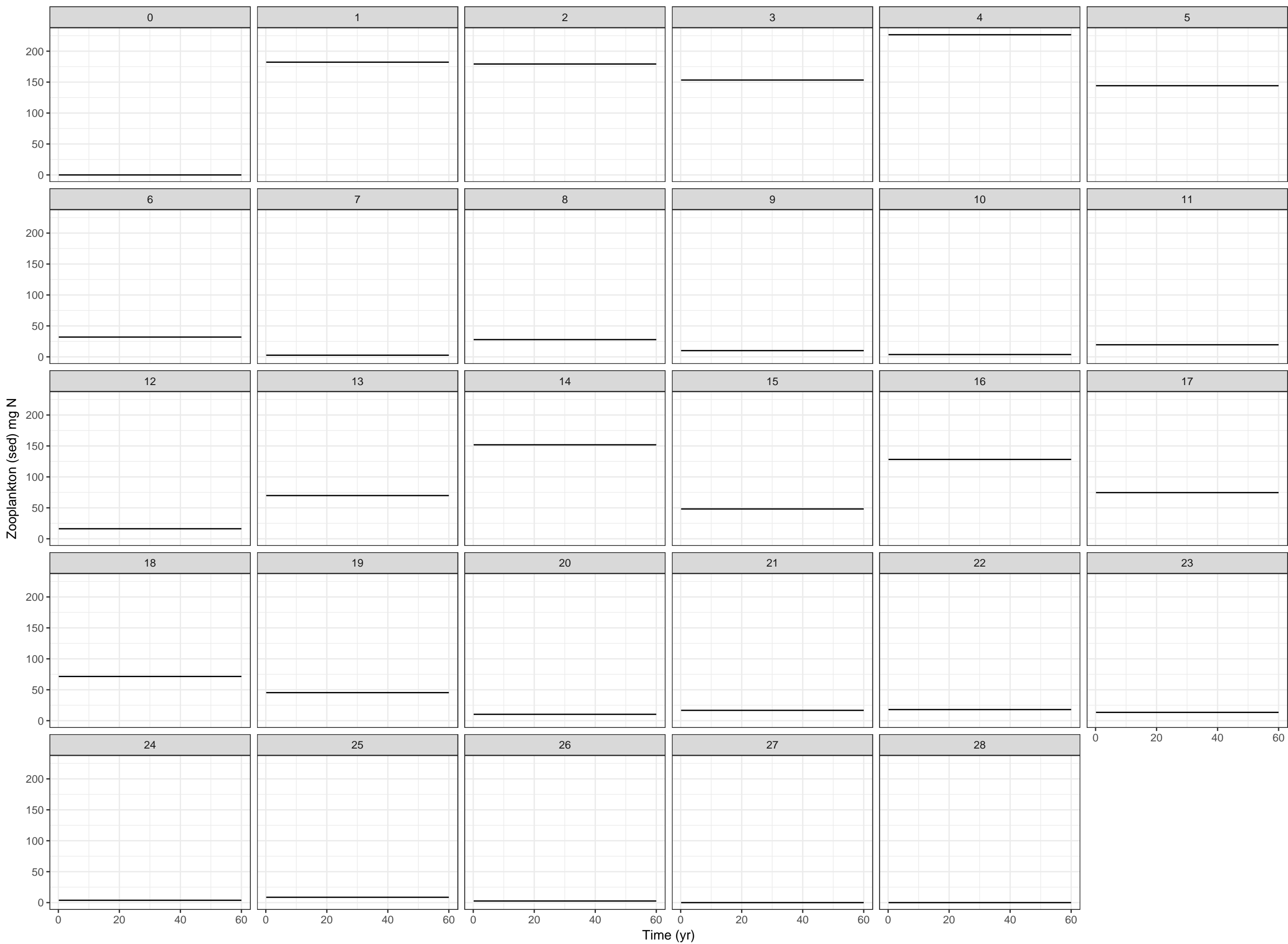

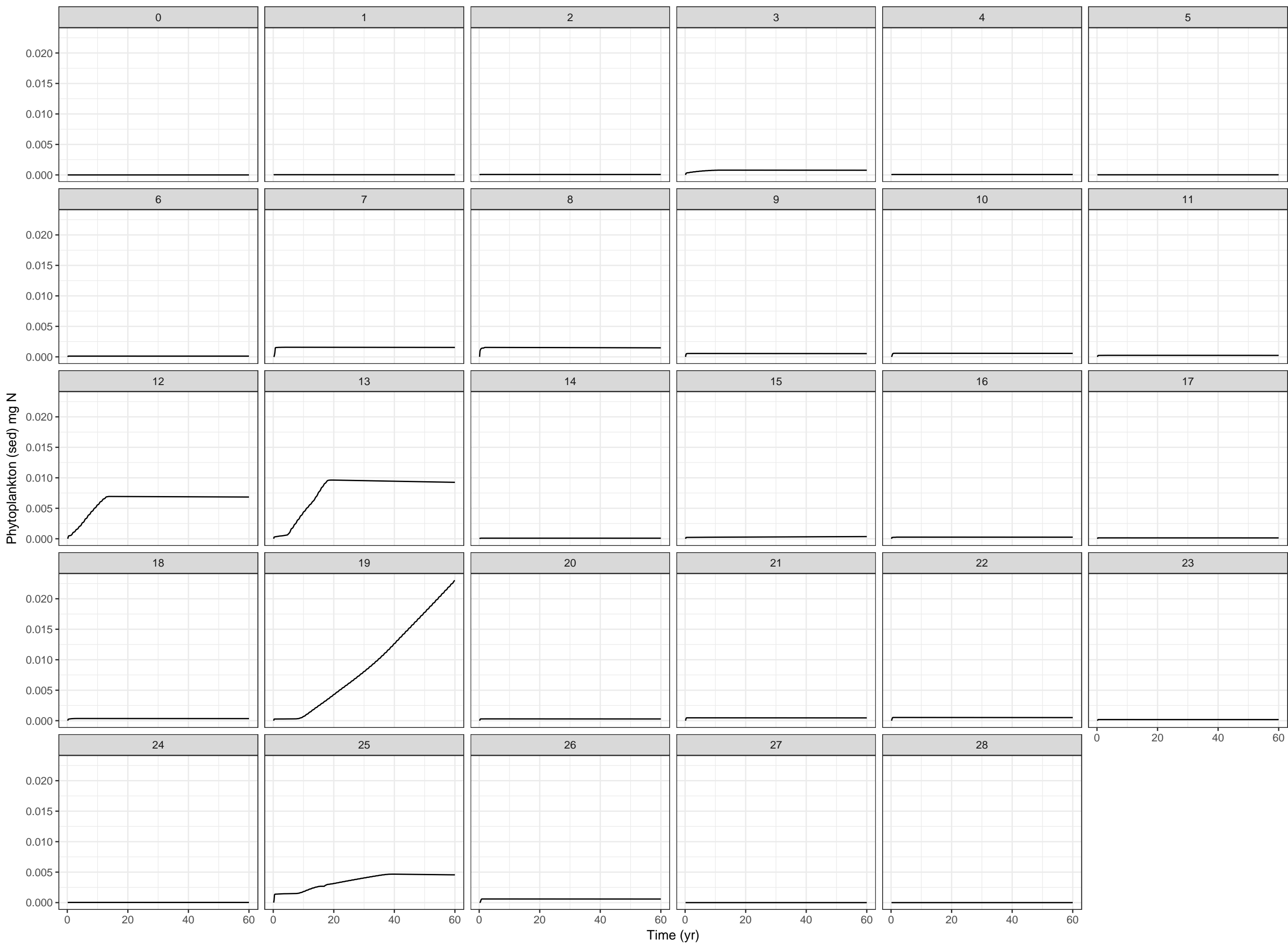

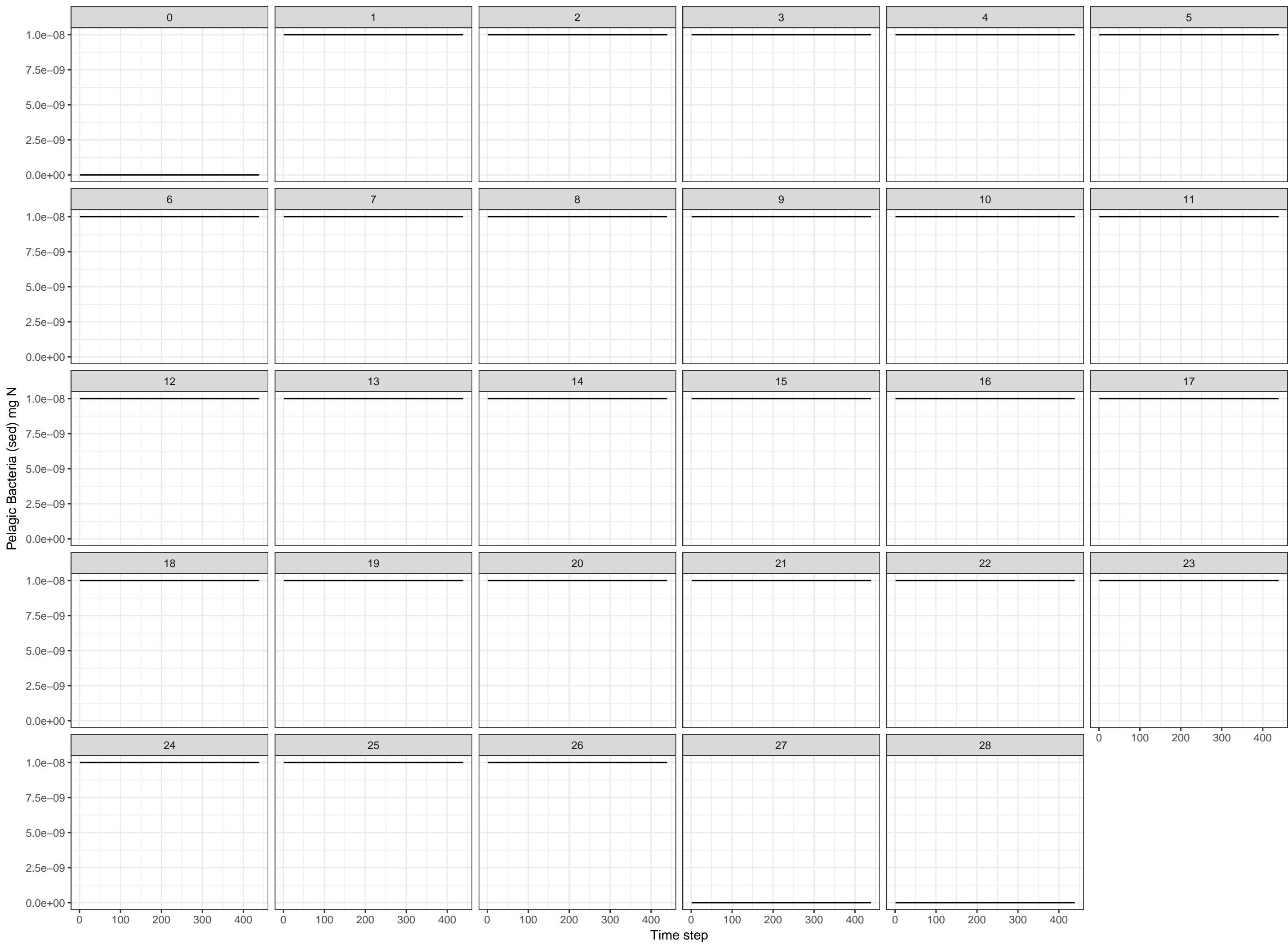

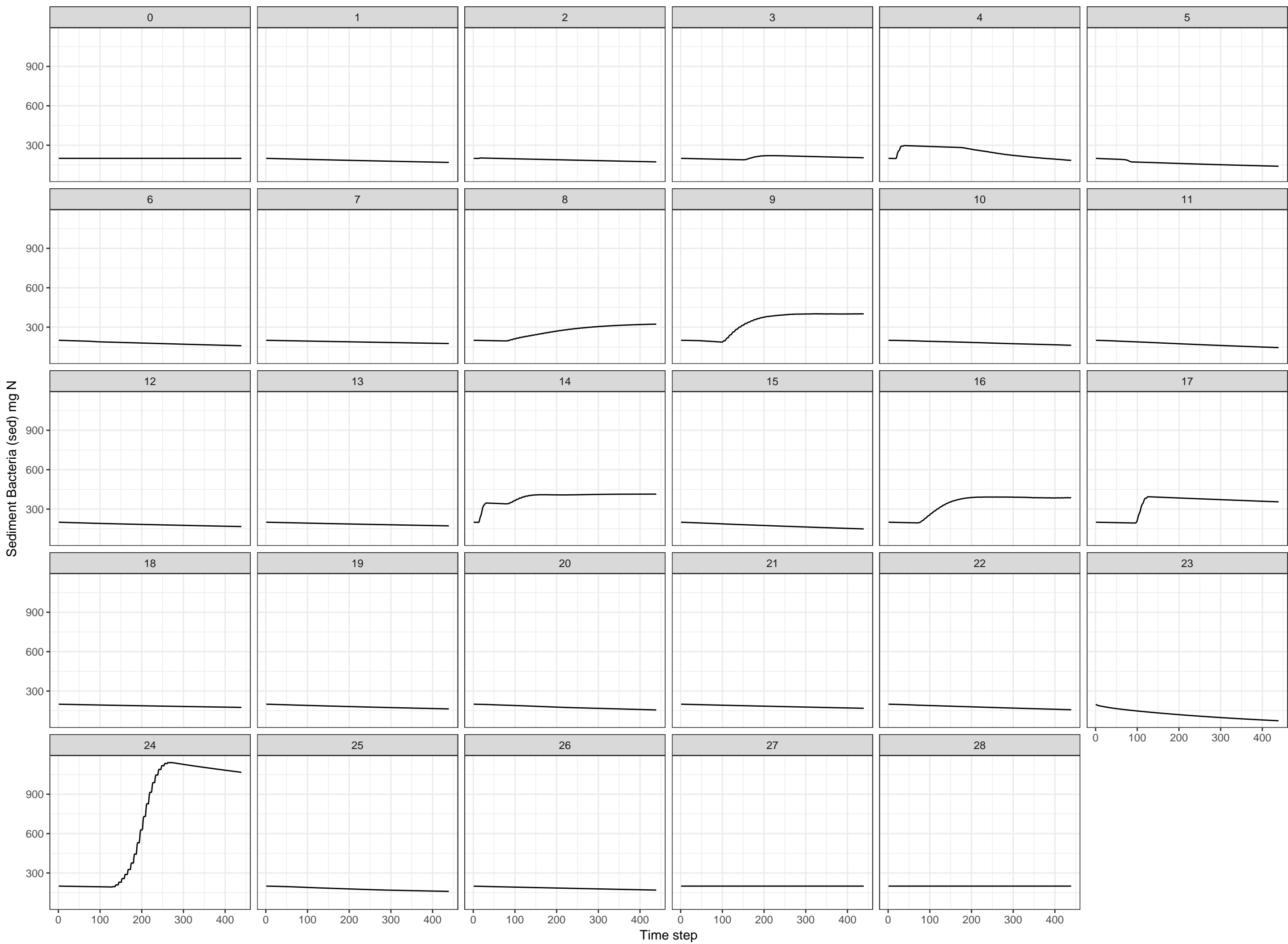

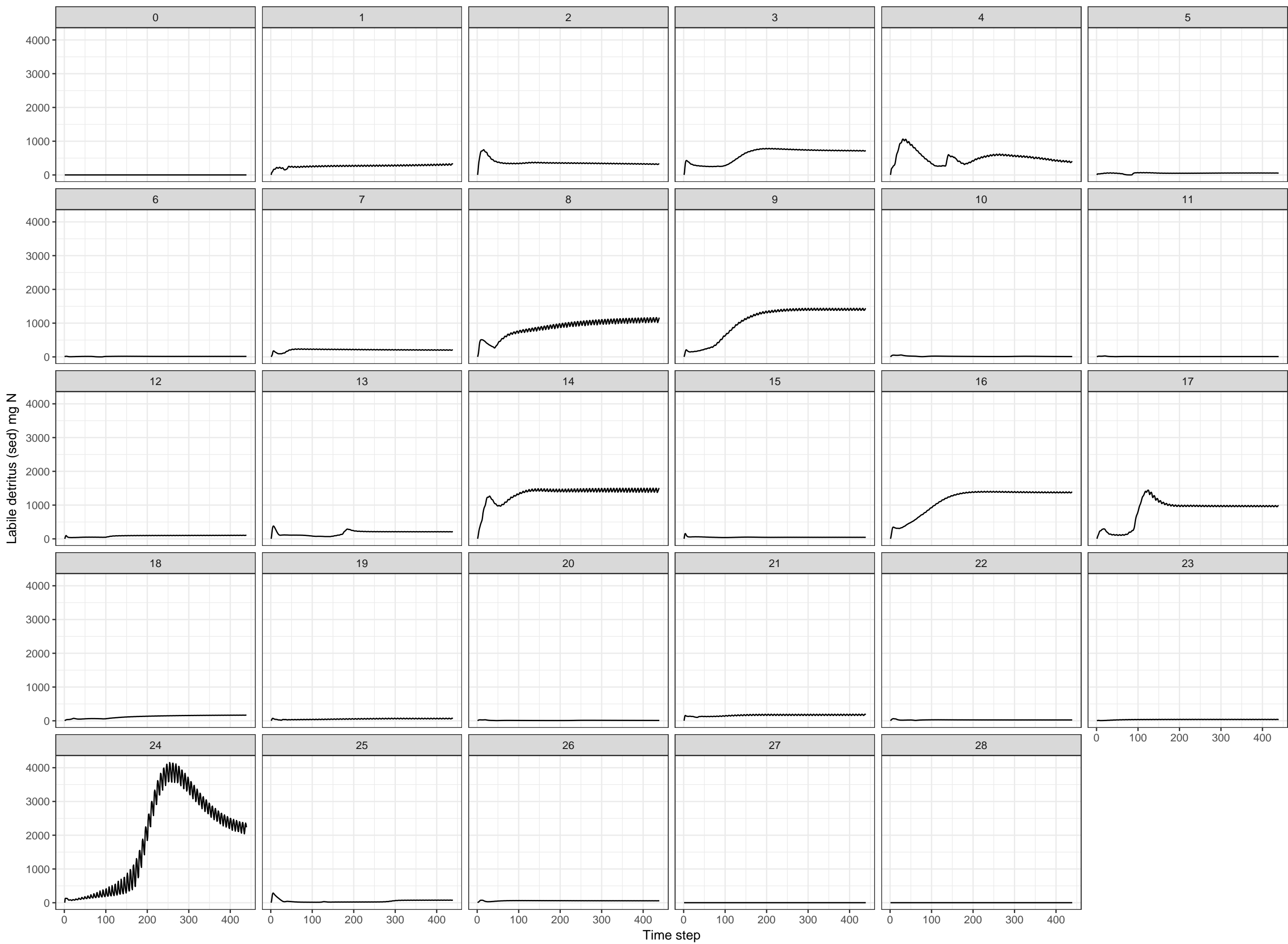

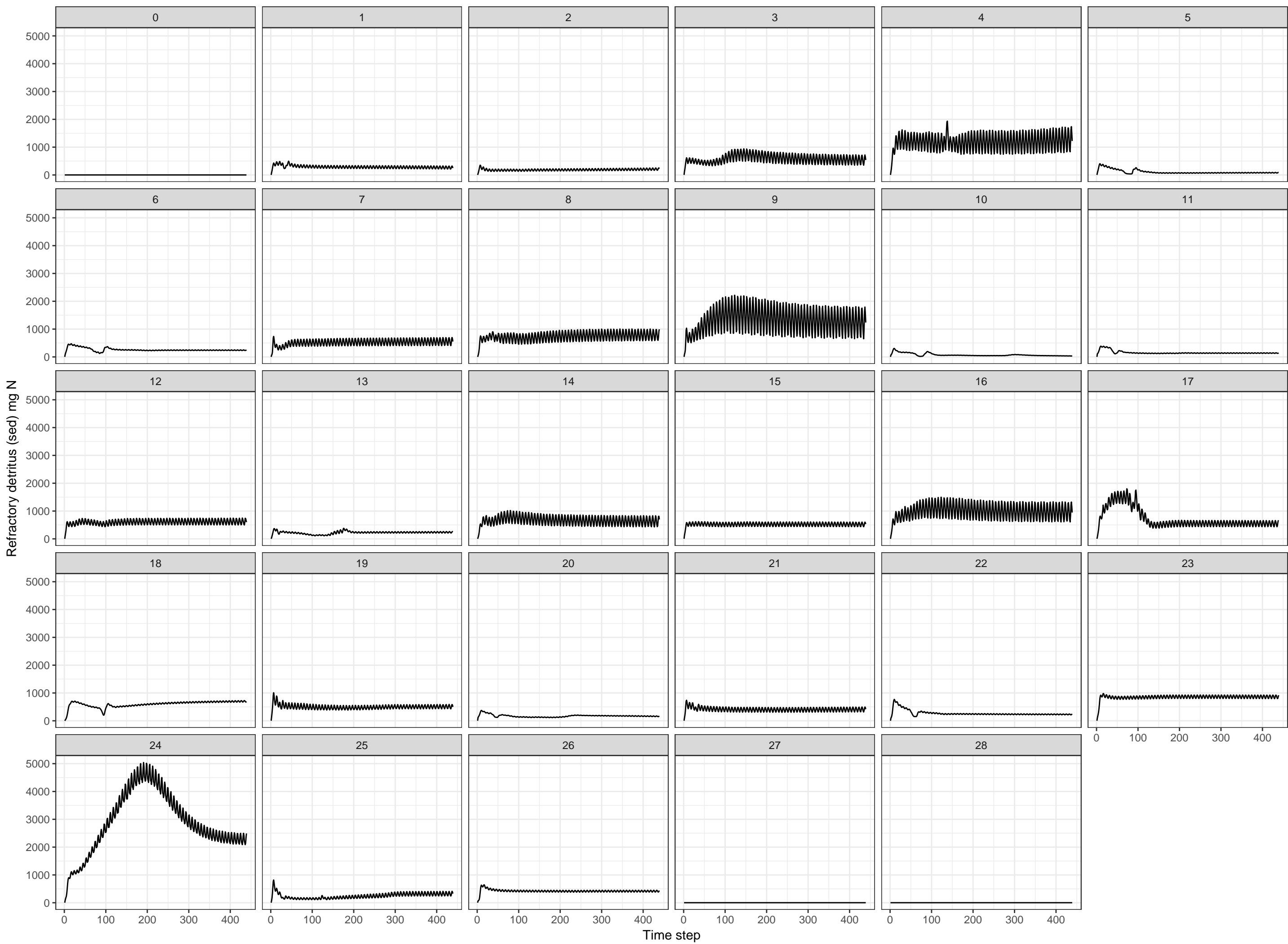

Supplement: S1 File — Supporting Information A. The Baltic Sea Atlantis: File A. Input data of the tracers per box for the Baltic Atlantis File B. Input data of the tracers per box and layer for the Baltic Atlantis File C. Input data for the fill values for the tracers for the Baltic Atlantis Figure A. Schematic diagram illustrating the structure of the coupled HBM-ERGOM model system Figure B. 120 year simulation run Figure C. The FISHRENT model diagram, here applied to Kattegat and Western Baltic. Figure D. One-year cycle of Chl-a in the different polygons Figure E. Relative biomass–initial condition values compared with simulation outcome Figure F. Diet composition of all predators Figure G. Biomass per age group over time for all vertebrates Figure H. Demography distribution for all vertebrates—the number of individuals for each age group Figure I. Geographical distribution of all functional groups Figure J. Geographical distribution of oxygen in the different layers. Panel 1 = top layer, panel 7 = bottom layer Figure K. Total biomass of Cod for scenario 1 (baseline) compared to scenario 5 Figure L. Relative prey biomass for predator cod, baseline compared to scenario 5 Figure M. One-year cycle of nutrients in the different polygons Table A. Physical and geochemical parameters used to internally force the Baltic Atlantis model. Table B. Summary of riverine + direct point source waterborne nitrogen loads applied to the Baltic Atlantis grid based on information from the Review of the Fifth Baltic Sea Pollution Load Compilation for the 2013 HELCOM Ministerial Meeting (HELCOM PLC-5.5). Nitrogen fractionation between DIN and DON based on Savchuk et al. (2012). Bioavailable fraction of DON assumed equal to labile DON as in Savchuk and Wolff (2009). Coastal retention fractions from Savchuk and Wolff (2009). Table C. Summary of key sources used to inform the biological module of Baltic Atlantis in relation to abundance and biomass, demography, prey-predator interaction and other functions. Ta [file pone.0199168.s001.zip › FigI.pdf]

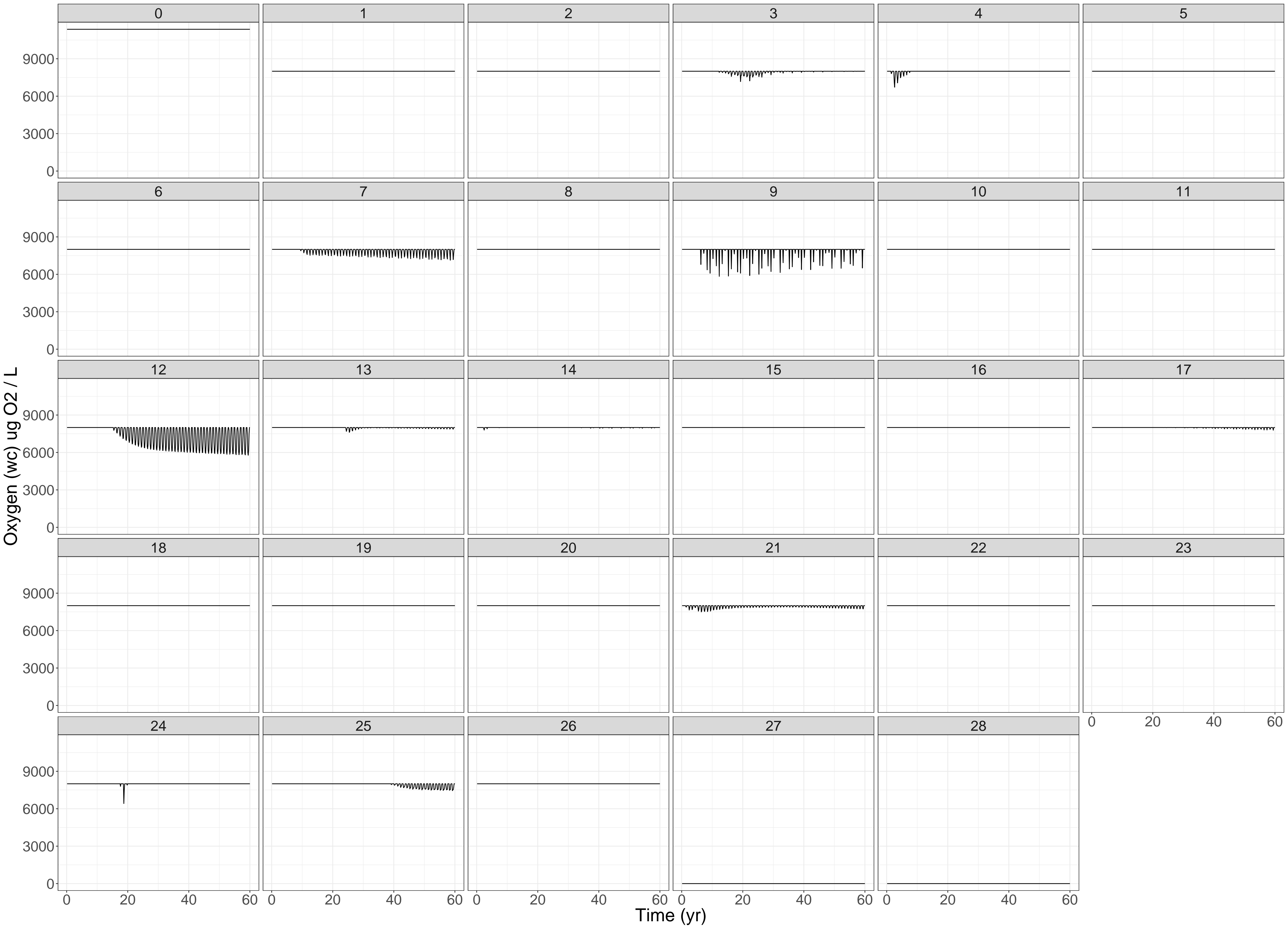

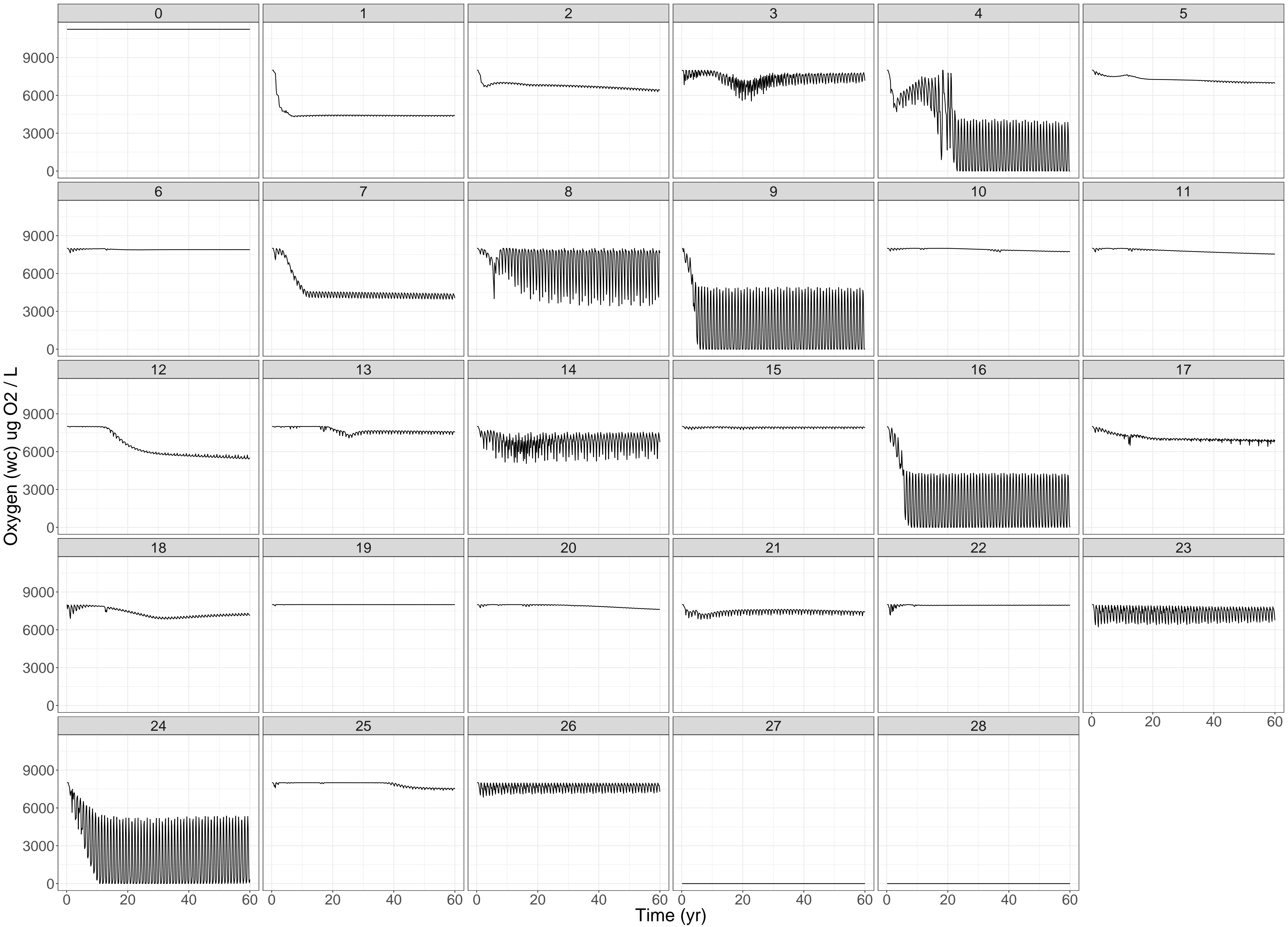

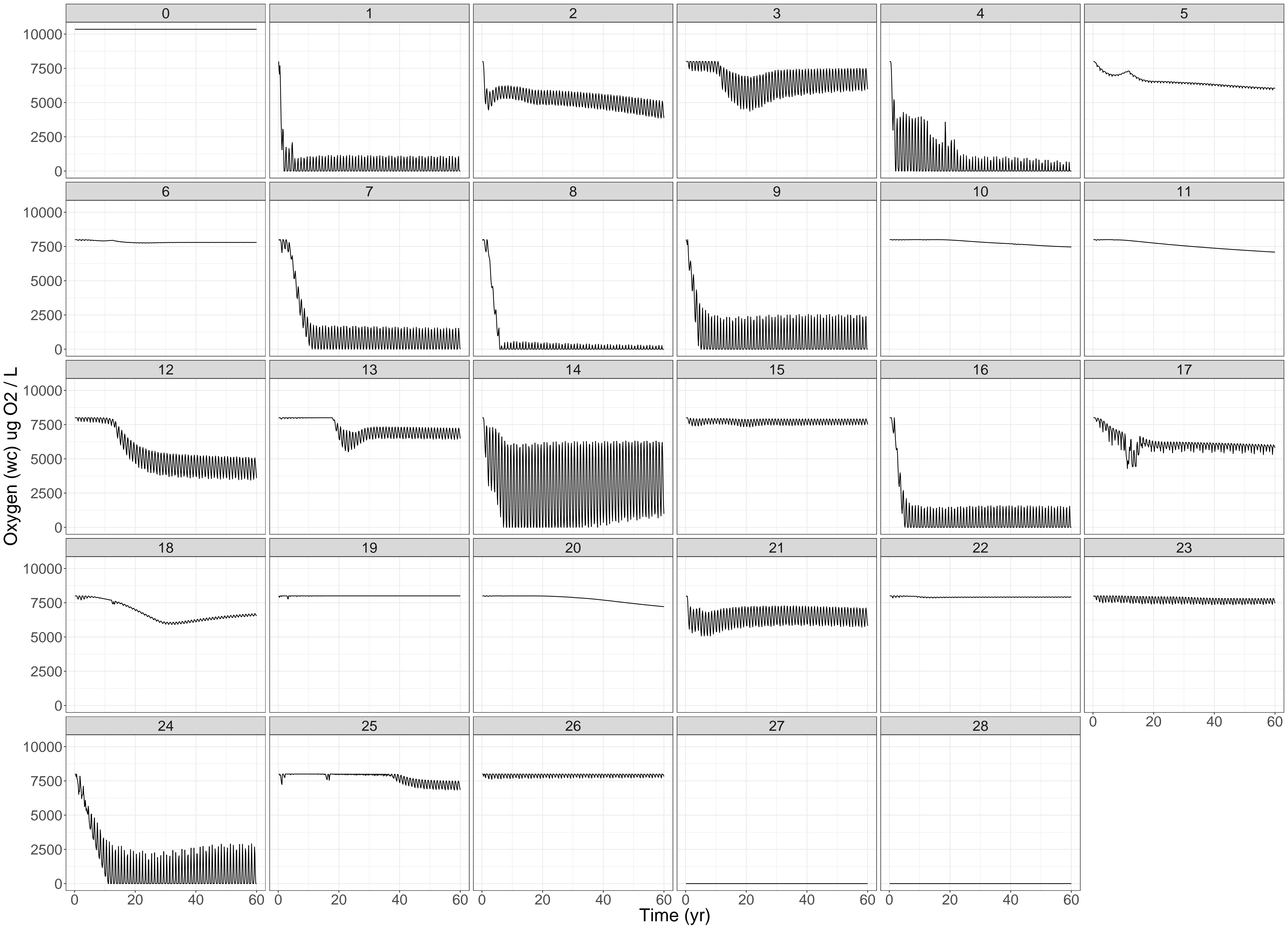

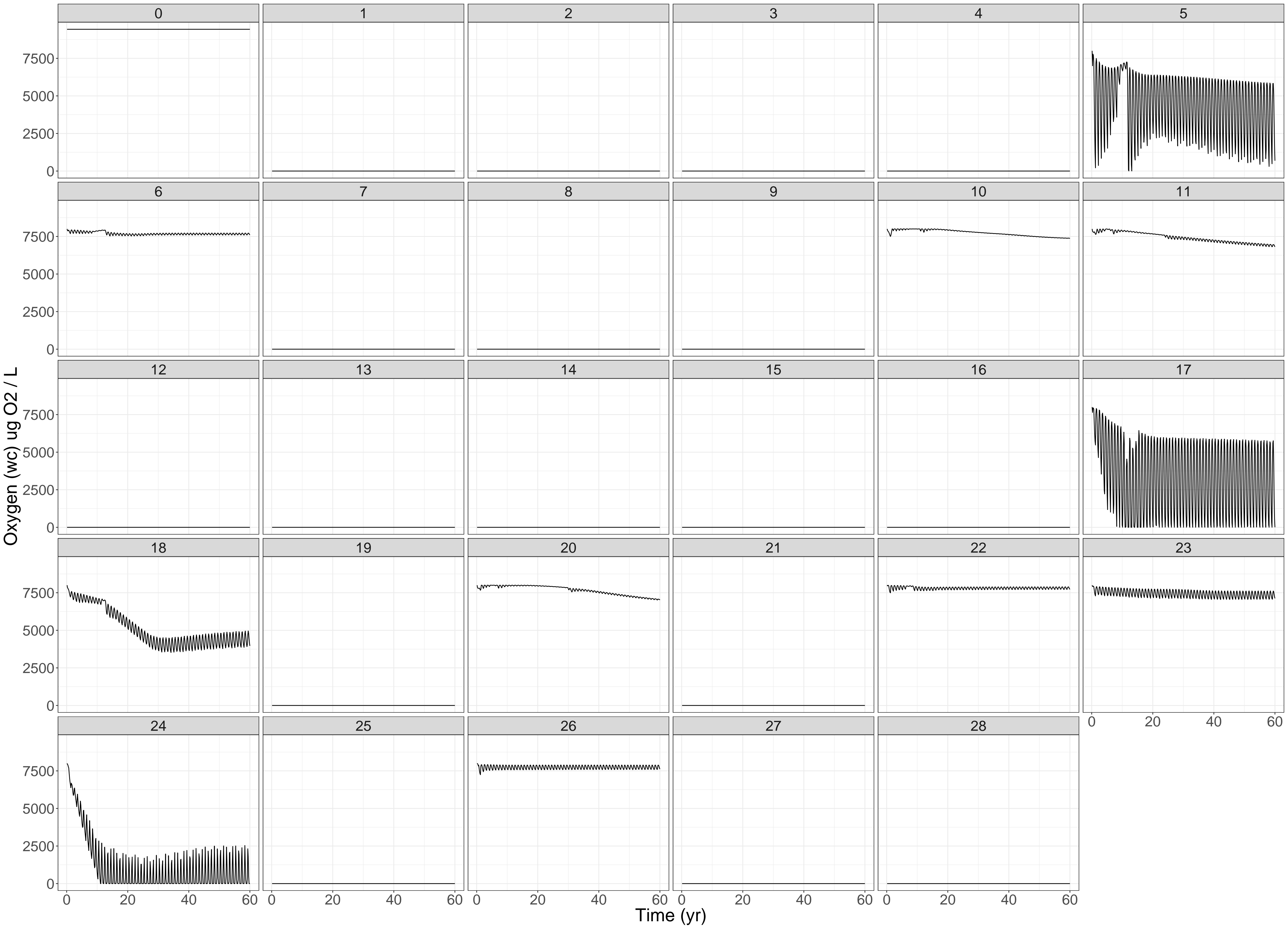

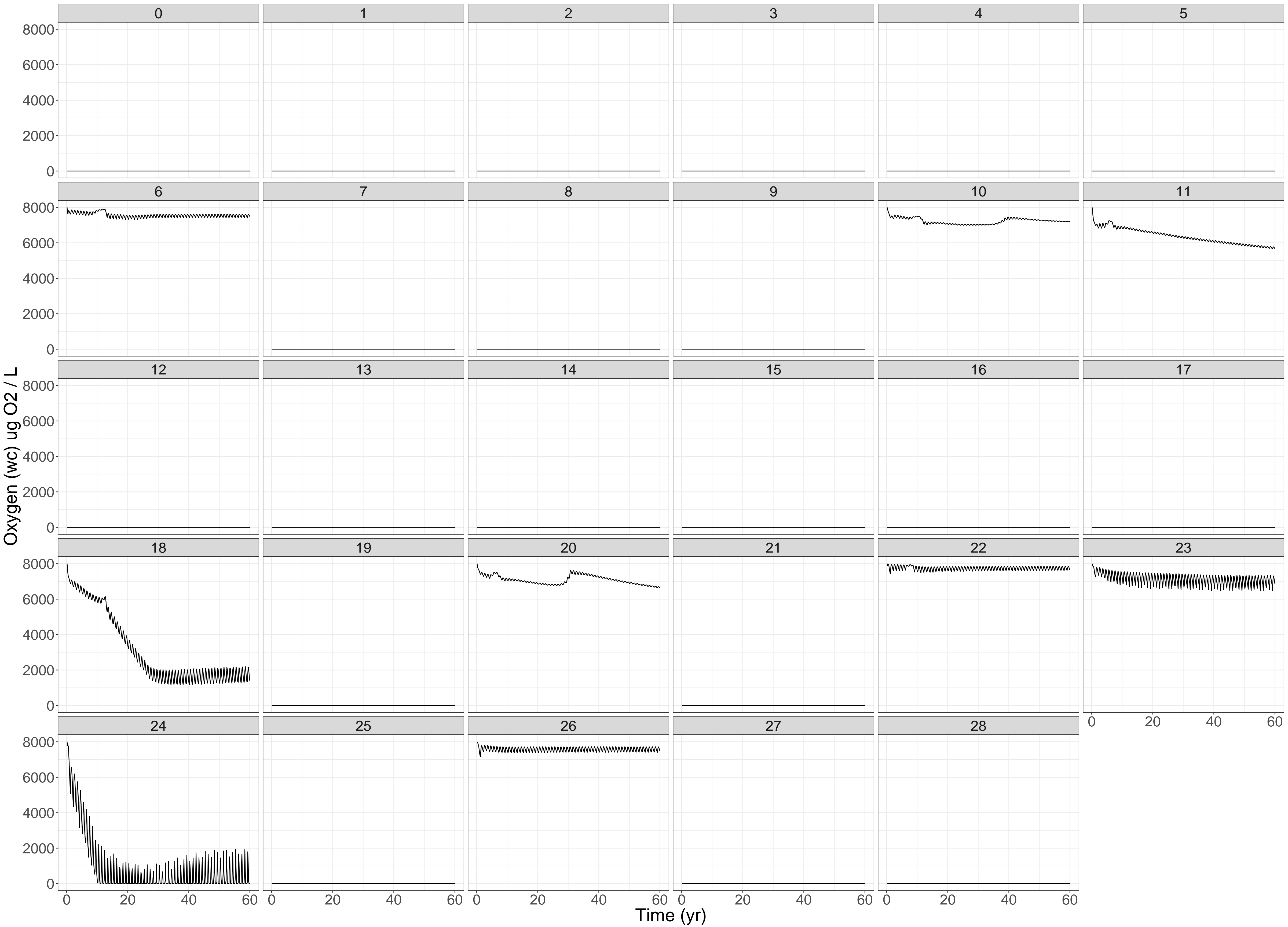

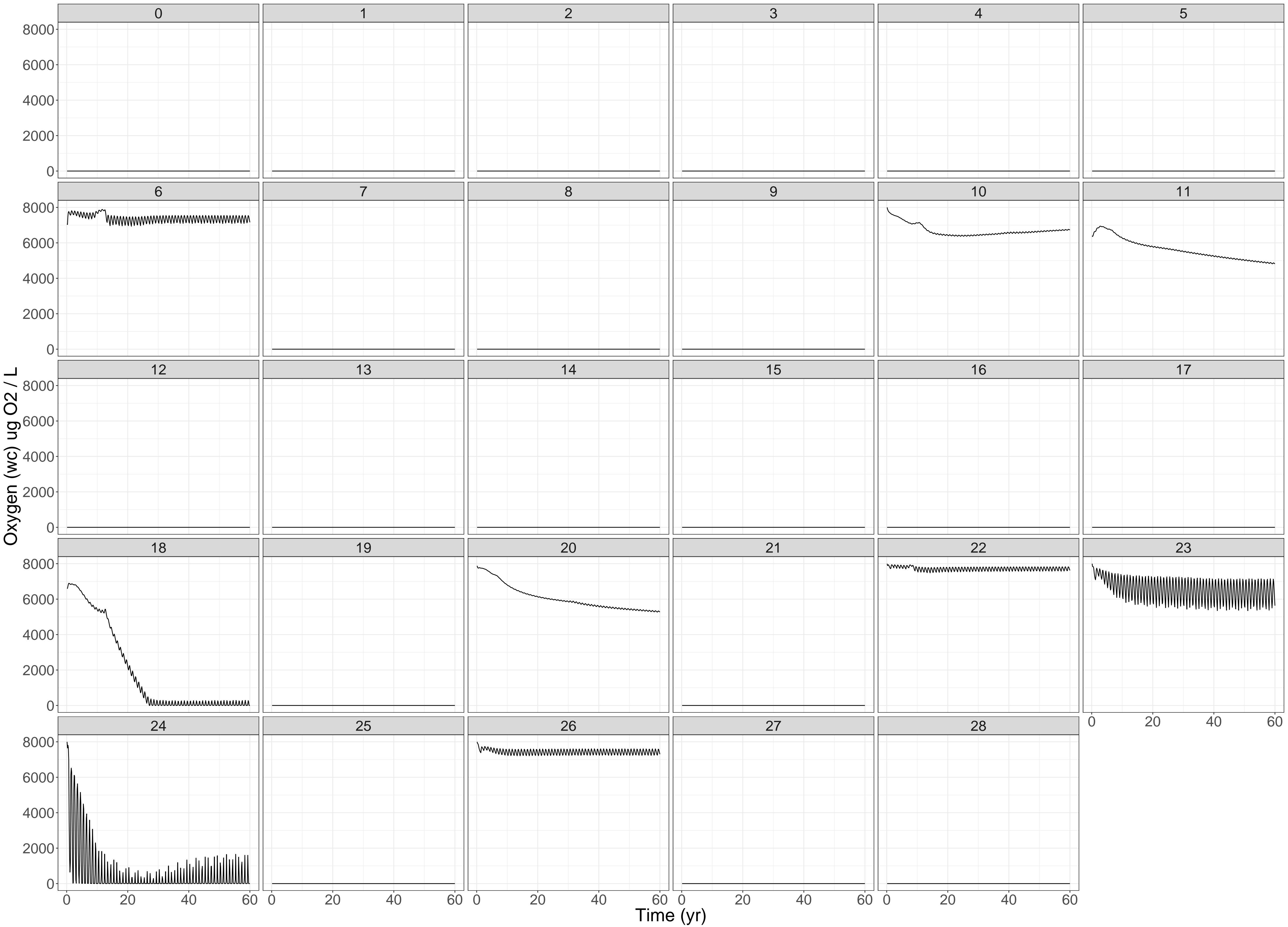

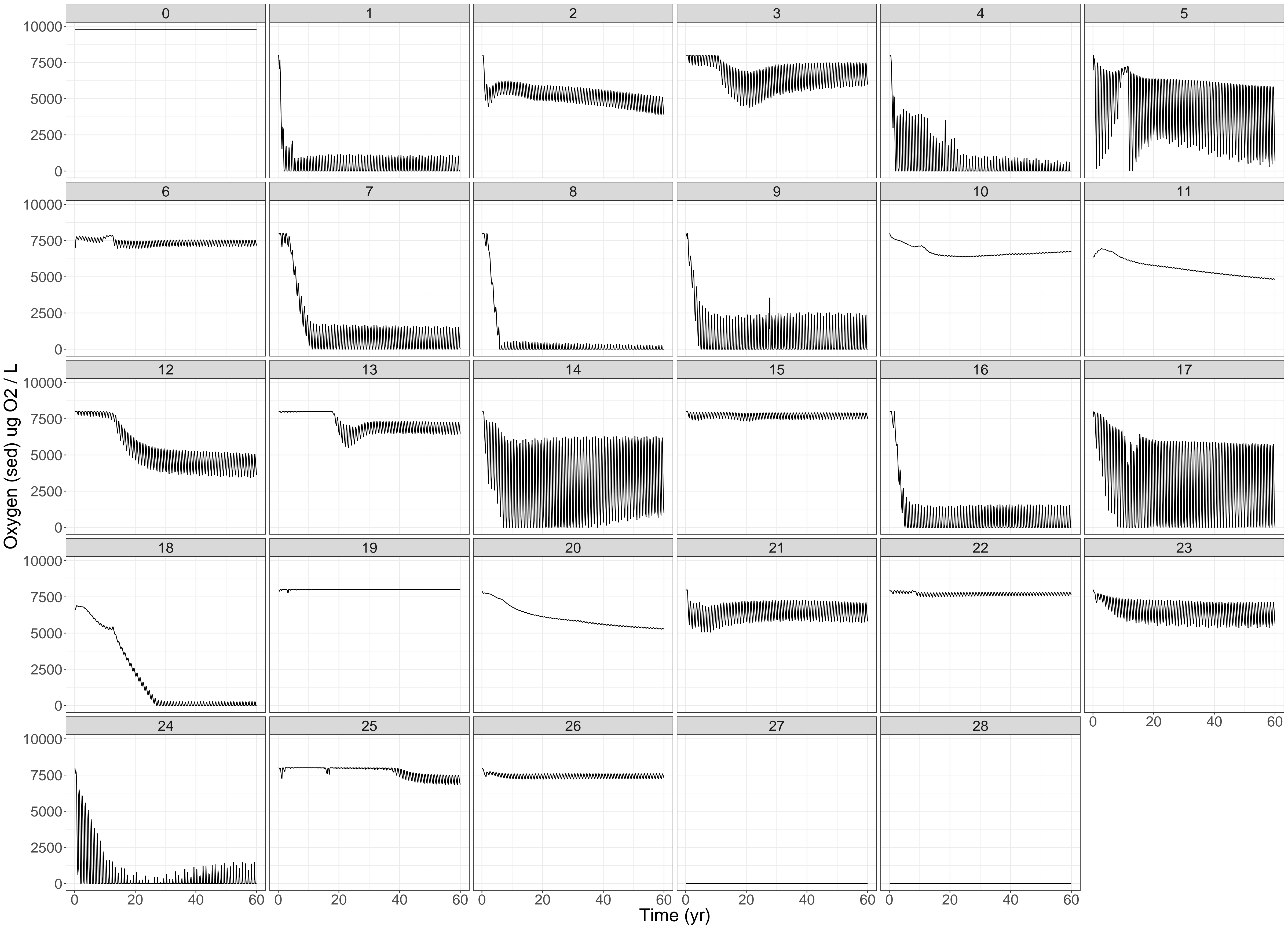

Supplement: S1 File — Supporting Information A. The Baltic Sea Atlantis: File A. Input data of the tracers per box for the Baltic Atlantis File B. Input data of the tracers per box and layer for the Baltic Atlantis File C. Input data for the fill values for the tracers for the Baltic Atlantis Figure A. Schematic diagram illustrating the structure of the coupled HBM-ERGOM model system Figure B. 120 year simulation run Figure C. The FISHRENT model diagram, here applied to Kattegat and Western Baltic. Figure D. One-year cycle of Chl-a in the different polygons Figure E. Relative biomass–initial condition values compared with simulation outcome Figure F. Diet composition of all predators Figure G. Biomass per age group over time for all vertebrates Figure H. Demography distribution for all vertebrates—the number of individuals for each age group Figure I. Geographical distribution of all functional groups Figure J. Geographical distribution of oxygen in the different layers. Panel 1 = top layer, panel 7 = bottom layer Figure K. Total biomass of Cod for scenario 1 (baseline) compared to scenario 5 Figure L. Relative prey biomass for predator cod, baseline compared to scenario 5 Figure M. One-year cycle of nutrients in the different polygons Table A. Physical and geochemical parameters used to internally force the Baltic Atlantis model. Table B. Summary of riverine + direct point source waterborne nitrogen loads applied to the Baltic Atlantis grid based on information from the Review of the Fifth Baltic Sea Pollution Load Compilation for the 2013 HELCOM Ministerial Meeting (HELCOM PLC-5.5). Nitrogen fractionation between DIN and DON based on Savchuk et al. (2012). Bioavailable fraction of DON assumed equal to labile DON as in Savchuk and Wolff (2009). Coastal retention fractions from Savchuk and Wolff (2009). Table C. Summary of key sources used to inform the biological module of Baltic Atlantis in relation to abundance and biomass, demography, prey-predator interaction and other functions. Ta [file pone.0199168.s001.zip › FigJ.pdf]

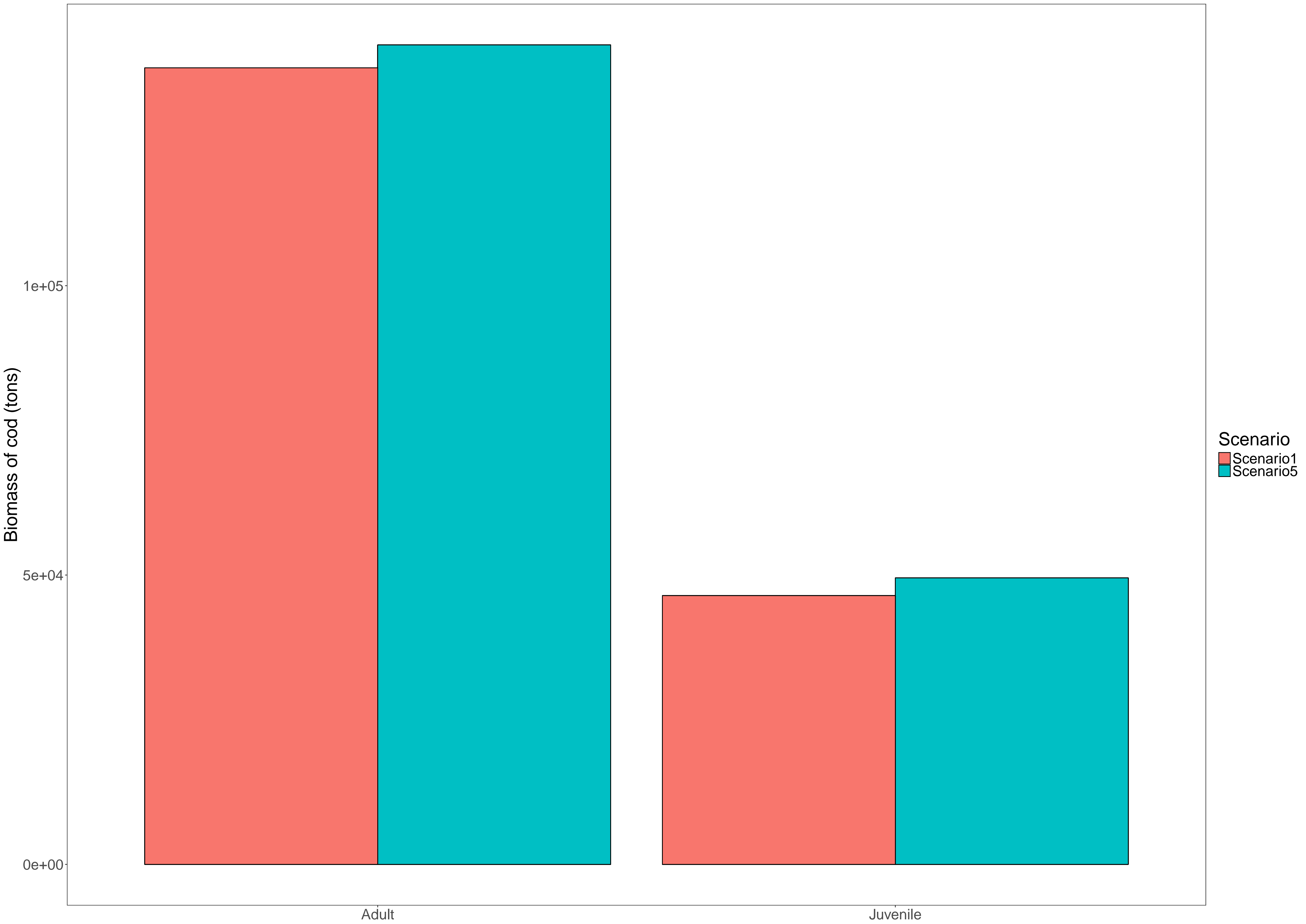

Supplement: S1 File — Supporting Information A. The Baltic Sea Atlantis: File A. Input data of the tracers per box for the Baltic Atlantis File B. Input data of the tracers per box and layer for the Baltic Atlantis File C. Input data for the fill values for the tracers for the Baltic Atlantis Figure A. Schematic diagram illustrating the structure of the coupled HBM-ERGOM model system Figure B. 120 year simulation run Figure C. The FISHRENT model diagram, here applied to Kattegat and Western Baltic. Figure D. One-year cycle of Chl-a in the different polygons Figure E. Relative biomass–initial condition values compared with simulation outcome Figure F. Diet composition of all predators Figure G. Biomass per age group over time for all vertebrates Figure H. Demography distribution for all vertebrates—the number of individuals for each age group Figure I. Geographical distribution of all functional groups Figure J. Geographical distribution of oxygen in the different layers. Panel 1 = top layer, panel 7 = bottom layer Figure K. Total biomass of Cod for scenario 1 (baseline) compared to scenario 5 Figure L. Relative prey biomass for predator cod, baseline compared to scenario 5 Figure M. One-year cycle of nutrients in the different polygons Table A. Physical and geochemical parameters used to internally force the Baltic Atlantis model. Table B. Summary of riverine + direct point source waterborne nitrogen loads applied to the Baltic Atlantis grid based on information from the Review of the Fifth Baltic Sea Pollution Load Compilation for the 2013 HELCOM Ministerial Meeting (HELCOM PLC-5.5). Nitrogen fractionation between DIN and DON based on Savchuk et al. (2012). Bioavailable fraction of DON assumed equal to labile DON as in Savchuk and Wolff (2009). Coastal retention fractions from Savchuk and Wolff (2009). Table C. Summary of key sources used to inform the biological module of Baltic Atlantis in relation to abundance and biomass, demography, prey-predator interaction and other functions. Ta [file pone.0199168.s001.zip › FigK.pdf]

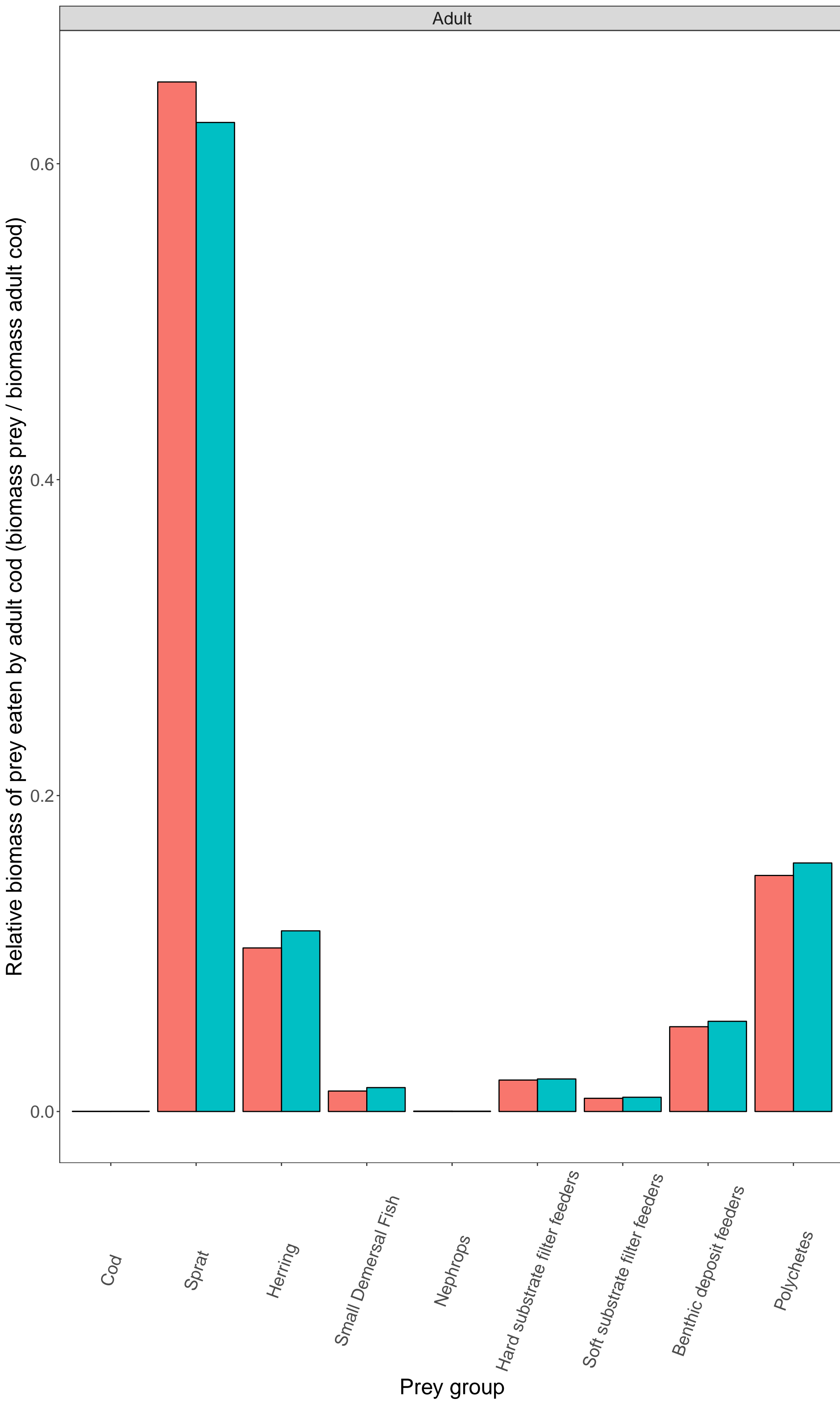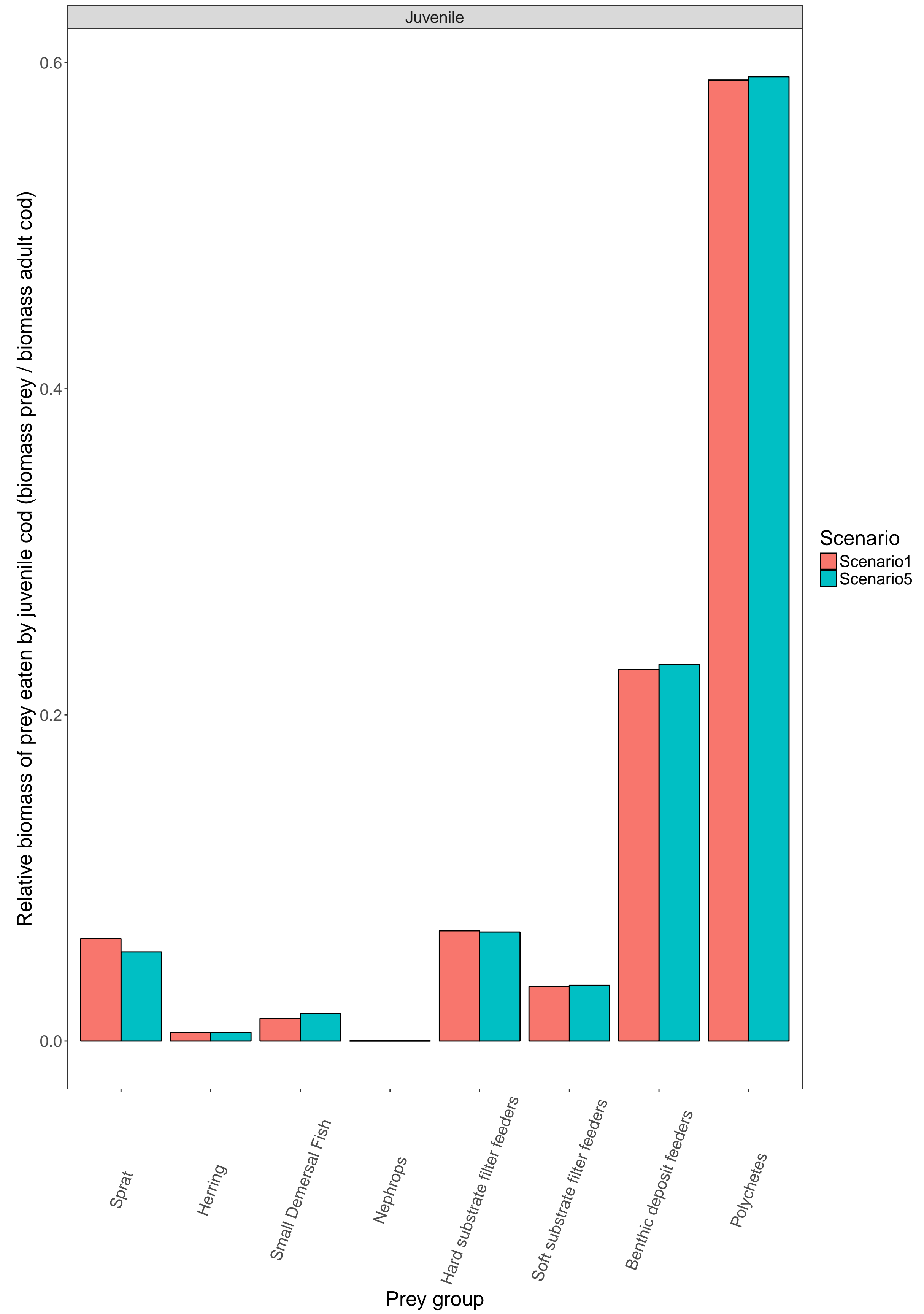

Supplement: S1 File — Supporting Information A. The Baltic Sea Atlantis: File A. Input data of the tracers per box for the Baltic Atlantis File B. Input data of the tracers per box and layer for the Baltic Atlantis File C. Input data for the fill values for the tracers for the Baltic Atlantis Figure A. Schematic diagram illustrating the structure of the coupled HBM-ERGOM model system Figure B. 120 year simulation run Figure C. The FISHRENT model diagram, here applied to Kattegat and Western Baltic. Figure D. One-year cycle of Chl-a in the different polygons Figure E. Relative biomass–initial condition values compared with simulation outcome Figure F. Diet composition of all predators Figure G. Biomass per age group over time for all vertebrates Figure H. Demography distribution for all vertebrates—the number of individuals for each age group Figure I. Geographical distribution of all functional groups Figure J. Geographical distribution of oxygen in the different layers. Panel 1 = top layer, panel 7 = bottom layer Figure K. Total biomass of Cod for scenario 1 (baseline) compared to scenario 5 Figure L. Relative prey biomass for predator cod, baseline compared to scenario 5 Figure M. One-year cycle of nutrients in the different polygons Table A. Physical and geochemical parameters used to internally force the Baltic Atlantis model. Table B. Summary of riverine + direct point source waterborne nitrogen loads applied to the Baltic Atlantis grid based on information from the Review of the Fifth Baltic Sea Pollution Load Compilation for the 2013 HELCOM Ministerial Meeting (HELCOM PLC-5.5). Nitrogen fractionation between DIN and DON based on Savchuk et al. (2012). Bioavailable fraction of DON assumed equal to labile DON as in Savchuk and Wolff (2009). Coastal retention fractions from Savchuk and Wolff (2009). Table C. Summary of key sources used to inform the biological module of Baltic Atlantis in relation to abundance and biomass, demography, prey-predator interaction and other functions. Ta [file pone.0199168.s001.zip › FigL.pdf]

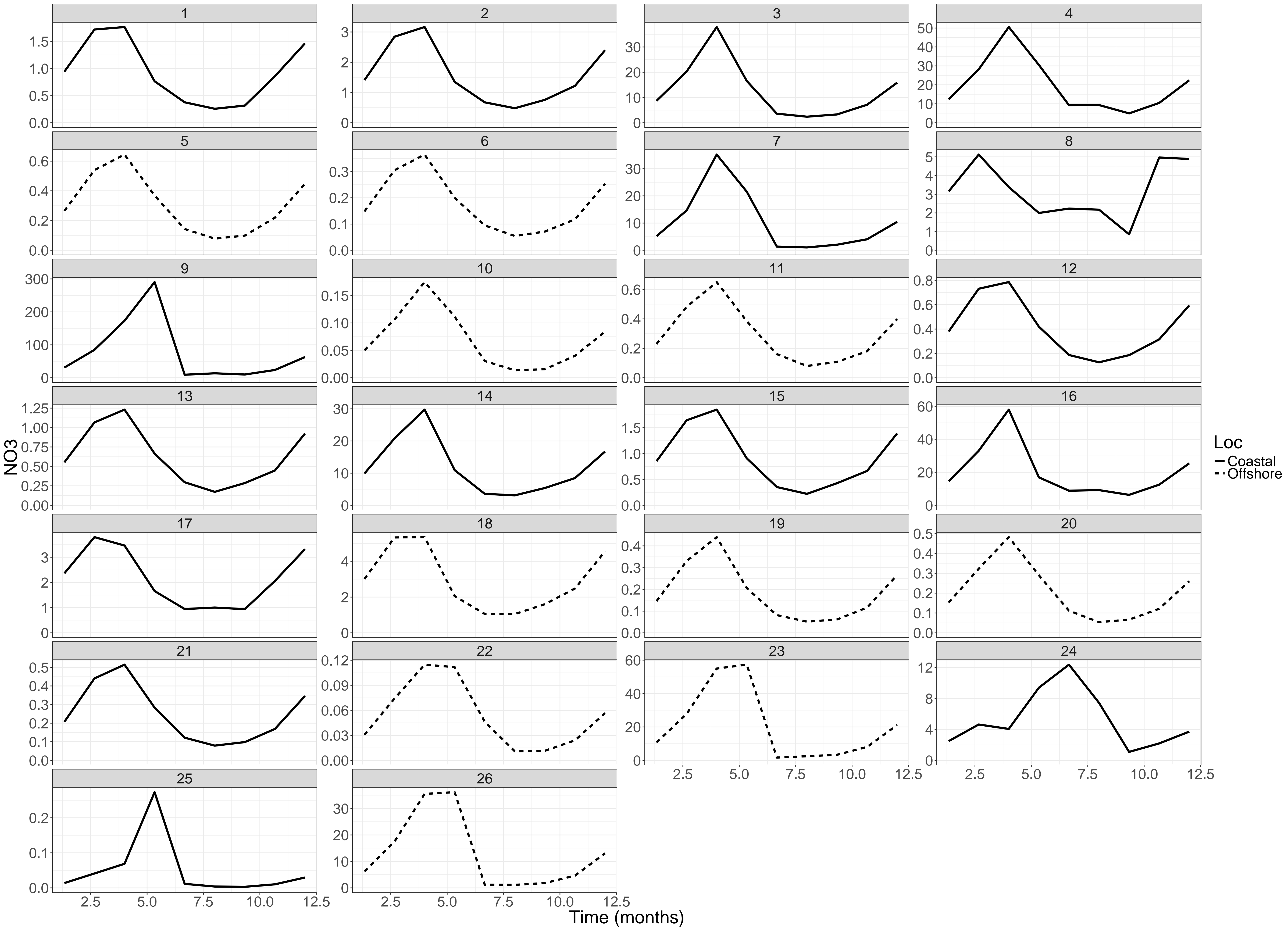

Supplement: S1 File — Supporting Information A. The Baltic Sea Atlantis: File A. Input data of the tracers per box for the Baltic Atlantis File B. Input data of the tracers per box and layer for the Baltic Atlantis File C. Input data for the fill values for the tracers for the Baltic Atlantis Figure A. Schematic diagram illustrating the structure of the coupled HBM-ERGOM model system Figure B. 120 year simulation run Figure C. The FISHRENT model diagram, here applied to Kattegat and Western Baltic. Figure D. One-year cycle of Chl-a in the different polygons Figure E. Relative biomass–initial condition values compared with simulation outcome Figure F. Diet composition of all predators Figure G. Biomass per age group over time for all vertebrates Figure H. Demography distribution for all vertebrates—the number of individuals for each age group Figure I. Geographical distribution of all functional groups Figure J. Geographical distribution of oxygen in the different layers. Panel 1 = top layer, panel 7 = bottom layer Figure K. Total biomass of Cod for scenario 1 (baseline) compared to scenario 5 Figure L. Relative prey biomass for predator cod, baseline compared to scenario 5 Figure M. One-year cycle of nutrients in the different polygons Table A. Physical and geochemical parameters used to internally force the Baltic Atlantis model. Table B. Summary of riverine + direct point source waterborne nitrogen loads applied to the Baltic Atlantis grid based on information from the Review of the Fifth Baltic Sea Pollution Load Compilation for the 2013 HELCOM Ministerial Meeting (HELCOM PLC-5.5). Nitrogen fractionation between DIN and DON based on Savchuk et al. (2012). Bioavailable fraction of DON assumed equal to labile DON as in Savchuk and Wolff (2009). Coastal retention fractions from Savchuk and Wolff (2009). Table C. Summary of key sources used to inform the biological module of Baltic Atlantis in relation to abundance and biomass, demography, prey-predator interaction and other functions. Ta [file pone.0199168.s001.zip › FigM.pdf]
